# Axially Chiral 2-Hydroxybiaryls by Palladium-Catalyzed Enantioselective C–H Activation

**DOI:** 10.1021/acscatal.3c03867

**Published:** 2023-10-16

**Authors:** Pablo Losada, Laura Goicoechea, José Luis Mascareñas, Moisés Gulías

**Affiliations:** Centro Singular de Investigación en Química Biolóxica e Materiais Moleculares (CIQUS) and Departamento de Química Orgánica, Universidade de Santiago de Compostela, 15782 Santiago de Compostela, Spain

**Keywords:** C−H activation, catalysis, palladium, enantioselective, atroposelective, phenol, naphthol, atropoisomers

## Abstract

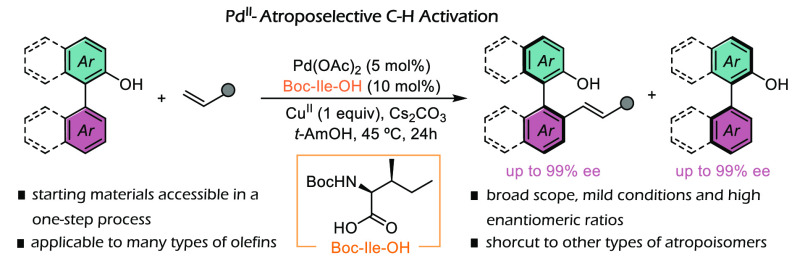

This article describes the discovery and development of a palladium-catalyzed asymmetric C–H olefination of 2-hydroxybiaryls. The strategy allows a direct assembly of optically active, axially chiral 2-substituted-2′-hydroxybiaryls from readily available precursors and demonstrates that the native hydroxy unit of the substrates can work as an efficient directing group for the C–H activation. This represents a substantial advantage over other approaches that require the preinstallation of metal coordinating units. The simplicity of the approach and versatility of the products allow a practical and efficient synthesis of a broad variety of optically active binaphthyl derivatives.

Past decades have witnessed a steady progress in the field of metal-catalyzed C–H activation/functionalization reactions.^1^ These methodologies are highly attractive from a synthetic perspective because they allow consideration of the C–H bond as a latent functional group. Even more appealing is the possibility of performing these reactions in an asymmetric fashion and, hence, obtaining valuable chiral products from nonfunctionalized racemic or achiral starting materials.^2^ In this context, there has been a growing interest in the use of enantioselective C–H bond functionalizations for producing axially chiral biaryls because these structures represent privileged scaffolds for catalysis and synthesis.^3^ Despite recent progress in this area, most of the examples so far described require the preinstallation of auxiliary directing groups for the C–H activation, such as pyridines, sulfoxides, thioethers, phosphine oxides, pyridine oxides, amides or aldehydes, most of which are not needed for the subsequent exploitation of the chiral products.^4^ Indeed, the presence of these groups restricts the utility of the resulting atropoisomers for the synthesis of biorelevant products or for the preparation of chiral ligands and catalysts.

Therefore, the development of methods that allow direct metal-catalyzed atroposelective C–H activation/functionalization in precursors with “native” functional groups, like free amines, alcohols, or carboxylic acids, is of major relevance. However, progress in this matter has been slow. To the best of our knowledge, the only two examples reported so far are limited to arylamine precursors. Specifically, these articles deal with an enantioselective desymmetrization of *ortho*-arylanilines using a chiral phosphoric acid (CPA) as metal ligand^5a^ or with a remote *meta*-C–H arylation of 2-arylanilines on the basis of a relay strategy using a chiral norbornene as transient mediator.^5b^

Considering the relevance of axially chiral biaryl systems featuring *ortho*-hydroxy groups in one of the rings, either for natural product synthesis^6^ or as immediate precursors of metal ligands or organocatalysts (Figure 1a),^7^ the development of direct asymmetric approaches to these frameworks is highly desirable. Moreover, the hydroxyl group can also be readily converted into other functionalities, which further stresses the relevance of this type of scaffold.

**Figure 1:**
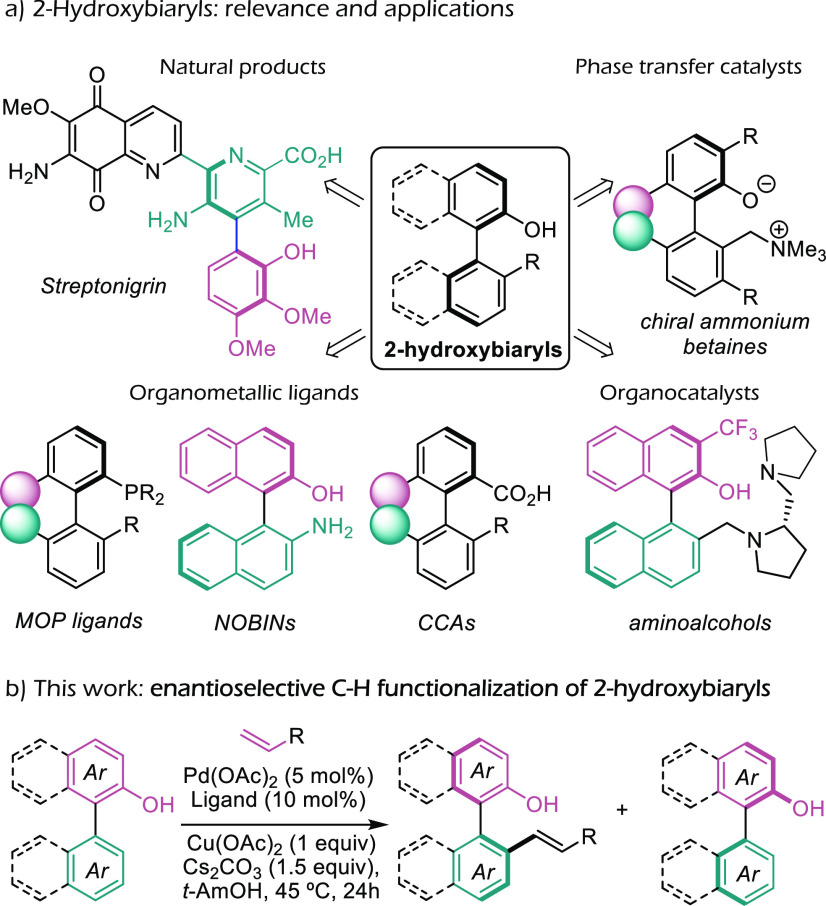
(a) Chiral *ortho*-hydroxyaryl scaffolds as versatile precursors for different applications. (b) This work: asymmetric resolution of *ortho*-hydroxybinaphthyls and related atropoisomer precursors.

Curiously, despite the abundance of commercially available phenol and naphthol precursors, atroposelective approaches to chiral 2-hydroxy-1,1-biaryl products using C–H bond functionalization reactions have not been reported. This lack of reports is likely due to the assumption that the hydroxyl group is not a suitable directing group for enantioselective metal-promoted C–H activations and that its small size may hamper the obtention of configurationally stable, optically active atropoisomers. In this context, there are some isolated examples of olefination of *ortho*-aryl phenols via C–H activation; however, they tend to give mixtures of products and have never been implemented in an asymmetric fashion.^8^

It is also worthwhile to note that other strategies to build optically active 2′-hydroxylbiaryl scaffolds are also very scarce, usually require multistep processes, and are essentially limited to binaphthyl structures.^9^ This is the case for ring-opening methods based on alkylative cross-couplings.^10^

Therefore, in line with our ongoing work on metal-catalyzed asymmetric C–H functionalization reactions,^11^ herein, we report a simple and practical asymmetric approach to optically active 2-substituted-2′-hydroxyl-biaryls through the kinetic resolution of 2-arylnaphthols and 2-arylphenols (Figure 1b). The strategy is implemented in a palladium-catalyzed C–H olefination reaction, which is of further interest because of the versatility of the double bond for the ensuing manipulations. The reactions show good enantioselectivities and broad scope, and the methodology allows the access of highly valuable products in an enantioenriched manner.

Our research started with racemic naphthol **1a** that was synthesized in just one step from inexpensive commercial sources and with methyl acrylate as the alkene partner. The initial screening (see the Supporting Information for more details) revealed *tert*-amyl alcohol as the preferred solvent for the reaction, copper(II) acetate as the oxidant, and cesium carbonate as the base. Using these conditions, 10 mol % palladium acetate, and 30 mol % of *N*-protected amino acids as ligands, we observed the formation of the desired product under mild temperatures (45 °C).^12^ The detailed results are included in Table 1 (entries 1–10). The best results in terms of yield and enantioselectivities were observed using Boc-protected isoleucine as ligand, which allowed the obtainment of high enantiomeric ratios of both the product and the starting material (entry 5). NOBINAc ligands led to poor results in these atroposelective transformations (entries 11 and 12).^11a^ Remarkably, the amount of the palladium source could be reduced to 5 mol %, and the ligand could be reduced to 10 mol %. Indeed, under these conditions and through the use of only 1 equiv of the acrylate partner and of copper acetate, the desired product could be isolated in an excellent 96:4 ratio (**3aa**) while the starting precursor **1a** was recovered in 97:3 er (entry 13, selectivity factor *s* = 85). The absolute configuration of the product was confirmed by X-ray structural determination.

**Table 1:**
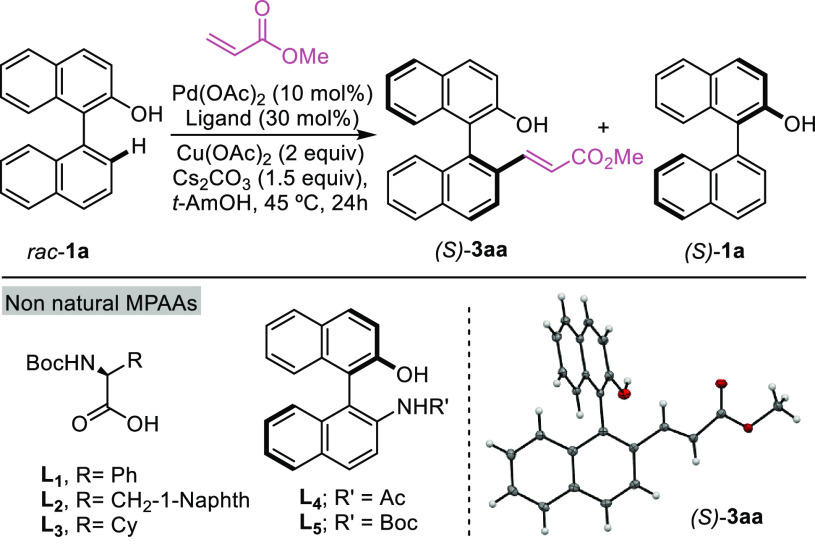
Optimization of Conditions

entry	deviation from above conditions^a^	yield (%) **3aa/1a**	er **3aa**	er **1a**
1	Boc-Leu-OH	40/45	95.5:4.5	87:13
2	Boc-*t*-Leu-OH	34/47	95:5	80:20
3	Boc-Phe-OH	42/40	95:5	93:7
4	Boc-Val-OH	43/40	95:5	92:8
5	Boc-Ile-OH	49/41	95.5:4.5	96.6:3.5
6	L1	21/69	92:8	60:40
7	L2	47/46	95:5	91:9
8	L3	47/39	94:6	97:3
9	Ac-Phe-OH	10/73	90:10	54:46
10	Ac-Ile-OH	6/83	79:21	51:49
11^b^	L4	20/70	56:44	52:48
12	L5	16/64	57:43	51:49
13^c^	Boc-Ile-OH	50/42	96:4	97:3

a Conditions found in the table scheme.

b Temp = 100 °C.

c Optimized conditions: *rac*-**1a** (0.1 mmol), **2a** (0.1 mmol), Pd(OAc)_2_ (5 mol %), ligand (10 mol %), Cu(OAc)_2_·H_2_O (1 equiv), Cs_2_CO_3_ (1.5 equiv), *t*-amylOH (0.1 M), air, 45 °C, 24 h.

With the optimized conditions in hand, we studied the scope with respect to the alkene partner. We were pleased to find that alkenes equipped with other electron-withdrawing substituents, such *tert*-butyl ester, phosphonate, or amides, were also effective, and the reaction led to the expected products with enantiomeric ratios up to 95.5:4.5 (**3ab**, **3ac**, and **3ad**) and up to 96:4 for the starting materials (Table 2).

**Table 2:**
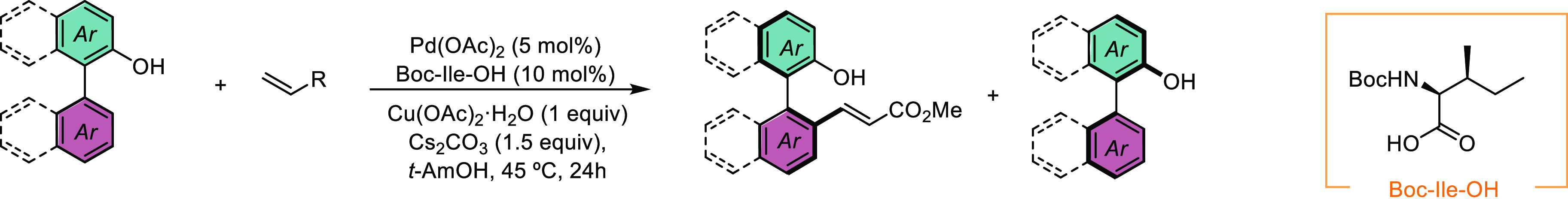

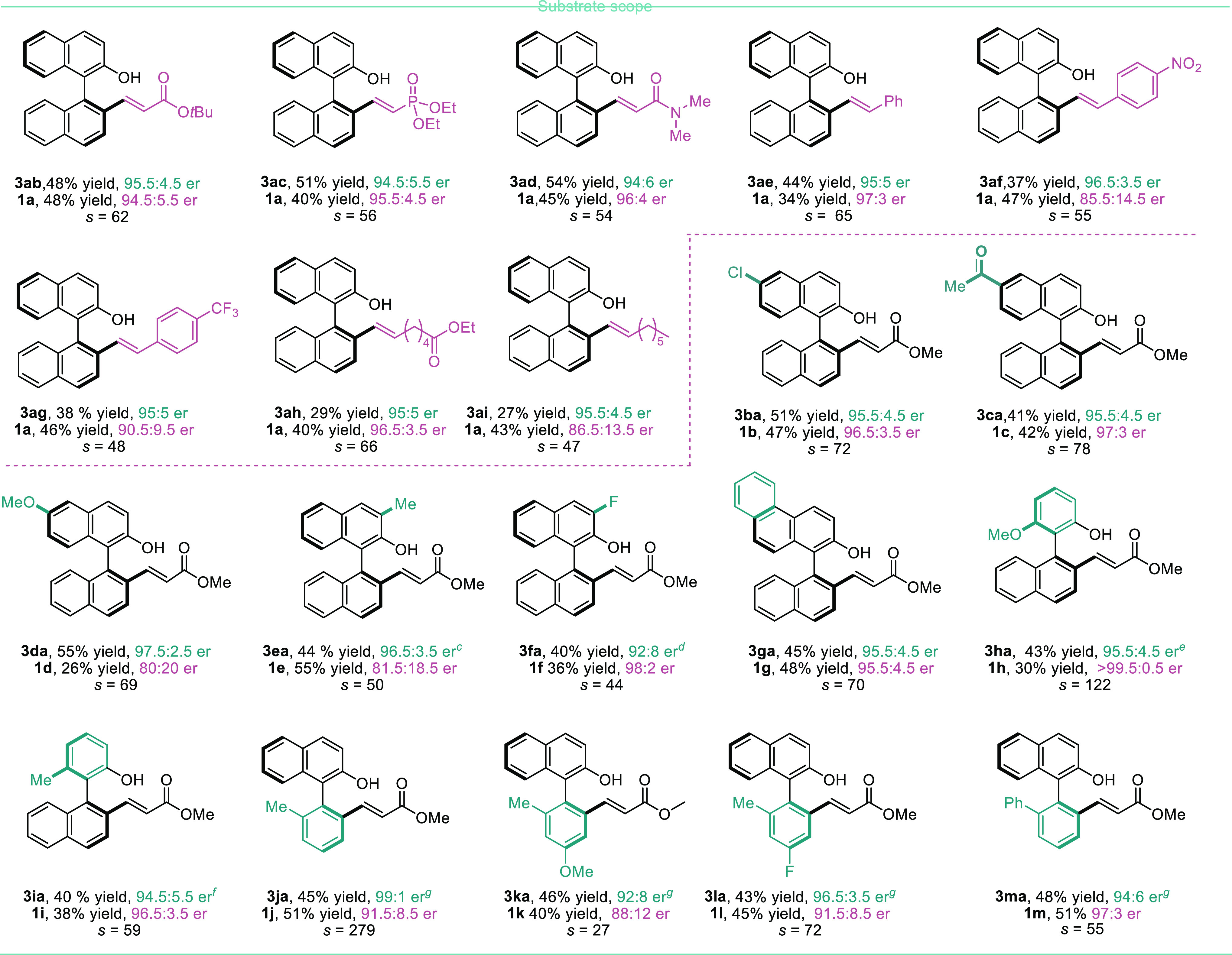
Scope^a^^,^^b^

a Reaction conditions: *rac*-**1a** (0.1 mmol), **2a** (0.1 mmol), Pd(OAc)_2_ (5 mol %), Boc-Ile-OH (10 mol %), Cu(OAc)_2_·H_2_O (1 equiv), Cs_2_CO_3_ (1.5 equiv), *t*-AmylOH, (0.1 M), air, 45 °C, 24 h.

b Selectivity (*s*) = ln[(1 – *C*)(1 – ee^SM^)]/ln[(1 – *C*)(1 + ee^SM^)]. Calculated conversion (*C*) = ee^SM^/(ee^SM^ + ee^PR^).

c Reaction time of 48 h.

d Conducted at rt for 17 h.

e Boc-Val-OH as ligand, 50 °C, 26 h.

f Conducted at 60 °C for 48 h.

g Conducted at 60 °C for 24 h.

Styrene-type alkenes are also well tolerated, and therefore, the product **3ae** was formed with enantiomeric ratios up to 95:5, whereas the starting material **1a** was recovered with 97:3 er. Partners with electron-withdrawing groups in the phenyl moiety of the styrene were also effective (**3af** and **3ag**, up to 96:5:3.5 er).

Interestingly, alkyl-substituted olefins, which tend to be more reluctant partners in other C–H olefination reactions, were also found to be effective in our reaction. Therefore, products **3ah** and **3ai** were isolated with up to 95.5:4.5 er, and the starting precursors were recovered with up to 96.5:3.5 er. The lower yields observed are likely due to a slower reaction, which leads to a higher degradation of the starting materials.

We also analyzed the scope regarding the biaryl component. Gratifyingly, the reaction tolerates different functionalities on the naphthol ring, including chlorine, methoxy, ketone, methyl, and fluorine. The enantiomeric ratios for the functionalized products (**3ba**–**3fa**) were up to 97.5:2.5, while good enantiomeric ratios of up to 98:2 were also found for the starting materials. In the case of precursor with a fluorine atom in the 3-position of the naphthol ring, we found a strong accelerating effect, probably due to the increased acidity of the phenol. Indeed, in this case, the reaction proceeds even at room temperature.

We could also replace the naphthol ring for other aromatic systems, such as anthracenol or phenol derivatives, and thus, products **3ga, 3ha**, and **3ia** were prepared with enantiomeric ratios up to 95.5:4.5. Similarly, the bottom naphthyl group can be replaced by *ortho*-substituted aryl rings to give the expected products, like **3ja**–**3ma**, with good enantiomeric ratios up to 99:1 and remarkable selectivity up to 279. It is also important to highlight that many of the precursors used in the above resolution processes are accessible from commercially available materials in just one step. Therefore, the route to the chiral products involves only two steps (see the Supporting Information).

Overall, these results not only confirm the success of the strategy with binaphthyl systems but also with other type of racemic hydroxyaryl precursors, which had been much less explored in other asymmetric processes.^13^ Importantly, the methoxy derivative **Me-1a** is unreactive under the reaction conditions (Figure 2, eq 1), which confirms the critical role of the hydroxyl group in promoting the process.

**Figure 2:**
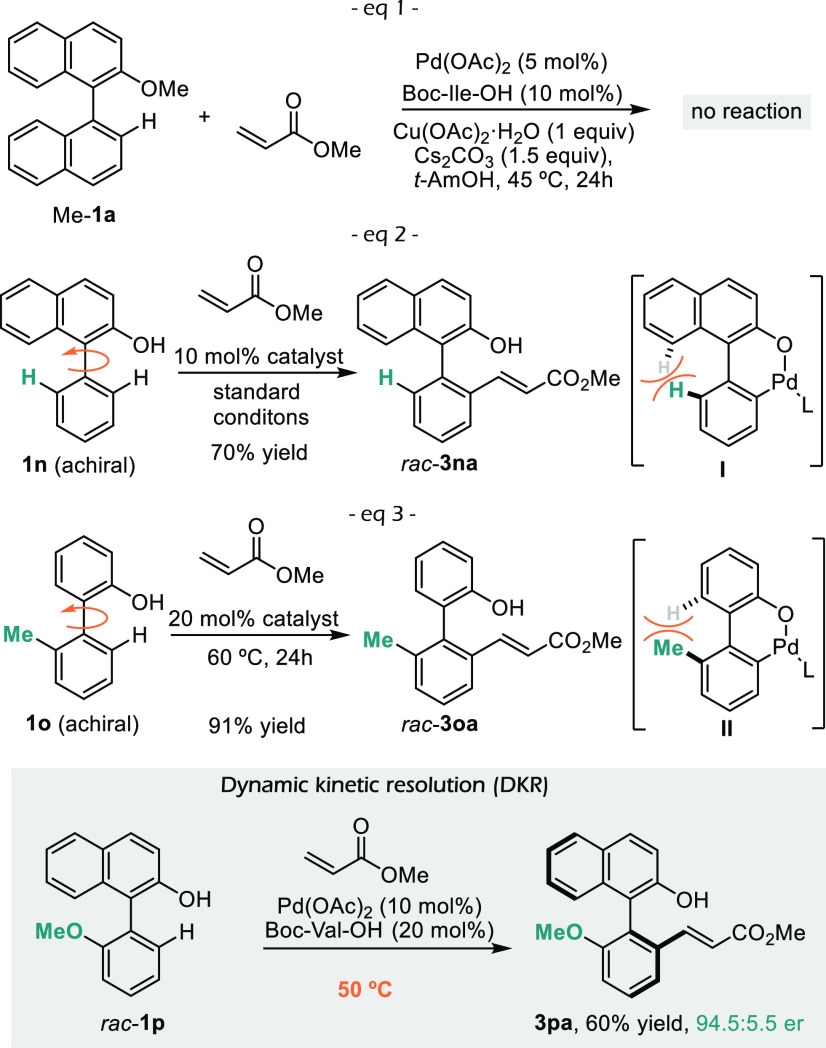
Control experiments and dynamic kinetic resolution of 1-arylnaphth-2-ols.

We also explored the viability of enantioselective desymmetrizations with achiral precursors **1n** and **1o**. Unfortunately, the olefinated products were obtained as racemic mixtures, likely because of the low rotation kinetic barriers in metallacycle intermediates **I** and **II** (Figure 2, eq 2 and 3).^9a^ Fortunately, it is possible to perform dynamic kinetic resolutions in substrates like *rac*-**1p**, which can atropisomerize at 50 °C. Therefore, the product **3pa** was obtained in 60% yield and 94.5:5.5 enantiomeric ratio (Figure 2).

The enantioselective alkenylation protocol could be scaled up to grams without a detrimental effect on the reaction yield (Figure 3). For example, the chiral olefination of **1j** (prepared in one step using a Suzuki coupling from cheap and commercially available materials) at a 8 mmol scale provided 1.19 g of compound (*S*)-**3ja** and 0.75 g of (*S*)-**1j** with exceptional enantiomeric ratios (99.9:0.1 for the product and 96.5:3.5 for the starting material).

**Figure 3:**
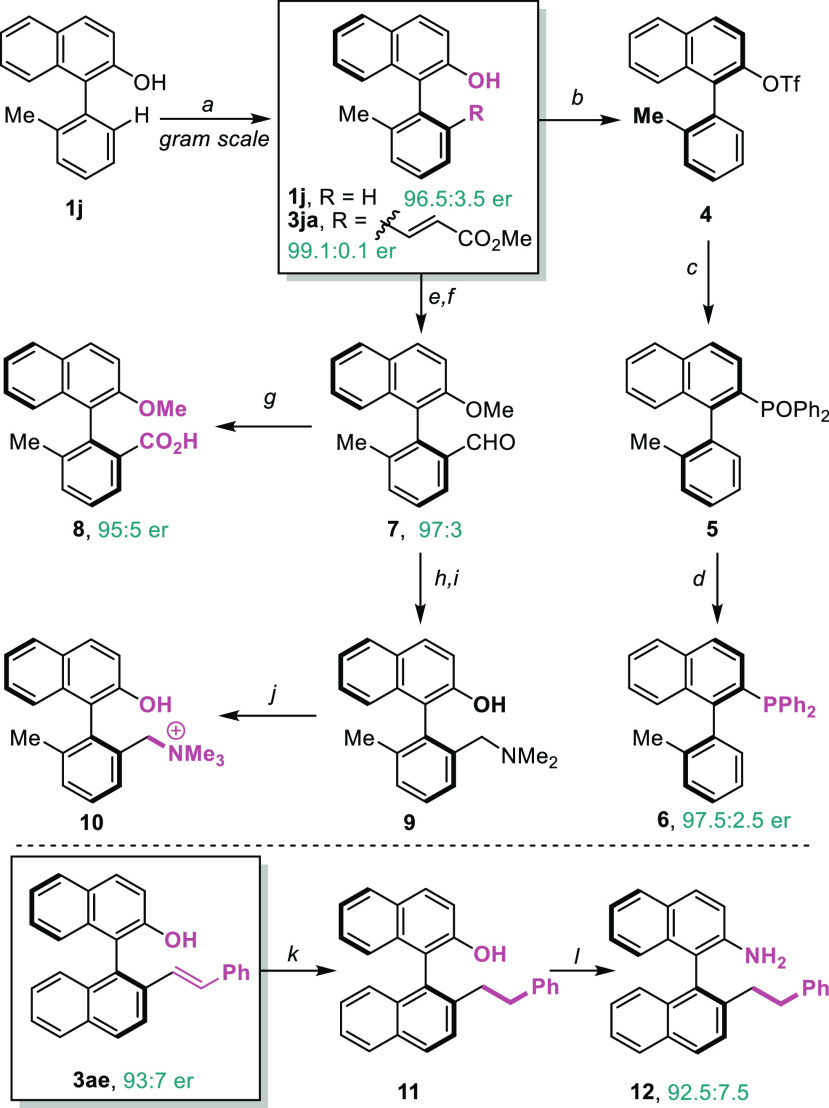
Gram-scale reaction and derivatizations of the products. Conditions: (a) Conditions in Table 2. (b) From **1j**: Tf_2_O, Et_3_N, CH_2_Cl_2_, −78 °C to rt, 87% yield. (c) HP(O)Ph_2_, Pd(OAc)_2_/dppb, DIPEA, DMSO, 100 °C, 78% yield. (d) HSiCl_3_, Et_3_N, *p*-xylene, 120 °C, 75% yield. (e) From **3ja**: MeI, K_2_CO_3_, acetone, 30 °C, 77% yield. (f) K_2_OsO_4_·2H_2_O, NaIO_4_, THF/H_2_O, rt, 59% yield. (g) NaClO_2_, NaH_2_PO_4_·2H_2_O, H_2_O_2_, THF/*t*-BuOH/H_2_O, rt, 81% yield. (h) NaBH(OAc)_3_, NHMe_2_·HCl, NaOAc/AcOH, THF, rt. (i) BBr_3_, CH_2_Cl_2_, 0 °C to rt, 43% yield. (j) MeI, MeCN, rt, 67% yield. (k) Pd/C, H_2_, MeOH/AcOEt, rt, 67% yield. (l) 2-Bromopropionamide, KI/K_2_CO_3_, DMSO, 80 °C; then, NaOH, 150 °C, 59% yield.

Importantly, the enantioenriched 1-arylnaphth-2-ols can be transformed into a variety of useful products using simple manipulations thanks to the versatility of the hydroxyl and/or the olefin units of the molecules. Thus, the enantioenriched naphthol (*S*)-**1j** was transformed into very appealing chiral monodentade phosphine (MOP) **6**, in a three-step process involving basic triflation, a palladium-catalyzed coupling, and a reduction step. All of these transformations do not convey the loss of optical purity.

The olefinated product **3ja** was transformed into the chiral aldehyde **7** by methylation and oxidative rupture. This compound can be transformed into the chiral carboxylic acid **8** by oxidation or into the prebetaine (**10**), which might be highly valuable in phase-transfer catalysis, using a reductive amination protocol. With the olefinated product **3ae**, we confirmed that it can be readily hydrogenated to give the expected alkyl derivative, which can be further used for the formation of an aniline analogue in a one-pot sequence. It is worthwhile to note that these binaphthyl systems could not be accessed by desymmetrization of *ortho*-biaryl anilines.^5a^ Overall, these preliminary manipulations illustrate the versatility of the products to produce a variety of enantioenriched products exhibiting axial chirality and different types of substitutions. It is important to note that most of these products are difficult to build by using other asymmetric methodologies.

In conclusion, we have reported the first examples of enantioselective C–H functionalizations of *ortho*-hydroxy binaphthyl or arylnaphthyl precursors. The Pd-catalyzed enantioselective olefination tolerates an important range of functional groups and presents a broad scope with respect to the phenol and alkenyl coupling partners. The use of a kinetic resolution strategy is advantageous in terms of obtaining both highly valuable alkenylated products and unreacted biaryls with high enantioselectivity. The chiral materials can be easily transformed into a large variety of useful derivatives because of the versatility of the hydroxyl or alkenyl group.

## Supporting Information Available

Experimental details and characterization data for all new compounds (PDF)
CIF data of (*S*)-**3aa** (CIF)

## Supplementary Material

cs3c03867_si_001.pdf

cs3c03867_si_002.cif
