# Supplementary material for: Axially Chiral 2-Hydroxybiaryls by Palladium-Catalyzed Enantioselective C–H Activation
Source: ACS Catal. 2023 Oct 16;13(21):13994–9. doi: 10.1021/acscatal.3c03867 (PMC10629138; doi:10.1021/acscatal.3c03867)

Supporting Information

Axially Chiral 2-Hydroxybiaryls by Palladium-Catalyzed Enantioselective C-H Activation

Pablo Losada, Laura Goicoechea, José Luis Mascareñas*, Moisés Gulías*

e-mail: joseluis.mascarenas@usc.es, moises.gulias@usc.es

Centro Singular de Investigación en Química Biológica y Materiales Moleculares (CiQUS) and Departamento de Química Orgánica, Universidad de Santiago de Compostela, 15782 Santiago de Compostela, Spain.

Table of contents

1. General experimental information **1**

2. Obtention of 1-bromo-2-naphthols, 2-bromophenols and alkenes **2**

3. Synthesis of starting materials **3**

3.1. General Suzuki coupling procedure 3

3.2. Synthesis of 3-fluoro-[1,1'-binaphthalen]-2-ol (*rac*-1f) 8

3.3. Synthesis of 1-([1,1'-biphenyl]-2-yl)naphthalen-2-ol (*rac*-1m) 10

3.4. Synthesis of 2'-methyl-[1,1'-biphenyl]-2-ol (1o) 11

3.5. Synthesis of 1-(2-methoxyphenyl)naphthalen-2-ol (*rac*-1p) 12

4. Kinetic resolution of biaryl alcohols through Pd(II)-catalyzed alkenylation **13**

4.1. Optimization of the reaction conditions 13

4.1.1. Temperature and time optimization 13

4.1.2. Chiral ligand screening 14

4.1.3. Solvent screening 15

4.1.4. Oxidant screening 16

4.1.5. Loading studies 17

4.2. Reaction scope 18

4.3. Gram scale kinetic resolution of *rac*-1j 62

4.4. Mechanistic experiments 65

4.4.1. Synthesis of the deuterated starting material (d_7-1a) 65

4.4.2. Measurement of the KIE by parallel test method 66

5. Other reactions **67**

5.1. Attempt of desymmetrization of 1-phenyl-2-naphthol (1n) 67

5.2. Attempt of atroposelective alkenylation of 2'-methyl-[1,1'-biphenyl]-2-ol (1o) . 69

5.3. Dynamic kinetic resolution through Pd(II)-catalyzed C-H alkenylation 71

6. Synthetic applications of the enantioenriched biaryls **73**

6.1. Triflation 73

6.2. C-P cross coupling 74

6.3. Phosphine oxide reduction 76

6.4. Methylation and oxidative cleavage 78

6.5. Oxidation of the aldehyde to the carboxylic acid 80

6.6. Reductive amination and hydroxyl deprotection 82

6.7. Quaternarization of the amine 84

6.8. Hydrogenation of the double bond 85

6.9. Amine-alcohol exchange by Smiles rearrangement 87

7. Mechanistic proposal and stereomodel for the C-H activation step **89**

8. NMR Spectra **90**

1. General experimental information

Dry solvents were obtained from Across Organics, Extra Dry over Molecular Sieves, and used without further purification. $Pd(OAc)_2$ (98%) [3375-31-1] was obtained from Strem. All other chemicals were purchased from Sigma Aldrich, Acros Organics, Alfa Aesar, Fluorochem, TCI Chemical, Fluka or BLD Pharm, and were used as received; unless NEt_3 and DIPEA that were distilled over CaH_2 and ninhydrin and CaH_2, respectively. Inert-atmosphere reactions were carried out with dry solvents in flame-dried flasks. All palladium-catalyzed C-H alkenylations were carried without precautions to elude moisture or oxygen. The abbreviation "rt" refers to a temperature between 20-25 ºC. Reaction mixtures were stirred using Teflon-coated magnetic stir bars. Thin layer chromatography (TLC) was carried out on pre-coated silica gel F_{254} plates with visualization under UV light or by dipping the plate into *p*-anisaldehyde or ceric ammonium molybdate solutions followed by heating. Column chromatography was performed on silica gel (40-60 µm) unless otherwise stated. NMR data was collected on Varian Mercury 300 MHz or Bruker AVIII 500 MHz spectrometers. Chemical shifts are given in ppm (δ) and are referenced to the residual solvent ($CHCl_3$ or DMSO). NMR data was analyzed using MestReNova NMR data processing software (http://mestrelab.com/). High Resolution Mass Spectra (HRMS) were performed at the CACTUS facility of the University of Santiago de Compostela on a Bruker micrOTOF spectrometer. Enantiomeric ratios (er) were determined on an Agilent HPLC 1100 Series using commercially available chiral columns. All racemic products were prepared under the same procedure than the chiral ones but with the employment of a racemic mono-protected amino acid (Boc-DL-Phe-OH) as ligand. X-ray crystallographic analysis of compound **3aa** was performed at the CACTI facility of the University of Vigo on a Bruker D8 Venture Photon II CMOS apparatus and the absolute stereochemistry of all compounds was assigned by analogy.

2. Obtention of 1-bromo-2-naphthols, 2-bromophenols and alkenes

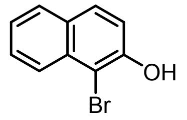

S1

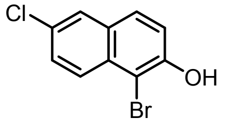

S2

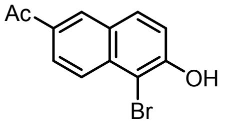

S3

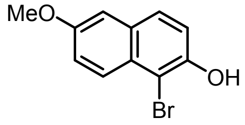

S4

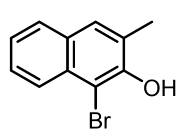

S5

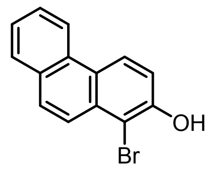

S6

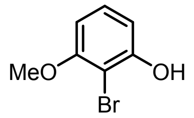

S7

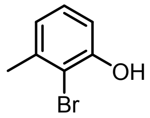

S8

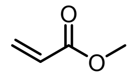

2a

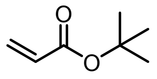

2b

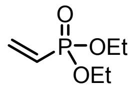

2c

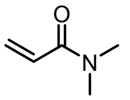

2d

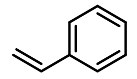

2e

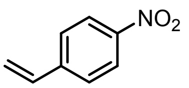

2f

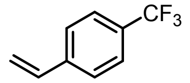

2g

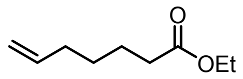

2h

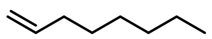

2i

Compounds **S1**, **S7** and **S8** were bought to the corresponding commercial source. **S2** and **S4** were prepared by direct bromination of the corresponding commercially available naphthols following a reported procedure.[1] **S3** was synthesized from 6-methoxy-2-acetylnaphthalene by a reported methyl cleavage method[2] followed by the above-mentioned bromination method. **S5** was prepared from 2-bromo-3-methoxynaphthalene following a reported procedure[3] followed by bromination. **S6** was synthesized from 2-acetylphenanthrene following a reported 3-step synthetic route.[4]

All alkenes **2a-2i** were purchased to the corresponding commercial source unless **2g** that was synthesized according to a literature procedure.[5]

---

[1] Zuo, Z.; Wang, H.; Fan, L.; Liu, J.; Wang, Y.; Luan, X. *Angew. Chem. Int. Ed.* **2017**, *56*, 2767-2771.
[2] Yao, W.; Yan, Y.; Xue, L.; Zhang, C.; Li, G.; Zheng, Q.; Zhao, Y. S.; Jiang, H.; Yao, J. *Angew. Chem. Int. Ed.* **2013**, *52*, 8713-8717.
[3] Dyadyuk, A.; Vershinin, V.; Shalit, H.; Shalev, H.; More, N. Y.; Pappo, D. *J. Am. Chem. Soc.* **2022**, *144*, 3676-3684.
[4] Barbasiewicz, M.; Szadkowska, A.; Makal, A.; Jarzembska, K.; Woźniak, K.; Grela, K. *Chemistry – A European Journal* **2008**, *14*, 9330-9337.
[5] Zhang, J.; Tang, Y. *Adv. Synth. Catal.* **2016**, *358*, 752-764.

3. Synthesis of starting materials

3.1. General Suzuki coupling procedure

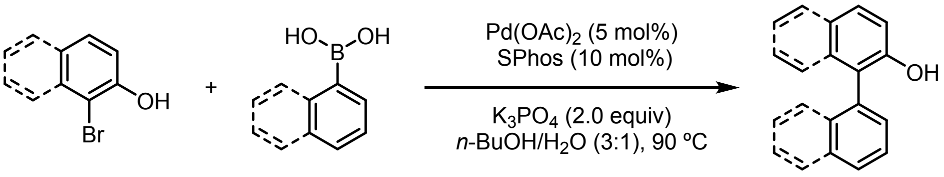

1.1 equiv

rac-**1**

A solution of the appropriate 1-bromo-2-naphthol or 2-bromophenol (1.0 equiv), arylboronic acid (1.1 equiv), $Pd(OAc)_2$ (5.0 mol%), SPhos (10 mol%) and K_3PO_4 (2.0 equiv) in *n*-BuOH (0.20 M) and water (0.67 M) was degassed by bubbling an argon steam and then stirred at 90 ºC until full conversion was observed by TLC (typically less than 30 min is enough). After cooling to rt, the reaction mixture was partitioned between AcOEt and water. The layers were separated, and the aqueous phase was extracted with AcOEt (x2). The combined organic phase was washed with brine, dried over $MgSO_4$, and concentrated under reduced pressure (55-60 ºC). The resulting residue was purified by flash column chromatography (AcOEt/hexane) to afford the desired biaryl alcohol.

[1,1'-binaphthalen]-2-ol (*rac*-1a)

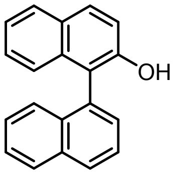

rac-**1a**

rac-**1a** was obtained after column chromatography (AcOEt/hexane 6:94 to 9:91) as a white solid (1.08 g, 89%). **Rf:** 0.60 (AcOEt/hexane 25:75, brown in *p*-anisaldehyde). **^{1}H NMR** (300 MHz, $CDCl_3$) δ: 8.04 (dd, J = 11.6, 8.0 Hz, 2H), 7.94 (dd, J = 11.9, 8.5 Hz, 2H), 7.69 (t, J = 7.6 Hz, 1H), 7.63 – 7.53 (m, 2H), 7.46 (d, J = 8.2 Hz, 1H), 7.43 – 7.34 (m, 3H), 7.29 (t, J = 7.6 Hz, 1H), 7.17 (d, J = 8.4 Hz, 1H), 5.00 (s, 1H). **^{13}C NMR** (75 MHz, $CDCl_3$) δ: 151.1 (COH), 134.3 (C), 134.0 (C), 133.0 (C), 131.6 (C), 130.0 (CH), 129.8 (CH), 129.3 (CH), 129.1 (C), 128.6 (CH), 128.1 (CH), 127.0 (CH), 126.7 (2CH), 126.1 (CH), 125.9 (CH), 125.1 (CH), 123.5 (CH), 118.9 (C), 117.6 (CH). **HRMS** (APCI+) *m/z* calcd. for $C_{20}H_{15}O$ [M+H]: 271.1117; found: 271.1108.

2-methoxy-1,1'-binaphthalene (Me-1a)

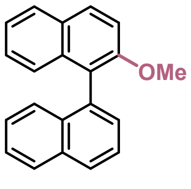

Me-**1a**

Me-**1a** was obtained from 1-iodo-2-methoxynaphthalene after column chromatography (AcOEt/hexane 3:97) as a white solid (128 mg, 90%). **Rf:** 0.53 (AcOEt/hexane 10:90, grayish blue in *p*-anisaldehyde). **^{1}H NMR** (300 MHz, $CDCl_3$) δ 8.02 – 7.93 (m, 3H), 7.88 (d, J = 8.1 Hz, 1H), 7.63 (t, J = 7.6 Hz, 1H), 7.45 (dd, J = 8.2, 3.3 Hz, 3H), 7.37 – 7.22 (m, 4H), 7.18 (t, J = 8.5 Hz, 1H), 3.77 (s, 3H). **^{13}C NMR** (75 MHz, $CDCl_3$) δ: 154.8 (COMe),

134.7 (C), 134.4 (C), 133.9 (C), 133.1 (C), 129.6 (CH), 129.2 (C), 128.6 (CH), 128.4 (CH), 127.9 (CH), 127.9 (CH), 126.5 (CH), 126.3 (CH), 126.0 (CH), 125.8 (CH), 125.7 (CH), 125.7 (CH), 123.7 (CH), 123.5 (C), 114.1 (CH), 57.0 (OCH_3). **HRMS** (APCI+) *m/z* calcd. for $C_{21}H_{17}O$ [M+H]: 285.1274; found: 285.1269.

6-chloro-[1,1'-binaphthalen]-2-ol (*rac*-1b)

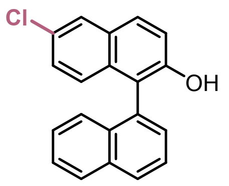

rac-**1b**

rac-**1b** was obtained after column chromatography (AcOEt/hexane 5:95 to 10:90) as a white solid (311 mg, 68%). **Rf:** 0.50 (AcOEt/hexane 25:75, brown in *p*-anisaldehyde). **^{1}H NMR** (300 MHz, $CDCl_3$) δ: 8.04 (d, *J* = 7.0 Hz, 1H), 7.99 (d, *J* = 8.2 Hz, 1H), 7.88 – 7.77 (m, 2H), 7.67 (t, *J* = 7.5 Hz, 1H), 7.58 – 7.50 (m, 2H), 7.40 – 7.31 (m, 3H), 7.17 (dd, *J* = 9.0, 2.2 Hz, 1H), 7.03 (d, *J* = 9.0 Hz, 1H), 4.93 (s, 1H). **^{13}C NMR** (75 MHz, $CDCl_3$) δ: 151.4 (COH), 134.4 (C), 132.8 (C), 132.4 (C), 131.0 (C), 129.8 (CH), 129.7 (C), 129.7 (CH), 129.3 (C), 129.1 (CH), 128.7 (CH), 127.5 (CH), 127.2 (CH), 126.8 (2CH), 126.8 (CH), 126.2 (CH), 125.7 (CH), 119.2 (C), 118.8 (CH). **HRMS** (APCI+) *m/z* calcd. for $C_{20}H_{13}ClO$ [M+H]: 304.0655; found: 304.0644.

6-acetyl-[1,1'-binaphthalen]-2-ol (*rac*-1c)

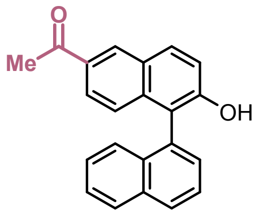

rac-**1c**

rac-**1c** was obtained after column chromatography (AcOEt/hexane 25:75 to 40:60) as a cream powder (196 mg, 50%). **Rf:** 0.50 (AcOEt/hexane 40:60, red in *p*-anisaldehyde). **^{1}H NMR** (300 MHz, DMSO-d_6) δ: 9.98 (s, 1H), 8.64 (d, *J* = 2.0 Hz, 1H), 8.13 (d, *J* = 8.9 Hz, 1H), 8.02 (d, *J* = 8.3 Hz, 2H), 7.71 – 7.62 (m, 2H), 7.53 – 7.38 (m, 3H), 7.32 (ddd, *J* = 8.1, 6.7, 1.3 Hz, 1H), 7.18 (d, *J* = 7.4 Hz, 1H), 6.97 (d, *J* = 8.9 Hz, 1H), 2.63 (s, 3H). **^{13}C NMR** (75 MHz, DMSO-d_6) δ: 197.3 (CO), 155.1 (COH), 136.4 (C), 133.8 (C), 133.4 (C), 132.2 (C), 131.2 (C), 131.1 (CH), 130.7 (CH), 128.5 (CH), 128.2 (CH), 127.6 (CH), 126.7 (C), 126.0 (CH), 125.8 (2CH), 125.5 (CH), 124.4 (CH), 124.0 (CH), 119.2 (CH), 119.1 (C), 26.5 (CH_3). **HRMS** (APCI+) *m/z* calcd. for $C_{22}H_{17}O_2$ [M+H]: 313.1223; found: 313.1219.

6-methoxy-[1,1'-binaphthalen]-2-ol (*rac*-1d)

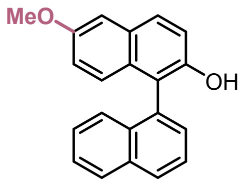

rac-**1d**

rac-**1d** was obtained after column chromatography (AcOEt/hexane 15:85) as a white solid (478 mg, 78%). **Rf:** 0.68 (AcOEt/hexane 20:80, dark green in *p*-anisaldehyde). **^{1}H NMR** (300 MHz, $CDCl_3$) δ: 8.02 (d, *J* = 8.2 Hz, 1H), 7.98 (d, *J* = 8.3 Hz, 1H), 7.81 (d, *J* = 8.9 Hz, 1H), 7.66 (t, *J* = 7.6 Hz, 1H), 7.58 – 7.49 (m, 2H), 7.44 – 7.30 (m, 3H), 7.21 (d, *J* = 2.6 Hz, 1H), 7.03 (d, *J* = 9.2 Hz, 1H), 6.93 (dd, *J* = 9.2, 2.6 Hz, 1H),

4.79 (s, 1H), 3.91 (s, 3H). **^{13}C NMR** (75 MHz, $CDCl_3$) δ: 156.0 (COMe), 149.5 (COH), 134.3 (C), 132.9 (C), 131.7 (C), 129.9 (C), 129.7 (CH), 129.3 (CH), 128.6 (CH), 128.6 (CH), 128.5 (C), 127.0 (CH), 126.7 (2CH), 126.1 (CH), 125.9 (CH), 119.2 (C), 119.1 (CH), 118.0 (CH), 106.5 (CH), 55.5 (OCH_3). **HRMS** (APCI+) *m/z* calcd. for $C_{21}H_{17}O_2$ [M+H]: 301.1223; found: 301.1234.

3-methyl-[1,1'-binaphthalen]-2-ol (*rac*-1e)

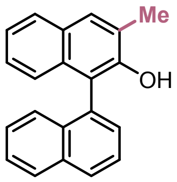

rac-**1e**

rac-**1e** was obtained after column chromatography (AcOEt/hexane 5:95) as a white solid (235 mg, 58%). **Rf:** 0.66 (AcOEt/hexane 20:80, brown in *p*-anisaldehyde). **^{1}H NMR** (300 MHz, $CDCl_3$) δ: 8.05 (d, *J* = 8.2 Hz, 1H), 8.00 (d, *J* = 8.4 Hz, 1H), 7.81 (d, *J* = 8.3 Hz, 1H), 7.77 (s, 1H), 7.72 – 7.64 (m, 1H), 7.59 – 7.51 (m, 2H), 7.44 – 7.29 (m, 3H), 7.19 (ddd, *J* = 8.2, 6.7, 1.4 Hz, 1H), 7.05 (d, *J* = 7.6 Hz, 1H), 4.99 (s, 1H), 2.53 (s, 3H). **^{13}C NMR** (75 MHz, $CDCl_3$) δ: 150.4 (COH), 134.4 (C), 133.0 (C), 132.8 (C), 132.0 (C), 129.8 (CH), 129.4 (CH), 129.4 (CH), 129.0 (C), 128.6 (CH), 127.4 (CH), 127.0 (C), 126.8 (CH), 126.7 (CH), 126.2 (CH), 126.0 (CH), 125.7 (CH), 124.9 (CH), 123.4 (CH), 118.4 (C), 17.2 (CH_3). **HRMS** (APCI+) *m/z* calcd. for $C_{21}H_{17}O$ [M+H]: 285.1274; found: 285.1271.

1-(naphthalen-1-yl)phenanthren-2-ol (*rac*-1g)

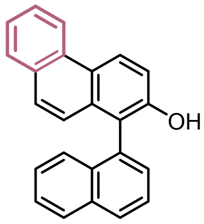

rac-**1g**

rac-**1g** was obtained after column chromatography (AcOEt/hexane 10:90) as a white powder (143 mg, 81%). **Rf:** 0.60 (AcOEt/hexane 20:80, dark brown in *p*-anisaldehyde). **^{1}H NMR** (300 MHz, $CDCl_3$) δ: 8.79 (d, *J* = 9.1 Hz, 1H), 8.72 (d, *J* = 8.3 Hz, 1H), 8.06 (d, *J* = 8.2 Hz, 1H), 8.01 (d, *J* = 8.2 Hz, 1H), 7.82 (d, *J* = 7.9 Hz, 1H), 7.74 – 7.65 (m, 2H), 7.62 – 7.47 (m, 5H), 7.45 – 7.32 (m, 2H), 7.09 (d, *J* = 9.2 Hz, 1H), 4.97 (s, 1H). **^{13}C NMR** (75 MHz, $CDCl_3$) δ: 152.0 (COH), 134.3 (C), 133.1 (C), 132.6 (C), 131.8 (C), 130.8 (C), 130.7 (C), 129.7 (CH), 129.5 (CH), 128.6 (2CH), 127.8 (CH), 127.1 (CH), 127.0 (CH), 126.8 (CH), 126.2 (CH), 125.9 (2CH), 124.8 (C), 124.6 (CH), 124.4 (CH), 122.4 (CH), 121.1 (C), 116.5 (CH). **HRMS** (APCI+) *m/z* calcd. for $C_{24}H_{17}O$ [M+H]: 321.1274; found: 321.1282.

3-methoxy-2-(naphthalen-1-yl)phenol (*rac*-1h)

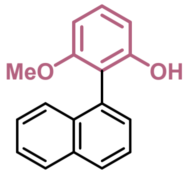

rac-**1h**

rac-**1h** was obtained after column chromatography (AcOEt/hexane 4:96) as colorless crystals (67.8 mg, 21%). **Rf:** 0.20 (AcOEt/hexane 10:90, orange in *p*-anisaldehyde). **^{1}H NMR** (500 MHz, $CDCl_3$) δ: 7.93 (t, *J* = 8.8 Hz, 2H), 7.59 (dd, *J* = 8.2, 7.0 Hz, 1H), 7.55 (d, *J* = 8.4 Hz, 1H), 7.51 (ddd, *J* = 8.2, 6.8, 1.2 Hz, 1H), 7.47 (dd, *J* = 6.9, 1.2 Hz, 1H), 7.42

(ddd, J = 8.3, 6.8, 1.3 Hz, 1H), 7.33 (t, J = 8.3 Hz, 1H), 6.74 (d, J = 8.3 Hz, 1H), 6.65 (d, J = 8.4 Hz, 1H), 4.71 (s, 1H), 3.65 (s, 3H). **^{13}C NMR** (126 MHz, $CDCl_3$) δ: 158.3 (COMe), 154.5 (COH), 134.2 (C), 132.6 (C), 130.1 (C), 129.7 (CH), 129.1 (CH), 129.0 (CH), 128.5 (CH), 126.7 (CH), 126.4 (CH), 126.0 (CH), 125.8 (CH), 115.2 (C), 108.5 (CH), 103.3 (CH), 56.0 (OCH_3). **HRMS** (APCI+) *m/z* calcd. for $C_{17}H_{14}O_2$ [M+H]: 251.1067; found: 251.1069.

3-methyl-2-(naphthalen-1-yl)phenol (*rac*-1i)

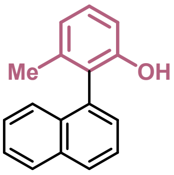

rac-**1i**

rac-**1i** was obtained after column chromatography (AcOEt/hexane 5:95 to 7:93) as a white powder (311 mg, 88%). **Rf:** 0.60 (AcOEt/hexane 25:75, pink in *p*-anisaldehyde). **^{1}H NMR** (300 MHz, $CDCl_3$) δ: 8.00 – 7.91 (m, 2H), 7.63 – 7.40 (m, 5H), 7.28 (t, J = 7.6 Hz, 1H), 6.98 – 6.90 (m, 2H), 4.56 (s, 1H), 1.95 (s, 3H). **^{13}C NMR** (75 MHz, $CDCl_3$) δ: 153.5 (COH), 138.4 (C), 134.3 (C), 132.8 (C), 132.3 (C), 129.1 (2CH), 128.6 (CH), 128.5 (CH), 127.0 (CH), 126.6 (CH), 126.1 (CH), 126.0 (C), 125.4 (CH), 122.1 (CH), 112.9 (CH), 20.2 (CH_3). **HRMS** (APCI+) *m/z* calcd. for $C_{17}H_{15}O$ [M+H]: 235.1117; found: 235.1112.

1-(*o*-tolyl)naphthalen-2-ol (*rac*-1j)

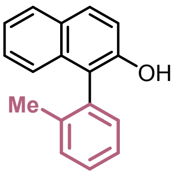

rac-**1j**

rac-**1j** was obtained using PhMe/H_2O (3:1, 0.38 M) as solvent after column chromatography (AcOEt/hexane 5:95 to 7:93) as a white powder (1.25 g, 89%). **Rf:** 0.65 (AcOEt/hexane 25:75, greyish blue in *p*-anisaldehyde). **^{1}H NMR** (300 MHz, $CDCl_3$) δ: 7.88 – 7.79 (m, 2H), 7.49 – 7.42 (m, 2H), 7.42 – 7.37 (m, 1H), 7.37 – 7.32 (m, 2H), 7.32 – 7.27 (m, 2H), 7.21 (dt, J = 6.2, 3.5 Hz, 1H), 4.93 (s, 1H), 2.05 (s, 3H). **^{13}C NMR** (75 MHz, $CDCl_3$) δ: 150.2 (COH), 139.1 (C), 133.2 (C), 133.2 (C), 131.7 (CH), 131.1 (CH), 129.6 (CH), 129.1 (CH), 128.2 (CH), 127.0 (CH), 126.7 (CH), 124.6 (CH), 123.4 (CH), 120.4 (C), 117.4 (CH), 19.7 (CH_3). **HRMS** (APCI+) *m/z* calcd. for $C_{17}H_{15}O$ [M+H]: 235.1117; found: 235.1111.

1-(4-methoxy-2-methylphenyl)naphthalen-2-ol (*rac*-1k)

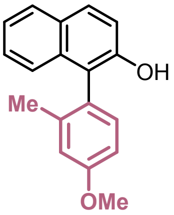

rac-**1k**

rac-**1k** was obtained after column chromatography (AcOEt/hexane 5:95) as a cream powder (219 mg, 55%). **Rf:** 0.58 (AcOEt/hexane 25:75, greyish blue in *p*-anisaldehyde). **^{1}H NMR** (300 MHz, $CDCl_3$) δ: 7.85 – 7.77 (m, 2H), 7.37 – 7.30 (m, 2H), 7.29 – 7.17 (m, 3H), 6.99 (d, J = 2.8 Hz, 1H), 6.93 (dd, J = 8.3, 2.7 Hz, 1H), 4.97 (s, 1H), 3.90 (s, 3H), 2.00 (s, 3H). **^{13}C NMR** (75 MHz, $CDCl_3$) δ: 160.2 (COMe), 150.6 (COH), 140.6 (C), 133.67 (C), 132.8 (CH), 129.4 (CH), 129.1 (C), 128.2 (CH), 126.6 (CH), 125.0 (C),

124.6 (CH), 123.4 (CH), 120.0 (C), 117.3 (CH), 116.6 (CH), 112.4 (CH), 55.5 (OCH_3), 20.0 (CH_3). **HRMS** (APCI+) *m/z* calcd. for $C_{18}H_{17}O_2$ [M+H]: 265.1123; found: 265.1218.

1-(4-fluoro-2-methylphenyl)naphthalen-2-ol (*rac*-1l)

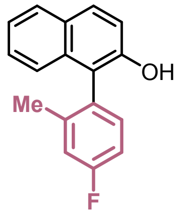

rac-**1l**

rac-**1l** was obtained after column chromatography (AcOEt/hexane 5:95 to 6:94) as a white powder (220 mg, 58%). **Rf:** 0.62 (AcOEt/hexane 25:75, grey in *p*-anisaldehyde). **^{1}H NMR** (300 MHz, $CDCl_3$) δ: 7.88 – 7.79 (m, 2H), 7.38 – 7.30 (m, 2H), 7.29 – 7.23 (m, 2H), 7.21 – 7.13 (m, 2H), 7.13 – 7.05 (m, 1H), 4.84 (s, 1H), 2.02 (s, 3H). **^{13}C NMR** (75 MHz, $CDCl_3$) δ: 164.9 (CF), 150.5 (COH), 141.8 (C), 133.4 (d, J = 8.3 Hz, CH), 133.3 (C), 129.8 (CH), 129.2 (C), 128.3 (CH), 126.9 (CH), 124.3 (CH), 123.6 (CH), 117.8 (d, J = 21.0 Hz, CH), 117.4 (CH), 117.4 (C), 113.9 (d, J = 21.0 Hz, CH), 19.9 (CH_3). **^{19}F NMR** (282 MHz, $CDCl_3$) δ: -113.5 (q, J = 8.7 Hz). **HRMS** (APCI+) *m/z* calcd. for $C_{17}H_{14}FO$ [M+H]: 253.1023; found: 253.1018.

1-phenylnaphthalen-2-ol (1n)

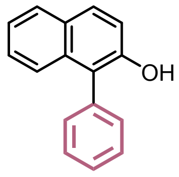

1n

1n was obtained after column chromatography (AcOEt/hexane 5:95 to 8:92) as a light-yellow solid (723 mg, 59%). **Rf:** 0.62 (AcOEt/hexane 25:75, garnet in *p*-anisaldehyde). **^{1}H NMR** (300 MHz, $CDCl_3$) δ: 7.86 – 7.76 (m, 2H), 7.59 (m, 2H), 7.53 (d, J = 6.9 Hz, 1H), 7.42 (m, 3H), 7.37 – 7.30 (m, 2H), 7.27 (m, 1H), 5.13 (s, 1H). **^{13}C NMR** (75 MHz, $CDCl_3$) δ: 150.3 (COH), 134.3 (C), 133.4 (C), 131.3 (2CH), 129.7 (2CH), 129.6 (CH), 129.0 (C), 128.6 (CH), 128.2 (CH), 126.6 (CH), 124.7 (CH), 123.4 (CH), 121.1 (C), 117.5 (CH). **HRMS** (APCI+) *m/z* calcd. for $C_{16}H_{13}O$ [M+H]: 221.0961; found: 221.0954.

3.2. Synthesis of 3-fluoro-[1,1'-binaphthalen]-2-ol (*rac*-**1f**)

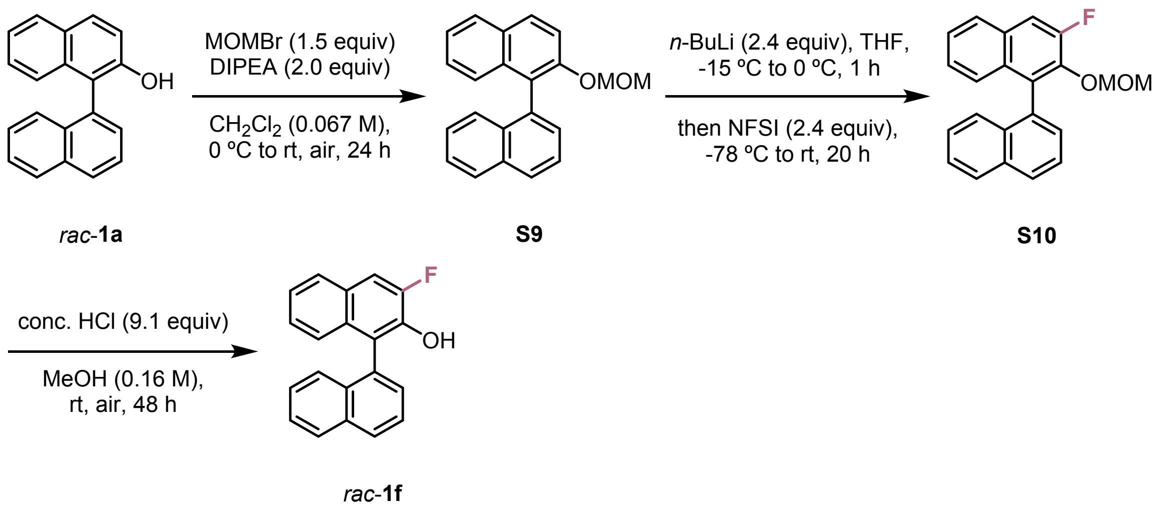

To a solution of *rac*-**1a** (925 mg, 3.42 mmol, 1.0 equiv) and MOMBr (641 mg, 5.13 mmol, 1.5 equiv) in CH_2Cl_2 (51.3 mL) at 0 °C, DIPEA (1.2 mL, 6.84 mmol, 2.0 equiv) was dropwise added, and the mixture was stirred at rt under air for 24 h. The reaction mixture was quenched with water and the layers were separated. The organic phase was washed with 0.5 M HCl, water, 2.0 M NaOH, and brine; dried over anhydrous Na_2SO_4 and concentrated under reduced pressure. The resulting residue was purified by flash column chromatography (AcOEt/hexane 4:96) to afford 674.5 mg (63%) of **S9** as a yellow sticky solid. **Rf**: 0.76 (AcOEt/hexane 25:75, dark green in *p*-anisaldehyde). **^{1}H NMR** (300 MHz, $CDCl_3$): δ: 7.97 (d, *J* = 8.9 Hz, 2H), 7.89 (d, *J* = 8.2 Hz, 1H), 7.63 (d, *J* = 8.3 Hz, 1H), 7.58 (d, *J* = 9.1 Hz, 1H), 7.53 –7.42 (m, 2H), 7.35 (d, *J* = 7.2 Hz, 2H), 7.32 – 7.14 (m, 4H), 5.03 (s, 2H), 3.15 (s, 3H).

To a solution of **S9** (650 mg, 2.07 mmol, 1.0 equiv) in THF (10.5 mL) under nitrogen at -15 °C, *n*-BuLi (2.5 M in hexanes, 2.0 mL, 4.96 mmol, 2.4 equiv) was added, and the resulting mixture was stirred at 0 °C for 1 h. The reaction mixture was cooled down to -78 °C and a solution of NFSI (1.57 g, 4.96 mmol, 2.4 equiv) in THF (10.5 mL) was added. After stirring for 20 h at rt, the reaction was quenched with water and diluted with AcOEt. The layers were separated, and the organic phase was washed with sat. aq. $NaHCO_3$, water, and brine; dried over Na_2SO_4 and concentrated under reduced pressure. The resulting residue was purified by flash column chromatography (AcOEt/hexane 3:97) to afford 441 mg (64%) of **S10** as a yellow sticky solid. **Rf**: 0.80 (AcOEt/hexane 20:80, green in *p*-anisaldehyde). **^{1}H NMR** (300 MHz, $CDCl_3$): δ: 7.98 –7.92 (m, 2H), 7.88 (d, *J* = 8.1 Hz, 1H), 7.61 (d, *J* = 8.3 Hz, 1H), 7.56 (d, *J* = 9.1 Hz, 1H), 7.49 – 7.41 (m, 2H), 7.39 – 7.32 (m, 2H), 7.31 –7.22 (m, 2H), 7.19 (t, *J* = 6.9 Hz, 1H), 5.02 (s, 2H), 3.15 (s, 3H).

To a solution of **S10** (332 mg, 1.32 mmol, 1.0 equiv) in MeOH (8.3 mL, 0.16 M), conc. HCl (1.0 mL, 12 mmol, 9.1 equiv) was slowly added and the mixture was stirred at rt under air for 48 h. The reaction mixture was neutralized with sat. aq. $NaHCO_3$ and extracted with AcOEt (x3). The combined organic phase was dried over Na_2SO_4 and concentrated under reduced pressure. The resulting residue was purified by flash column chromatography (AcOEt/hexane 5:95 to 10:90) to afford 346 mg (91%) of *rac*-**1f** as a white solid. **Rf**: 0.40 (AcOEt/hexane 20:80, green in *p*-anisaldehyde). **^{1}H NMR** (500 MHz, $CDCl_3$) δ: 8.04 (d, *J* = 8.3 Hz, 1H), 7.99 (d, *J* = 8.2 Hz, 1H), 7.81 (d, *J* = 8.2 Hz, 1H), 7.71 – 7.62 (m, 2H), 7.58 – 7.49 (m, 2H), 7.41 – 7.32 (m, 3H), 7.22 (t, *J* = 7.6 Hz 1H), 7.12 (d, *J* = 8.5 Hz, 1H), 5.10 (d, *J* = 1.7 Hz, 1H). **^{13}C NMR** (126 MHz, $CDCl_3$) δ: 151.3 (d, *J* = 246.6 Hz, CF), 141.1 (d, *J* = 15.0, COH), 134.2 (C), 132.7 (C), 131.2 (d, *J* = 2.9 Hz, C), 130.9 (C), 129.5 (CH), 129.4 (CH), 128.7 (CH), 128.5 (d, *J* = 8.4 Hz, C), 127.5 (d, *J* = 5.4 Hz, CH), 127.0 (CH), 126.6 (CH), 126.0 (CH), 125.9 (d, *J* = 2.6 Hz, CH), 125.8 (CH), 125.4 (d, *J* = 1.9 Hz, CH), 124.7 (CH), 122.4 (d, *J* = 2.2 Hz, C), 112.2 (d, *J* = 17.4 Hz, CH). **^{19}F NMR** (282 MHz, $CDCl_3$): δ: -135.4. **HRMS** (APCI+) *m/z* calcd. for $C_{20}H_{14}FO$ [M+H]: 289.1023; found: 289.1015.

3.3. Synthesis of 1-([1,1'-biphenyl]-2-yl)naphthalen-2-ol (*rac*-1m)

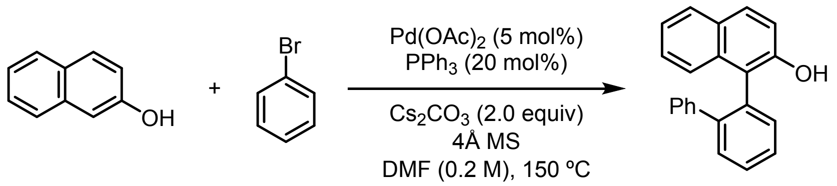

2.5 equiv *rac*-**1m**

Following a literature procedure[6], a mixture of 2-naphthol (1.44 g, 10.0 mmol, 1.0 equiv), $Pd(OAc)_2$ (112 mg, 5.0 mol%), PPh_3 (525 mg, 20 mol%), Cs_2CO_3 (6.52 g, 20.0 mmol, 2.0 equiv), and 4 Å MS (2.0 g) in DMF (50 mL) was degassed by bubbling an argon steam. Bromobenzene (1.5 mL, 25.0 mmol, 2.5 equiv) was added, and the mixture was stirred at 150 ºC for 16 h. After cooling to rt, the reaction mixture was poured into 10% HCl (50 mL) and extracted with Et_2O (x3). The combined organic phase was washed with water, dried over Na_2SO_4, and concentrated under reduced pressure. The resulting residue was purified by flash column chromatography (AcOEt/hexane 4:96) to afford 1.05 g (36%) of *rac*-**1m** as a white powder. **Rf:** 0.45 (AcOEt/hexane 20:80, red in *p*-anisaldehyde). **^{1}H NMR** (500 MHz, $CDCl_3$) δ: 7.75 (d, *J* = 7.9 Hz, 1H), 7.70 (d, *J* = 8.8 Hz, 1H), 7.66 – 7.56 (m, 2H), 7.55 (td, *J* = 7.4, 1.8 Hz, 1H), 7.42 (d, *J* = 7.5 Hz, 1H), 7.38 (d, *J* = 8.2 Hz, 1H), 7.35 – 7.25 (m, 2H), 7.14 – 7.04 (m, 6H), 4.96 (s, 1H). **^{13}C NMR** (126 MHz, $CDCl_3$) δ: 150.2 (COH), 143.6 (C), 140.4 (C), 133.7 (C), 132.8 (CH), 132.3 (C), 131.1 (CH), 129.3 (CH), 128.9 (C), 128.6 (2CH), 128.4 (CH), 128.1 (CH), 127.9 (2CH), 127.6 (CH), 127.2 (CH), 126.6 (CH), 124.9 (CH), 123.3 (CH), 120.6 (C), 117.3 (CH). **HRMS** (APCI+) *m/z* calcd. for $C_{22}H_{16}O$ [M+H]: 297.1274; found: 297.1272.

[6] Satoh, T.; Inoh, J.; Kawamura, Y.; Kawamura, Y.; Miura, M.; Nomura, M. *Bull. Chem. Soc. Jpn.* **1998**, *71*, 2239-2246.

3.4. Synthesis of 2'-methyl-[1,1'-biphenyl]-2-ol (1o)

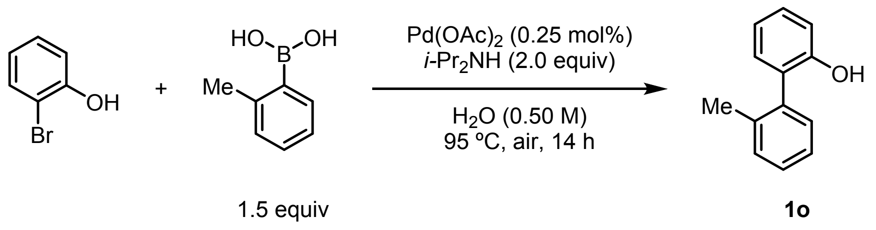

Following a literature procedure[7], a mixture of 2-bromophenol (0.346 g, 2.0 mmol, 1.0 equiv), *o*-tolylboronic acid (0.408 g, 3.0 mmol, 1.5 equiv), $Pd(OAc)_2$ (1.1 mg, 0.005 mmol, 0.25 mol%) and *i*-Pr_2NH (0.56 mL, 4.0 mmol, 2.0 equiv) in H_2O (4.0 mL) was stirred at 95 ºC under air for 14 h. The mixture was quenched with brine and extracted with AcOEt (x3). The combined organic phase was dried over Na_2SO_4 and concentrated under reduced pressure. The resulting residue was purified by flash column chromatography (AcOEt/hexane 1:99) to afford 223.3 mg (61%) of **1o** as a colorless oil. **Rf:** 0.26 (AcOEt/hexane 5:95, orange in *p*-anisaldehyde). **^{1}H NMR** (300 MHz, $CDCl_3$) δ: 7.38 – 7.21 (m, 5H), 7.12 (d, *J* = 7.3, 1.8 Hz, 1H), 7.00 (d, *J* = 7.2 Hz, 2H), 4.75 (s, 1H), 2.18 (s, 3H). **^{13}C NMR** (75 MHz, $CDCl_3$) δ: 152.7 (COH), 137.6 (C), 135.9 (C), 130.9 (CH), 130.6 (CH), 130.3 (CH), 129.3 (CH), 182.7 (CH), 127.9 (C), 126.6 (CH), 120.6 (CH), 115.4 (CH), 19.9 (CH_3). **HRMS** (APCI+) *m/z* calcd. for $C_{13}H_{13}O$ [M+H]: 185.0961; found: 185.0955.

[7] Duan, S.; Xu, Y.; Zhang, X.; Fan, X. *Chem. Commun.* **2016**, *52*, 10529-10532.

3.5. Synthesis of 1-(2-methoxyphenyl)naphthalen-2-ol (*rac*-1p)

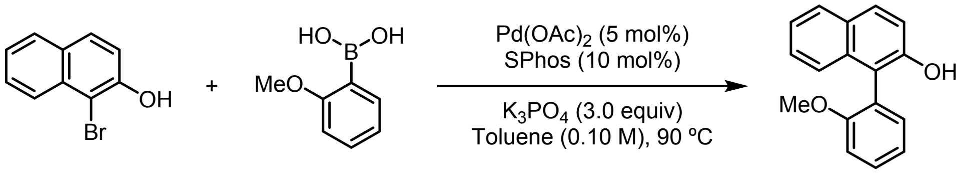

S1 1.2 equiv *rac*-**1p**

A mixture of **S1** (0.446 g, 2.0 mmol, 1.0 equiv), $Pd(OAc)_2$ (22.5 mg, 0.10 mmol, 5 mol%), SPhos (82.1 mg, 0.20 mmol, 10 mol%) and K_3PO_4 (1.27 g, 6.0 mmol, 3.0 equiv) in toluene (20 mL) was degassed by bubbling argon. (2-methoxyphenyl)boronic acid (0.365 g, 2.4 mmol 1.2 equiv) was added portionwise, and the mixture was stirred at 90 ºC for 20 h. After cooling to rt, the reaction mixture was partitioned between water and CH_2Cl_2. The layers were separated, and the aqueous phase was extracted with CH_2Cl_2 (x2). The combined organic phase was dried with Na_2SO_4 and concentrated under reduced pressure. The resulting residue was purified by flash column chromatography (AcOEt/hexane 2.5:97.5 to 10:90) to afford 132.5 mg (27%) of *rac*-**1p** as a sticky red solid. **Rf:** 0.30 (AcOEt/hexane 20:80, dark red in *p*-anisaldehyde). **^{1}H NMR** (500 MHz, $CDCl_3$) δ: 7.84 – 7.79 (m, 2H), 7.51 (td, *J* = 7.9, 1.9 Hz, 1H), 7.34 (m, 4H), 7.28 (d, *J* = 8.9 Hz, 1H), 7.19 – 7.13 (m, 2H), 5.28 (s, 1H), 3.76 (s, 3H). **^{13}C NMR** (126 MHz, $CDCl_3$) δ: 158.0 (COH), 150.8 (C), 133.6 (C), 133.5 (CH), 130.4 (CH), 129.6 (CH), 129.3 (C), 128.2 (CH), 126.4 (CH), 125.0 (CH), 123.3 (CH), 122.7 (C), 121.7 (CH), 118.0 (C), 117.9 (CH), 112.1 (CH), 56.0 (OCH_3). **HRMS** (APCI+) *m/z* calcd. for $C_{17}H_{14}O_2$ [M+H]: 251.1067; found: 251.1067.

4. Kinetic resolution of biaryl alcohols through Pd(II)-catalyzed C-H alkenylation

4.1. Optimization of the reaction conditions

4.1.1. Temperature and time optimization

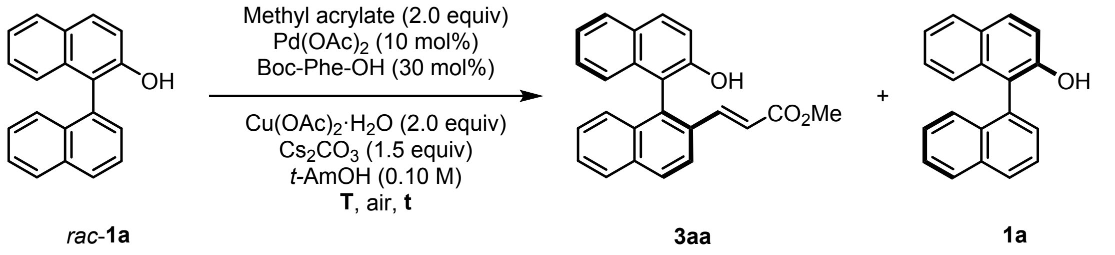

Entry	T (ºC)	t (h)	**1a** yield (%)	**1a** er	**3aa** yield (%)	**3aa** er
1	80	2.5	40	92:8	40	94:6
2	60	2.5	50	82:18	43	94:6
**3**	**45**	**24**	**40**	**93:7**	**42**	**95:5**
4	35	25.5	43	82:18	32	97.5:2.5
5	21	24	60	63:37	20	98:2

Table S1. Temperature and time optimization.

Reaction conditions: **1a** (0.1 mmol), **2a** (0.2 mmol), $Pd(OAc)_2$ (10 mol%), Boc-Phe-OH (30 mol%), $Cu(OAc)_2 \cdot H_2O$ (2.0 equiv), Cs_2CO_3 (1.5 equiv), *t*-AmOH (1.0 mL), air. Isolated yields. Enantiomeric ratios (er) were determined by chiral HPLC analysis of the isolated pure product.

4.1.2. Chiral ligand screening

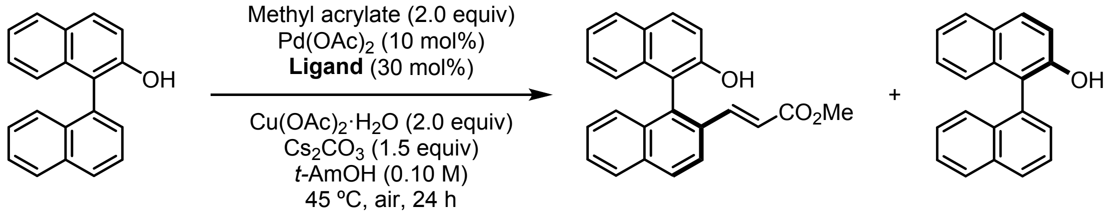

rac-**1a** **3aa** **1a**

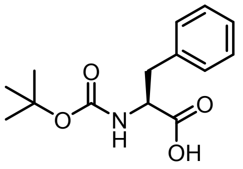

1a: 40%, 93:7 er
3aa: 42%, 95:5 er

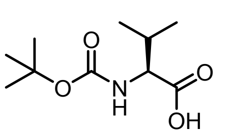

1a: 40%, 92:8 er
3aa: 43%, 95:5 er

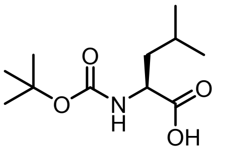

1a: 45%, 87:13 er
3aa: 40%, 95.5:4.5 er

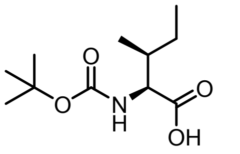

1a: 41%, 96.5:3.5 er
3aa: 49%, 95.5:4.5 er

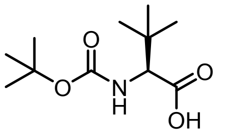

1a: 47%, 79.7:20.3 er
3aa: 34%, 95:5 er

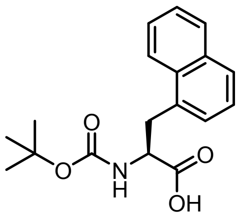

1a: 46%, 91:9 er
3aa: 47%, 95:5 er

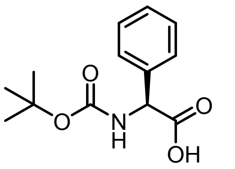

1a: 69%, 60:40 er
3aa: 21%, 91.7:8.3 er

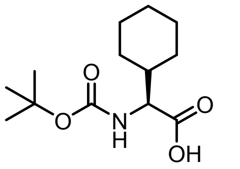

1a: 39%, 97:3 er
3aa: 47%, 94:6 er

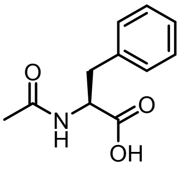

1a: 73%, 54:46 er
3aa: 10%, 90:10 er

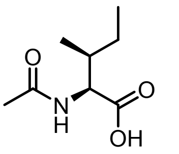

1a: 83%, 51:49 er
3aa: 6%, 79:21 er

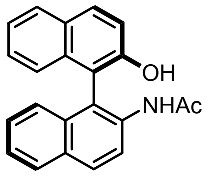

1a: 70%, 52:48 er
3aa: 20%, 56:44 er

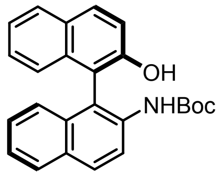

1a: 64%, 51:49 er
3aa: 16%, 57:43 er

Table S2. MPAA and NOBINAc-type chiral ligand screening.

Reaction conditions: **1a** (0.1 mmol), **2a** (0.2 mmol), $Pd(OAc)_2$ (10 mol%), ligand (30 mol%), $Cu(OAc)_2 \cdot H_2O$ (2.0 equiv), Cs_2CO_3 (1.5 equiv), *t*-AmOH (1.0 mL), air, 45 °C, 24 h. Isolated yields. Enantiomeric ratios (er) were determined by chiral HPLC analysis of the isolated pure product.

4.1.3. Solvent screening

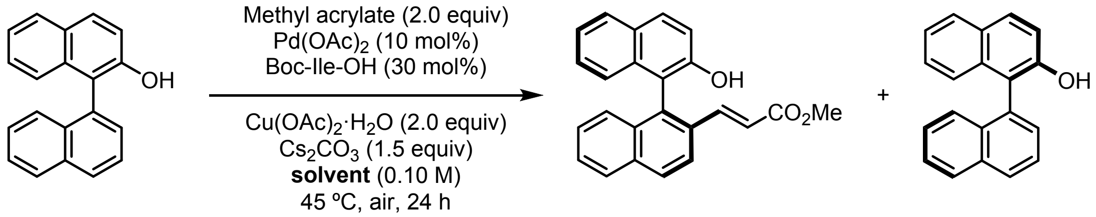

rac-**1a** **3aa** **1a**

Entry	Solvent	**1a** yield (%)	**1a** er	**3aa** yield (%)	**3aa** er
**1**	***t*-AmOH**	**41**	**96.5:3.5**	**49**	**95.5:4.5**
2	MeOH	70	54.5:45.5	8	96:4
3	HFIP	62	50:50	18	50:50
4	MeCN	43	90:10	41	94:6

Table S3. Solvent screening.

Reaction conditions: **1a** (0.1 mmol), **2a** (0.2 mmol), $Pd(OAc)_2$ (10 mol%), Boc-Ile-OH (30 mol%), $Cu(OAc)_2 \cdot H_2O$ (2.0 equiv), Cs_2CO_3 (1.5 equiv), solvent (1.0 mL), air, 45 °C, 24 h. Isolated yields. Enantiomeric ratios (er) were determined by chiral HPLC analysis of the isolated pure product.

4.1.4. Oxidant screening

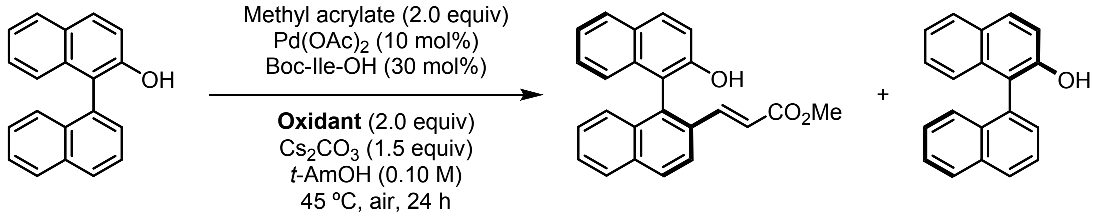

rac-**1a** **3aa** **1a**

Entry	Oxidant	**1a** yield (%)	**1a** er	**3aa** yield (%)	**3aa** er
**1**	**$Cu(OAc)_2·H_2O$**	**41**	**96.5:3.5**	**49**	**95.5:4.5**
2	AgOAc	0	-	0	-
3	Ag_3PO_4	30	73:27	38	93:7
4	Ag_2CO_3	0	-	0	-

Table S4. Oxidant screening.

Reaction conditions: **1a** (0.1 mmol), **2a** (0.2 mmol), $Pd(OAc)_2$ (10 mol%), Boc-Ile-OH (30 mol%), Oxidant (2.0 equiv), Cs_2CO_3 (1.5 equiv), *t*-AmOH (1.0 mL), air, 45 °C, 24 h. Isolated yields. Enantiomeric ratios (er) were determined by chiral HPLC analysis of the isolated pure product.

4.1.5. Loading studies

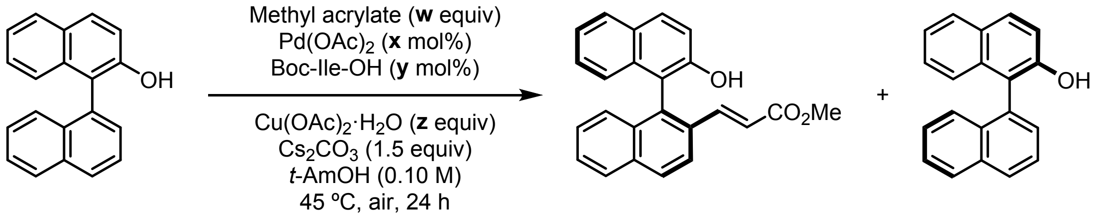

rac-**1a** **3aa** **1a**

Entry	w	z	x	y	**1a** yield (%)	SM er	**3aa** yield (%)	**3aa** er
1	2	2	10	30	41	96.5:3.5	49	95.5:3.5
2	2	1	10	30	43	96.3:3.7	51	95.6:4.3
3	2	1	10	20	45	96.6:3.4	54	95:5
4	2	0.2	10	20	34	98.4:1.6	47	81:19
5	2	1	10	10	40	97.5:2.5	51	92:8
6	2	1	5	10	36	95:5	51	96:4
7	1	1	5	10	42	97.2:2.8	50	96:4

Table S5. Loading studies.

Reaction conditions: **1a** (0.1 mmol), **2a**, $Pd(OAc)_2$, Boc-Ile-OH, $Cu(OAc)_2{\cdot}H_2O$, Cs_2CO_3 (1.5 equiv), *t*-AmOH (1.0 mL), air, 45 °C, 24 h. Isolated yields. Enantiomeric ratios (er) were determined by chiral HPLC analysis of the isolated pure product. **Entry 7** Optimized conditions.

4.2. Reaction scope

General procedure (GP) for the kinetic resolution of biaryl alcohols through Pd(II)-catalyzed C-H alkenylation

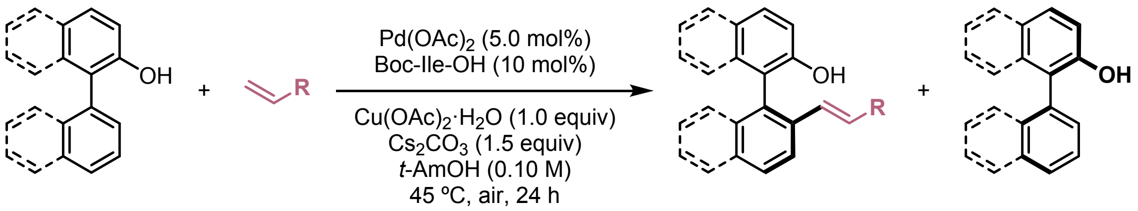

rac-**1** **2**, 1.0 equiv **3** **1**

The corresponding biaryl alcohol *rac*-**1** (0.10 mmol, 1.0 equiv), $Pd(OAc)_2$ (1.1 mg, 5.0 mol%), Boc-Ile-OH (2.3 mg, 10 mol%), $Cu(OAc)_2{\cdot}H_2O$ (20 mg, 1.0 equiv) and Cs_2CO_3 (49 mg, 1.5 equiv) were weighed and added into a Schlenk flask under air. Then, *t*-AmOH (1.0 mL, 0.10 M) and the corresponding alkene (0.10 mmol, 1.0 equiv) were added. The flask was sealed with a rubber septum and the mixture was stirred under air at 45 ºC for 24 h. After cooling to rt, the reaction mixture was diluted with AcOEt and filtered through a Celite pad, washing the flask and the pad with more AcOEt (x3). The filtrate was concentrated under reduced pressure (50 ºC) and the resulting residue was purified by flash column chromatography (AcOEt/hexane) to afford **1** and **3**.

Methyl (*S*,*E*)-3-(2'-hydroxy-[1,1'-binaphthalen]-2-yl)acrylate (3aa)

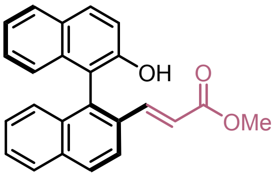

3aa

3aa was obtained by following the GP after column chromatography (AcOEt/hexane 15:85 to 25:75) as a white solid (50%, 96:4 er). **Rf:** 0.35 (AcOEt/hexane 25:75, brown in *p*-anisaldehyde). **^{1}H NMR** (500 MHz, $CDCl_3$) δ: 8.01 (d, J = 8.8 Hz, 1H), 7.96 – 7.88 (m, 4H), 7.52 (ddd, J = 8.1, 6.6, 1.4 Hz, 1H), 7.41 (d, J = 16.1 Hz, 1H), 7.35 – 7.32 (m, 2H), 7.31 – 7.28 (m, 1H), 7.27 – 7.25 (m, 1H), 7.22 (ddd, J = 8.3, 6.8, 1.5 Hz, 1H), 6.90 (d, J = 8.4 Hz, 1H), 6.48 (d, J = 16.0 Hz, 1H), 4.99 (s, 1H), 3.59 (s, 3H). **^{13}C NMR** (126 MHz, $CDCl_3$) δ: 167.2 (CO), 151.4 (COH), 142.3 (CH), 134.7 (C), 134.0 (C), 133.4 (C), 133.3 (C), 133.0 (C), 130.8 (CH), 129.7 (CH), 129.1 (C), 128.4 (CH), 128.3 (CH), 127.7 (CH), 127.6 (CH), 127.1 (CH), 126.9 (CH), 124.8 (CH), 123.7 (CH), 123.4 (CH), 120.1 (CH), 117.8 (CH), 115.7 (C), 51.7 (OCH_3). **HRMS** (APCI+) *m/z* calcd. for $C_{24}H_{19}O_3$ [M+H]: 355.1329; found: 355.1342.

Absolute configuration of compound **3aa** was determined by X-ray crystallography. The structure was deposited in the Cambridge Structural Database: 2286789.

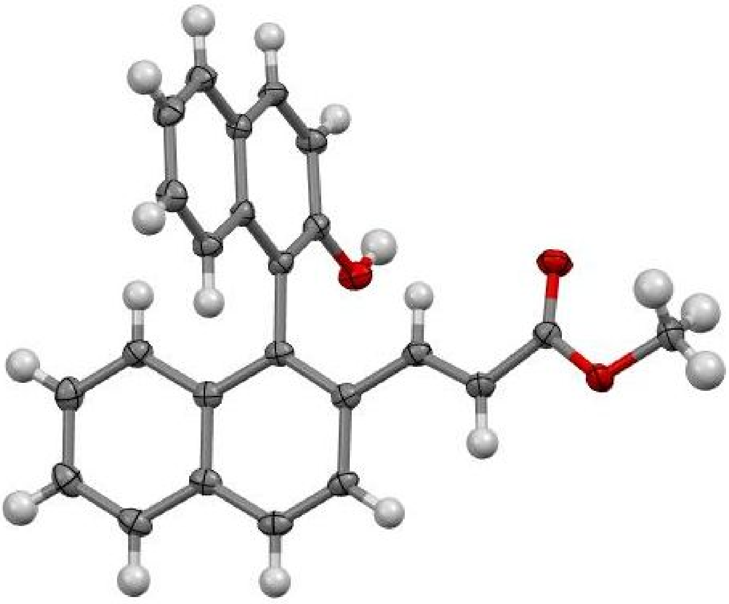

Enantioselectivity of the product was determined by chiral HPLC analysis (Chiralpak IA3, IPA/hexane 15:85, 0.5 mL/min, λ=254 nm).

Racemic sample

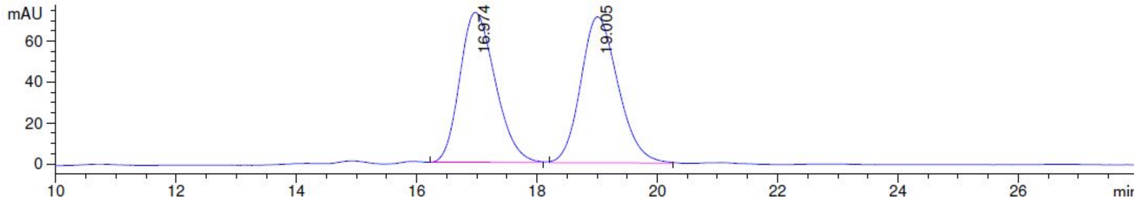

Peak #	RetTime [min]	Type	Width [min]	Area [mAU*s]	Height [mAU]	Area %
1	16.975	PB	0.6474	6837.99463	164.36688	49.6864
2	19.007	BB	0.6687	6924.32227	158.79199	50.3136

Chiral sample

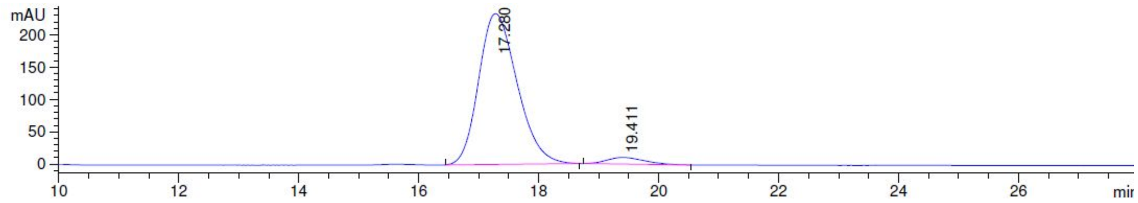

Peak #	RetTime [min]	Type	Width [min]	Area [mAU*s]	Height [mAU]	Area %
1	17.280	BP	0.6822	1.04065e4	233.36993	95.9219
2	19.411	PB	0.5178	442.43039	10.24627	4.0781

Enantioselectivity of the remaining starting material (42%, 97:3 er) was determined by chiral HPLC analysis (Chiralpak IA3, IPA/hexane 20:80, 1.0 mL/min, λ=220 nm).

Racemic sample

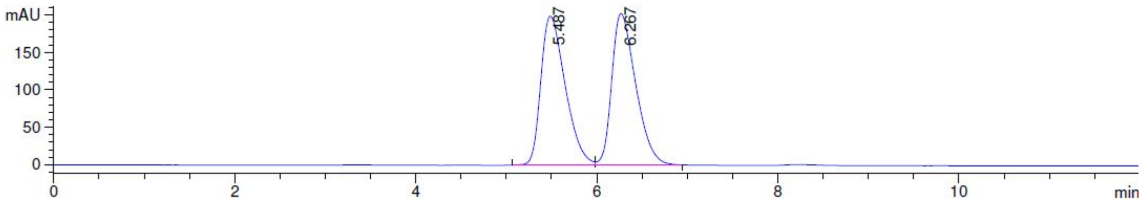

Peak #	RetTime [min]	Type	Width [min]	Area [mAU*s]	Height [mAU]	Area %
1	5.487	BV	0.2903	3729.95630	199.31519	49.9451
2	6.267	VB	0.2811	3738.15479	202.79050	50.0549

Chiral sample

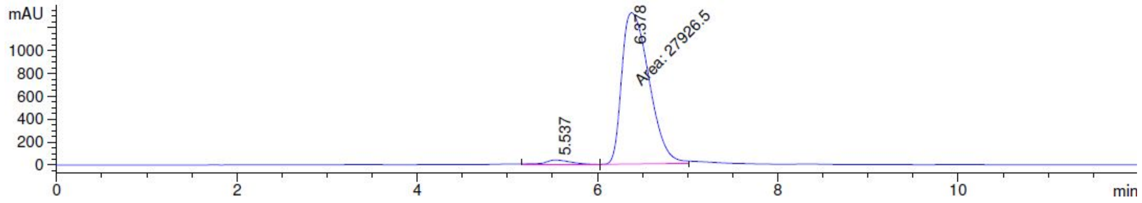

Peak #	RetTime [min]	Type	Width [min]	Area [mAU*s]	Height [mAU]	Area %
1	5.537	VV	0.2998	810.58215	40.44770	2.8207
2	6.378	MM	0.3516	2.79265e4	1323.91187	97.1793

tert-Butyl (_S_,_E_)-3-(2'-hydroxy-[1,1'-binaphthalen]-2-yl)acrylate (3ab)

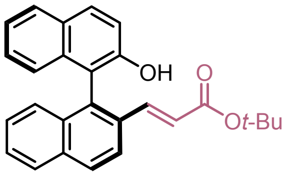

3ab

3ab was obtained by following the GP after column chromatography (AcOEt/hexane 10:90) as white solid (48%, 95.5:4.5 er). **Rf:** 0.58 (AcOEt/hexane 25:75, brown in *p*-anisaldehyde). **^{1}H NMR** (500 MHz, $CDCl_3$) δ: 8.02 (d, *J* = 8.8 Hz, 1H), 7.97 – 7.91 (m, 3H), 7.87 (d, *J* = 8.2 Hz, 1H), 7.52 (td, *J* = 7.4, 1.3 Hz, 1H), 7.36 – 7.26 (m, 5H), 7.21 (td, *J* = 7.6, 1.4 Hz, 1H), 6.90 (d, *J* = 8.5 Hz, 1H), 6.45 (d, *J* = 15.9 Hz, 1H), 4.69 (s, 1H), 1.37 (s, 9H). **^{13}C NMR** (75 MHz, $CDCl_3$) δ: 165.9 (CO), 151.3 (COH), 141.0 (CH), 134.6 (C), 133.9 (C), 133.3 (C), 133.3 (C), 132.8 (C), 130.8 (CH), 129.8 (CH), 129.2 (C), 128.4 (CH), 128.3 (CH), 127.6 (CH), 127.6 (CH), 127.1 (CH), 126.9 (CH), 124.9 (CH), 123.7 (CH), 123.4 (CH), 122.4 (CH), 117.6 (CH), 115.9 (C), 86.5 (C), 28.2 ($3CH_3$). **HRMS** (APCI+) *m/z* calcd. for $C_{27}H_{24}O_3$ [M+H]: 397.1725; found: 397.1729.

Enantioselectivity of the product was determined by chiral HPLC analysis (Chiralpak IF3, IPA/hexane 15:85, flow rate = 0.5 mL/min, λ=300 nm).

Racemic sample

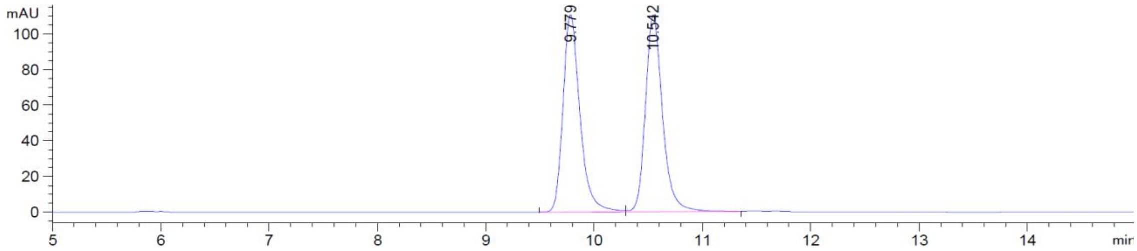

Peak #	RetTime [min]	Type	Width [min]	Area [mAU*s]	Height [mAU]	Area %
1	9.779	BV	0.1649	1204.34753	110.74083	49.8272
2	10.542	VB	0.1680	1212.70129	110.52292	50.1728

Chiral sample

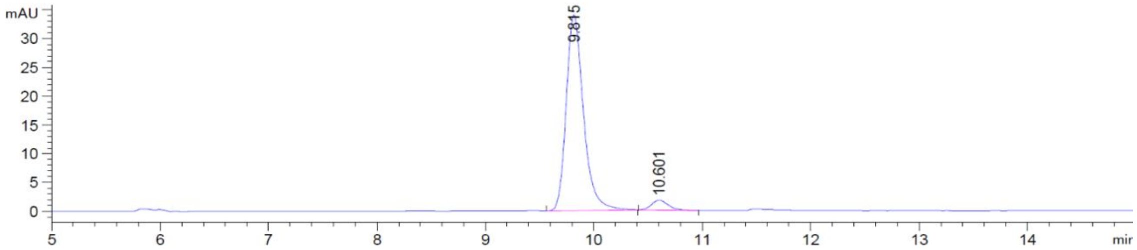

Peak #	RetTime [min]	Type	Width [min]	Area [mAU*s]	Height [mAU]	Area %
1	9.815	BB	0.1690	376.51291	34.04079	95.3583
2	10.601	BB	0.1616	18.32714	1.73053	4.6417

Enantioselectivity of the remaining starting material (48%, 94.5:5.5 er) was determined by chiral HPLC analysis (Chiralpak IA3, IPA/hexane 20:80, 1.0 mL/min, λ=220 nm).

Racemic sample

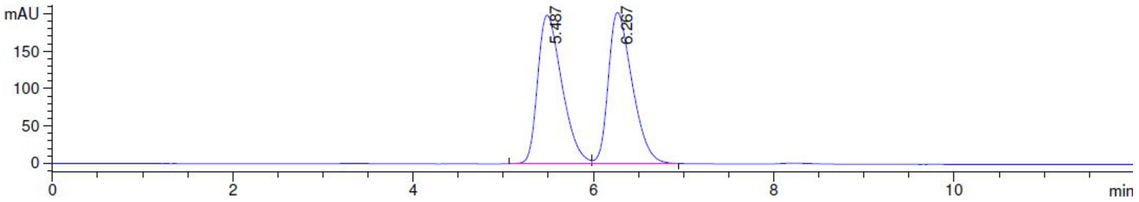

Peak #	RetTime [min]	Type	Width [min]	Area [mAU*s]	Height [mAU]	Area %
1	5.487	BV	0.2903	3729.95630	199.31519	49.9451
2	6.267	VB	0.2811	3738.15479	202.79050	50.0549

Chiral sample

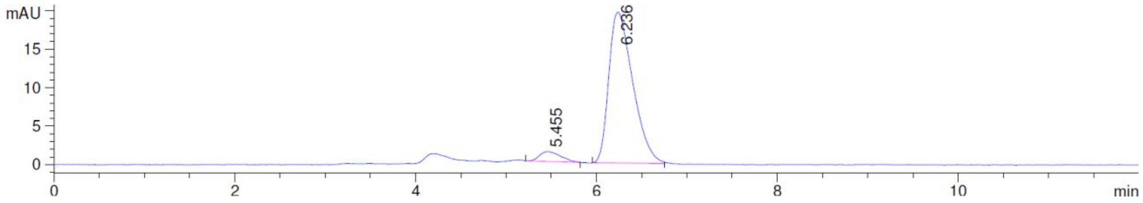

Peak #	RetTime [min]	Type	Width [min]	Area [mAU*s]	Height [mAU]	Area %
1	5.455	PB	0.2150	20.71888	1.27881	5.4213
2	6.236	BB	0.2751	361.45444	19.60280	94.5787

Diethyl (*S*,*E*)-(2-(2'-hydroxy-[1,1'-binaphthalen]-2-yl)vinyl)phosphonate (3ac)

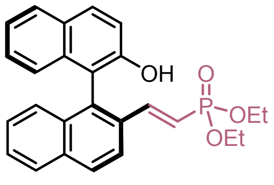

3ac

3ac was obtained by following the GP after column chromatography (AcOEt/hexane 12:88 to 100:0) as a white solid (51%, 94.5:5.5 er). **Rf:** 0.40 (MeOH/CH_2Cl_2 5:95, red in *p*-anisaldehyde). **^{1}H NMR** (300 MHz, $CDCl_3$) δ: 8.03 (d, *J* = 8.7 Hz, 1H), 7.96 – 7.84 (m, 4H), 7.57 – 7.49 (m, 1H), 7.35 – 7.28 (m, 4H), 7.20 (ddd, *J* = 8.3, 6.8, 1.4 Hz, 1H), 7.15 – 7.01 (m, 1H), 6.88 (d, *J* = 8.4 Hz, 1H), 6.29 (t, *J* = 18.1 Hz, 1H), 5.33 (s, 1H), 3.80 (p, *J* = 7.1 Hz, 3H), 3.73 – 3.59 (m, 1H), 1.09 (t, *J* = 7.1 Hz, 3H), 1.00 (t, *J* = 7.1 Hz, 3H). **^{13}C NMR** (75 MHz, $CDCl_3$) δ: 151.7 (COH), 145.7 (d, *J* = 6.8 Hz, CH), 134.6 (C), 134.0 (C), 133.6 (C), 133.3 (C), 133.2 (C), 132.9 (C), 130.7 (CH), 129.7 (CH), 129.1 (C), 128.4 (CH), 128.3 (CH), 127.7 (CH), 127.6 (CH), 127.1 (CH), 126.9 (CH), 124.8 (CH), 123.7 (CH), 123.2 (CH), 117.8 (CH), 117.7 (C), 115.8 (C), 115.2 (CH), 62.1 (d, *J* = 5.8 Hz, 2CH), 16.2 (t, *J* = 7.0 Hz, $2CH_3$). **^{31}P NMR** (202 MHz, $CDCl_3$) δ: 19.4. **HRMS** (APCI+) *m/z* calcd. for $C_{26}H_{26}O_4P$ [M+H]: 433.1563; found: 433.1557.

Enantioselectivity of the product was determined by chiral HPLC analysis (Chiralpak IE3, IPA/hexane 20:80, 0.5 mL/min, λ=254 nm).

Racemic sample

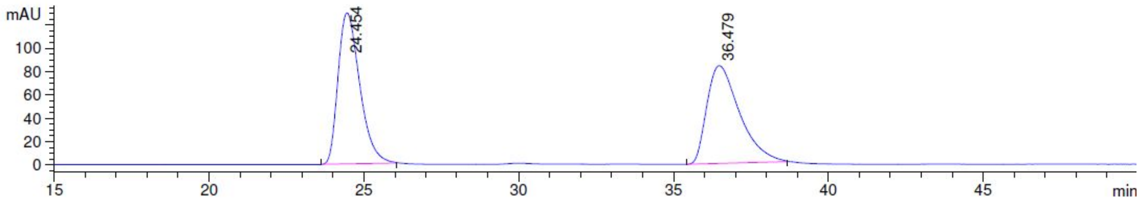

Peak #	RetTime [min]	Type	Width [min]	Area [mAU*s]	Height [mAU]	Area %
1	24.455	BB	0.7463	7208.46582	147.46434	50.3620
2	36.479	PB	1.0508	7104.82715	95.63235	49.6380

Chiral sample

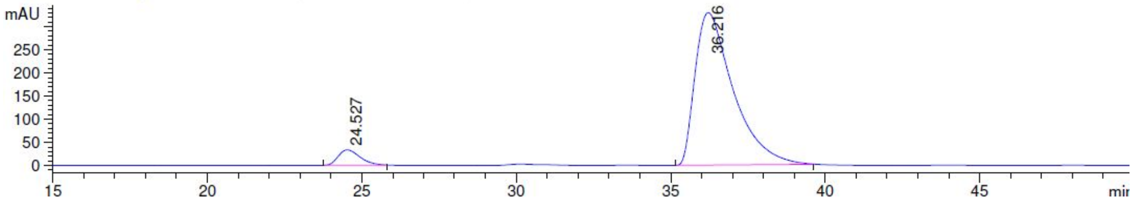

Peak #	RetTime [min]	Type	Width [min]	Area [mAU*s]	Height [mAU]	Area %
1	24.527	BB	0.7190	1622.01550	33.14369	5.4010
2	36.216	PB	1.2892	2.84099e4	329.73676	94.5990

Enantioselectivity of the remaining starting material (40%, 95.5:4.5) was determined by chiral HPLC analysis (Chiralpak IA3, IPA/hexane 20:80, 1.0 mL/min, λ=220 nm).

Racemic sample

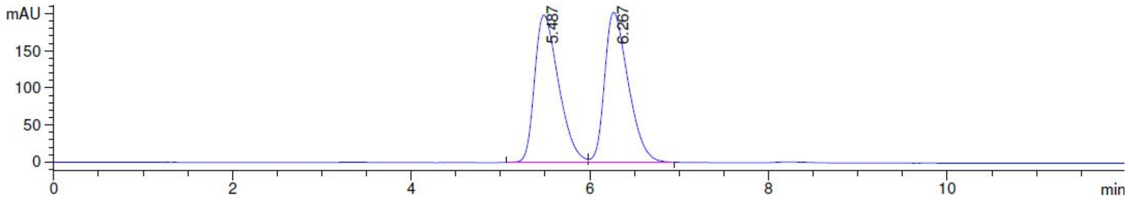

Peak #	RetTime [min]	Type	Width [min]	Area [mAU*s]	Height [mAU]	Area %
1	5.487	BV	0.2903	3729.95630	199.31519	49.9451
2	6.267	VB	0.2811	3738.15479	202.79050	50.0549

Chiral sample

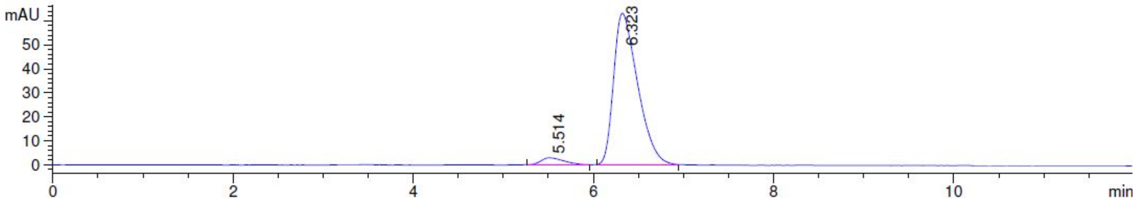

Peak #	RetTime [min]	Type	Width [min]	Area [mAU*s]	Height [mAU]	Area %
1	5.514	BB	0.2676	52.92949	2.91885	4.3019
2	6.323	BB	0.2833	1177.44629	63.20213	95.6981

(*S*,*E*)-3-(2'-hydroxy-[1,1'-binaphthalen]-2-yl)-*N*,*N*-dimethylacrylamide (3ad)

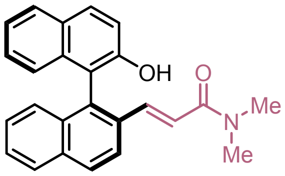

3ad

3ad was obtained by following the GP after column chromatography (AcOEt/hexane 10:90 to 100:0) as white solid (54%, 94:6 er). **Rf:** 0.54 (EtOAc, pale yellow in *p*-anisaldehyde). **^{1}H NMR** (300 MHz, $CDCl_3$) δ: 8.00 (d, *J* = 8.7 Hz, 1H), 7.95 – 7.85 (m, 4H), 7.50 (ddd, *J* = 8.1, 5.6, 2.4 Hz, 1H), 7.40 – 7.26 (m, 5H), 7.20 (ddd, *J* = 8.1, 6.8, 1.3 Hz, 1H), 6.91 (d, *J* = 8.4 Hz, 1H), 6.74 (d, *J* = 15.6 Hz, 1H), 5.69 (s, 1H), 2.90 (s, 3H), 2.84 (s, 3H). **^{13}C NMR** (75 MHz, $CDCl_3$) δ: 166.9 (CO), 151.7 (COH), 139.9 (CH), 134.3 (C), 134.1 (C), 133.8 (C), 133.5 (C), 132.9 (C), 130.5 (CH), 129.3 (CH), 128.3 (CH), 128.2 (CH), 127.3 (CH), 127.2 (CH), 127.0 (CH), 126.9 (CH), 124.8 (CH), 124.3 (CH), 123.5 (CH), 120.2 (CH), 118.1 (CH), 117.9 (C), 116.2 (C), 37.4 (CH_3), 35.8 (CH_3). **HRMS** (APCI+) *m/z* calcd. for $C_{25}H_{22}NO_2$ [M+H]: 368.1645; found: 368.1645.

Enantioselectivity of the product was determined by chiral HPLC analysis (Chiralpak IA3, IPA/hexane 15:85, flow rate = 0.5 mL/min, λ = 254 nm).

Racemic sample

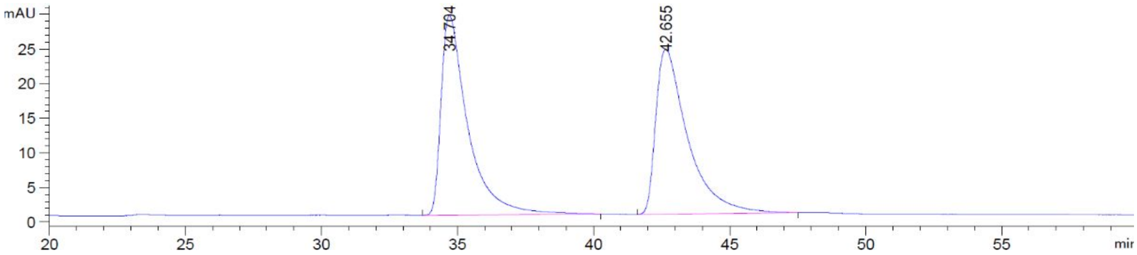

Peak #	RetTime [min]	Type	Width [min]	Area [mAU*s]	Height [mAU]	Area %
1	34.704	BB	0.9771	1978.82080	28.88938	50.5114
2	42.655	BB	1.1555	1938.74805	23.82257	49.4886

Chiral sample

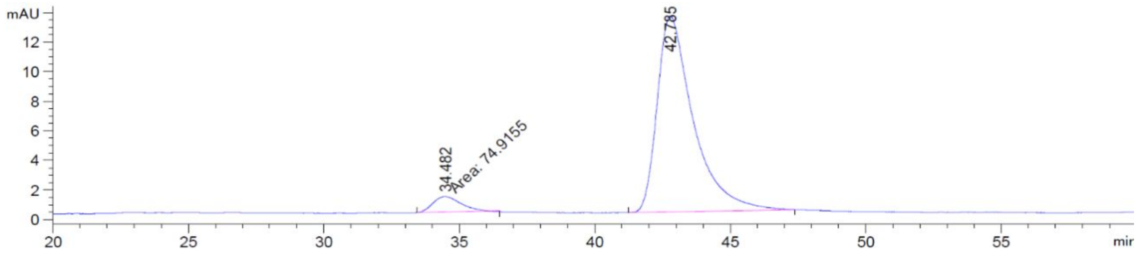

Peak #	RetTime [min]	Type	Width [min]	Area [mAU*s]	Height [mAU]	Area %
1	34.482	MM	1.1865	74.91554	1.05237	5.7583
2	42.785	BB	1.2472	1226.08313	13.25453	94.2417

Enantioselectivity of the remaining starting material (45%, 96:4 er) was determined by chiral HPLC analysis (Chiralpak IA3, IPA/hexane 15:85, 0.5 mL/min, λ=220 nm).

Racemic Sample

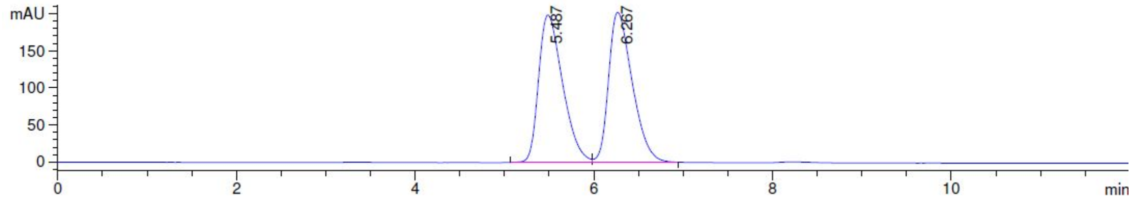

Peak #	RetTime [min]	Type	Width [min]	Area [mAU*s]	Height [mAU]	Area %
1	5.487	BV	0.2903	3729.95630	199.31519	49.9451
2	6.267	VB	0.2811	3738.15479	202.79050	50.0549

Chiral Sample

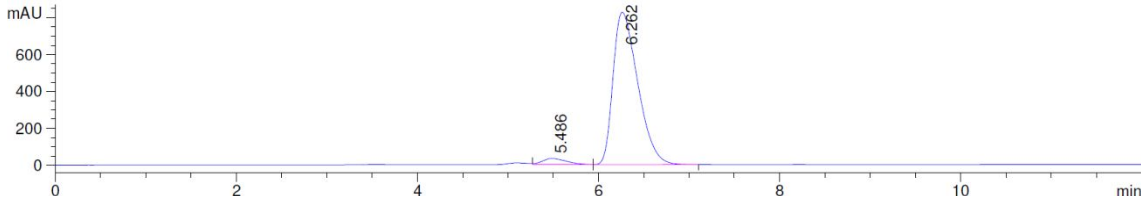

Peak #	RetTime [min]	Type	Width [min]	Area [mAU*s]	Height [mAU]	Area %
1	5.486	VV	0.2849	640.30023	33.81559	3.9188
2	6.262	VB	0.2934	1.56987e4	827.20300	96.0812

(*S*,*E*)-2'-styryl-[1,1'-binaphthalen]-2-ol (3ae)

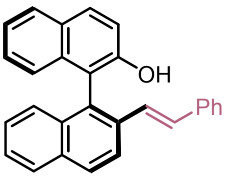

3ae

3ae was obtained by following the GP after column chromatography (AcOEt/hexane 5:95 to 7:93) as a white solid (44%, 95:5 er). **Rf:** 0.40 (AcOEt/hexane 15:85, brown in *p*-anisaldehyde). **^{1}H NMR** (300 MHz, $CDCl_3$) δ: 8.06 (q, *J* = 8.7 Hz, 2H), 7.98 (d, *J* = 8.9 Hz, 1H), 7.92 (t, *J* = 7.4 Hz, 2H), 7.48 (ddd, *J* = 8.1, 6.6, 1.5 Hz, 1H), 7.38 (d, *J* = 8.9 Hz, 1H), 7.35 – 7.14 (m, 10H), 7.02 (d, *J* = 8.5 Hz, 1H), 6.81 (d, *J* = 16.3 Hz, 1H), 4.82 (s, 1H). **^{13}C NMR** (75 MHz, $CDCl_3$) δ: 151.4 (COH), 137.3 (C), 135.8 (C), 134.0 (C), 133.6 (C), 131.2 (CH), 130.4 (CH), 129.6 (CH), 129.3 (C), 129.2 (C), 128.7 (2CH), 128.3 (CH), 128.3 (CH), 127.9 (CH), 127.4 (CH), 127.0 (CH), 126.8 (2CH), 126.6 (CH), 126.4 (CH), 126.3 (CH), 125.1 (CH), 123.7 (CH), 123.3 (CH), 117.6 (CH), 116.7 (C). **HRMS** (APCI+) *m/z* calcd. for $C_{28}H_{21}O$ [M+H]: 373.1587; found: 373.1586.

Enantioselectivity of the product was determined by chiral HPLC analysis (Chiralpak IB, IPA/hexane 5:95, 0.5 mL/min, λ=254 nm).

Racemic sample

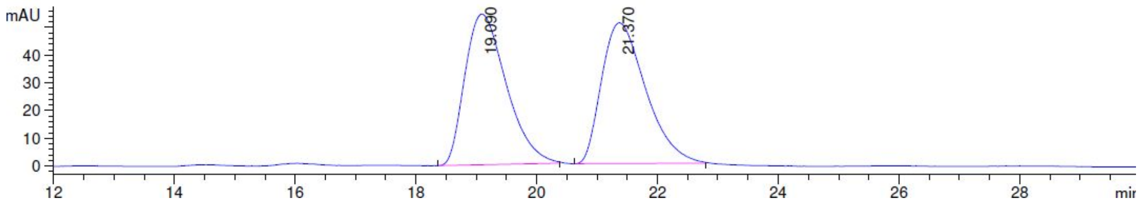

Peak #	RetTime [min]	Type	Width [min]	Area [mAU*s]	Height [mAU]	Area %
1	19.090	BB	0.7215	2589.34741	54.38093	50.0598
2	21.370	PB	0.7473	2583.15698	50.79741	49.9402

Chiral sample

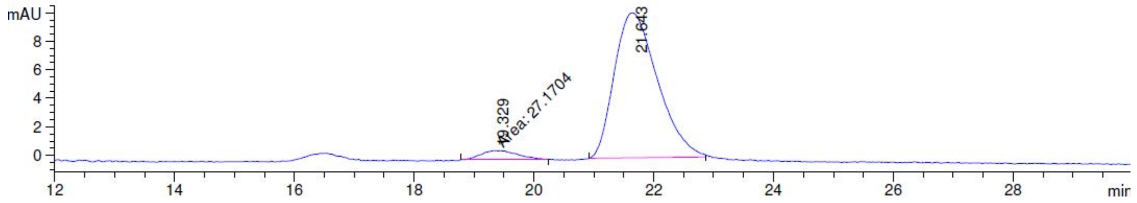

Peak #	RetTime [min]	Type	Width [min]	Area [mAU*s]	Height [mAU]	Area %
1	19.329	MM	0.7061	27.17039	6.41311e-1	5.0608
2	21.643	PB	0.6422	509.70972	10.17928	94.9392

Enantioselectivity of the remaining starting material (34%, 97:3 er) was determined by chiral HPLC analysis (Chiralpak IA3, IPA/hexane 20:80, 1.0 mL/min, λ=254 nm).

Racemic sample

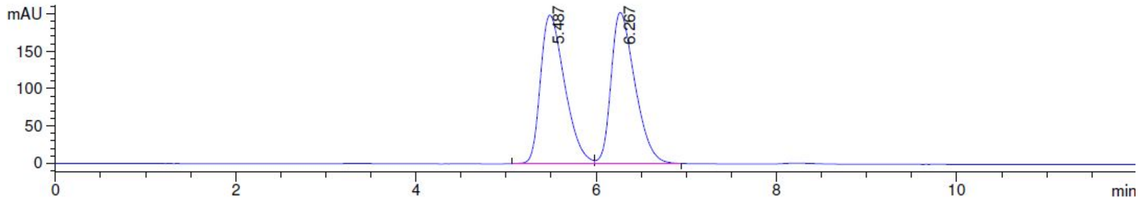

Peak #	RetTime [min]	Type	Width [min]	Area [mAU*s]	Height [mAU]	Area %
1	5.487	BV	0.2903	3729.95630	199.31519	49.9451
2	6.267	VB	0.2811	3738.15479	202.79050	50.0549

Chiral sample

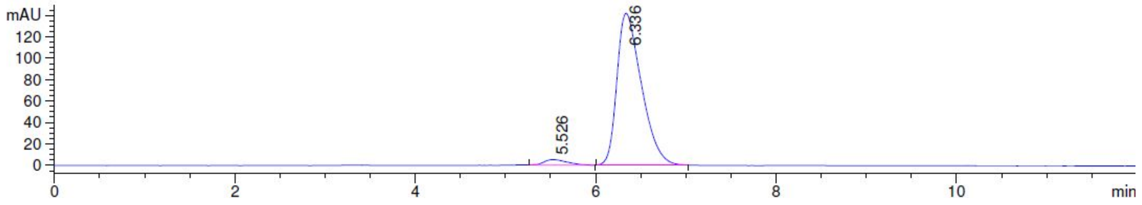

Peak #	RetTime [min]	Type	Width [min]	Area [mAU*s]	Height [mAU]	Area %
1	5.526	BB	0.2612	90.05308	5.07058	3.2460
2	6.336	BB	0.2886	2684.20703	141.96140	96.7540

(*S*,*E*)-2'-(4-nitrostyryl)-[1,1'-binaphthalen]-2-ol (3af)

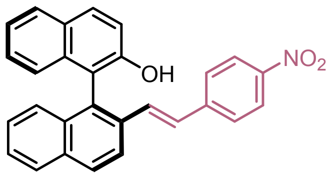

3af

3af was obtained by following the GP after column chromatography (AcOEt/hexane 8:92 to 25:75) as a bright yellow solid (37%, 96.5:3.5 er). **Rf:** 0.40 (AcOEt/hexane 25:75, cream color in *p*-anisaldehyde). **^{1}H NMR** (300 MHz, $CDCl_3$) δ: 8.14 – 8.00 (m, 5H), 7.96 (t, *J* = 8.4 Hz, 2H), 7.54 (t, *J* = 7.3 Hz, 1H), 7.43 – 7.22 (m, 8H), 7.06 – 6.93 (m, 2H), 4.82 (s, 1H). **^{13}C NMR** (75 MHz, $CDCl_3$) δ: 151.5 (COH), 147.0 (C), 143.7 (C), 134.7 (C), 134.1 (C), 133.9 (C), 133.5 (C), 130.9 (CH), 130.7 (CH), 129.9 (CH), 129.2 (C), 128.6 (CH), 128.4 (CH), 128.4 (CH), 127.7 (CH), 127.3 (CH), 127.2 (CH), 127.2 (2CH), 126.6 (CH), 125.0 (CH), 124.1 (2CH), 123.9 (CH), 123.0 (CH), 117.6 (CH), 116.2 (C). **HRMS** (APCI+) *m/z* calcd. for $C_{28}H_{20}NO_3$ [M+H]: 418.1438; found: 418.1439.

Enantioselectivity of the product was determined by chiral HPLC analysis (Chiralpak IA3, IPA/hexane 20:80, 1.0 mL/min, λ=220 nm).

Racemic sample

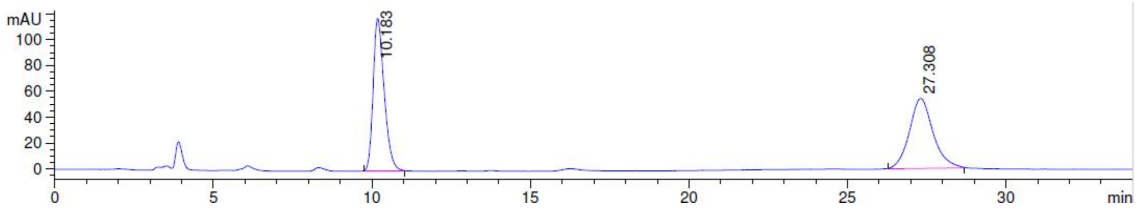

Peak #	RetTime [min]	Type	Width [min]	Area [mAU*s]	Height [mAU]	Area %
1	10.183	BB	0.3685	2846.26538	117.56312	50.3399
2	27.308	BB	0.7467	2807.83179	53.99488	49.6601

Chiral sample

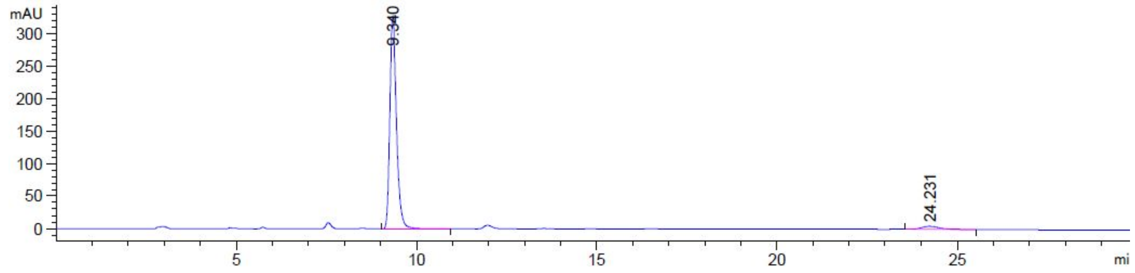

Peak #	RetTime [min]	Type	Width [min]	Area [mAU*s]	Height [mAU]	Area %
1	9.340	BB	0.1937	4211.76172	328.22903	96.2540
2	24.231	BB	0.5221	163.91087	4.64001	3.7460

Enantioselectivity of the remaining starting material (47%, 85.5:14.5 er) was determined by chiral HPLC analysis (Chiralpak IA3, IPA/hexane 20:80, 1.0 mL/min, λ=254 nm).

Racemic sample

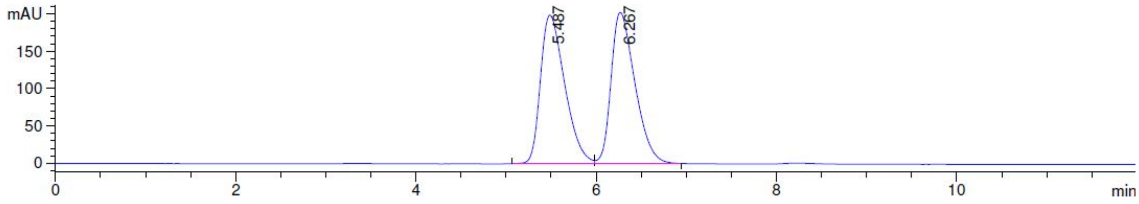

Peak #	RetTime [min]	Type	Width [min]	Area [mAU*s]	Height [mAU]	Area %
1	5.487	BV	0.2903	3729.95630	199.31519	49.9451
2	6.267	VB	0.2811	3738.15479	202.79050	50.0549

Chiral sample

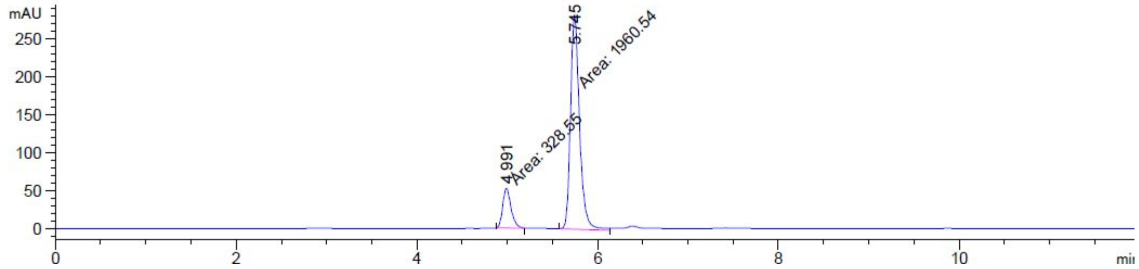

Peak #	RetTime [min]	Type	Width [min]	Area [mAU*s]	Height [mAU]	Area %
1	4.991	MM T	0.1056	328.54990	51.87346	14.3529
2	5.745	MM T	0.1157	1960.54163	282.29678	85.6471

(*S*,*E*)-2'-(4-(trifluoromethyl)styryl)-[1,1'-binaphthalen]-2-ol (3ag)

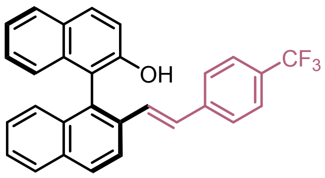

3ag

3ag was obtained by following the GP after column chromatography (Et_2O/hexane 10:90 to 15:85) as a white solid (38%, 95:5 er). **Rf:** 0.40 (Et_2O/hexane 40:60, light brown in *p*-anisaldehyde). **^{1}H NMR** (300 MHz, $CDCl_3$) δ: 8.10 – 8.02 (m, 2H), 7.98 (d, *J* = 8.9 Hz, 1H), 7.93 (t, *J* = 8.3 Hz, 2H), 7.50 (ddd, *J* = 8.1, 6.6, 1.5 Hz, 1H), 7.47 – 7.29 (m, 5H), 7.27 – 7.19 (m, 5H), 7.00 (d, *J* = 8.3 Hz, 1H), 6.88 (d, *J* = 16.3 Hz, 1H), 4.79 (s, 1H). **^{13}C NMR** (75 MHz, $CDCl_3$) δ: 151.4 (COH), 140.7 (C), 135.1 (C), 133.9 (C), 133.9 (C), 133.5 (C), 130.6 (CH), 130.1 (C), 129.8 (CH), 129.5 (CH), 129.2 (C), 128.8 (CH), 128.4 (CH), 128.3 (CH), 127.5 (CH), 127.1 (CH), 127.0 (CH) 126.9 (2CH), 126.5 (CH), 125.6 (q, *J* = 4.0 Hz, CH), 125.0 (CH), 123.8 (CH), 123.1 (CH), 117.6 (CH), 116.4 (C). **^{19}F NMR** (282 MHz, $CDCl_3$) δ: -62.6. **HRMS** (APCI+) *m/z* calcd. for $C_{29}H_{20}FO_3$ [M+H]: 441.1461; found: 441.1456.

Enantioselectivity of the product was determined by chiral HPLC analysis (Chiralpak IA3, IPA/hexane 20:80, 1.0 mL/min, λ=254 nm).

Racemic sample

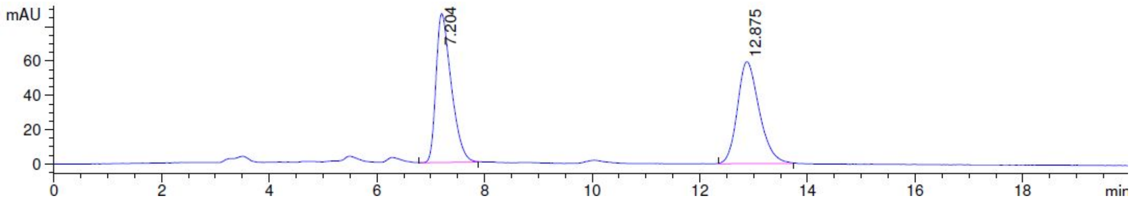

Peak #	RetTime [min]	Type	Width [min]	Area [mAU*s]	Height [mAU]	Area %
1	7.204	VB	0.2936	1706.81262	86.72709	50.4263
2	12.875	BB	0.4261	1677.95300	59.29704	49.5737

Chiral sample

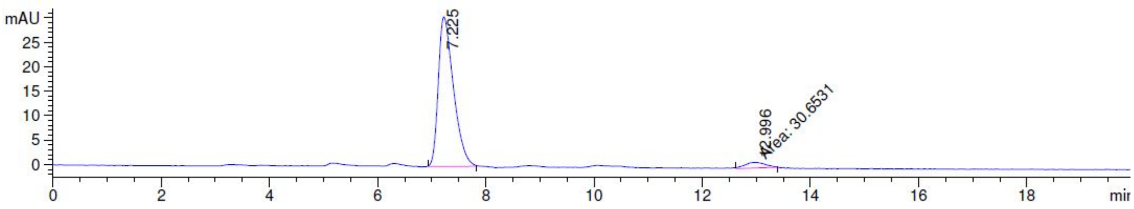

Peak #	RetTime [min]	Type	Width [min]	Area [mAU*s]	Height [mAU]	Area %
1	7.225	BB	0.2915	596.67700	30.60363	95.1137
2	12.996	MM	0.4413	30.65313	1.15780	4.8863

Enantioselectivity of the remaining starting material (46%, 90.5:9.5 er) was determined by chiral HPLC analysis (Chiralpak IA3, IPA/hexane 20:80, 1.0 mL/min, λ=254 nm).

Racemic sample

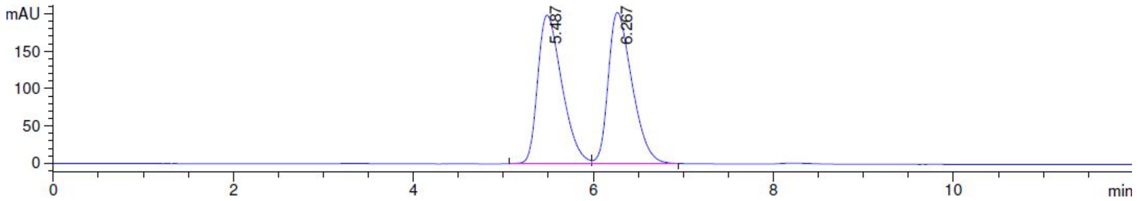

Peak #	RetTime [min]	Type	Width [min]	Area [mAU*s]	Height [mAU]	Area %
1	5.487	BV	0.2903	3729.95630	199.31519	49.9451
2	6.267	VB	0.2811	3738.15479	202.79050	50.0549

Chiral sample

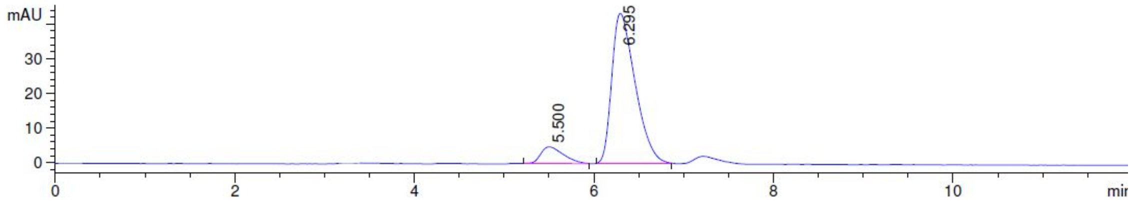

Peak #	RetTime [min]	Type	Width [min]	Area [mAU*s]	Height [mAU]	Area %
1	5.500	BP	0.2525	83.27390	4.79886	9.5725
2	6.295	BB	0.2797	786.65222	43.35854	90.4275

Ethyl (*R*,*E*)-7-(2'-hydroxy-[1,1'-binaphthalen]-2-yl)hept-6-enoate (3ah)

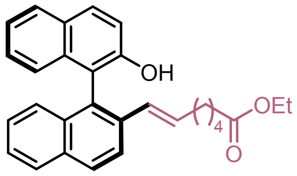

3ah

3ah was obtained by following the GP after column chromatography (AcOEt/hexane 5:95 to 15:85) as a light-yellow oil (29%, 95:5 er). **Rf:** 0.50 (AcOEt/hexane 25:75, reddish-brown in *p*-anisaldehyde). **^{1}H NMR** (300 MHz, $CDCl_3$) δ: 8.02 – 7.86 (m, 5H), 7.46 (t, *J* = 6.6, 1H), 7.38 – 7.19 (m, 5H), 7.00 (d, *J* = 8.6 Hz, 1H), 6.31 (dt, *J* = 15.7, 7.0 Hz, 1H), 6.10 (d, *J* = 15.9 Hz, 1H), 5.04 (s, 1H), 4.13 (q, *J* = 7.1 Hz, 2H), 2.20 (t, *J* = 7.7 Hz, 2H), 2.03 (q, *J* = 7.0 Hz, 2H), 1.59 – 1.48 (m, 2H), 1.36 (p, *J* = 7.4 Hz, 2H), 1.25 (t, *J* = 6.5 Hz, 3H). **^{13}C NMR** (75 MHz, $CDCl_3$) δ: 173.9 (CO), 151.4 (COH), 136.2 (C), 133.9 (C), 133.5 (C), 133.3 (CH), 133.3 (C), 130.1 (CH), 129.3 (CH), 128.2 (2CH), 128.1 (CH), 127.1 (CH), 126.8 (CH), 126.2 (CH), 126.1 (CH), 125.2 (CH), 123.6 (CH), 123.5 (CH), 117.6 (CH), 117.1 (C), 60.4 (OCH_2), 34.2 (CH_2), 32.8 (CH_2), 28.7 (CH_2), 24.3 (CH_2), 14.4 (CH_3). **HRMS** (APCI+) *m/z* calcd. for $C_{29}H_{29}O_3$ [M+H]: 425.2111; found: 425.2236.

Enantioselectivity of the product was determined by chiral HPLC analysis (Chiralpak IA3, IPA/hexane 20:80, flow rate = 1.0 mL/min, λ = 254 nm).

Racemic sample

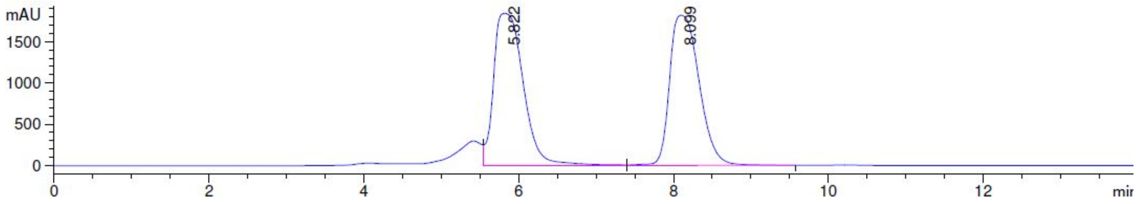

Peak #	RetTime [min]	Type	Width [min]	Area [mAU*s]	Height [mAU]	Area %
1	5.822	VV	0.4150	4.90817e4	1841.24683	50.4852
2	8.099	VB	0.4171	4.81383e4	1816.98108	49.5148

Chiral sample

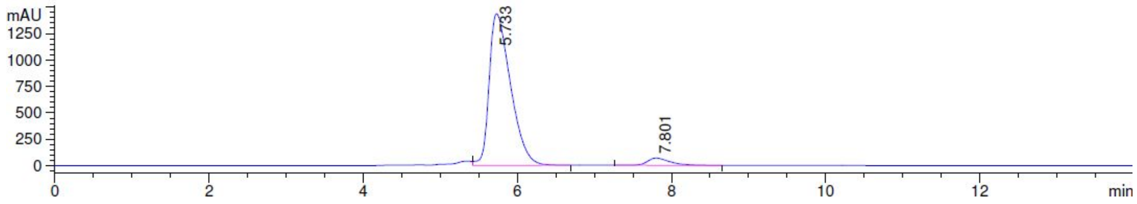

Peak #	RetTime [min]	Type	Width [min]	Area [mAU*s]	Height [mAU]	Area %
1	5.733	VP	0.2972	2.77314e4	1436.86243	95.1302
2	7.801	BB	0.3043	1419.58533	68.92339	4.8698

Enantioselectivity of the remaining starting material (40%, 96.5:3.5 er) was determined by chiral HPLC analysis (Chiralpak IA3, IPA/hexane 20:80, 1.0 mL/min, λ=254 nm).

Racemic sample

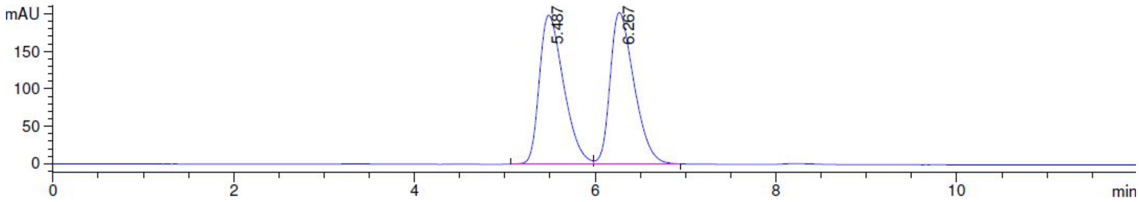

Peak #	RetTime [min]	Type	Width [min]	Area [mAU*s]	Height [mAU]	Area %
1	5.487	BV	0.2903	3729.95630	199.31519	49.9451
2	6.267	VB	0.2811	3738.15479	202.79050	50.0549

Chiral sample

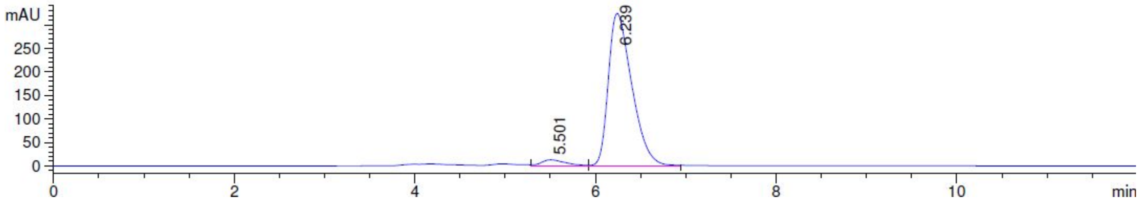

Peak #	RetTime [min]	Type	Width [min]	Area [mAU*s]	Height [mAU]	Area %
1	5.501	VV	0.2710	221.17145	12.22974	3.6648
2	6.239	VB	0.2737	5813.89648	323.39731	96.3352

(*R*,*E*)-2'-(oct-1-en-1-yl)-[1,1'-binaphthalen]-2-ol (3ai)

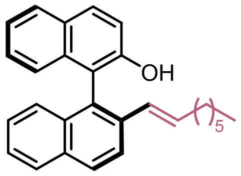

3ai

3ai was obtained by following the GP after column chromatography (AcOEt/hexane 3:97) as colorless granules (27%, 95.5:4.5 er). **Rf:** 0.65 (AcOEt/hexane 15:85, clear brown in *p*-anisaldehyde). **^{1}H NMR** (300 MHz, $CDCl_3$) δ: 8.00 – 7.84 (m, 5H), 7.47 – 7.39 (m, 1H), 7.37 – 7.14 (m, 5H), 6.98 (d, *J* = 8.4 Hz, 1H), 6.33 (dt, *J* = 15.7, 7.0 Hz, 1H), 6.07 (d, *J* = 15.8 Hz, 1H), 4.80 (s, 1H), 1.99 (q, *J* = 7.4 Hz, 2H), 1.36 – 1.09 (m, 8H), 0.83 (t, *J* = 6.8 Hz, 3H). **^{13}C NMR** (75 MHz, $CDCl_3$) δ: 151.3 (COH), 136.5 (C), 134.4 (CH), 133.9 (C), 133.6 (C), 133.3 (C), 130.1 (CH), 129.4 (CH), 129.2 (C), 128.2 (CH), 128.2 (CH), 127.5 (CH), 127.1 (CH), 126.8 (CH), 126.2 (CH), 126.1 (CH), 125.2 (CH), 123.7 (CH), 123.6 (CH), 117.6 (CH), 117.1 (C), 33.3 (CH_2), 31.8 (CH_2), 29.3 (CH_2), 28.8 (CH_2), 22.7 (CH_2), 14.2 (CH_3). **HRMS** (APCI+) *m/z* calcd. for $C_{28}H_{29}O$ [M+H]: 381.2213; found: 381.2217.

Enantioselectivity of the product was determined by chiral HPLC analysis (Chiralpak IA3, IPA/hexane 15:85, flow rate = 0.5 mL/min, λ=254 nm).

Racemic sample

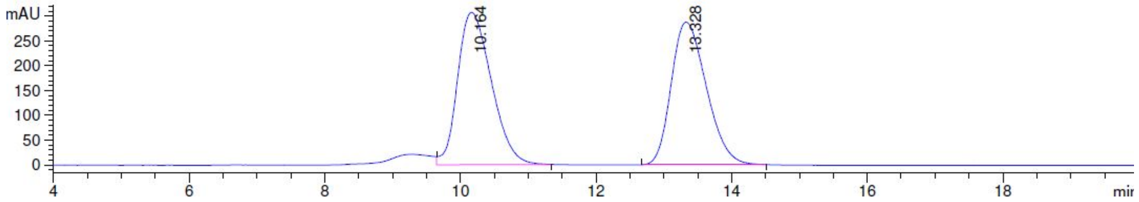

Peak #	RetTime [min]	Type	Width [min]	Area [mAU*s]	Height [mAU]	Area %
1	10.164	VB	0.5466	1.06297e4	306.48865	50.6600
2	13.328	BB	0.5665	1.03528e4	287.26288	49.3400

Chiral sample

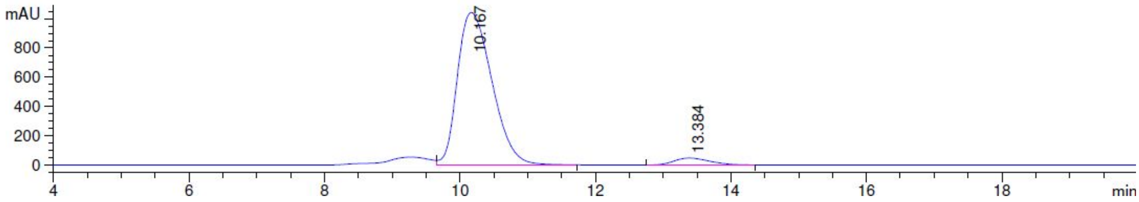

Peak #	RetTime [min]	Type	Width [min]	Area [mAU*s]	Height [mAU]	Area %
1	10.167	VB	0.5630	3.68670e4	1041.39417	95.6328
2	13.384	PB	0.5637	1683.59436	46.79809	4.3672

Enantioselectivity of the remaining starting material (43%, 86.5:13.5 er) was determined by chiral HPLC analysis (Chiralpak IA3, IPA/hexane 20:80, 1.0 mL/min, λ=254 nm).

Racemic sample

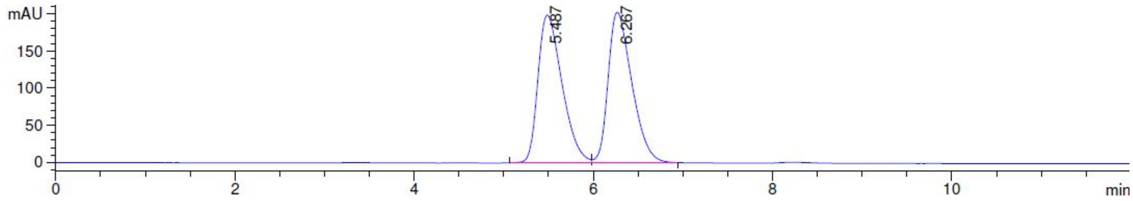

Peak #	RetTime [min]	Type	Width [min]	Area [mAU*s]	Height [mAU]	Area %
1	5.487	BV	0.2903	3729.95630	199.31519	49.9451
2	6.267	VB	0.2811	3738.15479	202.79050	50.0549

Chiral sample

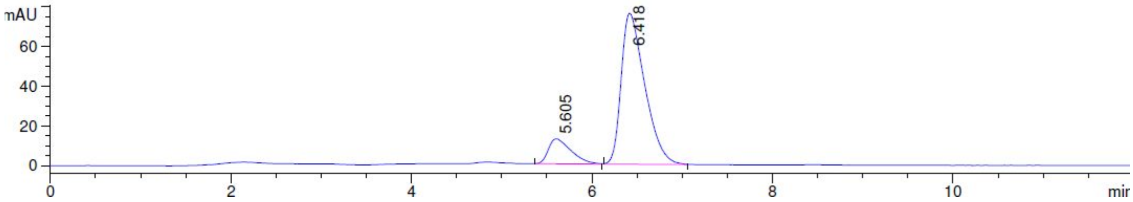

Peak #	RetTime [min]	Type	Width [min]	Area [mAU*s]	Height [mAU]	Area %
1	5.605	BB	0.2607	218.75723	12.59015	13.6813
2	6.418	BB	0.2758	1380.19495	76.03776	86.3187

Methyl (*S*,*E*)-3-(6'-chloro-2'-hydroxy-[1,1'-binaphthalen]-2-yl)acrylate (3ba)

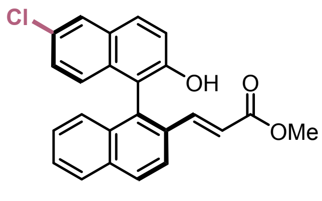

3ba

3ba was obtained by following the GP after column chromatography (AcOEt/hexane 15:85 to 25:75) as a white solid (51%, 95.5:4.5 er). **Rf:** 0.30 (AcOEt/hexane 25:75, brown in *p*-anisaldehyde). **^{1}H NMR** (300 MHz, $CDCl_3$) δ: 8.03 (d, *J* = 8.8 Hz, 1H), 7.96 – 7.91 (m, 2H), 7.89 – 7.84 (m, 2H), 7.54 (ddd, *J* = 8.1, 6.8, 1.3 Hz, 1H), 7.38 – 7.30 (m, 3H), 7.21 (d, *J* = 8.4 Hz, 1H), 7.15 (dd, *J* = 9.0, 2.2 Hz, 1H), 6.82 (d, *J* = 8.9 Hz, 1H), 6.50 (d, *J* = 16.0 Hz, 1H), 4.82 (s, 1H), 3.63 (s, 3H). **^{13}C NMR** (75 MHz, $CDCl_3$) δ: 167.0 (CO), 151.6 (COH), 141.9 (CH), 134.7 (C), 133.2 (C), 133.1 (C), 132.4 (C), 132.3 (C), 130.1 (CH), 129.9 (CH), 129.8 (C), 129.6 (C), 128.5 (CH), 127.9 (CH), 127.9 (CH), 127.8 (CH), 127.1 (CH), 126.7 (CH), 126.4 (CH), 123.4 (CH), 120.5 (CH), 118.9 (CH), 116.1 (C), 51.8 (OCH_3). **HRMS** (APCI+) *m/z* calcd. for $C_{24}H_{18}ClO_3$ [M+H]: 389.0939; found: 389.0949.

Enantioselectivity of the product was determined by chiral HPLC analysis (Chiralpak IB, IPA/hexane 15:85, 0.5 mL/min, λ=220 nm).

Racemic sample

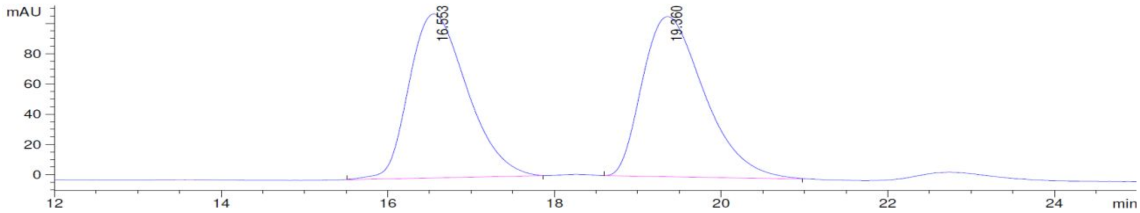

Peak #	RetTime [min]	Type	Width [min]	Area [mAU*s]	Height [mAU]	Area %
1	16.553	BB	0.7419	5238.35547	108.38425	48.9210
2	19.360	PB	0.7809	5469.43652	105.84270	51.0790

Chiral sample

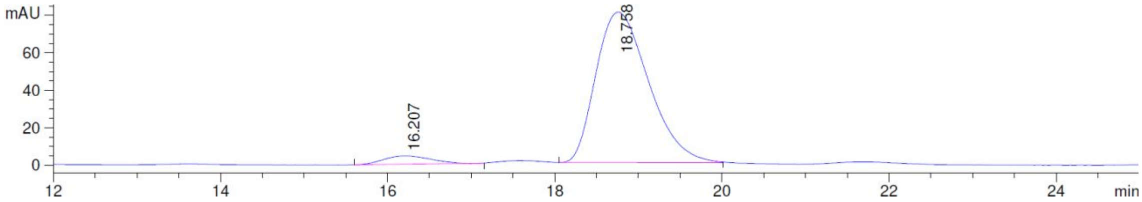

Peak #	RetTime [min]	Type	Width [min]	Area [mAU*s]	Height [mAU]	Area %
1	16.207	PP	0.4587	169.05441	4.41512	4.5668
2	18.758	PB	0.6783	3532.74756	80.44727	95.4332

Enantioselectivity of the remaining starting material (47%, 96.5:3.5 er) was determined by chiral HPLC analysis (Chiralpak IB, IPA/hexane 10:90, 0.5 mL/min, λ=220 nm).

Racemic sample

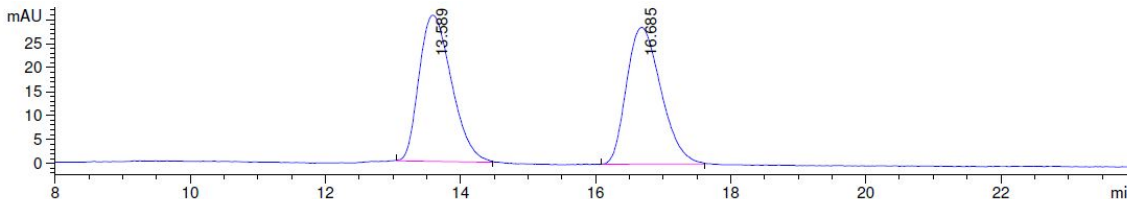

Peak #	RetTime [min]	Type	Width [min]	Area [mAU*s]	Height [mAU]	Area %
1	13.589	BB	0.4961	1019.74121	30.49773	50.2255
2	16.685	BB	0.5414	1010.58453	28.52160	49.7745

Chiral sample

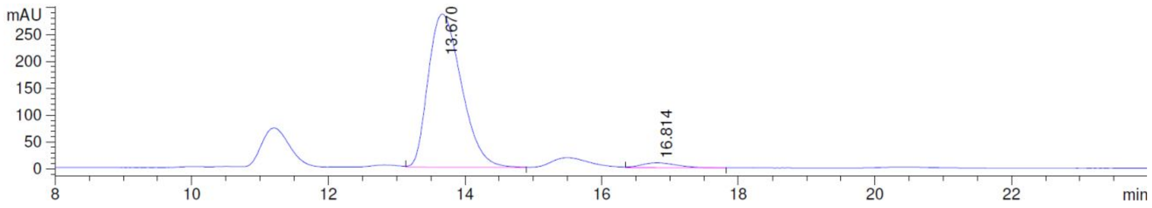

Peak #	RetTime [min]	Type	Width [min]	Area [mAU*s]	Height [mAU]	Area %
1	13.670	VB	0.5321	9627.31738	285.00226	96.7208
2	16.814	VB	0.4499	326.40692	9.02506	3.2792

Methyl (*S*,*E*)-3-(6'-acetyl-2'-hydroxy-[1,1'-binaphthalen]-2-yl)acrylate (3ca)

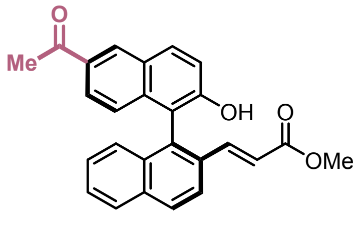

3ca

3ca was obtained by following the GP after column chromatography (AcOEt/hexane 20:80 to 45:75) as a white solid (41%, 95.5:4.5 er). **Rf:** 0.35 (AcOEt/hexane 45:55, red in *p*-anisaldehyde). **^{1}H NMR** (300 MHz, DMSO-d_6) δ: 10.17 (s, 1H), 8.68 (s, 1H), 8.22 (d, J = 8.8 Hz, 1H), 8.18 – 8.02 (m, 3H), 7.68 (dd, J = 8.7, 1.8 Hz, 1H), 7.58 – 7.48 (m, 2H), 7.32 (t, J = 7.7 Hz, 1H), 7.24 (d, J = 16.0 Hz, 1H), 7.06 (d, J = 8.3 Hz, 1H), 6.78 – 6.68 (m, 2H), 3.56 (s, 3H), 2.63 (s, 3H). **^{13}C NMR** (75 MHz, DMSO-d_6) δ: 197.2 (COMe), 166.6 (COOMe), 155.5 (COH), 142.4 (CH), 136.3 (C), 135.9 (C), 134.1 (C), 132.4 (C), 131.9 (CH), 131.4 (C), 130.9 (CH), 130.8 (C), 128.4 (CH), 128.2 (CH), 127.1 (CH), 126.9 (CH), 126.7 (C), 126.3 (CH), 124.6 (CH), 124.0 (CH), 123.3 (CH), 119.1 (CH), 118.7 (CH), 115.8 (C), 51.3 (OCH_3), 26.5 (CH_3). **HRMS** (APCI+) *m/z* calcd. for $C_{26}H_{21}O_4$ [M+H]: 397.1434; found: 397.1438.

Enantioselectivity of the product was determined by chiral HPLC analysis (Chiralpak IA3, IPA/hexane 15:85, 0.5 mL/min, λ=220 nm).

Racemic sample

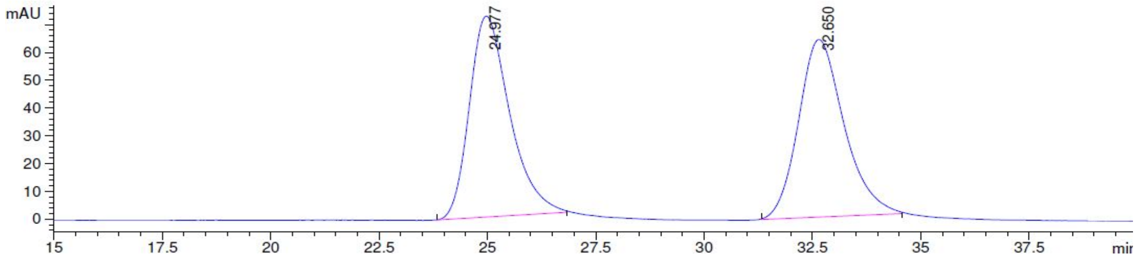

Peak #	RetTime [min]	Type	Width [min]	Area [mAU*s]	Height [mAU]	Area %
1	24.977	BB	0.9568	4676.63037	72.28939	50.0190
2	32.650	BB	1.0590	4673.08643	63.91262	49.9810

Chiral sample

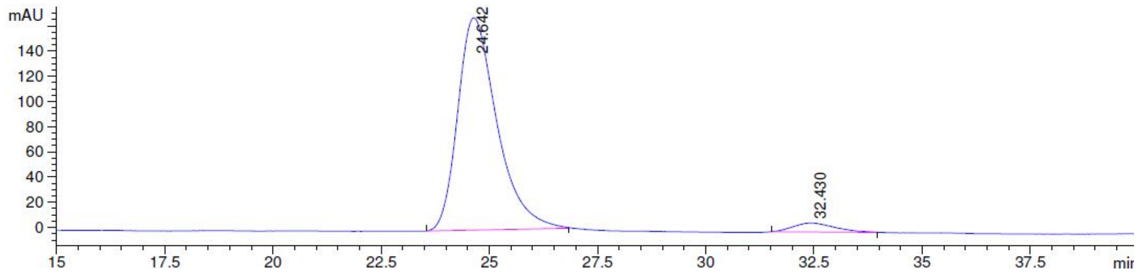

Peak #	RetTime [min]	Type	Width [min]	Area [mAU*s]	Height [mAU]	Area %
1	24.642	BB	0.9356	1.06536e4	168.51540	95.7050
2	32.430	BB	0.8035	478.10876	7.10103	4.2950

Enantioselectivity of the remaining starting material (42%, 97:3 er) was determined by chiral HPLC analysis (Chiralpak IB, IPA/hexane 15:85, 0.5 mL/min, λ=220 nm).

Racemic sample

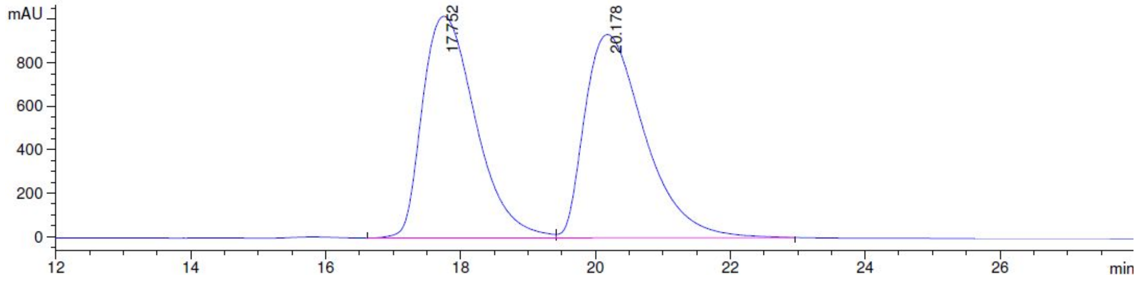

Peak #	RetTime [min]	Type	Width [min]	Area [mAU*s]	Height [mAU]	Area %
1	17.752	VV	0.8555	5.60477e4	1019.27789	49.2913
2	20.178	VB	0.9335	5.76593e4	934.92120	50.7087

Chiral sample

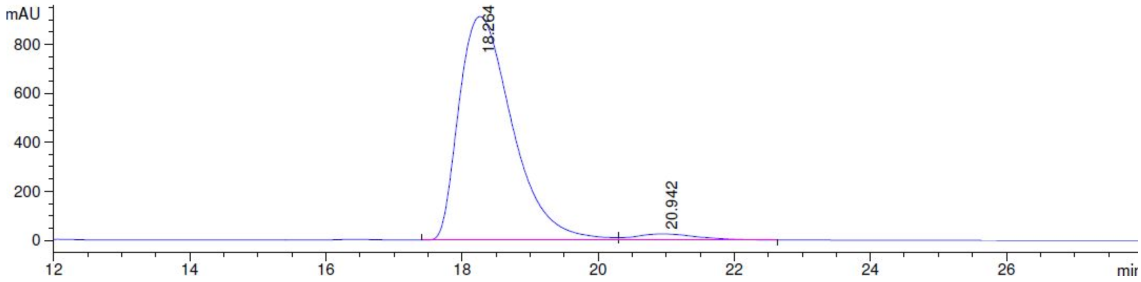

Peak #	RetTime [min]	Type	Width [min]	Area [mAU*s]	Height [mAU]	Area %
1	18.264	VB	0.8551	4.96928e4	912.80182	96.8721
2	20.942	BB	0.7484	1604.51465	25.55959	3.1279

Methyl (*S*,*E*)-3-(2'-hydroxy-6'-methoxy-[1,1'-binaphthalen]-2-yl)acrylate (3da)

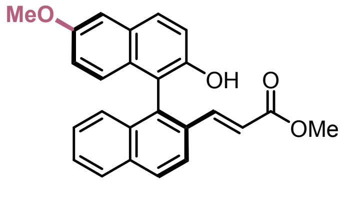

3da

3da was obtained by following the GP after column chromatography (AcOEt/hexane 20:80 to 30:70) as a white solid (55%, 97.5:2.5 er). **Rf:** 0.24 (AcOEt/hexane 25:75, grey in *p*-anisaldehyde). **^{1}H NMR** (300 MHz, $CDCl_3$) δ: 8.00 (d, *J* = 8.9 Hz, 1H), 7.97 – 7.88 (m, 2H), 7.84 (d, *J* = 8.9 Hz, 1H), 7.52 (ddd, *J* = 8.2, 6.5, 1.6 Hz, 1H), 7.40 (d, *J* = 16.0 Hz, 1H), 7.35 – 7.24 (m, 3H), 7.21 (d, *J* = 2.6 Hz, 1H), 6.90 (dd, *J* = 9.1, 2.6 Hz, 1H), 6.79 (d, *J* = 9.2 Hz, 1H), 6.49 (d, *J* = 16.0 Hz, 1H), 4.67 (s, 1H), 3.90 (s, 3H), 3.62 (s, 3H). **^{13}C NMR** (75 MHz, $CDCl_3$) δ: 167.1 (CO), 156.2 (COMe), 149.8 (COH), 142.3 (CH), 134.7 (C), 133.3 (C), 133.0 (C), 130.2 (C), 129.8 (CH), 129.5 (CH), 129.2 (C), 128.4 (CH), 127.8 (CH), 127.7 (CH), 127.0 (CH), 126.3 (CH), 123.4 (CH), 120.2 (CH), 119.5 (CH), 118.1 (CH), 116.1 (C), 106.9 (CH), 55.5 (OCH_3), 51.7 (OCH_3). **HRMS** (APCI+) *m/z* calcd. for $C_{25}H_{21}O_4$ [M+H]: 385.1434; found: 385.1437.

Enantioselectivity of the product was determined by chiral HPLC analysis (Chiralpak IB, IPA/hexane 15:85, 0.5 mL/min, λ=254 nm).

Racemic sample

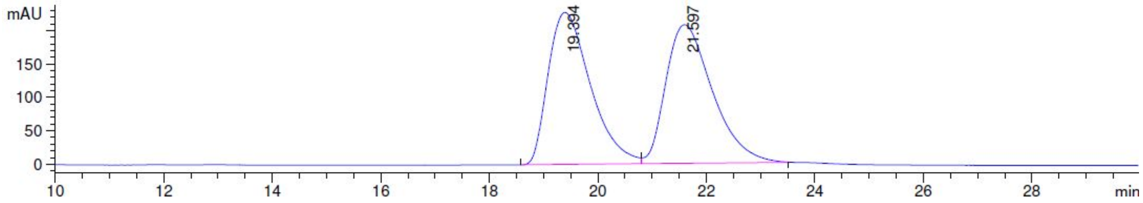

Peak #	RetTime [min]	Type	Width [min]	Area [mAU*s]	Height [mAU]	Area %
1	19.394	BV	0.8266	1.23270e4	227.34145	49.8540
2	21.597	VB	0.9078	1.23993e4	207.30821	50.1460

Chiral sample

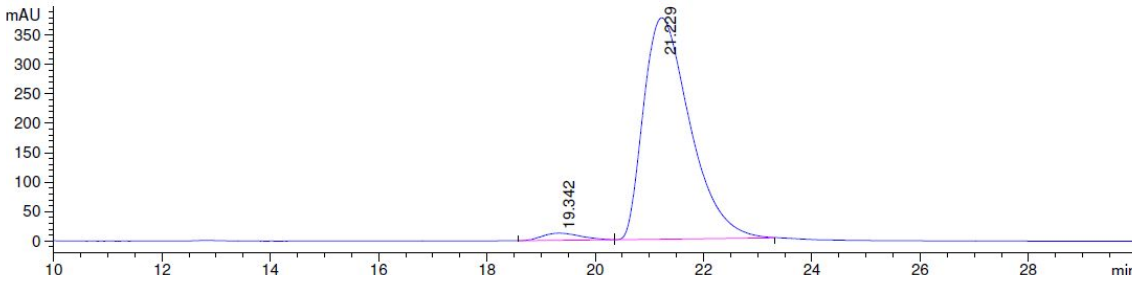

Peak #	RetTime [min]	Type	Width [min]	Area [mAU*s]	Height [mAU]	Area %
1	19.342	PV	0.5920	596.37805	12.16314	2.5917
2	21.229	VB	0.9092	2.24146e4	376.17514	97.4083

Enantioselectivity of the remaining starting material (26%, 80:20 er) was determined by chiral HPLC analysis (Chiralpak IB, IPA/hexane 15:85, 1.0 mL/min, λ=254 nm).

Racemic sample

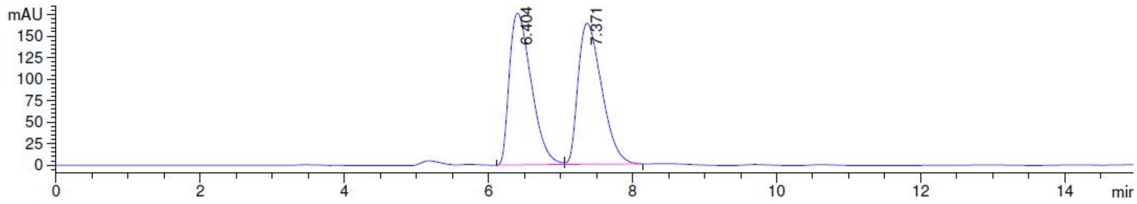

Peak #	RetTime [min]	Type	Width [min]	Area [mAU*s]	Height [mAU]	Area %
1	6.404	PV	0.3336	3747.64600	176.56641	49.9763
2	7.371	VP	0.3605	3751.20581	164.22641	50.0237

Chiral sample

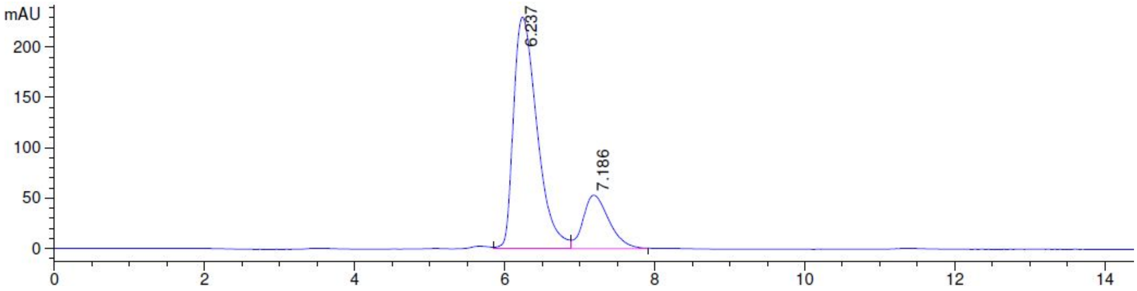

Peak #	RetTime [min]	Type	Width [min]	Area [mAU*s]	Height [mAU]	Area %
1	6.237	VV	0.3411	5033.82227	230.24834	79.7946
2	7.186	VB	0.3725	1274.65479	53.02093	20.2054

Methyl (*S*,*E*)-3-(2'-hydroxy-3'-methyl-[1,1'-binaphthalen]-2-yl)acrylate (3ea)

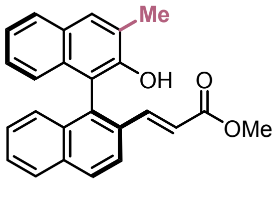

3ea

3ea was obtained by following the GP (48 h reaction time) after column chromatography (AcOEt/hexane 12:88 to 20:80) as a white solid (44%, 96.5:3.5 er). **Rf:** 0.35 (AcOEt/hexane 20:80, brown in *p*-anisaldehyde). **^{1}H NMR** (300 MHz, $CDCl_3$) δ: 8.03 (d, *J* = 8.7 Hz, 1H), 7.94 (d, *J* = 8.8 Hz, 2H), 7.81 (d, *J* = 9.7 Hz, 2H), 7.53 (ddd, *J* = 8.1, 6.6, 1.4 Hz, 1H), 7.39 (d, *J* = 16.0 Hz, 1H), 7.34 – 7.22 (m, 3H), 7.15 (ddd, *J* = 8.3, 6.9, 1.4 Hz, 1H), 6.82 (d, *J* = 8.4 Hz, 1H), 6.51 (d, *J* = 16.0 Hz, 1H), 4.71 (s, 1H), 3.63 (s, 3H), 2.52 (s, 3H). **^{13}C NMR** (75 MHz, $CDCl_3$) δ: 167.1 (CO), 150.7 (COH), 142.3 (CH), 134.8 (C), 133.4 (C), 133.4 (C), 133.2 (C), 132.8 (C), 130.3 (CH), 129.8 (CH), 129.2 (C), 128.4 (CH), 127.8 (CH), 127.7 (CH), 127.6 (CH), 127.0 (CH), 126.7 (C), 126.1 (CH), 124.6 (CH), 123.7 (CH), 123.4 (CH), 120.3 (CH), 115.2 (C), 51.7 (OCH_3), 17.1 (CH_3). **HRMS** (APCI+) *m/z* calcd. for $C_{25}H_{21}O_3$ [M+H]: 369.1485; found: 369.1485.

Enantioselectivity of the product was determined by chiral HPLC analysis (Chiralpak IA3, IPA/hexane 15:85, flow rate = 1.0 mL/min, λ=254 nm).

Racemic sample

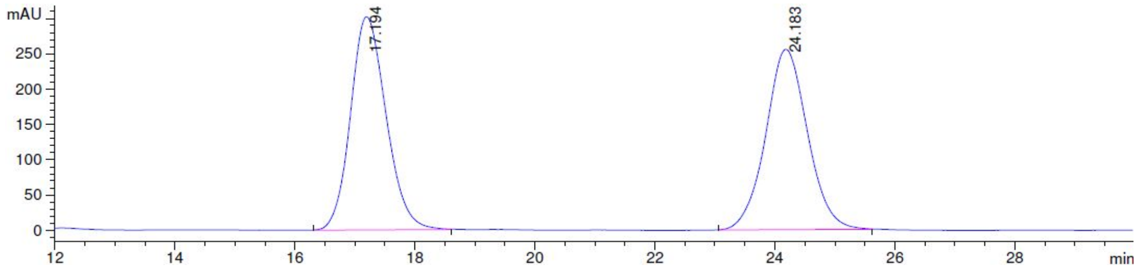

Peak #	RetTime [min]	Type	Width [min]	Area [mAU*s]	Height [mAU]	Area %
1	17.194	BB	0.6370	1.24464e4	301.85825	50.0970
2	24.183	BB	0.7420	1.23982e4	255.57239	49.9030

Chiral sample

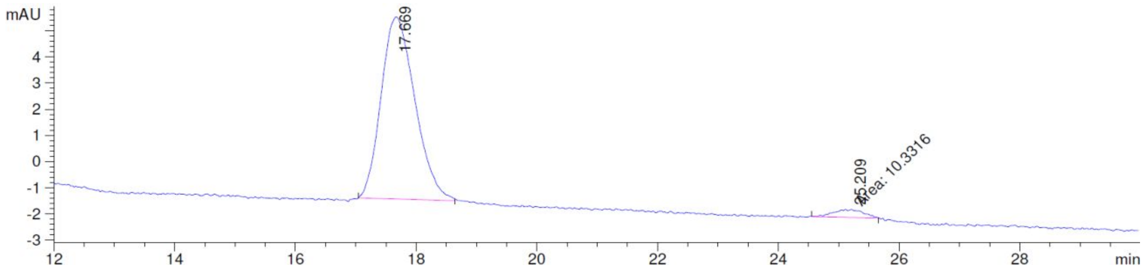

Peak #	RetTime [min]	Type	Width [min]	Area [mAU*s]	Height [mAU]	Area %
1	17.669	PB	0.4890	271.03830	6.94926	96.3281
2	25.209	MM	0.5630	10.33162	3.05832e-1	3.6719

Enantioselectivity of the remaining starting material (55%, 81.5:18.5 er) was determined by chiral HPLC analysis (Chiralpak IA3, IPA/hexane 20:80, 1.0 mL/min, λ=220.4 nm).

Racemic sample

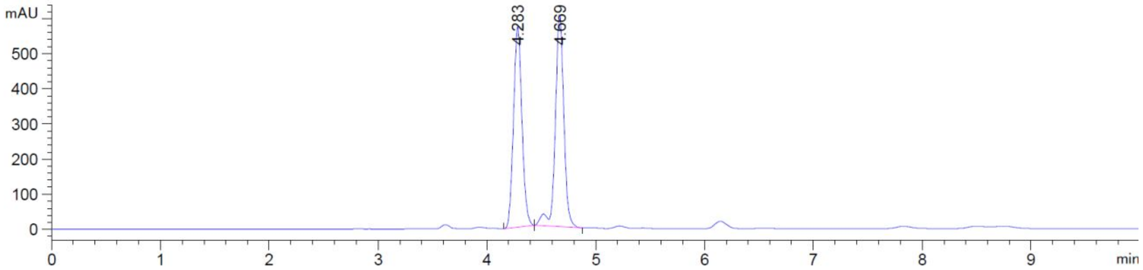

Peak #	RetTime [min]	Type	Width [min]	Area [mAU*s]	Height [mAU]	Area %
1	4.283	BB	0.0827	3084.73853	573.62024	48.2655
2	4.669	VB R	0.0811	3306.45117	603.55884	51.7345

Chiral sample

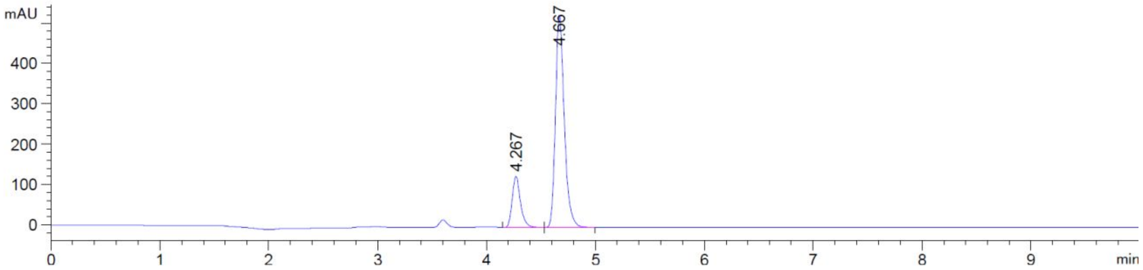

Peak #	RetTime [min]	Type	Width [min]	Area [mAU*s]	Height [mAU]	Area %
1	4.267	BV	0.0820	675.00427	127.05048	18.5197
2	4.667	VB	0.0858	2969.77856	526.91931	81.4803

Methyl (*S*,*E*)-3-(3'-fluoro-2'-hydroxy-[1,1'-binaphthalen]-2-yl)acrylate (3fa)

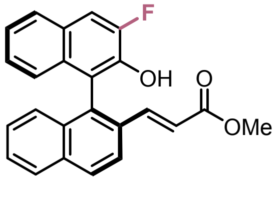

3fa

3fa was obtained by following the GP (at rt for 17 h) after column chromatography (AcOEt/hexane 5:95 to 20:80) as an orange solid (40%, 92:8 er). **Rf:** 0.38 (AcOEt/hexane 25:75, dark brown in *p*-anisaldehyde). **^{1}H NMR** (300 MHz, $CDCl_3$) δ: 8.02 (d, *J* = 8.7 Hz, 1H), 7.96 – 7.90 (m, 2H), 7.83 (d, *J* = 8.2 Hz, 1H), 7.70 (d, *J* = 11.1 Hz, 1H), 7.52 (ddd, *J* = 8.1, 6.8, 1.2 Hz, 1H), 7.43 – 7.33 (m, 3H), 7.29 (dd, *J* = 7.0, 1.4 Hz, 1H), 7.24 – 7.15 (m, 2H), 6.92 (d, *J* = 8.5 Hz, 1H), 6.51 (d, *J* = 15.9 Hz, 1H), 5.18 (s, 1H), 3.64 (s, 3H). **^{13}C NMR** (75 MHz, $CDCl_3$) δ: 167.2 (CO), 151.1 (d, *J* = 245.1 Hz, CF), 142.3 (CH), 141.6 (d, *J* = 16.0 Hz, COH), 134.6 (C), 133.0 (C), 132.4 (C), 130.9 (C), 129.7 (CH), 128.6 (C), 128.4 (CH), 127.7 (d, *J* = 5.3 Hz, CH), 127.5 (d, *J* = 8.0 Hz, CH), 126.8 (C), 126.8 (CH), 126.4 (d, *J* = 2.5 Hz, CH), 125.1 (CH), 125.1 (CH), 125.0 (CH), 123.3 (CH), 120.1 (CH), 119.4 (C), 112.8 (d, *J* = 17.6 Hz, CH), 51.7 (OCH_3). **^{19}F NMR** (282 MHz, $CDCl_3$) δ: -136.1. **HRMS** (APCI+) *m/z* calcd. for $C_{24}H_{17}FO_3$ [M+H]: 373.1234; found: 373.1235.

Enantioselectivity of the product was determined by chiral HPLC analysis (Chiralpak IF3, IPA/hexane 15:85, 0.5 mL/min, λ=220 nm).

Racemic sample

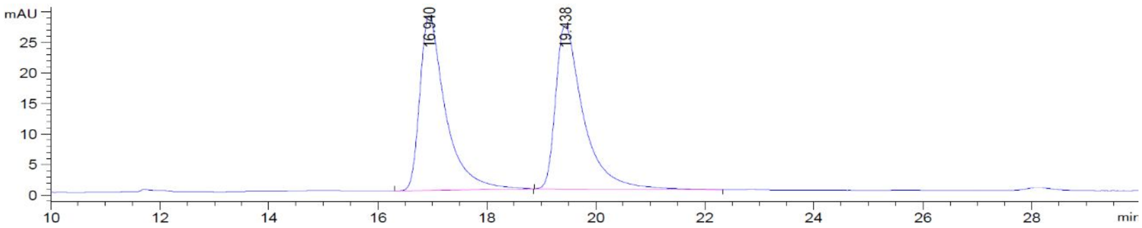

Peak #	RetTime [min]	Type	Width [min]	Area [mAU*s]	Height [mAU]	Area %
1	16.940	BB	0.4809	934.17438	28.61139	49.7473
2	19.438	BB	0.5173	943.66437	26.77050	50.2527

Chiral sample

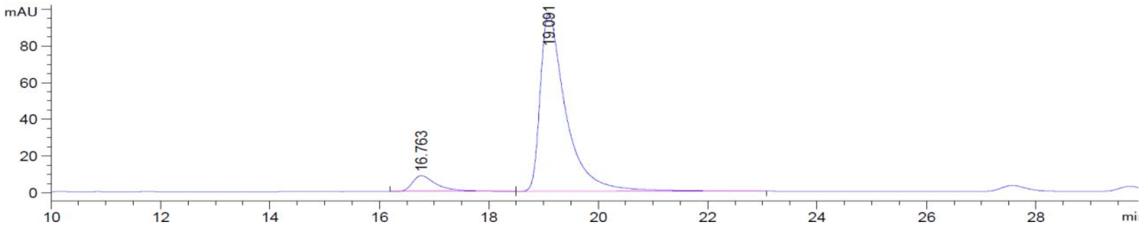

Peak #	RetTime [min]	Type	Width [min]	Area [mAU*s]	Height [mAU]	Area %
1	16.763	BB	0.4552	270.06168	8.67576	7.8534
2	19.091	BB	0.4785	3168.71436	97.15812	92.1466

Enantioselectivity of the remaining starting material (36%, 98:2 er) was determined by chiral HPLC analysis (Chiralpak IA3, IPA/hexane 15:85, 0.5 mL/min, λ=220 nm).

Racemic sample

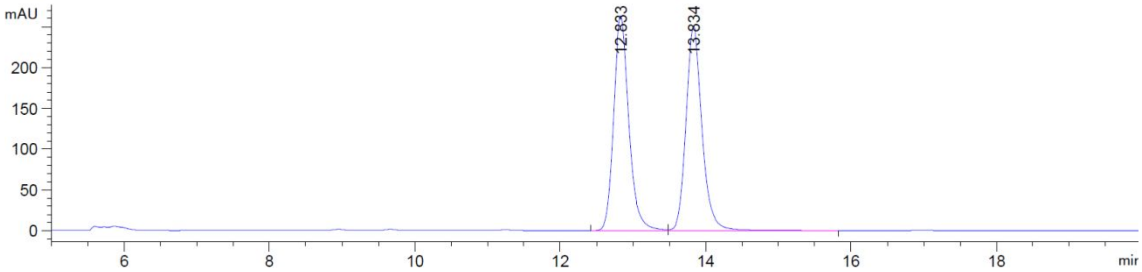

Peak #	RetTime [min]	Type	Width [min]	Area [mAU*s]	Height [mAU]	Area %
1	12.833	BV	0.2209	3807.11938	262.92841	49.8520
2	13.834	VB	0.2345	3829.73071	250.33888	50.1480

Chiral sample

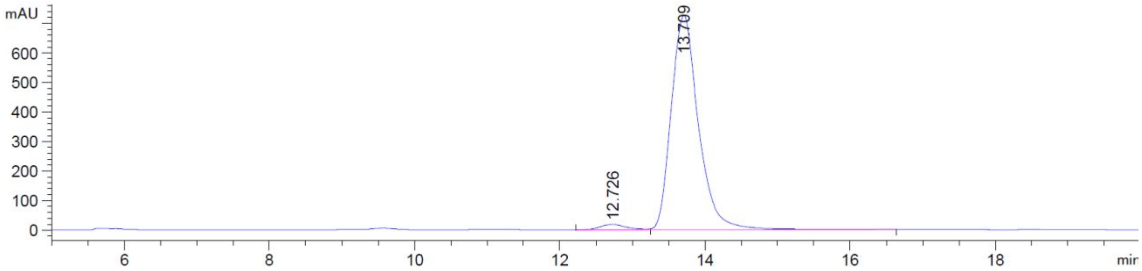

Peak #	RetTime [min]	Type	Width [min]	Area [mAU*s]	Height [mAU]	Area %
1	12.726	BV E	0.3713	430.57898	17.98468	2.2273
2	13.709	VB R	0.4018	1.89016e4	725.84869	97.7727

Methyl (*S*,*E*)-3-(1-(2-hydroxyphenanthren-1-yl)naphthalen-2-yl)acrylate (3ga)

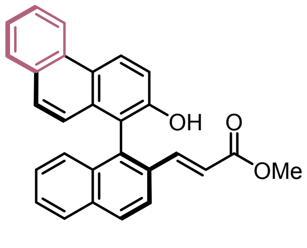

3ga

3ga was obtained by following the GP as a white solid (45%, 95.5:4.5 er). **Rf:** 0.30 (AcOEt/hexane 25:75, brown in *p*-anisaldehyde). **^{1}H NMR** (300 MHz, $CDCl_3$) δ: 8.83 (d, *J* = 9.1 Hz, 1H), 8.72 (d, *J* = 8.1 Hz, 1H), 8.05 (d, *J* = 8.7 Hz, 1H), 7.94 (d, *J* = 2.1 Hz, 2H), 7.80 (d, *J* = 7.9 Hz, 1H), 7.69 (ddd, *J* = 8.4, 7.0, 1.5 Hz, 1H), 7.59 – 7.46 (m, 4H), 7.42 (d, *J* = 16.0 Hz, 1H), 7.34 – 7.24 (m, 2H), 6.87 (d, *J* = 9.2 Hz, 1H), 6.51 (d, *J* = 16.0 Hz, 1H), 4.80 (s, 1H), 3.60 (s, 3H). **^{13}C NMR** (75 MHz, $CDCl_3$) δ: 167.0 (CO), 152.2 (COH), 142.1 (CH), 134.7 (C), 133.4 (C), 133.3 (C), 133.1 (C), 132.6 (C), 130.9 (C), 130.7 (C), 129.9 (CH), 128.7 (CH), 128.4 (CH), 128.3 (CH), 127.8 (CH), 127.6 (CH), 127.1 (CH), 126.9 (CH), 126.0 (CH), 125.4 (CH), 125.1 (C), 123.9 (CH), 123.5 (CH), 122.5 (CH), 120.5 (CH), 118.0 (C), 116.7(CH), 51.7 (OCH_3). **HRMS** (APCI+) *m/z* calcd. for $C_{28}H_{21}O_3$ [M+H]: 405.1485; found: 405.1489.

Enantioselectivity of the product was determined by chiral HPLC analysis (Chiralpak IA3, IPA/hexane 15:85, flow rate = 1.0 mL/min, λ=254 nm).

Racemic sample

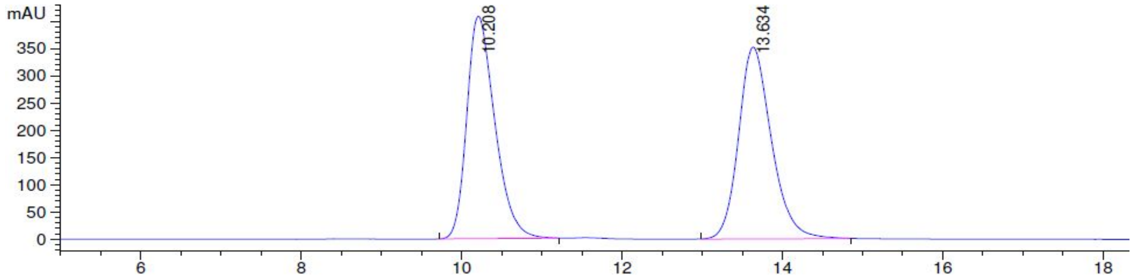

Peak #	RetTime [min]	Type	Width [min]	Area [mAU*s]	Height [mAU]	Area %
1	10.208	BB	0.3698	9939.71289	408.65201	49.7881
2	13.634	BB	0.4302	1.00243e4	352.04868	50.2119

Chiral sample

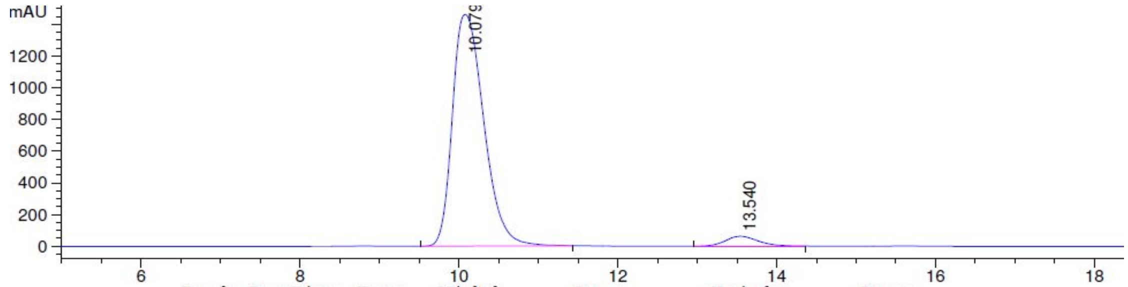

Peak #	RetTime [min]	Type	Width [min]	Area [mAU*s]	Height [mAU]	Area %
1	10.079	PB	0.4303	4.01174e4	1461.13879	95.6396
2	13.540	BB	0.4338	1829.03894	63.55130	4.3604

Enantioselectivity of the remaining starting material (48%, 95.5:4.5 er) was determined by chiral HPLC analysis (Chiralpak IA3, IPA/hexane 20:80, 1.0 mL/min, λ=254 nm).

Racemic sample

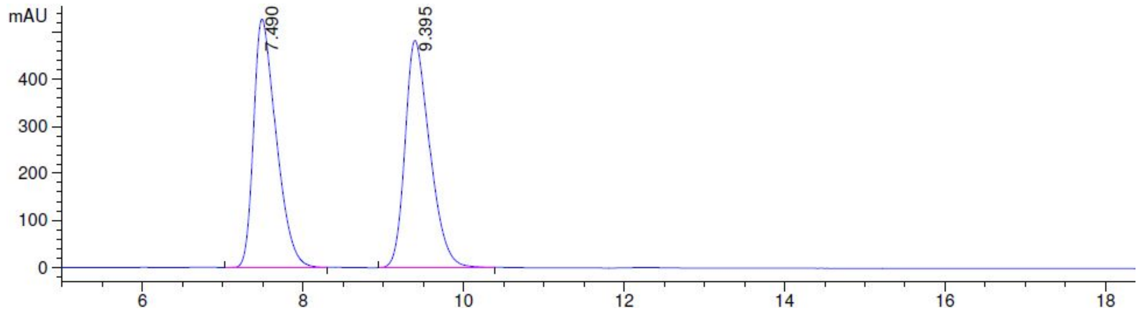

Peak #	RetTime [min]	Type	Width [min]	Area [mAU*s]	Height [mAU]	Area %
1	7.490	VB	0.2967	1.03700e4	529.09369	49.8861
2	9.395	BB	0.3252	1.04174e4	483.74457	50.1139

Chiral sample

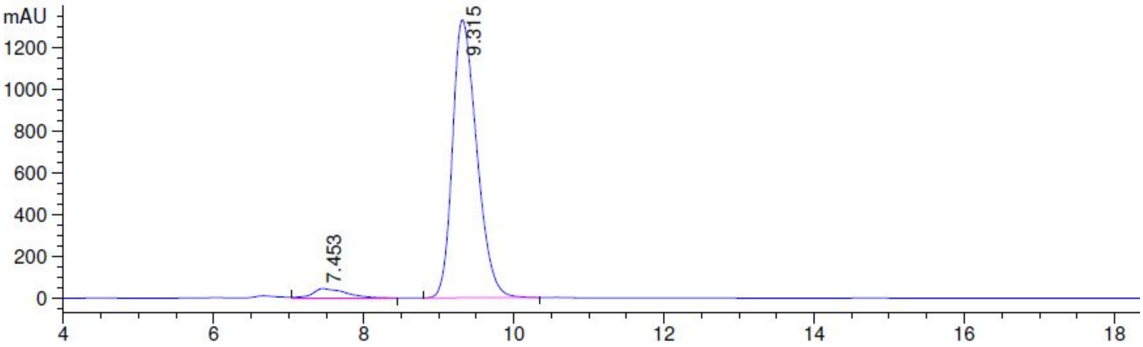

Peak #	RetTime [min]	Type	Width [min]	Area [mAU*s]	Height [mAU]	Area %
1	7.453	VB	0.4094	1365.33911	44.31547	4.3669
2	9.315	PB	0.3463	2.99005e4	1330.05212	95.6331

Methyl (*R*,*E*)-3-(1-(2-hydroxy-6-methoxyphenyl)naphthalen-2-yl)acrylate (3ha)

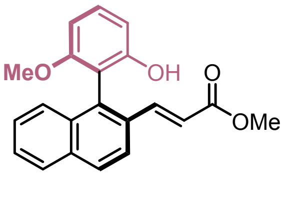

3ha

3ha was obtained by following the GP (at 50 ºC for 26 h, Boc-Val-OH) after column chromatography (AcOEt/hexane 25:75) as a white solid 43%, 95.5:4.5 er). **Rf:** 0.30 (AcOEt/hexane 25:75, pink in *p*-anisaldehyde). **^{1}H NMR** (500 MHz, $CDCl_3$) δ: 7.93 (d, *J* = 8.7 Hz, 1H), 7.88 (d, *J* = 8.2 Hz, 1H), 7.85 (d, *J* = 8.7 Hz, 1H), 7.59 (d, *J* = 16.0 Hz, 1H), 7.56 – 7.49 (m, 1H), 7.48 (d, *J* = 8.4 Hz, 1H), 7.45 – 7.35 (m, 2H), 6.73 (d, *J* = 8.3 Hz, 1H), 6.67 (d, *J* = 8.3 Hz, 1H), 6.50 (d, *J* = 15.9 Hz, 1H), 4.42 (s, 1H), 3.73 (s, 1H), 3.62 (s, 3H). **^{13}C NMR** (126 MHz, $CDCl_3$) δ: 167.4 (CO), 158.3 (COH), 154.3 (C), 143.0 (CH), 134.6 (C), 132.9 (C), 132.4 (C), 131.9 (C), 130.6 (CH), 129.4 (CH), 128.4 (CH), 127.5 (CH), 127.4 (CH), 126.6 (CH), 123.3 (CH), 119.6 (CH), 112.1 (C), 108.8 (CH), 103.6 (CH), 56.0 (OCH_3), 51.8 (OCH_3). **HRMS** (APCI+) *m/z* calcd. for $C_{21}H_{19}O_4$ [M+H]: 335.1278; found: 335.1277.

Enantioselectivity of the product was determined by chiral HPLC analysis (Chiralpak IE3, IPA/hexane 15:85, flow rate = 0.5 mL/min, λ=220 nm).

Chiral Sample

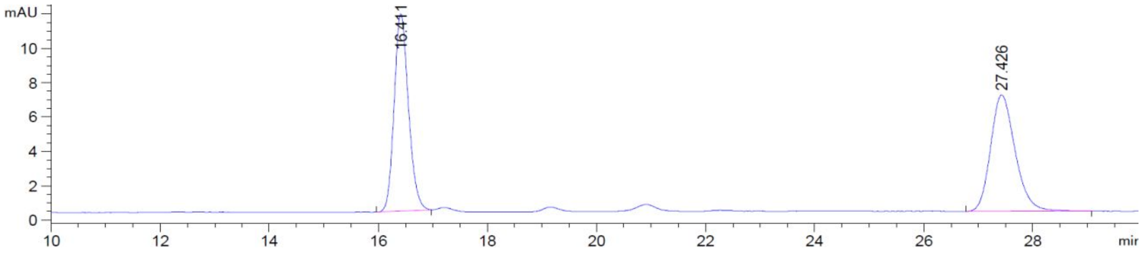

Peak #	RetTime [min]	Type	Width [min]	Area [mAU*s]	Height [mAU]	Area %
1	16.411	BB	0.2804	208.79288	11.46621	49.5737
2	27.426	BB	0.4810	212.38356	6.78787	50.4263

Racemic Sample

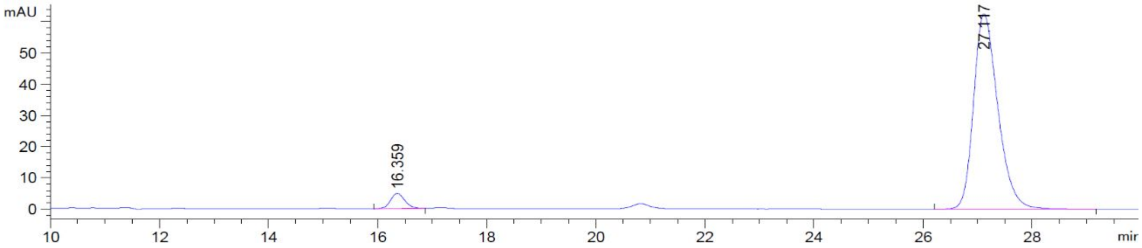

Peak #	RetTime [min]	Type	Width [min]	Area [mAU*s]	Height [mAU]	Area %
1	16.358	BB	0.2749	88.58233	4.90111	4.3773
2	27.117	BB	0.4700	1935.10547	62.71963	95.6227

Enantioselectivity of the remaining starting material (30%, >99.5:0.5 er) was determined by chiral HPLC analysis (Chiralpak IA3, IPA/hexane 20:80, 0.5 mL/min, λ=220 nm).

Chiral Sample

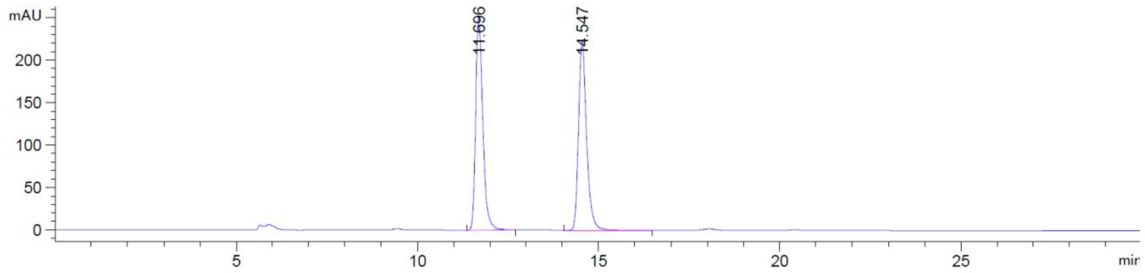

Peak #	RetTime [min]	Type	Width [min]	Area [mAU*s]	Height [mAU]	Area %
1	11.696	BB	0.2073	3433.65259	251.52771	49.8298
2	14.547	BB	0.2346	3457.11377	223.34483	50.1702

Chiral Sample

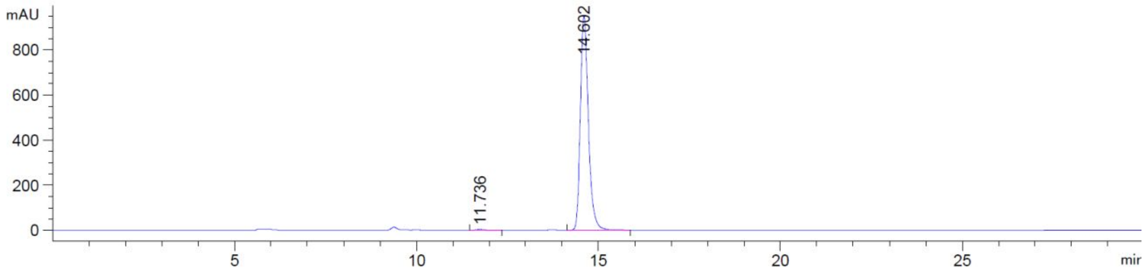

Peak #	RetTime [min]	Type	Width [min]	Area [mAU*s]	Height [mAU]	Area %
1	11.736	BB	0.2071	50.63848	3.66642	0.3474
2	14.602	BB	0.2323	1.45277e4	950.50488	99.6526

Methyl (*S*,*E*)-3-(1-(2-hydroxy-6-methylphenyl)naphthalen-2-yl)acrylate (3ia)

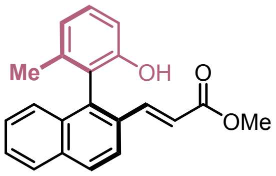

3ia

3ia was obtained by following the GP (at 60 ºC for 48 h) after column chromatography (AcOEt/hexane 15:85 to 20:80) as a white solid (40%, 94.5:5.5 er). **Rf:** 0.33 (AcOEt/hexane 25:75, light orange in *p*-anisaldehyde). **^{1}H NMR** (300 MHz, $CDCl_3$) δ: 7.97 – 7.84 (m, 3H), 7.57 – 7.48 (m, 2H), 7.45 – 7.38 (m, 2H), 7.32 (t, *J* = 7.8 Hz, 1H), 6.97 (d, *J* = 7.5 Hz, 1H), 6.91 (d, *J* = 7.6 Hz, 1H), 6.50 (d, *J* = 16.0 Hz, 1H), 4.31 (s, 1H), 3.72 (s, 3H), 1.82 (s, 3H). **^{13}C NMR** (75 MHz, $CDCl_3$) δ: 167.2 (CO), 153.4 (COH), 142.2 (CH), 138.6 (C), 134.6 (C), 134.5 (C), 132.6 (C), 131.9 (C), 129.8 (CH), 129.5 (CH), 128.4 (CH), 127.7 (CH), 127.7 (CH), 126.4 (CH), 123.4 (CH), 123.0 (C), 122.6 (CH), 120.1 (CH), 113.3 (CH), 51.8 (OCH_3), 20.0 (CH_3). **HRMS** (APCI+) *m/z* calcd. for $C_{21}H_{19}O_3$ [M+H]: 319.1329; found: 319.1340.

Enantioselectivity of the product was determined by chiral HPLC analysis (Chiralpak IE3, IPA/hexane 15:85, flow rate = 1.0 mL/min, λ=254 nm).

Racemic sample

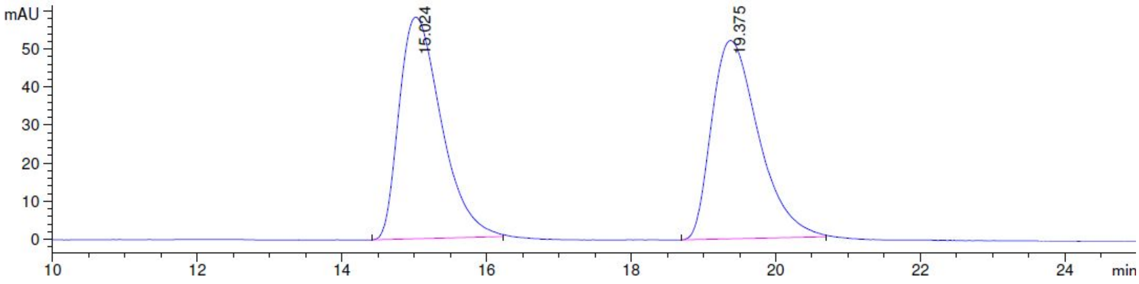

Peak #	RetTime [min]	Type	Width [min]	Area [mAU*s]	Height [mAU]	Area %
1	15.024	PB	0.6214	2365.06006	58.28360	50.0930
2	19.375	PB	0.6990	2356.28149	52.16713	49.9070

Chiral sample

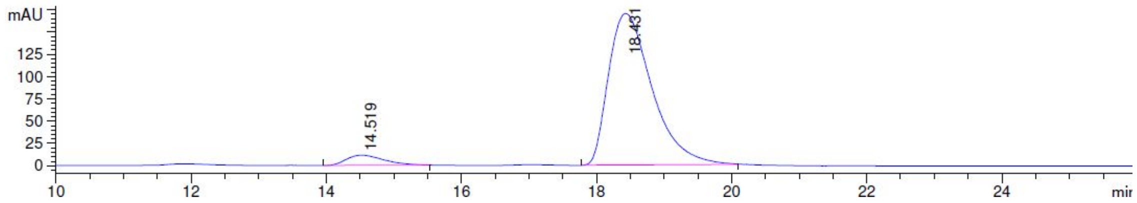

Peak #	RetTime [min]	Type	Width [min]	Area [mAU*s]	Height [mAU]	Area %
1	14.519	PB	0.5493	437.21213	11.25221	5.4247
2	18.431	PB	0.6910	7622.46436	170.03726	94.5753

Enantioselectivity of the remaining starting material (38%, 96.5:3.5 er) was determined by chiral HPLC analysis (Chiralpak IA3, IPA/hexane 15:85, 0.5 mL/min, λ=254 nm).

Racemic sample

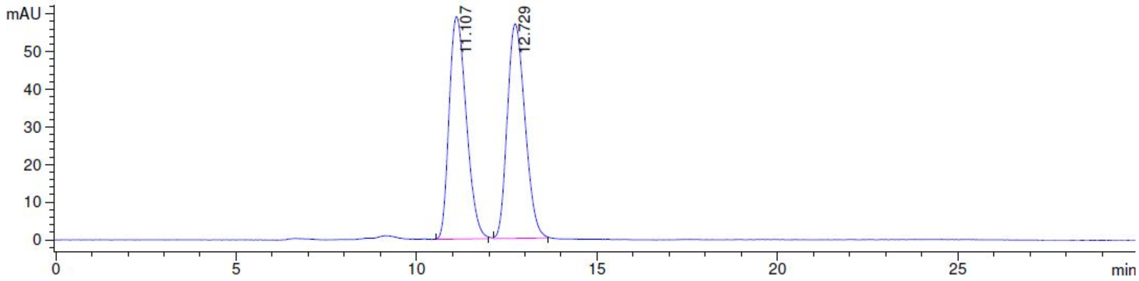

Peak #	RetTime [min]	Type	Width [min]	Area [mAU*s]	Height [mAU]	Area %
1	11.107	BB	0.5194	1961.23901	59.07012	50.1503
2	12.729	BB	0.5272	1949.48169	56.97868	49.8497

Chiral sample

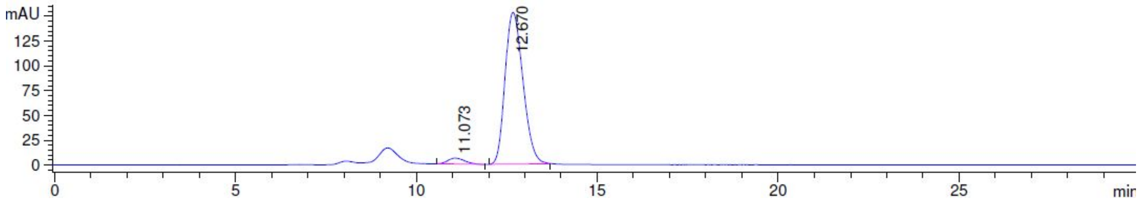

Peak #	RetTime [min]	Type	Width [min]	Area [mAU*s]	Height [mAU]	Area %
1	11.073	BP	0.4260	189.78139	5.91959	3.4764
2	12.670	BB	0.5397	5269.27588	153.03030	96.5236

Methyl (*S*,*E*)-3-(2-(2-hydroxynaphthalen-1-yl)-3-methylphenyl)acrylate (3ja)

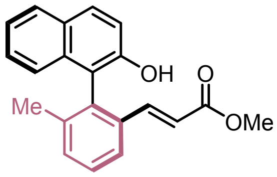

3ja

3ja was obtained by following the GP (at 60 ºC) after column chromatography (AcOEt/hexane 10:90 to 15:85) as a white solid (45%, 99:1 er). **Rf:** 0.37 (AcOEt/hexane 25:75, brown in *p*-anisaldehyde). **^{1}H NMR** (500 MHz, $CDCl_3$) δ: 7.85 (t, *J* = 8.8 Hz, 2H), 7.70 (t, *J* = 4.7 Hz, 1H), 7.45 (d, *J* = 5.1 Hz, 2H), 7.36 – 7.28 (m, 2H), 7.26 (d, *J* = 8.9 Hz, 1H), 7.20 (d, *J* = 16.0 Hz, 1H), 7.01 (dt, *J* = 8.0, 0.8 Hz, 1H), 6.31 (d, *J* = 16.0 Hz, 1H), 4.79 (s, 1H), 3.59 (s, 3H), 1.94 (s, 3H). **^{13}C NMR** (126 MHz, $CDCl_3$) δ: 167.1 (CO), 150.4 (COH), 142.6 (CH), 140.1 (C), 135.7 (C), 134.0 (C), 132.9 (C), 132.5 (CH), 130.4 (CH), 129.3 (CH), 129.3 (C), 128.5 (CH), 127.1 (CH), 124.9 (CH), 124.1 (CH), 123.7 (CH), 119.9 (CH), 117.6 (CH), 117.2 (C), 51.7 (OCH_3), 20.0 (CH_3). **HRMS** (APCI+) *m/z* calcd. for $C_{21}H_{19}O_3$ [M+H]: 319.1329; found: 319.1338.

Enantioselectivity of the product was determined by chiral HPLC analysis (Chiralpak IE3, IPA/hexane 15:85, 0.5 mL/min, λ=254 nm).

Racemic sample

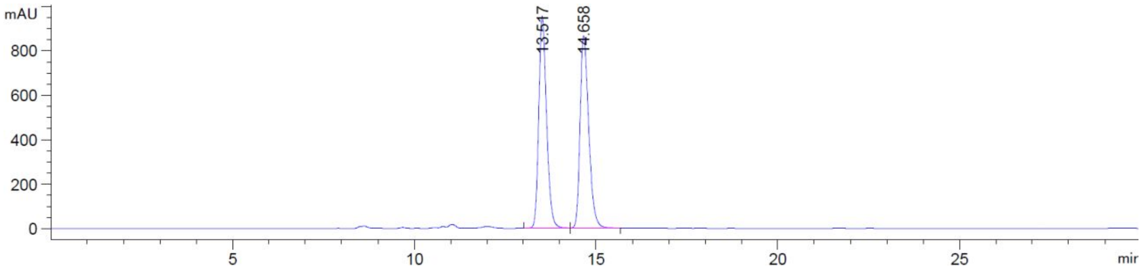

Peak #	RetTime [min]	Type	Width [min]	Area [mAU*s]	Height [mAU]	Area %
1	13.517	BB	0.2305	1.42899e4	955.53479	50.0015
2	14.658	BB	0.2525	1.42890e4	865.80457	49.9985

Chiral sample

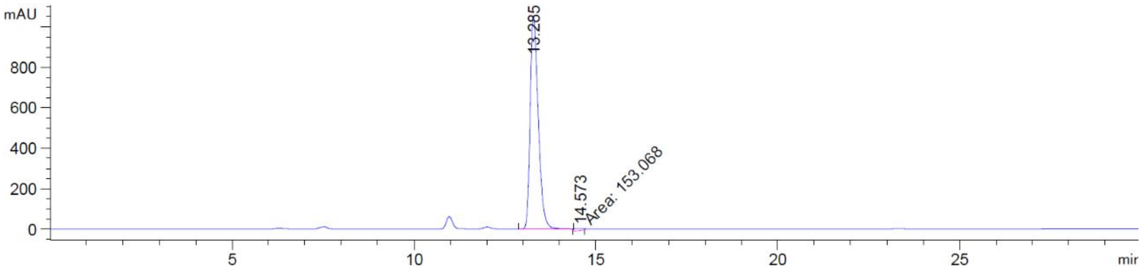

Peak #	RetTime [min]	Type	Width [min]	Area [mAU*s]	Height [mAU]	Area %
1	13.285	BB	0.2387	1.63492e4	1055.33435	99.0724
2	14.573	MM	0.2579	153.06758	8.70502	0.9276

Enantioselectivity of the remaining starting material (51%, 91.5:8.5 er) was determined by chiral HPLC analysis (Chiralpak IA3, IPA/hexane 15:85, 0.5 mL/min, λ=220 nm).

Racemic sample

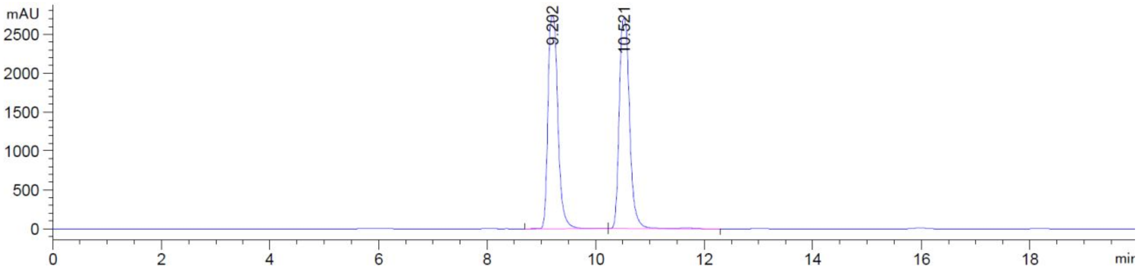

Peak #	RetTime [min]	Type	Width [min]	Area [mAU*s]	Height [mAU]	Area %
1	9.202	VB R	0.1973	3.46102e4	2739.46777	49.1314
2	10.521	BV R	0.2054	3.58339e4	2707.85254	50.8686

Chiral Sample

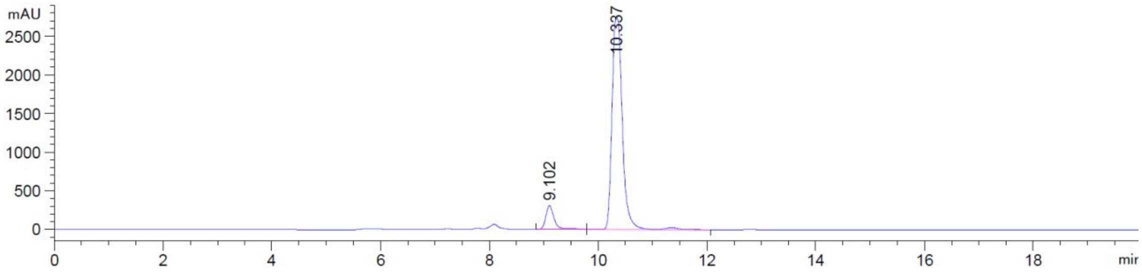

Peak #	RetTime [min]	Type	Width [min]	Area [mAU*s]	Height [mAU]	Area %
1	9.102	BV R	0.1560	3379.35498	308.94702	8.5567
2	10.337	VV R	0.2026	3.61145e4	2763.67578	91.4433

Methyl (*S*,E)-3-(5-methoxy-2-(2-hydroxynaphthalen-1-yl)-3-methylphenyl)acrylate (3ka)

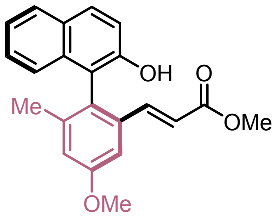

3ka

3ka was obtained by following the GP (at 60 ºC) after column chromatography (AcOEt/hexane 15:85 to 25:75) as a cream solid (46%, 92:8 er). **Rf:** 0.28 (AcOEt/hexane 25:75, light brown in *p*-anisaldehyde). **^{1}H NMR** (300 MHz, $CDCl_3$) δ: 7.89 – 7.83 (m, 2H), 7.36 – 7.31 (m, 2H), 7.28 (d, *J* = 9.0 Hz, 1H), 7.23 – 7.15 (m, 2H), 7.08 – 7.03 (m, 2H), 6.32 (d, *J* = 15.4 Hz, 1H), 4.85 (s, 1H), 3.93 (s, 3H), 3.61 (s, 3H), 1.93 (s, 3H). **^{13}C NMR** (75 MHz, $CDCl_3$) δ: 167.0 (CO), 160.1 (COMe), 150.9 (COH), 142.7 (CH), 141.7 (C), 136.9 (C), 133.5 (C), 130.3 (CH), 129.3 (C), 128.4 (CH), 127.0 (CH), 126.2 (C), 124.2 (CH), 123.6 (CH), 120.0 (CH), 118.8 (CH), 117.4 (CH), 117.0 (C), 109.4 (CH), 55.5 (OCH_3), 51.7 (OCH_3), 20.3 (CH_3). **HRMS** (APCI+) *m/z* calcd. for $C_{22}H_{21}O_4$ [M+H]: 349.1434; found: 349.1437.

Enantioselectivity of the product was determined by chiral HPLC analysis (Chiralpak IE3, IPA/hexane 15:85, 0.5 mL/min, λ=254 nm).

Racemic sample

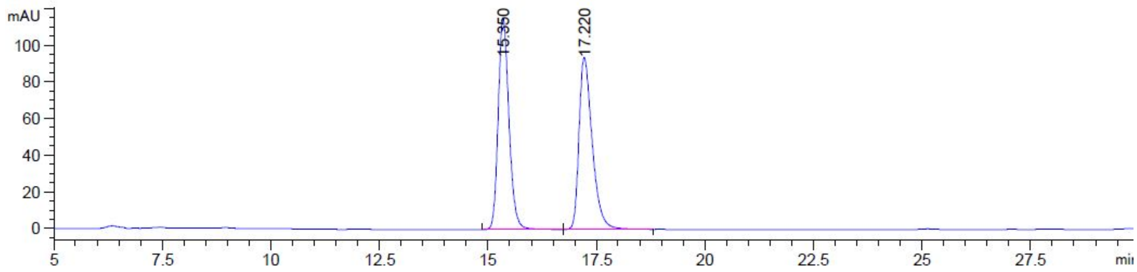

Peak #	RetTime [min]	Type	Width [min]	Area [mAU*s]	Height [mAU]	Area %
1	15.350	BB	0.2644	1981.61157	115.38930	50.0280
2	17.220	BB	0.3226	1979.39514	93.62877	49.9720

Chiral sample

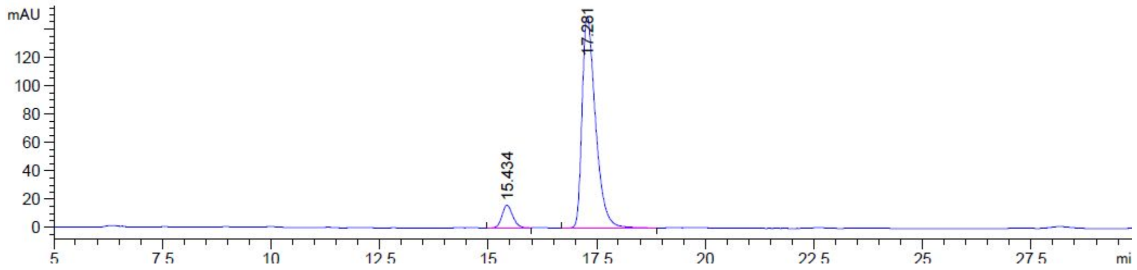

Peak #	RetTime [min]	Type	Width [min]	Area [mAU*s]	Height [mAU]	Area %
1	15.434	BB	0.2618	273.95554	16.00040	7.8789
2	17.281	BB	0.3265	3203.11304	149.19524	92.1211

Enantioselectivity of the remaining starting material (40%, 88:12 er) was determined by chiral HPLC analysis (Chiralpak IA3, IPA/hexane 15:85, 0.5 mL/min, λ=220 nm).

Racemic sample

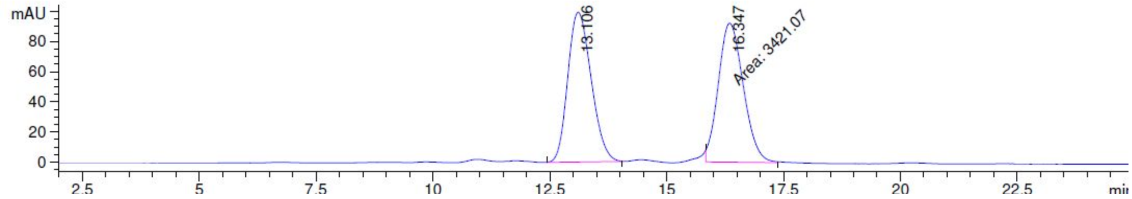

Peak #	RetTime [min]	Type	Width [min]	Area [mAU*s]	Height [mAU]	Area %
1	13.106	BB	0.5424	3448.09399	98.98386	50.1967
2	16.347	MM	0.6204	3421.07275	91.89847	49.8033

Chiral sample

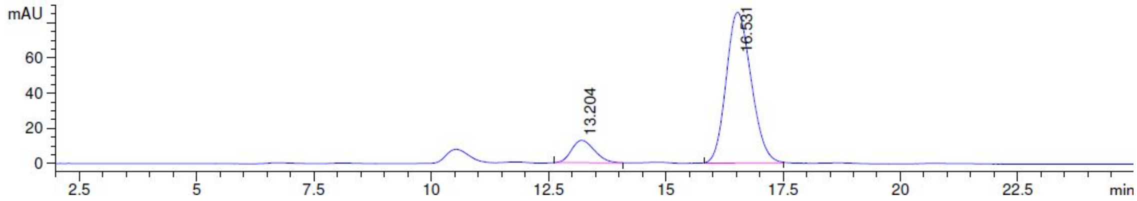

Peak #	RetTime [min]	Type	Width [min]	Area [mAU*s]	Height [mAU]	Area %
1	13.204	BB	0.5132	432.92471	12.71729	11.9798
2	16.531	BB	0.5770	3180.87451	85.71182	88.0202

Methyl (*S*,*E*)-3-(5-fluoro-2-(2-hydroxynaphthalen-1-yl)-3-methylphenyl)acrylate (3la)

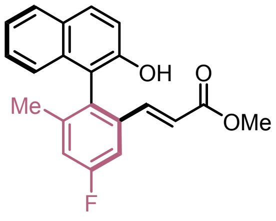

3la

3la was obtained by following the GP (at 60 ºC) after column chromatography (AcOEt/hexane 10:90 to 20:80) as a white solid (43%, 96.5:3.5 er). **Rf:** 0.35 (AcOEt/hexane 25:75, light brown in *p*-anisaldehyde). **^{1}H NMR** (300 MHz, $CDCl_3$) δ: 7.90 – 7.79 (m, 2H), 7.41 – 7.30 (m, 3H), 7.24 (d, *J* = 7.0 Hz, 1H), 7.20 – 7.09 (m, 2H), 7.02 – 6.95 (m, 1H), 6.29 (d, *J* = 15.9 Hz, 1H), 4.74 (s, 1H), 3.60 (s, 3H), 1.93 (s, 3H). **^{13}C NMR** (75 MHz, $CDCl_3$) δ: 166.7 (CO), 163.0 (d, *J* = 247 Hz, CF), 150.7 (COH), 142.9 (d, *J* = 8.0 Hz, C), 141.6 (d, *J* = 2.9 Hz, CH), 137.8 (d, *J* = 7.8 Hz, C), 133.1 (C), 130.6 (CH), 129.3 (C), 128.6 (CH), 127.3 (CH), 123.9 (d, *J* = 2.9 Hz, 2CH), 121.0 (CH), 119.4 (d, *J* = 21.0 Hz, CH), 117.6 (CH), 116.3 (C), 111.3 (d, *J* = 21.0 Hz, CH), 51.8 (OCH_3), 20.2 (CH_3). **^{19}F NMR** (282 MHz, $CDCl_3$) δ: -113.0 (t, *J* = 9.4 Hz). **HRMS** (APCI+) *m/z* calcd. for $C_{21}H_{18}FO_3$ [M+H]: 337.1234; found: 337.1234.

Enantioselectivity of the product was determined by chiral HPLC analysis (Chiralpak IB, IPA/hexane 15:85, 0.5 mL/min, λ=254 nm).

Racemic sample

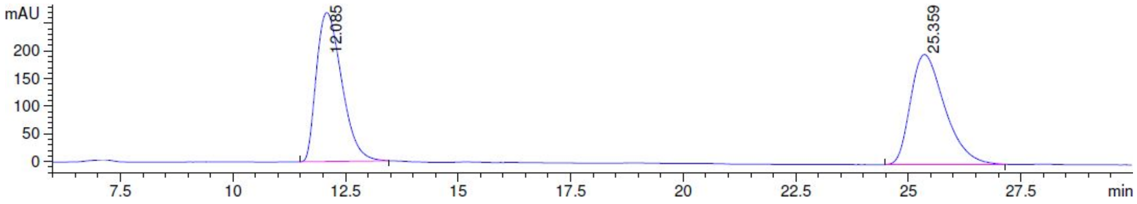

Peak #	RetTime [min]	Type	Width [min]	Area [mAU*s]	Height [mAU]	Area %
1	12.085	PB	0.6211	1.05535e4	269.33380	50.0529
2	25.359	BB	0.7957	1.05312e4	198.90410	49.9471

Chiral sample

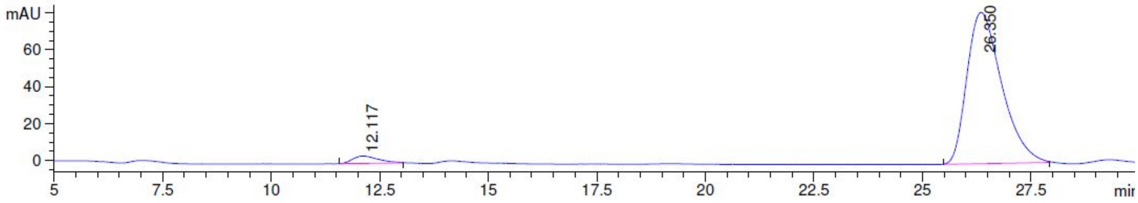

Peak #	RetTime [min]	Type	Width [min]	Area [mAU*s]	Height [mAU]	Area %
1	12.117	PB	0.5042	165.38063	3.93569	3.4930
2	26.350	BB	0.8487	4569.23730	81.92039	96.5070

Enantioselectivity of the remaining starting material (45%, 91.5:8.5 er) was determined by chiral HPLC analysis (Chiralpak IA3, IPA/hexane 15:85, 0.5 mL/min, λ=220 nm).

Racemic sample

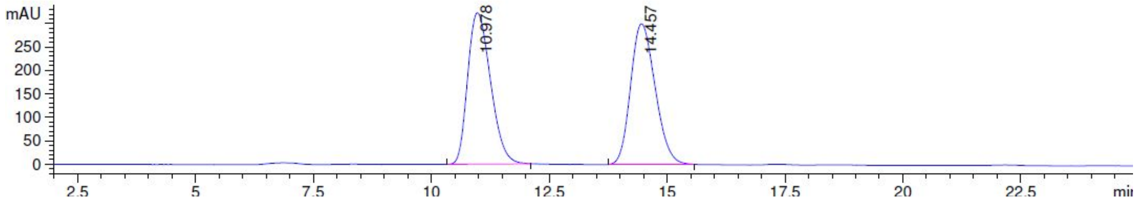

Peak #	RetTime [min]	Type	Width [min]	Area [mAU*s]	Height [mAU]	Area %
1	10.978	BB	0.5382	1.09013e4	322.64209	50.0672
2	14.457	BB	0.5726	1.08721e4	300.15512	49.9328

Chiral sample

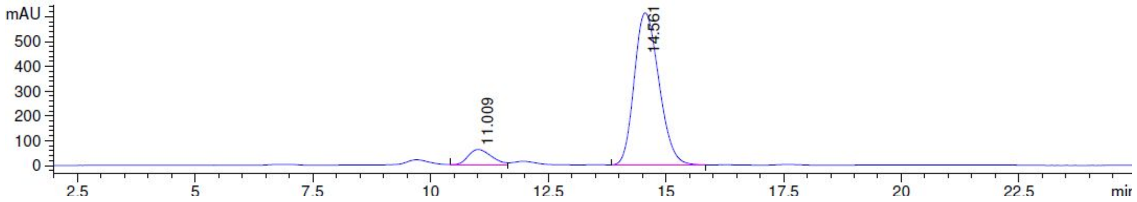

Peak #	RetTime [min]	Type	Width [min]	Area [mAU*s]	Height [mAU]	Area %
1	11.009	VV	0.5368	2108.85352	61.38950	8.6239
2	14.561	BB	0.5775	2.23447e4	612.68250	91.3761

Methyl (*R*,*E*)-3-(2-(2-hydroxynaphthalen-1-yl)-[1,1'-biphenyl]-3-yl)acrylate (3ma)

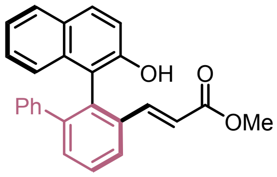

3ma

3ma was obtained by following the GP (at 60 ºC) after column chromatography (AcOEt/hexane 10:90 to 20:80) as a white solid (48%, 94:6 er). **Rf:** 0.26 (AcOEt/hexane 25:75, light orange in *p*-anisaldehyde). **^{1}H NMR** (500 MHz, $CDCl_3$) δ: 7.90 (dd, *J* = 7.6, 1.6 Hz, 1H), 7.77 – 7.73 (m, 2H), 7.68 – 7.61 (m, 2H), 7.34 – 7.27 (m, 3H), 7.14 (d, *J* = 7.3 Hz, 1H), 7.09 (d, *J* = 8.9 Hz, 1H), 7.07 – 7.01 (m, 5H), 6.42 (d, *J* = 16.0 Hz, 1H), 4.90 (s, 1H), 3.64 (s, 3H). **^{13}C NMR** (126 MHz, $CDCl_3$) δ: 167.0 (CO), 150.7 (COH), 145.1 (C), 142.7 (CH), 140.2 (C), 136.2 (C), 133.6 (C), 133.2 (C), 132.6 (CH), 130.4 (CH), 129.4 (CH), 128.8 (C), 128.4 (2CH), 128.3 (CH), 127.6 (2CH), 127.2 (CH), 126.9 (CH), 126.3 (CH), 124.6 (CH), 123.5 (CH), 120.3 (CH), 117.4 (C), 117.2 (CH), 51.7 (OCH_3). **HRMS** (APCI+) *m/z* calcd. for $C_{26}H_{20}O_3$ [M+H]: 381.1485; found: 381.1481.

Enantioselectivity of the product was determined by chiral HPLC analysis (Chiralpak IE3, IPA/hexane 15:85, flow rate = 0.5 mL/min, λ=220 nm).

Racemic Sample

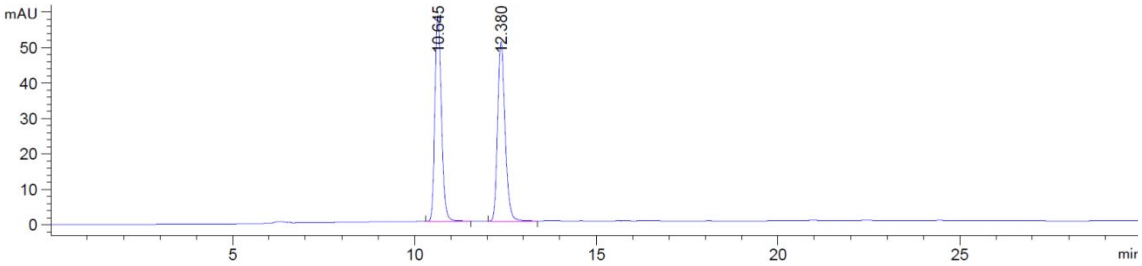

Peak #	RetTime [min]	Type	Width [min]	Area [mAU*s]	Height [mAU]	Area %
1	10.645	BB	0.1911	722.30890	58.09529	50.0234
2	12.380	BB	0.2186	721.63422	50.53850	49.9766

Chiral Sample

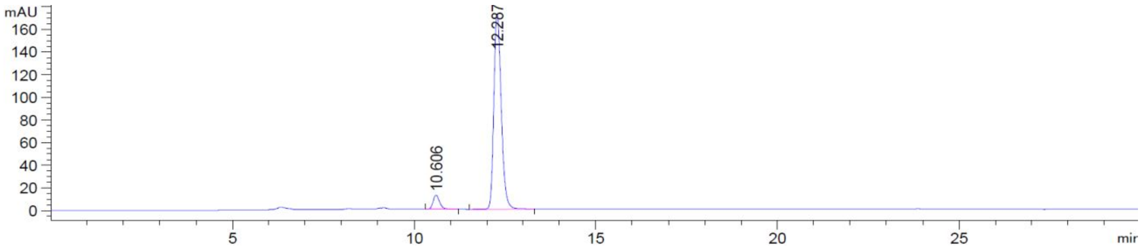

Peak #	RetTime [min]	Type	Width [min]	Area [mAU*s]	Height [mAU]	Area %
1	10.606	BB	0.1925	157.41000	12.53261	6.0569
2	12.287	BB	0.2167	2441.44604	172.96353	93.9431

Enantioselectivity of the remaining starting material (51%, 97:3 er) was determined by chiral HPLC analysis (Chiralpak IA3, IPA/hexane 15:85, 0.5 mL/min, λ=300 nm).

Racemic Sample

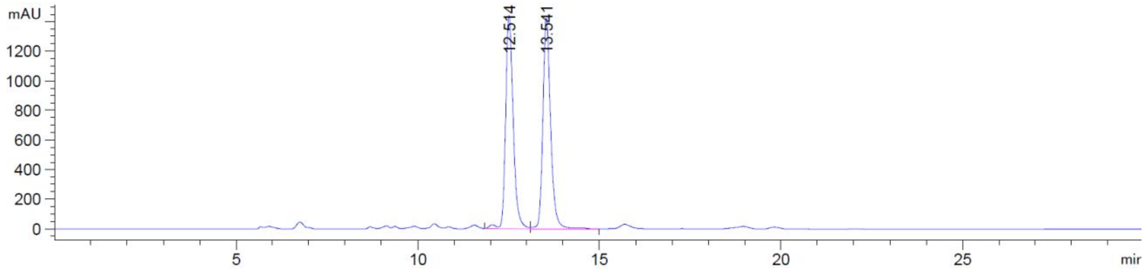

Peak #	RetTime [min]	Type	Width [min]	Area [mAU*s]	Height [mAU]	Area %
1	12.514	VV R	0.2208	2.09352e4	1442.16321	49.7297
2	13.541	VV R	0.2250	2.11628e4	1424.97925	50.2703

Chiral Sample

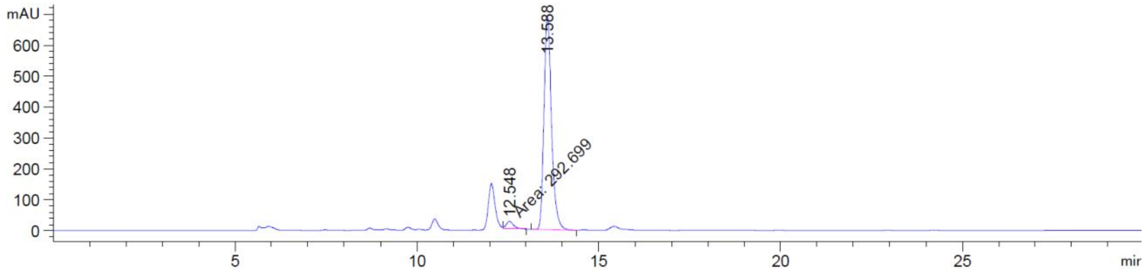

Peak #	RetTime [min]	Type	Width [min]	Area [mAU*s]	Height [mAU]	Area %
1	12.548	FM	0.2149	292.69907	22.69707	2.8526
2	13.588	BB	0.2198	9968.07813	692.97369	97.1474

4.3. Gram scale kinetic resolution of *rac*-1j

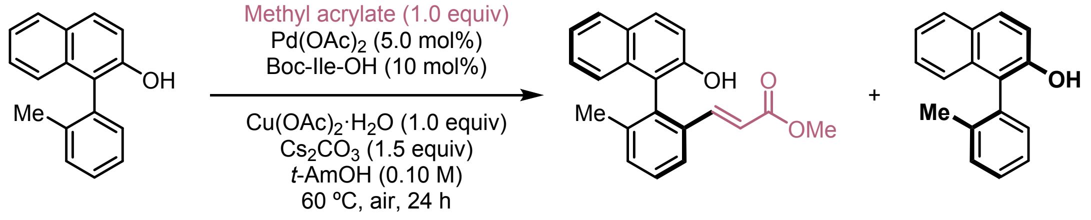

rac-**1j** 1.87 g (8.00 mmol) — **3ja** 1.19 g (47%) 99.9:0.1 er — **1j** 0.748 g (40%) 96.5:3.5 er

rac-**1j** (1.87 g, 8.00 mmol, 1.00 equiv), $Pd(OAc)_2$ (90 mg, 5.0 mol%), Boc-Ile-OH (185 mg, 10 mol%), $Cu(OAc)_2 \cdot H_2O$ (1.60 g, 8.00 mmol, 1.00 equiv) and Cs_2CO_3 (3.91 g, 12.0 mmol, 1.50 equiv) were weighed and added into a 250 mL one-necked RBF flask under air. Then, *t*-AmOH (80 mL, 0.10 M) and methyl acrylate (725 μL, 8.00 mmol, 1.00 equiv) were added. The flask was sealed with a rubber septum and the mixture was stirred under air at 60 ºC for 24 h. After cooling to rt, the reaction mixture was filtered through a Celite pad, washing the flask and the pad with AcOEt. The filtrate was concentrated under reduced pressure and the resulting residue was purified by flash column chromatography (AcOEt/hexane 5:95 to 20:80) to afford 0.748 g (40%, 96.4:3.6 er) of **1j** and 1.19 g (47%, 99.9:0.1 er) of **3ja**.

Enantioselectivity of the product was determined by chiral HPLC analysis (Chiralpak IE3, IPA/hexane 15:85, 0.5 mL/min, λ=254 nm).

Racemic sample

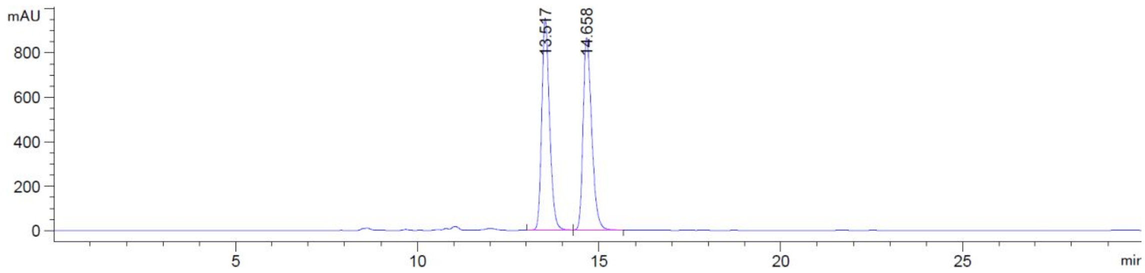

Peak #	RetTime [min]	Type	Width [min]	Area [mAU*s]	Height [mAU]	Area %
1	13.517	BB	0.2305	1.42899e4	955.53479	50.0015
2	14.658	BB	0.2525	1.42890e4	865.80457	49.9985

Chiral sample

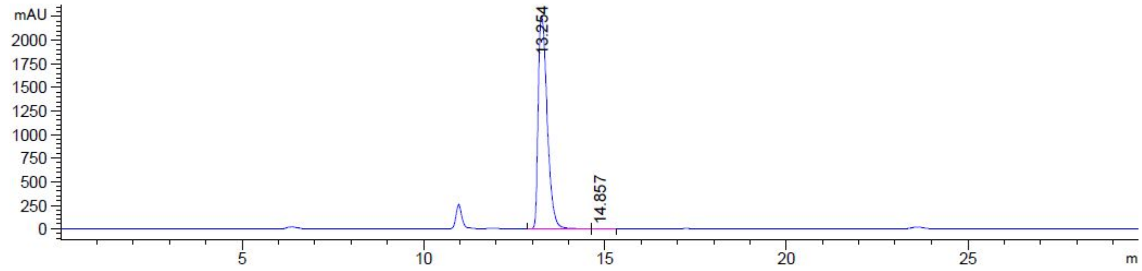

Peak #	RetTime [min]	Type	Width [min]	Area [mAU*s]	Height [mAU]	Area %
1	13.254	BB	0.2680	3.87410e4	2259.54883	99.9278
2	14.857	BB	0.2560	27.98061	1.68289	0.0722

Enantioselectivity of the remaining starting material was determined by chiral HPLC analysis (Chiralpak IA3, IPA/hexane 15:85, 0.5 mL/min, λ=220 nm).

Racemic sample

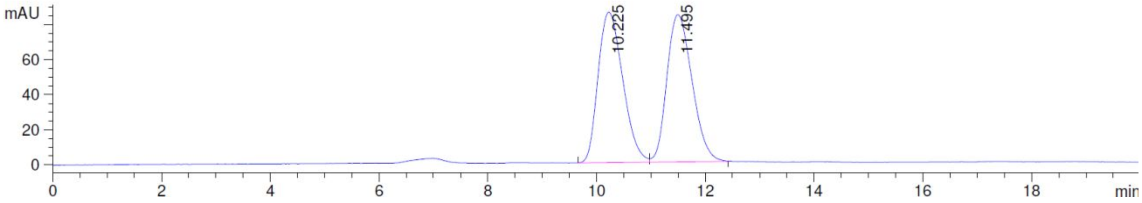

Peak #	RetTime [min]	Type	Width [min]	Area [mAU*s]	Height [mAU]	Area %
1	10.225	BV	0.4943	2706.71094	85.73557	50.0038
2	11.495	VB	0.5078	2706.30054	84.01353	49.9962

Chiral Sample

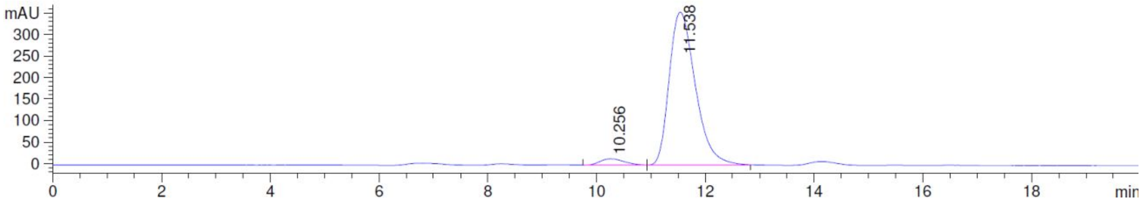

Peak #	RetTime [min]	Type	Width [min]	Area [mAU*s]	Height [mAU]	Area %
1	10.256	PV	0.4308	442.24673	14.54315	3.5953
2	11.538	VB	0.5194	1.18585e4	355.32376	96.4047

4.4. Mechanistic experiments

4.4.1. Synthesis of the deuterated starting material (d_7-1a)

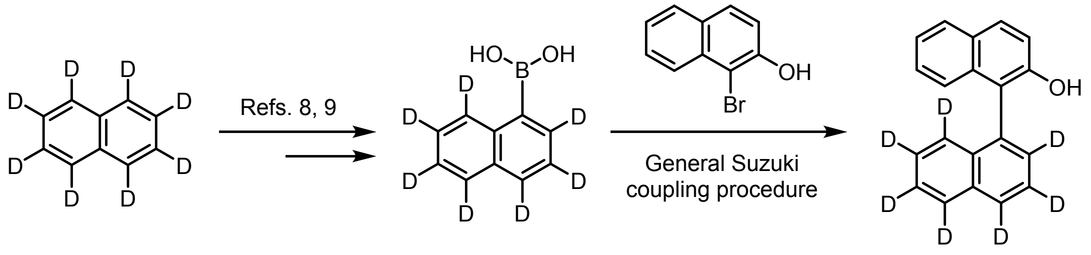

S11 S12 d_7-**1a**

Compound **S12** was synthesized from naphthalene-d_8 **(S11)** following reported procedures[8,9]. **S12** was converted to d_7-**1a** by the general Suzuki coupling procedure for the substrate synthesis. Its ^{1}H NMR spectrum is shown bellow.

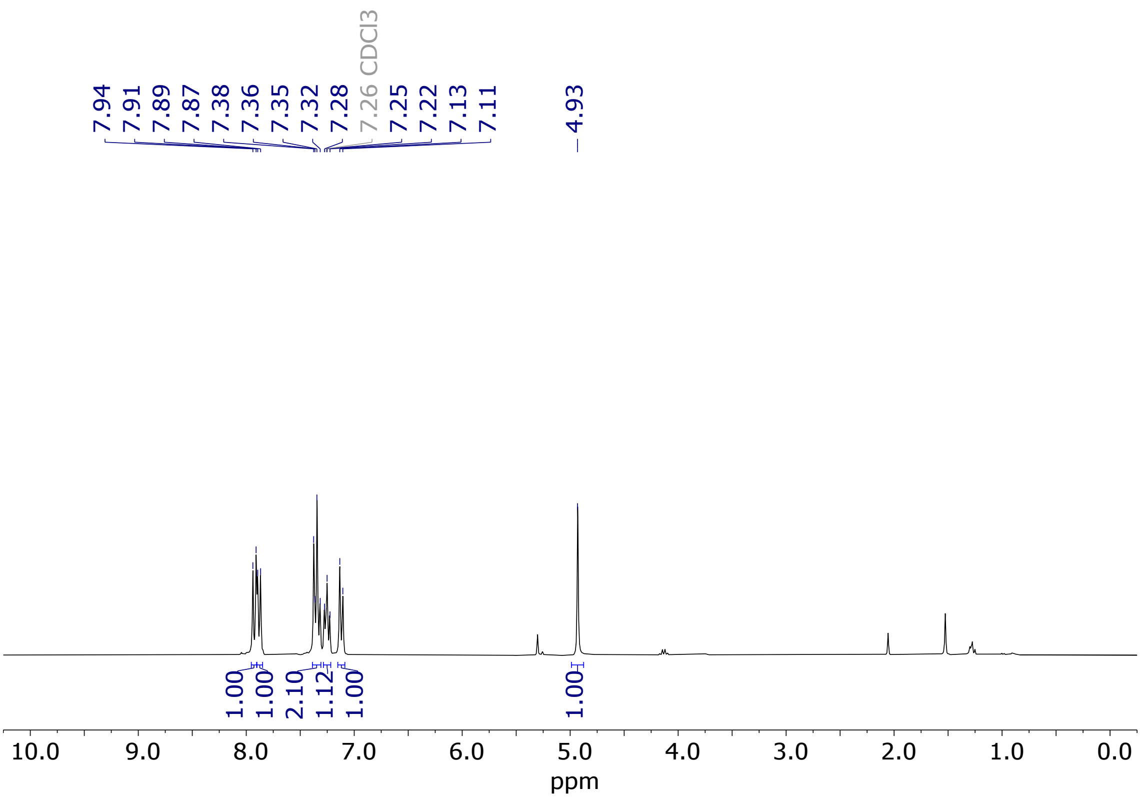

[8] Wang, Q.; Zhang, W.; Song, H.; Wang, J.; Zheng, C.; Gu, Q.; You, S. *J. Am. Chem. Soc.* **2020**, *142*, 15678-15685.
[9] Bonvallet, P. A.; Breitkreuz, C. J.; Kim, Y. S.; Todd, E. M.; Traynor, K.; Fry, C. G.; Ediger, M. D.; McMahon, R. *J. Org. Chem.* **2007**, *72*, 10051-10057.

4.4.2. Measurement of the Kinetic Isotope Effect (KIE) by parallel test method

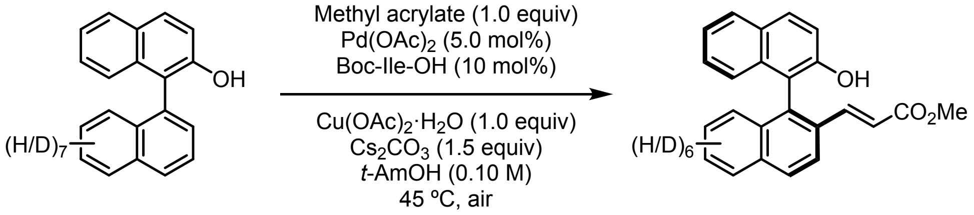

rac-**1a** or *d*$_7$-**1a** 3aa or *d*$_6$-**3aa**

Following the GP of the kinetic resolution, two reactions were set using two separated Schlenk tubes. *rac*-**1a** (0.20 mmol, 1.0 equiv) was added to one of the flasks and *d*$_7$-**1a** (0.20 mmol, 1.0 equiv) to the other. $Pd(OAc)_2$ (2.2 mg, 5.0 mol%), Boc-Ile-OH (4.6 mg, 10 mol%), $Cu(OAc)_2·H_2O$ (40 mg, 1.0 equiv) and Cs_2CO_3 (98 mg, 1.5 equiv) were added into each Schlenk flask under air. Then, *t*-AmOH (2.0 mL, 0.10 M) and methyl acrylate (0.20 mmol, 1.0 equiv) were added. The flasks were sealed with a rubber septum and heated to 45 ºC at the same time in the same heating block. Aliquots of 250 µL of each reaction were taken at 10, 20, 45, and 90 min; filtered through a Florisil© pad and eluted with AcOEt. The volatiles were removed under reduced pressure and the crude residues were analysed with ^{1}H NMR spectrometry.

To calculate KIE, the linear regression method was used. The conversion (quotient between the integral of **3aa** signal and the summatory of the integral of **1a** and **3aa**) was plotted as a function of time, so that the slope can be calculated with a least squares approximation. KIE was calculated as the quotient between the slope of the non-deuterated experiment and the deuterated experiment, resulting in a value of **2.5**.

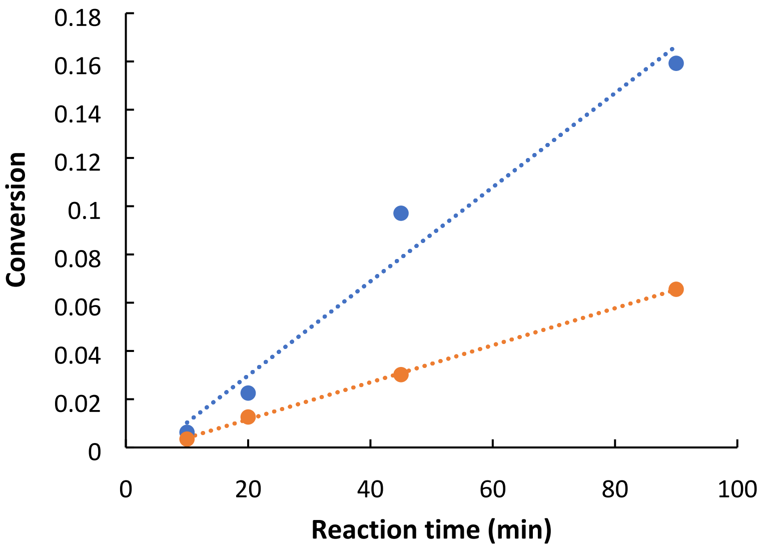

Non-Deuterated Sample	
**Time (min)**	**Conv.**
10	0,0063
20	0,0225
45	0,0970
90	0,1590

Deuterated Sample	
**Time (min)**	**Conv.**
10	0,0035
20	0,0126
45	0,0302
90	0,0656

5. Other reactions

5.1. Attempt of desymmetrization of 1-phenyl-2-naphthol (1n)

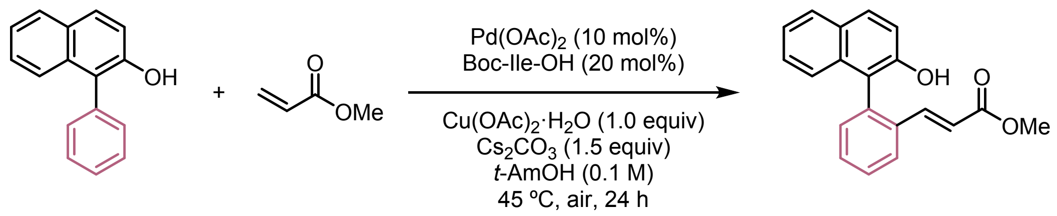

1n (22 mg, 0.10 mmol, 1.0 equiv), $Pd(OAc)_2$ (2.2 mg, 10 mol%), Boc-Ile-OH (4.6 mg, 20 mol%), $Cu(OAc)_2{\cdot}H_2O$ (20 mg, 1.0 equiv) and Cs_2CO_3 (49 mg, 1.5 equiv) were weighed and added into a Schlenk flask under air. Then, *t*-AmOH (1.0 mL, 0.10 M) and methyl acrylate (0.10 mmol, 1.0 equiv) were added. The flask was sealed with a rubber septum and the mixture was stirred under air at 45 ºC for 24 h. After cooling to rt, the reaction mixture was diluted with AcOEt and filtered through a Celite pad, washing the flask and the pad with more AcOEt (x3). The filtrate was concentrated under reduced pressure (50 ºC). The resulting residue was purified by flash column chromatography (AcOEt/hexane 15:85 to 20:80) to afford 21.2 mg (70%) of **3na** as a light-yellow solid. **Rf:** 0.35 (AcOEt/hexane 25:75, garnet in *p*-anisaldehyde). **^{1}H NMR** (500 MHz, $CDCl_3$) δ: 7.86 – 7.79 (m, 3H), 7.55 – 7.50 (m, 2H), 7.34 – 7.26 (m, 4H), 7.22 (d, *J* = 8.8 Hz, 1H), 7.12 – 7.09 (m, 1H), 6.34 (d, *J* = 16.0 Hz, 1H), 4.92 (s, 1H), 3.58 (s, 3H). **^{13}C NMR** (126 MHz, $CDCl_3$) δ: 167.1 (CO), 150.6 (COH), 142.1 (CH), 135.4 (C), 135.1 (C), 133.5 (C), 132.8 (CH), 131.1 (CH), 130.4 (CH), 129.4 (CH), 129.1 (C), 128.3 (CH), 127.4 (CH), 126.9 (CH), 124.6 (CH), 123.7 (CH), 119.9 (CH), 118.5 (C), 117.6 (CH), 51.7 (OCH_3). **HRMS** (APCI+) *m/z* calcd. for $C_{20}H_{17}O_3$ [M+H]: 305.1172; found: 305.1173.

Enantioselectivity of the product was determined by chiral HPLC analysis (Chiralpak IB, IPA/hexane 20:80, 1.0 mL/min, λ=220 nm).

Sample with racemic ligand

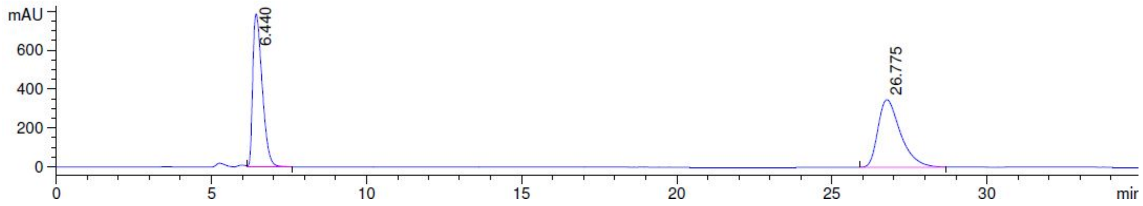

Peak #	RetTime [min]	Type	Width [min]	Area [mAU*s]	Height [mAU]	Area %
1	6.440	VB	0.3319	1.67008e4	785.94489	48.7943
2	26.775	BB	0.7694	1.75262e4	346.94434	51.2057

Sample with chiral ligand

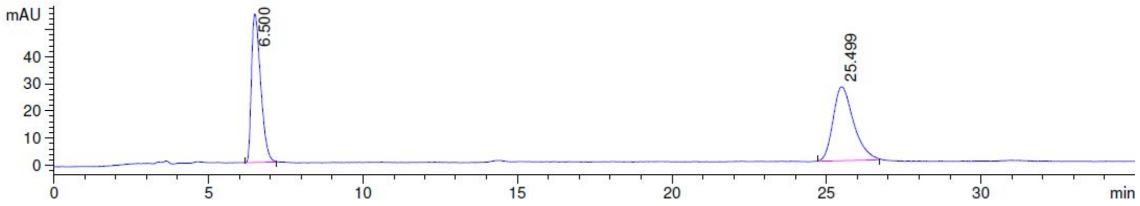

Peak #	RetTime [min]	Type	Width [min]	Area [mAU*s]	Height [mAU]	Area %
1	6.500	PB	0.3187	1138.06421	54.70285	47.4357
2	25.499	BB	0.6876	1261.10645	27.26560	52.5643

5.2. Attempt of atroposelective alkenylation of 2'-methyl-[1,1'-biphenyl]-2-ol (1o)

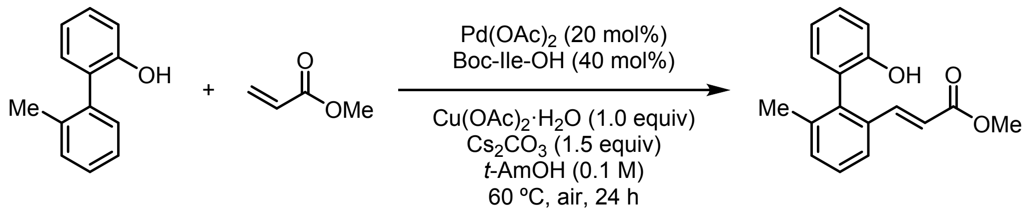

1o **2a**, 5.0 equiv **3oa**

1o (18.4 mg, 0.10 mmol, 1.0 equiv), $Pd(OAc)_2$ (4.5 mg, 20 mol%), Boc-Ile-OH (9.3 mg, 40 mol%), $Cu(OAc)_2 \cdot H_2O$ (20 mg, 1.0 equiv) and Cs_2CO_3 (49 mg, 1.5 equiv) were weighed and added into a Schlenk flask under air. Then, *t*-AmOH (1.0 mL, 0.10 M) and methyl acrylate (0.50 mmol, 5.0 equiv) were added. The flask was sealed with a rubber septum and the mixture was stirred under air at 60 ºC for 24 h. After cooling to rt, the reaction mixture was diluted with AcOEt and filtered through a Celite pad, washing the flask and the pad with more AcOEt (x3). The filtrate was concentrated under reduced pressure (50 ºC). The resulting residue was purified by flash column chromatography (AcOEt/hexane 15:85 to 20:80) to afford 24.3 mg (91%) of **3oa** as a light-yellow solid. **Rf:** 0.35 (AcOEt/hexane 25:75, pink in *p*-anisaldehyde). **^{1}H NMR** (500 MHz, $CDCl_3$) δ: 7.61 – 7.58 (m, 1H), 7.40 (d, *J* = 16.0 Hz, 1H), 7.39 – 7.33 (m, 2H), 7.32 (ddd, *J* = 8.1, 6.9, 2.1 Hz, 1H), 7.05 – 6.96 (m, 3H), 6.30 (d, *J* = 16.0 Hz, 1H), 4.61 (s, 1H), 3.69 (s, 3H), 2.09 (s, 3H).**^{13}C NMR** (126 MHz, $CDCl_3$) δ: 167.3 (COH), 152.6 (C), 143.1 (CH), 138.9 (C), 136.6 (C), 134.7 (C), 132.2 (CH), 130.6 (CH), 129.9 (CH), 128.8 (CH), 124.7 (C), 124.6 (CH), 121.1 (CH), 119.5 (CH), 115.9 (CH), 51.8 (OCH_3), 20.4 (CH_3). **HRMS** (APCI+) *m/z* calcd. for $C_{17}H_{17}O_3$ [M+H]: 269.1172 found: 269.1170.

Enantioselectivity of the product was determined by chiral HPLC analysis (Chiralpak IE3, IPA/hexane 20:80, 0.5 mL/min, λ=220 nm).

Sample with racemic ligand

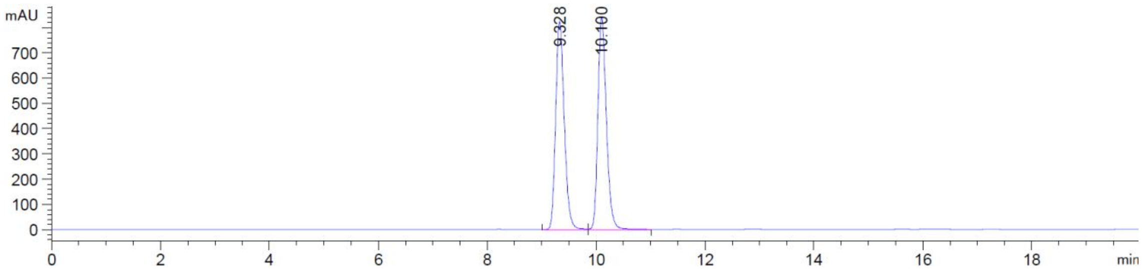

Peak #	RetTime [min]	Type	Width [min]	Area [mAU*s]	Height [mAU]	Area %
1	9.328	BV	0.1677	9024.62891	824.35577	49.8941
2	10.100	VB	0.1655	9062.93555	842.25098	50.1059

Sample with chiral ligand

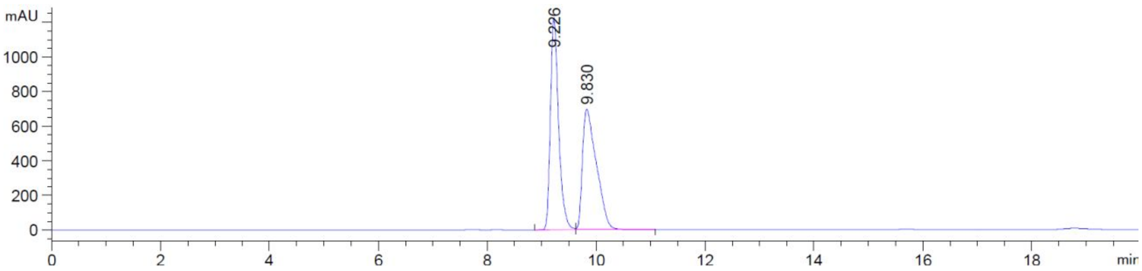

Peak #	RetTime [min]	Type	Width [min]	Area [mAU*s]	Height [mAU]	Area %
1	9.226	BV	0.1580	1.27960e4	1223.87549	50.8122
2	9.830	VB	0.2576	1.23869e4	695.99994	49.1878

5.3. Dynamic kinetic resolution of 1-(2-methoxyphenyl)naphthalen-2-ol (*rac*-1p) through Pd(II)-catalyzed C-H alkenylation

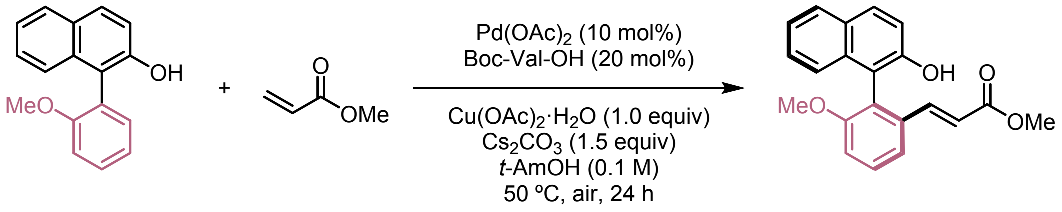

***rac*-1p** **2a**, 2.0 equiv **3pa**

rac-**1p** (25 mg, 0.10 mmol, 1.0 equiv), $Pd(OAc)_2$ (1.1 mg, 5 mol%), Boc-Val-OH (2.3 mg, 10 mol%), $Cu(OAc)_2{\cdot}H_2O$ (20 mg, 1.0 equiv) and Cs_2CO_3 (49 mg, 1.5 equiv) were weighed and added into a Schlenk flask under air. Then, *t*-AmOH (1.0 mL, 0.10 M) and methyl acrylate (0.20 mmol, 2.0 equiv) were added. The flask was sealed with a rubber septum and the mixture was stirred under air at 50 °C for 24 h. After cooling to rt, the reaction mixture was diluted with AcOEt and filtered through a Celite pad, washing the flask and the pad with more AcOEt (x3). The filtrate was concentrated under reduced pressure (50 °C). The resulting residue was purified by flash column chromatography (AcOEt/hexane 25:75) to afford 20 mg (60%, 94.5:5.5 er) of **3pa** as a white solid. **Rf:** 0.20 (AcOEt/hexane 25:75, blue in *p*-anisaldehyde). **^{1}H NMR** (500 MHz, $CDCl_3$) δ: 7.85 (d, *J* = 8.9 Hz, 1H), 7.82 (dd, *J* = 7.1, 2.4 Hz, 1H), 7.53 (t, *J* = 8 Hz, 1H), 7.47 (d, *J* = 7.8 Hz, 1H), 7.30 (dtd, *J* = 8.1, 6.0, 1.7 Hz, 2H), 7.26 (d, *J* = 9.0 Hz, 1H), 7.17 (d, *J* = 15.9 Hz, 1H), 7.12 (d, *J* = 8.2 Hz, 1H), 7.09 – 7.05 (m, 1H), 6.33 (d, *J* = 16.0 Hz, 1H), 4.85 (s, 1H), 3.67 (s, 3H), 3.59 (s, 3H). **^{13}C NMR** (126 MHz, $CDCl_3$) δ: 167.0 (CO), 158.8 (COH), 150.9 (C), 142.2 (CH), 137.0 (C), 133.6 (C), 130.4 (CH), 130.4 (CH), 129.2 (C), 128.3 (CH), 126.7 (CH). 124.4 (CH), 123.5 (CH), 123.3 (C), 120.4 (CH), 119.4 (CH), 117.6 (CH), 114.6 (C), 112.9 (CH), 56.2 (OCH_3), 51.7 (OCH_3). **HRMS** (APCI+) *m/z* calcd. for $C_{21}H_{18}O_4$ [M+H]: 335.1278; found: 335.1281.

Enantioselectivity of the product was determined by chiral HPLC analysis (Chiralpak IE3, IPA/hexane 20:80, 0.5 mL/min, λ=300 nm).

Racemic Sample

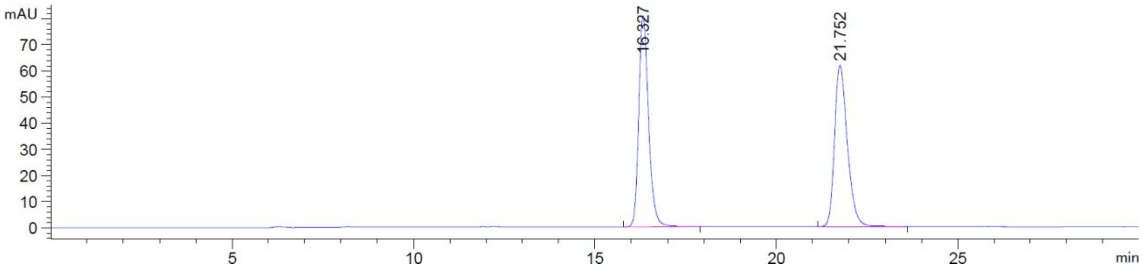

Peak #	RetTime [min]	Type	Width [min]	Area [mAU*s]	Height [mAU]	Area %
1	16.327	BB	0.2861	1508.23328	80.67456	49.9048
2	21.752	BB	0.3747	1513.98755	61.61275	50.0952

Chiral sample

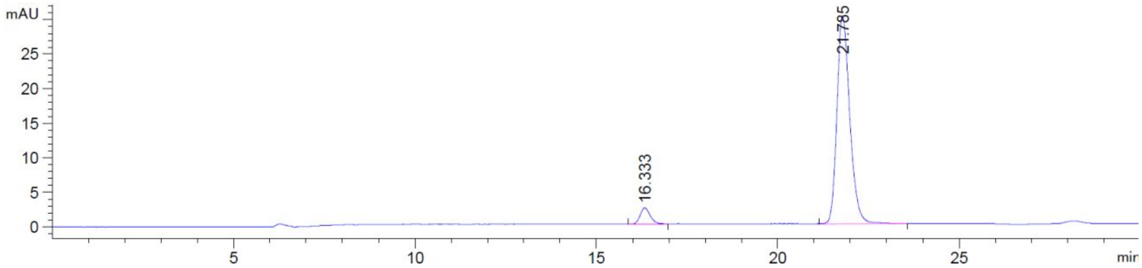

Peak #	RetTime [min]	Type	Width [min]	Area [mAU*s]	Height [mAU]	Area %
1	16.333	BB	0.2879	44.75937	2.35355	5.6968
2	21.785	BB	0.3770	740.93549	30.12297	94.3032

6. Synthetic applications of the enantioenriched biaryls

6.1. Triflation

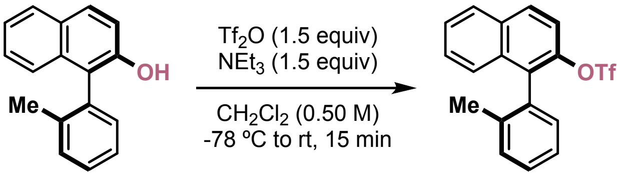

1j 4

To a solution of **1j** (0.234 g, 1.00 mmol, 1.00 equiv, 96.4:3.6 er) and NEt_3 (0.21 mL, 1.50 mmol, 1.50 equiv) in CH_2Cl_2 (2.0 mL) at -78 ºC, Tf_2O (0.25 mL, 1.50 mmol, 1.50 equiv) was dropwise added under argon and the resulting garnet solution was warmed to rt and stirred for 15 min. The reaction was quenched with 1.0 M HCl, and the mixture was partitioned between CH_2Cl_2 and water. The layers were separated, and the aqueous phase was extracted with CH_2Cl_2 (x2). The combined organic phase was dried over Na_2SO_4 and concentrated under reduced pressure. The resulting residue was purified by flash column chromatography (AcOEt/hexane 2:98) to afford 0.320 g (87%) of **4** as a colorless oil. **Rf:** 0.65 (AcOEt/hexane 10:90, colorless in *p*-anisaldehyde). **^{1}H NMR** (300 MHz, $CDCl_3$) δ: 7.96 (dd, *J* = 8.6, 5.2 Hz, 2H), 7.57 (ddd, *J* = 8.1, 5.3, 2.9 Hz, 1H), 7.52 – 7.45 (m, 3H), 7.44 – 7.30 (m, 3H), 7.25 (d, *J* = 6.2 Hz, 1H), 2.01 (s, 3H). **^{13}C NMR (**75 MHz, $CDCl_3$) δ: 144.3 (C), 137.6 (C), 133.3 (C), 132.7 (C), 132.6 (C), 132.2 (C), 131.1 (CH), 130.4 (CH), 130.1 (CH), 129.0 (CH), 128.3 (CH), 127.7 (CH), 127.1 (CH), 126.8 (CH), 125.9 (CH), 119.6 (CH), 118.5 (d, *J* = 319 Hz, CF_3), 19.8 (CH_3). **^{19}F NMR** (282 MHz, $CDCl_3$) δ: -74.5. **HRMS** (APCI+) *m/z* calcd. for $C_{18}H_{14}F_3O_3S$ [M+H]: 366.0532; found: 366.0525.

6.2. C-P cross coupling

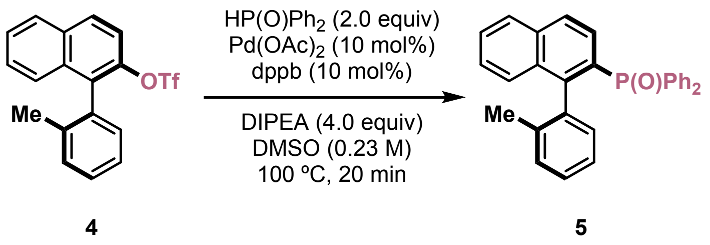

Following a literature procedure[10], to a solution of **4** (110 mg, 0.30 mmol, 1.0 equiv), $HP(O)Ph_2$ (121 mg, 0.60 mmol, 2.0 equiv), $Pd(OAc)_2$ (6.7 mg, 10 mol%) and dppb (12.8 mg, 10 mol%) in DMSO (1.3 mL) was added DIPEA (0.21 mL, 1.2 mmol, 4.0 equiv) and the mixture was stirred at 100 ºC under argon for 20 min. After cooling to rt, the reaction mixture was diluted with a high excess of water and extracted with AcOEt (x3). The combined organic phase was washed with water, dried over Na_2SO_4, and concentrated under reduced pressure. The resulting residue was purified by flash column chromatography (AcOEt/hexane 60:40) to afford 98.4 mg (78%, 96.5:3.5 er) of **5** as cream foamy solid. **Rf:** 0.30 (AcOEt/hexane 1:1, white in *p*-anisaldehyde). **^{1}H NMR** (500 MHz, $CDCl_3$) δ: 7.92 – 7.85 (m, 2H), 7.73 (dd, *J* = 11.7, 8.6 Hz, 1H), 7.63 – 7.53 (m, 3H), 7.53 – 7.48 (m, 2H), 7.46 – 7.40 (m, 2H), 7.38 – 7.33 (m, 3H), 7.30 (td, *J* = 7.7, 3.1 Hz, 2H), 7.23 (d, *J* = 8.6 Hz, 1H), 7.12 (td, *J* = 7.5, 1.5 Hz, 1H), 6.95 – 6.88 (m, 2H), 6.84 (dd, *J* = 7.5, 1.5 Hz, 1H), 1.66 (s, 3H). **^{13}C NMR** (126 MHz, $CDCl_3$) δ: 146.0 (d, *J* = 9.0 Hz, C), 138.0 (C), 136.5 (d, *J* = 4.9 Hz, C), 134.9 (d, *J* = 2.3 Hz, C), 134.0 (d, *J* = 25.2 Hz, C), 133.1 (d, *J* = 25.5, C), 132.9 (d, *J* = 11.7 Hz, C), 132.0 (d, *J* = 9.4 Hz, 2CH), 131.8 (d, *J* = 9.6 Hz, 2CH), 131.4 (d, *J* = 2.8 Hz, CH), 131.2 (CH), 131.2 (2CH), 129.5 (CH), 129.0 (C), 128.7 (d, *J* = 12.1 Hz, CH), 128.3 (d, *J* = 11.9 Hz, 2CH), 128.2 (d, *J* = 12.3 Hz, 2CH), 128.1 (d, *J* = 14.5 Hz, 2CH), 127.3 (d, *J* = 12.3 Hz, CH), 127.0 (d, *J* = 4.9 Hz, 2CH), 124.7 (CH), 20.4 (CH_3). **^{31}P NMR** (202 MHz, $CDCl_3$) δ: 27.4. **HRMS** (APCI+) *m/z* calcd. for $C_{29}H_{24}OP$ [M+H]: 419.1559; found: 419.1562.

[10] Uozumi, Y.; Suzuki, N.; Ogiwara, A.; Hayashi, T. *Tetrahedron* **1994***, 50*, 4293-4302.

Enantioselectivity of the product was determined by chiral HPLC analysis (Chiralpak IG3, IPA/hexane 20:80, 0.5 mL/min, λ=220 nm).

Racemic sample

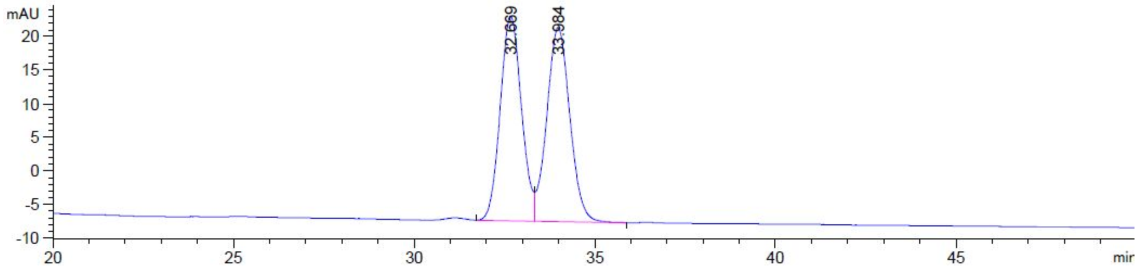

Peak #	RetTime [min]	Type	Width [min]	Area [mAU*s]	Height [mAU]	Area %
1	32.669	BV	0.6323	1244.96399	30.49198	49.2487
2	33.984	VB	0.6878	1282.94836	28.90899	50.7513

Chiral sample

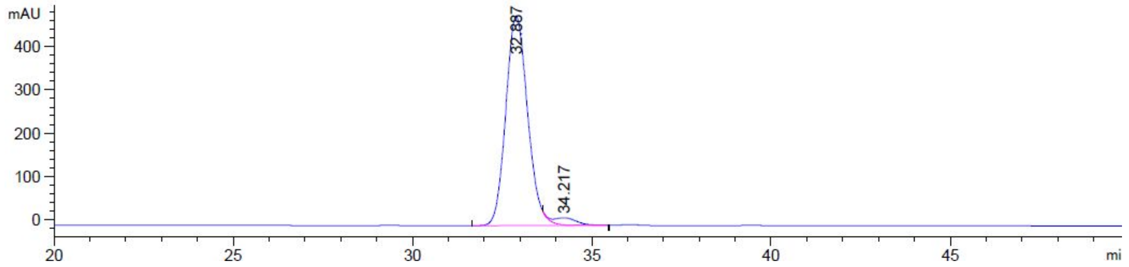

Peak #	RetTime [min]	Type	Width [min]	Area [mAU*s]	Height [mAU]	Area %
1	32.887	BV R	0.6591	2.05135e4	483.41119	96.7231
2	34.217	VB E	0.6374	694.98767	16.17125	3.2769

6.3. Phosphine oxide reduction

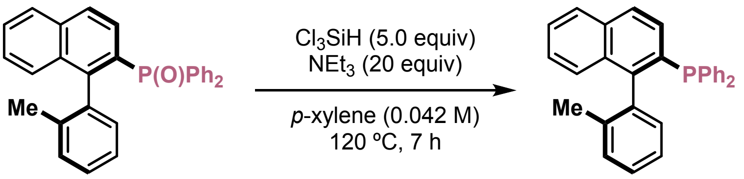

5 **6**

Following a literature procedure[11], to a solution of **5** (130 mg, 0.331 mmol, 1.0 equiv, 96.5:3.5 er) and NEt_3 (0.86 mL, 6.21 mmol, 20 equiv) in *p*-xylene (7.8 mL) under N_2 atmosphere, Cl_3SiH (157 µL, 1.55 mmol, 5.0 equiv) was added dropwise and the mixture was stirred at 120 °C for 7 h. After cooling to rt, the reaction was quenched with sat. aq. $NaHCO_3$. The mixture was filtered through Celite, and the solids were washed with Et_2O. The filtrate was dried over $MgSO_4$ and concentrated under reduced pressure. The resulting residue was purified by flash column chromatography (AcOEt/hexane 5:95) to afford 93.7 mg (75%, 97.5:2.5 er) of **6** as a white solid. **Rf:** 0.77 (AcOEt/hexane 25:75, blue in ceric ammonium molybdate). **^{1}H NMR** (500 MHz, $CDCl_3$) δ: 7.81 (d, *J* = 8.2 Hz, 1H), 7.73 (d, *J* = 8.5 Hz, 1H), 7.44 (ddd, *J* = 8.1, 6.6, 1.5 Hz, 1H), 7.33 – 7.14 (m, 15H), 7.06 (td, *J* = 6.9, 2.5 Hz, 1H), 6.82 (d, *J* = 7.1 Hz, 1H), 1.88 (s, 3H). **^{13}C NMR** (126 MHz, $CDCl_3$) δ: 146.6 (d, *J* = 33.1 Hz, C), 139.1 (d, *J* = 8.6 Hz, C), 138.1 (d, *J* = 13.1 Hz, C), 137.7 (d, *J* = 12.3 Hz, C), 136.9 (d, *J* = 1.6 Hz, C), 133.9 (d, *J* = 11.3 Hz, 2CH), 133.8 (d, *J* = 10.5 Hz, C), 133.8 (d, *J* = 11.6 Hz, 2CH), 133.7 (C), 132.5 (d, *J* = 6.9 Hz, C), 131.3 (d, *J* = 3.3 Hz, CH), 130.0 (d, *J* = 1.6 Hz, CH), 129.9 (CH), 128.5 (d, *J* = 6.2 Hz, 2CH), 128.5 (d, *J* = 2.8 Hz, 2CH), 128.4 (d, *J* = 6.6 Hz, 2CH), 128.1 (d, *J* = 3.8 Hz, 2CH), 127.7 (CH), 126.7 (CH), 126.6 (d, *J* = 2.8 Hz, CH), 126.5 (CH), 125.4 (CH), 20.3 (d, *J* = 2.8 Hz, CH_3). **^{31}P NMR** (202 MHz, $CDCl_3$) δ -13.5. **HRMS** (APCI+) *m/z* calcd. for $C_{29}H_{24}P$ [M+H]: 403.1610; found: 403.1614.

[11] Uozumi, Y.; Tanahashi, A.; Lee, S. Y.; Hayashi, T. *J. Org. Chem.* **1993**, *58*, 1945-1948.

Enantioselectivity of the product was determined by chiral HPLC analysis (Chiralpak IG3, IPA/hexane 15:85, 0.5 mL/min, λ=210 nm).

Racemic sample

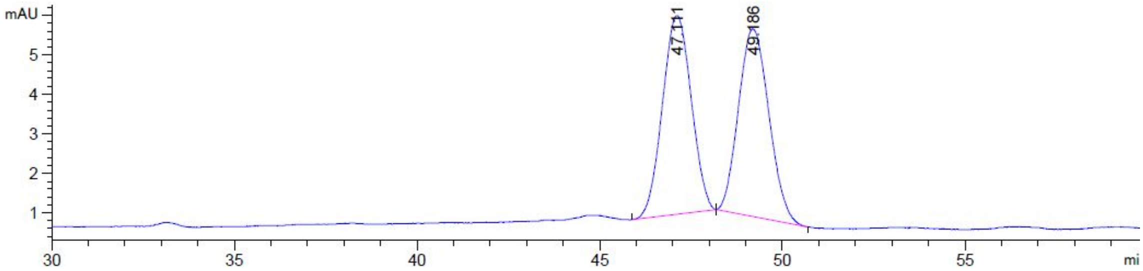

Peak #	RetTime [min]	Type	Width [min]	Area [mAU*s]	Height [mAU]	Area %
1	47.112	BB	0.8563	1823.21021	33.32220	50.1572
2	49.190	BB	0.9002	1811.78040	31.35147	49.8428

Chiral sample

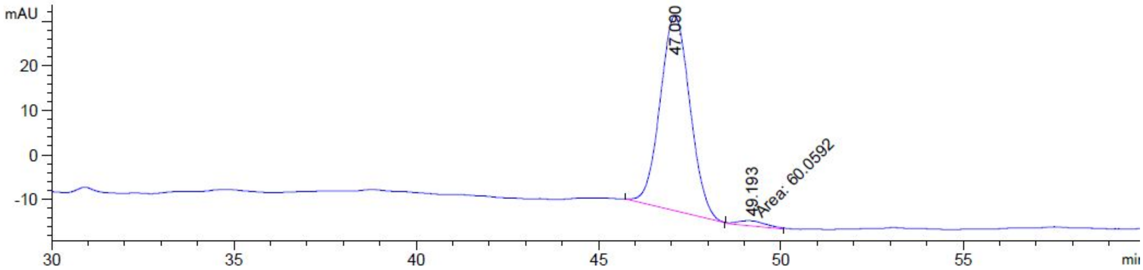

Peak #	RetTime [min]	Type	Width [min]	Area [mAU*s]	Height [mAU]	Area %
1	47.090	BB	0.8796	2484.64941	43.67977	97.6398
2	49.193	MM	0.8898	60.05915	1.12494	2.3602

6.4. Methylation and oxidative cleavage

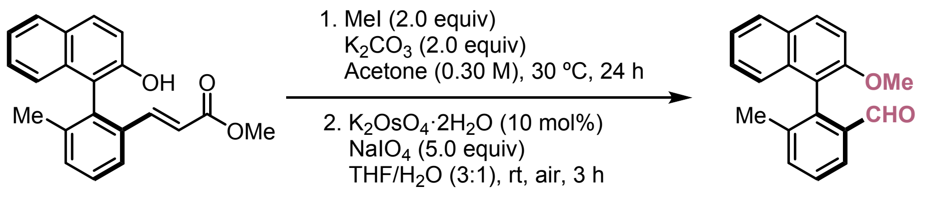

3ja **7**

To a suspension of **3ja** (318 mg, 1.00 mmol, 1.00 equiv, 99.9:0.1 er) and K_2CO_3 (276 mg, 2.00 mmol, 2.00 equiv) in acetone (3.3 mL), MeI (125 µL, 2.00 mmol, 2.00 equiv) was added and the mixture was stirred at 30 ºC under argon for 23 h. The reaction mixture was partitioned between AcOEt and water. The layers were separated, and the aqueous phase was extracted with AcOEt (x2). The combined organic phase was dried over Na_2SO_4 and concentrated under reduced pressure. The resulting residue was purified by flash column chromatography (AcOEt/hexane 10:90) to afford 256 mg (77%) of the methyl ether as a white solid. **Rf:** 0.55 (AcOEt/hexane 20:80, dark brown in *p*-anisaldehyde). **^{1}H NMR** (300 MHz, $CDCl_3$) δ: 7.94 (d, *J* = 9.2 Hz, 1H), 7.88 – 7.81 (m, 1H), 7.68 – 7.62 (m, 1H), 7.42 – 7.36 (m, 3H), 7.31 (td, *J* = 7.4, 1.5 Hz, 2H), 7.20 (d, *J* = 16.0 Hz, 1H), 7.08 – 7.01 (m, 1H), 6.29 (d, *J* = 15.9 Hz, 1H), 3.82 (s, 3H), 3.59 (s, 3H), 1.88 (s, 3H).

A solution of the above methylated product (100 mg, 0.30 mmol, 1.0 equiv) in THF (3.0 mL) was dropwise added to a stirred suspension of $K_2OsO_4·2H_2O$ (11.1 mg, 10 mol%) and $NaIO_4$ (321 mg, 1.5 mmol, 5.0 equiv) in water (1.5 mL) and the resulting suspension was stirred under air at rt for 3 h. The reaction was quenched with $Na_2S_2O_3$, and the mixture was partitioned between AcOEt and water. The layers were separated, and the aqueous phase was extracted with AcOEt. The combined organic phase was dried over Na_2SO_4 and concentrated under reduced pressure. The resulting residue was purified by flash column chromatography (AcOEt/hexane 5:95 to 10:90) to afford 49.4 mg (59%, 97:3 er) of **7** as a colorless sticky oil that foams under vacuum. **Rf:** 0.50 (AcOEt/hexane 15:85, intense garnet in *p*-anisaldehyde). **^{1}H NMR** (300 MHz, $CDCl_3$) δ: 9.47 (s, 1H), 8.01 – 8.94 (m, 2H), 7.90 – 7.83 (m, 1H), 7.62 (d, *J* = 7.4 Hz, 1H), 7.49 (t, *J* = 7.6 Hz, 1H), 7.40 (d, *J* = 9.2 Hz, 1H), 7.38 – 7.29 (m, 2H), 7.12 – 7.05 (m, 1H), 3.84 (s, 3H), 1.96 (s, 3H). **^{13}C NMR** (75 MHz, $CDCl_3$) δ: 193.2 (CHO), 154.3 (COMe), 140.4 (C), 139.0 (C), 135.8 (CH), 135.2 (C), 133.7 (C), 130.3 (CH), 129.0 (C), 128.3 (CH), 128.0 (CH), 127.3 (CH), 124.8 (CH), 124.5 (CH), 124.0 (CH), 118.7 (C), 112.9 (CH), 56.4 (OCH_3), 19.5 (CH_3). **HRMS** (APCI+) *m/z* calcd. for $C_{19}H_{17}O_2$ [M+H]: 277.1223; found: 277.1229.

Enantioselectivity of the product was determined by chiral HPLC analysis (Chiralpak IE3, IPA/hexane 1:99, 1.0 mL/min, λ=220 nm).

Racemic sample

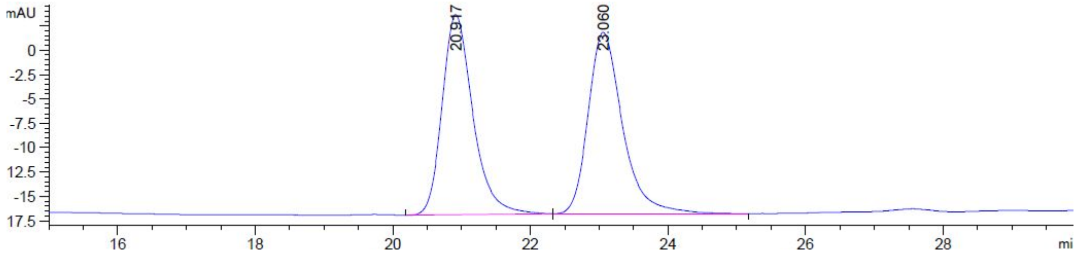

Peak #	RetTime [min]	Type	Width [min]	Area [mAU*s]	Height [mAU]	Area %
1	20.917	BB	0.4724	638.13586	20.54253	49.6506
2	23.060	BB	0.5254	647.11621	18.62170	50.3494

Chiral sample (3 h of reaction time)

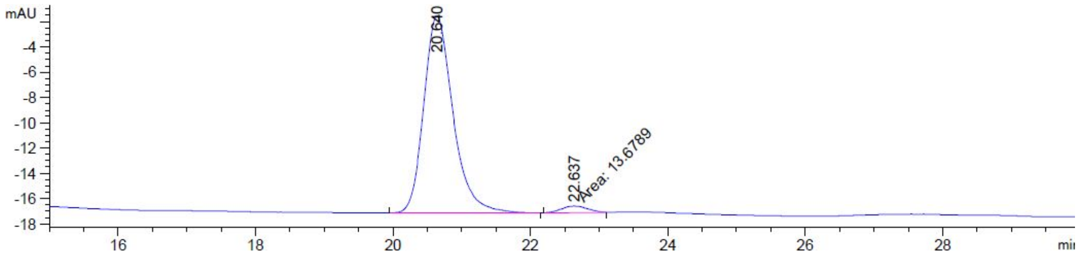

Peak #	RetTime [min]	Type	Width [min]	Area [mAU*s]	Height [mAU]	Area %
1	20.640	BB	0.4495	462.95618	15.63363	97.1301
2	22.637	MM	0.4369	13.67886	5.21842e-1	2.8699

Chiral sample (28 h of reaction time, 0.5 mL/min)

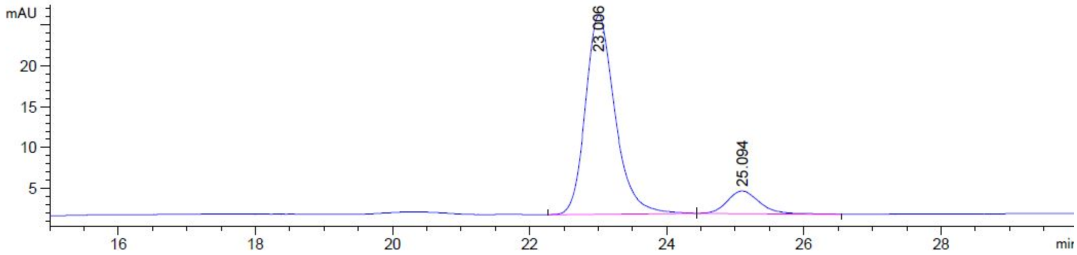

Peak #	RetTime [min]	Type	Width [min]	Area [mAU*s]	Height [mAU]	Area %
1	23.006	BB	0.4551	727.27478	24.30830	89.0391
2	25.094	BB	0.4809	89.52942	2.75658	10.9609

Note: Aldehyde **7** is not very conformationally stable in solution. After 3 h of reaction time full conversion was observed and there is almost no depletion in the enantiopurity. However, if the reaction is let to stir overnight the enantiopurity decreases significantly.

6.5. Oxidation of the aldehyde to the carboxylic acid

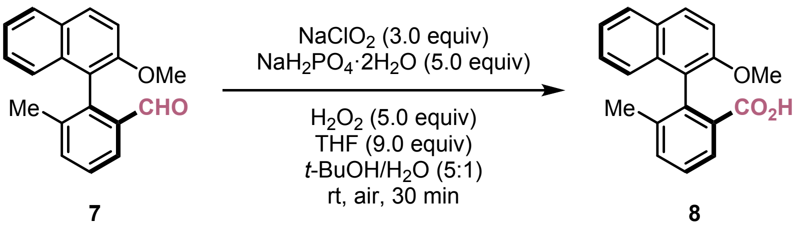

A solution of **7** (44.7 mg, 0.16 mmol, 1.0 equiv), $NaClO_2$ (80%, 54.9 mg, 0.49 mmol, 3.0 equiv), $NaH_2PO_4·2H_2O$ (126 mg, 0.81 mmol, 5.0 equiv), H_2O_2 (30%, 83 µL, 0.81 mmol, 5.0 equiv) and THF (0.12 mL, 1.46 mmol, 9.0 equiv) in *t*-BuOH (1.6 mL) and water (0.32 mL) was stirred at rt under air for 30 min. The volatiles were removed under reduced pressure and the aqueous residue was partitioned between water and CH_2Cl_2. The layers were separated, and the aqueous phase was extracted with CH_2Cl_2 (x2). The combined organic phase was dried over Na_2SO_4, and concentrated under reduced pressure. The resulting residue was purified by flash column chromatography (AcOH/AcOEt/hexane 1:10:90 to 1:15:85) to afford 38.5 mg (81%, 95:5 er) of **8** as a white solid. **Rf:** 0.33 (AcOH/AcOEt/hexane 1:25:75, red when freshly stained in *p*-anisaldehyde). **^{1}H NMR** (300 MHz, $CDCl_3$) δ: 9.69 (br s, 1H), 7.96 (d, *J* = 7.8 Hz, 1H), 7.87 (d, *J* = 9.1 Hz, 1H), 7.82 (d, *J* = 7.9 Hz, 1H), 7.54 (d, *J* = 7.5 Hz, 1H), 7.41 (t, *J* = 7.7 Hz, 1H), 7.35 – 7.22 (m, 3H), 7.05 (d, *J* = 8.0 Hz, 1H), 3.75 (s, 3H), 1.90 (s, 3H). **^{13}C NMR** (75 MHz, $CDCl_3$) δ: 172.4 (CO_2H), 153.4 (COMe), 139.1 (C), 137.6 (C), 134.5 (CH), 133.0 (C), 130.8 (C), 129.2 (CH), 129.1 (C), 128.8 (CH), 128.1 (CH), 127.5 (CH), 126.6 (CH), 124.2 (CH), 123.5 (CH), 123.0 (C), 113.6 (CH), 56.6 (OCH_3), 20.2 (CH_3). **HRMS** (APCI+) *m/z* calcd. for $C_{19}H_{17}O_3$ [M+H]: 293.1172; found: 293.1181.

Enantioselectivity of the product was determined by chiral HPLC analysis (Chiralpak IG3, IPA/hexane 10:90, 1.0 mL/min, λ=210 nm).

Racemic sample

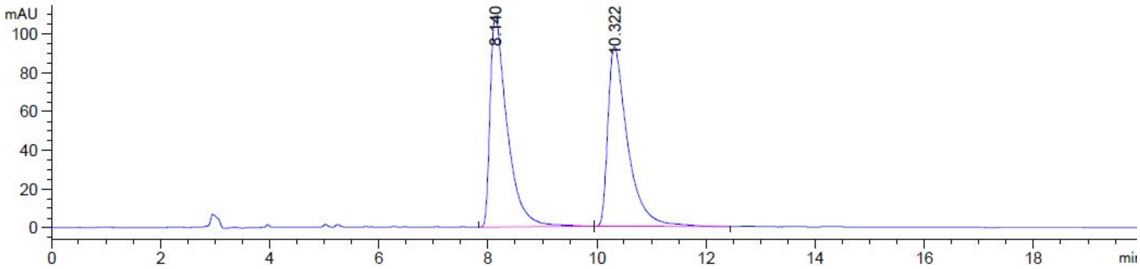

Peak #	RetTime [min]	Type	Width [min]	Area [mAU*s]	Height [mAU]	Area %
1	8.140	BB	0.3166	2381.49731	109.11007	50.3912
2	10.322	BB	0.3726	2344.52563	92.86121	49.6088

Chiral sample

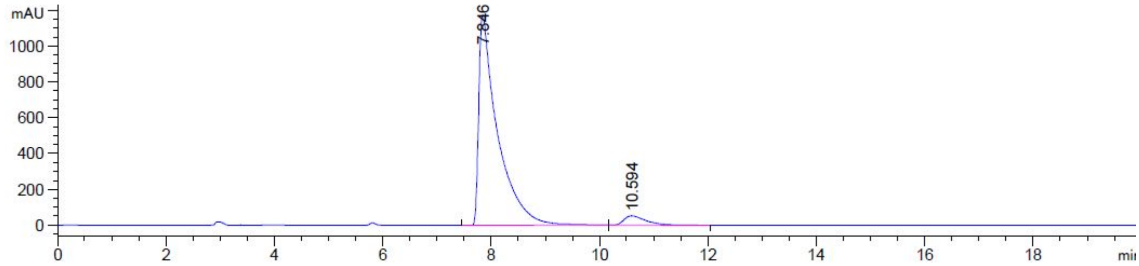

Peak #	RetTime [min]	Type	Width [min]	Area [mAU*s]	Height [mAU]	Area %
1	7.846	BB	0.3269	2.82849e4	1174.55981	95.0714
2	10.594	BB	0.4157	1466.30615	52.20145	4.9286

Notes: The pure solid carboxylic acid is insoluble in most part of common organic solvents. However, the reamining acetic acid from the chromatographic purification helps to dissolve it in chloroform, so the NMR experiments were recorded previously to drying it and signals of residual dichloromethane and acetic acid can be observed in the spectra. The yield was calculated after solidification and drying under high vacuum.

6.6. Reductive amination and hydroxyl deprotection

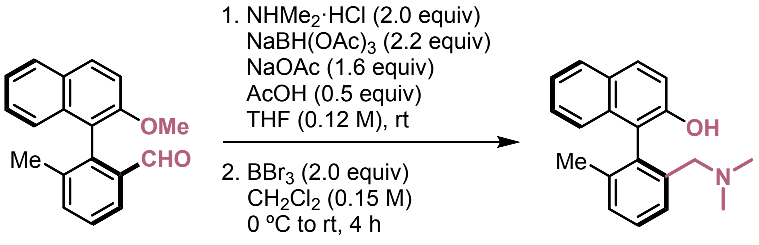

7 **9**

Following a literature procedure[12], a solution of **7** (30.2 mg, 0.11 mmol, 1.00 equiv, 89:11 er), $NHMe_2$·HCl (17.8 mg, 0.22 mmol, 2.00 equiv), NaOAc (14.3 mg, 0.18 mmol, 1.60 equiv) and AcOH (3 µL, 0.055 mmol, 0.50 equiv) in THF (0.95 mL) under argon was stirred at rt for 10 min. $NaBH(OAc)_3$ (51.7 mg, 0.24 mmol, 2.23 equiv) was added and the mixture was stirred at rt for 19 h. The solvent was removed under reduced pressure and the residue was partitioned between Et_2O and water. The biphasic mixture was extracted with 10% aq. citric acid (x3). The combined aqueous phase was washed with Et_2O, basified to pH 8 with solid $NaHCO_3$, and extracted with AcOEt (x3). The combined AcOEt organic phase was dried over Na_2SO_4 and concentrated under reduced pressure to afford a colorless oil that was used without further purification.

A solution of above oil in CH_2Cl_2 (0.15 M) under argon was cooled to 0 ºC and BBr_3 (1.0 M in CH_2Cl_2, 2.0 equiv) was dropwise added. The resulting mixture was warmed to rt and stirred for 3 h. The reaction was quenched with sat. aq. $NaHCO_3$ and the mixture was partitioned between water and CH_2Cl_2. The layers were separated, and the aqueous phase was extracted with CH_2Cl_2 (x2). The combined organic phase was dried over Na_2SO_4 and concentrated under reduced pressure. The resulting residue was purified by flash column chromatography (NEt_3/MeOH/CH_2Cl_2 0:2:98 to 0.5:3:97) to afford 13.7 mg (43%, 2 steps, 88:12 er) of **9** as a white solid. **Rf:** 0.40 (MeOH/CH_2Cl_2 10:90, white in *p*-anisaldehyde). **^{1}H NMR** (300 MHz, $CDCl_3$) δ: 7.85 – 7.77 (m, 2H), 7.39 – 7.32 (m, 3H), 7.31 – 7.18 (m, 3H), 6.97 (d, *J* = 7.6 Hz, 1H), 3.52 (d, *J* = 11.7 Hz, 1H), 2.99 (d, *J* = 11.8 Hz, 1H), 2.22 (s, 6H), 1.78 (s, 3H). **^{13}C NMR** (75 MHz, $CDCl_3$) δ: 153.5 (COH), 139.5 (C), 137.3 (C), 136.7 (C), 133.6 (C), 130.6 (CH), 129.4 (CH), 128.4 (CH), 128.1 (CH), 127.2 (CH), 126.2 (CH), 124.4 (CH), 123.5 (C), 123.2 (CH), 122.9 (CH), 63.7 ($2NCH_3$), 44.0 (CH_2), 20.6 (CH_3). **HRMS** (APCI+) *m/z* calcd. for $C_{20}H_{22}NO$ [M+H]: 292.1696; found: 292.1670.

---

[12] Dong, Z.; Wang, J.; Dong, G. *J. Am. Chem. Soc.* **2015**, *137*, 5887-5890.

Enantioselectivity of the product was determined by chiral HPLC analysis (Chiralpak OZ-H, IPA/hexane 15:85, 0.5 mL/min, λ=220 nm).

Racemic sample

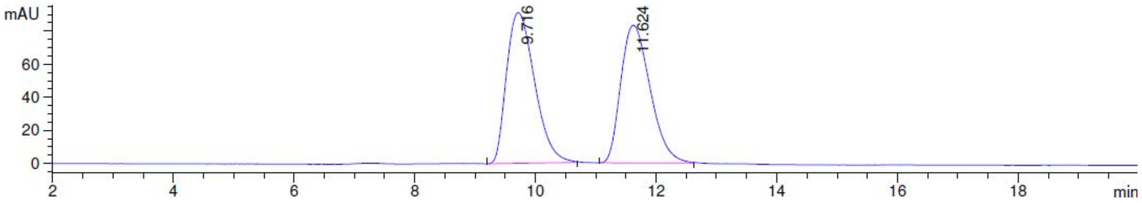

Peak #	RetTime [min]	Type	Width [min]	Area [mAU*s]	Height [mAU]	Area %
1	9.716	PB	0.5155	2951.29932	91.19952	51.1585
2	11.624	PB	0.5305	2817.62793	83.32009	48.8415

Chiral sample

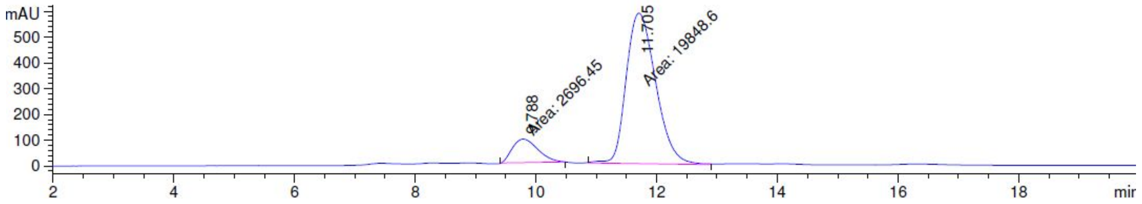

Peak #	RetTime [min]	Type	Width [min]	Area [mAU*s]	Height [mAU]	Area %
1	9.788	MM	0.4936	2696.44653	91.04956	11.9603
2	11.705	MM	0.5666	1.98486e4	583.86511	88.0397

6.7. Quaternarization of the amine

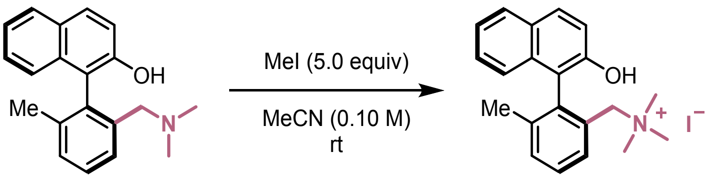

Following a literature procedure[13], to a solution of **9** (19.6 mg, 0.067 mmol, 1.0 equiv) in MeCN (0.67 mL), MeI (8 µL, 0.14 mmol, 2.0 equiv) was added and the mixture was stirred at rt under argon for 21.5 h. More MeI (12 µL, 3.0 equiv) was added and the mixture was stirred for another 7 h. The solvent was removed under reduced pressure and the resulting residue was purified by flash column chromatography (MeOH/CH_2Cl_2 5:95) to afford 18.4 mg (63%) of **10** as a cream solid. **Rf:** 0.15 (MeOH/CH_2Cl_2 10:90, white in *p*-anisaldehyde). **^{1}H NMR** (300 MHz, $CDCl_3$) δ: 7.87 – 7.59 (m, 5H), 7.46 (d, *J* = 7.1 Hz, 1H), 7.39 – 7.28 (m, 3H), 6.97 – 6.86 (m, 1H), 5.32 (s, 1H), 4.46 (d, *J* = 12.8 Hz, 1H), 4.24 (d, *J* = 12.9 Hz, 1H), 2.95 (s, 9H), 1.97 (s, 3H). **^{13}C NMR** (75 MHz, $CDCl_3$) δ: 151.5 (COH), 140.6 (C), 138.8 (C), 133.2 (CH), 132.8 (C), 132.1 (CH), 130.6 (CH), 129.0 (CH), 128.4 (CH), 127.7 (CH), 127.3 (C), 123.8 (CH), 123.2 (CH), 119.3 (CH), 117.1 (C), 67.7 (CH_2), 53.5 (NCH_3), 20.8 (CH_3).

[13] Deng, Y.; Shi, X.; Shi, G.; Lu, X.; Luo, J.; Deng, L. *JACS Au* **2022**, *2*, 2678-2685.

6.8. Hydrogenation of the double bond

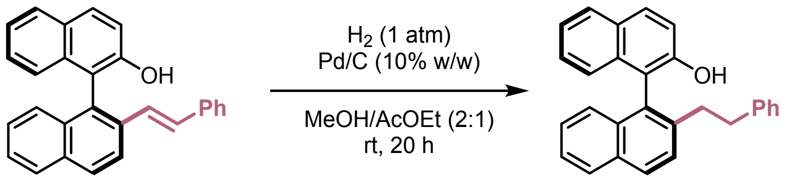

3ae **11**

To a solution of **3ae** (153 mg, 0.41 mmol, 1.0 equiv, 93:7 er) in MeOH/AcOEt (2:1, 3.1 mL) under N_2, Pd/C (10% w/w, 43.6 mg, 10 mol%) was added, and the black suspension was purged with H_2. After stirring at rt under H_2 atmosphere (balloon) for 20 h, the reaction mixture was filtered through Celite, and the solids were washed with AcOEt. The filtrate was concentrated under reduced pressure and the resulting residue was purified by flash column chromatography (AcOEt/hexane 2:98) to afford 102 mg (67%, 93:7 er) of **11** as white fluffy solid. **Rf:** 0.57 (AcOEt/hexane 20:80, grey in *p*-anisaldehyde). **^{1}H NMR** (300 MHz, $CDCl_3$) δ: 8.03 – 7.89 (m, 4H), 7.60 (d, *J* = 8.5 Hz, 1H), 7.49 (ddd, *J* = 8.1, 6.4, 1.6 Hz, 1H), 7.39 (d, *J* = 8.9 Hz, 1H), 7.36 – 7.22 (m, 4H), 7.20 – 7.08 (m, 3H), 7.03 (d, *J* = 8.4 Hz, 1H), 6.85 (dt, *J* = 7.6, 1.3 Hz, 2H), 4.75 (s, 1H), 2.83 – 2.67 (m, 4H). **^{13}C NMR** (75 MHz, $CDCl_3$) δ: 151.1 (COH), 141.6 (C), 141.0 (C), 133.9 (C), 133.5 (C), 132.9 (C), 130.1 (CH), 129.3 (CH), 129.3 (C), 128.9 (C), 128.4 (4CH), 128.3 (2CH), 128.2 (CH), 127.1 (CH), 126.9 (CH), 126.0 (2CH), 125.8 (CH), 124.9 (CH), 123.6 (CH), 117.6 (CH), 117.4 (C), 37.2 (CH_2), 36.5 (CH_2). **HRMS** (APCI+) *m/z* calcd. for $C_{28}H_{23}O$ [M+H]: 375.1743; found: 375.1733.

Enantioselectivity of the product was determined by chiral HPLC analysis (Chiralpak IA3, IPA/hexane 2:98, 1.0 mL/min, λ=220 nm).

Racemic sample

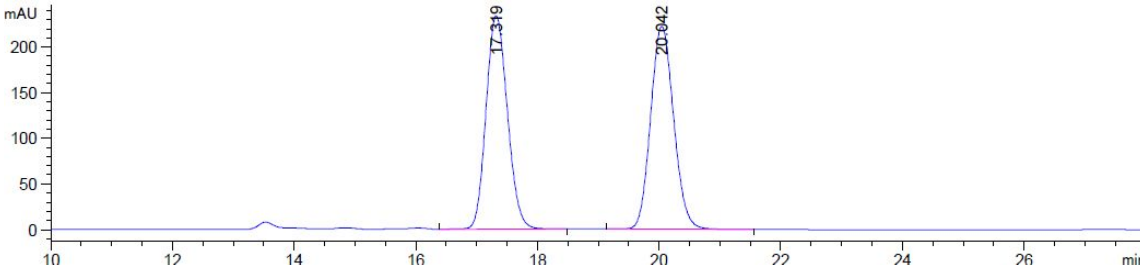

Peak #	RetTime [min]	Type	Width [min]	Area [mAU*s]	Height [mAU]	Area %
1	17.319	BB	0.3832	5710.61621	233.63649	49.4373
2	20.042	BB	0.4127	5840.61035	222.18661	50.5627

Chiral sample

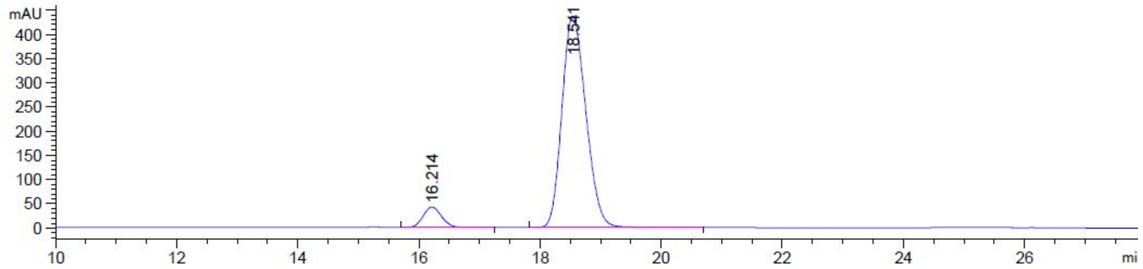

Peak #	RetTime [min]	Type	Width [min]	Area [mAU*s]	Height [mAU]	Area %
1	16.214	BB	0.3308	898.57483	42.13327	6.9737
2	18.541	BB	0.4282	1.19866e4	436.71841	93.0263

6.9. Amine-alcohol exchange by Smiles rearrangement

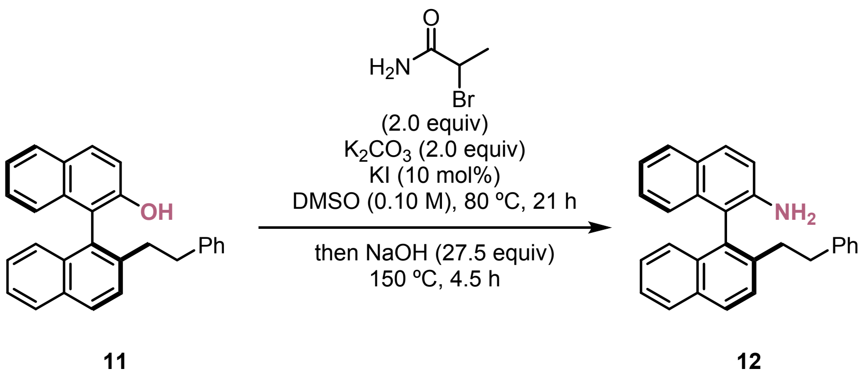

Following a slight modification of a reported procedure[14], a solution of **11** (47.6 mg, 0.127 mmol, 1.0 equiv, 93:7 er), 2-bromopropionamide (38.6 mg, 0.254 mmol, 2.0 equiv), K_2CO_3 (35.1 mg, 0.254 mmol, 2.0 equiv) and KI (2.1 mg, 10 mol%) in DMSO (1.3 mL) was stirred at 80 °C under argon for 21 h. NaOH (140 mg, 3.50 mmol, 27.5 equiv) was added, and the mixture was stirred at 150 °C for 4.5 h. After cooling to rt, the reaction mixture was partitioned between water and AcOEt. The layers were separated, and the aqueous phase was extracted with AcOEt (x2). The combined organic phase was washed with water and brine, dried over Na_2SO_4, and concentrated under reduced pressure. The resulting residue was purified by flash column chromatography (AcOEt/hexane 3:97 to 4:96) to afford 27.8 mg (59%, 92.5:7.5 er) of **12** as a white solid. **Rf:** 0.55 (AcOEt/hexane 20:80, yellow in *p*-anisaldehyde). **^{1}H NMR** (300 MHz, $CDCl_3$) δ: 7.99 – 7.92 (m, 2H), 7.89 – 7.82 (m, 2H), 7.61 (d, *J* = 8.5 Hz, 1H), 7.48 (ddd, *J* = 8.1, 4.8, 3.3 Hz, 1H), 7.33 – 7.12 (m, 8H), 6.97 (d, *J* = 8.3 Hz, 1H), 6.89 – 6.83 (m, 2H), 3.45 (s, 2H), 2.86 – 2.74 (m, 4H). **^{13}C NMR** (75 MHz, $CDCl_3$) δ: 142.2 (C), 142.1 (C), 139.8 (C), 134.4 (C), 133.0 (C), 133.0 (C), 132.3 (C), 129.2 (CH), 128.6 (CH), 128.3 (2CH), 128.3 (CH), 128.23 (CH), 128.2 (CH), 128.1 (CH), 126.7 (CH), 126.6 (CH), 126.0 (CH), 125.8 (2CH), 125.6 (CH), 124.4 (CH), 122.4 (CH), 118.2 (CH), 116.0 (C), 37.0 (CH_2), 36.4 (CH_2). **HRMS** (APCI+) *m/z* calcd. for $C_{28}H_{24}N$ [M+H]: 374.1903; found: 374.1905.

[14] Chang, X.; Zhang, Q.; Guo, C. *Org. Lett.* **2019**, *21*, 4915-4918.

Enantioselectivity of the product was determined by chiral HPLC analysis (Chiralpak IA3, IPA/hexane 2:98, 1.0 mL/min, λ=220 nm).

Racemic sample

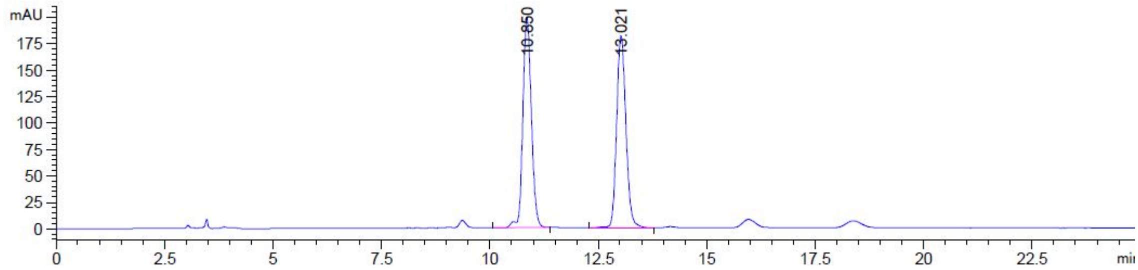

Peak #	RetTime [min]	Type	Width [min]	Area [mAU*s]	Height [mAU]	Area %
1	10.850	VB R	0.2108	2724.84790	199.10034	50.1283
2	13.021	BB	0.2302	2710.89551	181.54243	49.8717

Chiral sample

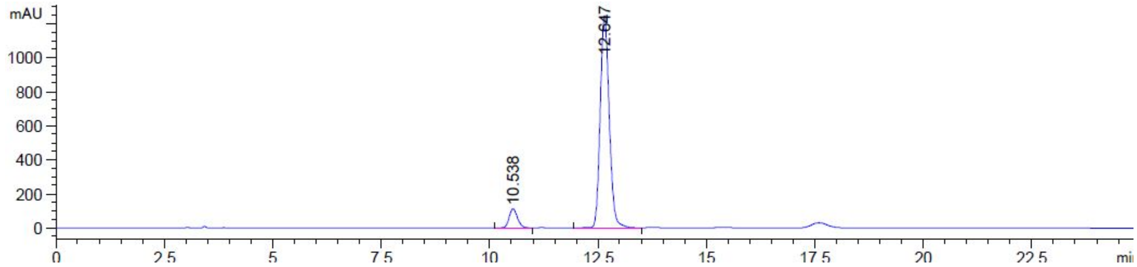

Peak #	RetTime [min]	Type	Width [min]	Area [mAU*s]	Height [mAU]	Area %
1	10.538	BB	0.2036	1465.20313	111.30727	7.5024
2	12.647	BB	0.2253	1.80647e4	1244.68542	92.4976

7. Mechanistic proposal and stereomodel for the C-H activation step

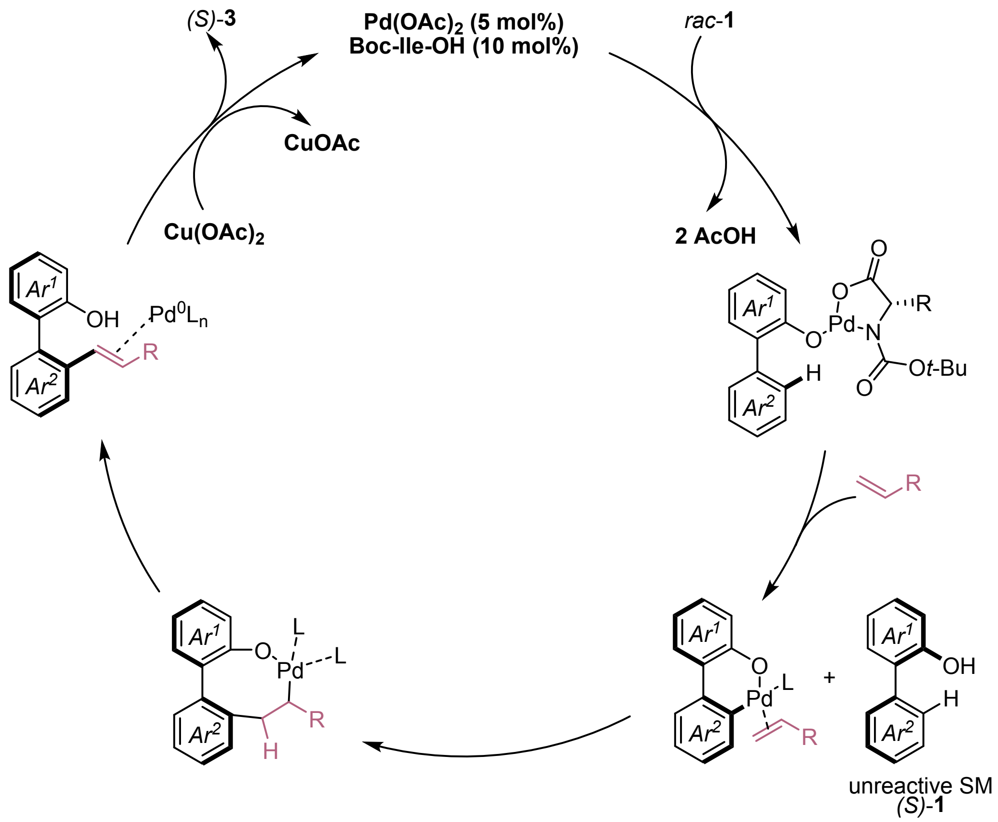

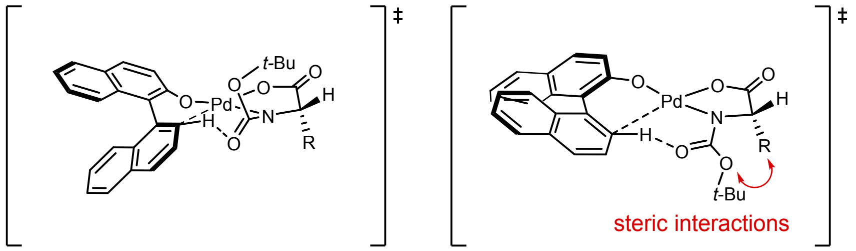

NOTE: Plausible qualitative stereomodel for the C-H activation step

8. NMR Spectra

^{1}H NMR (300 MHz, $CDCl_3$)

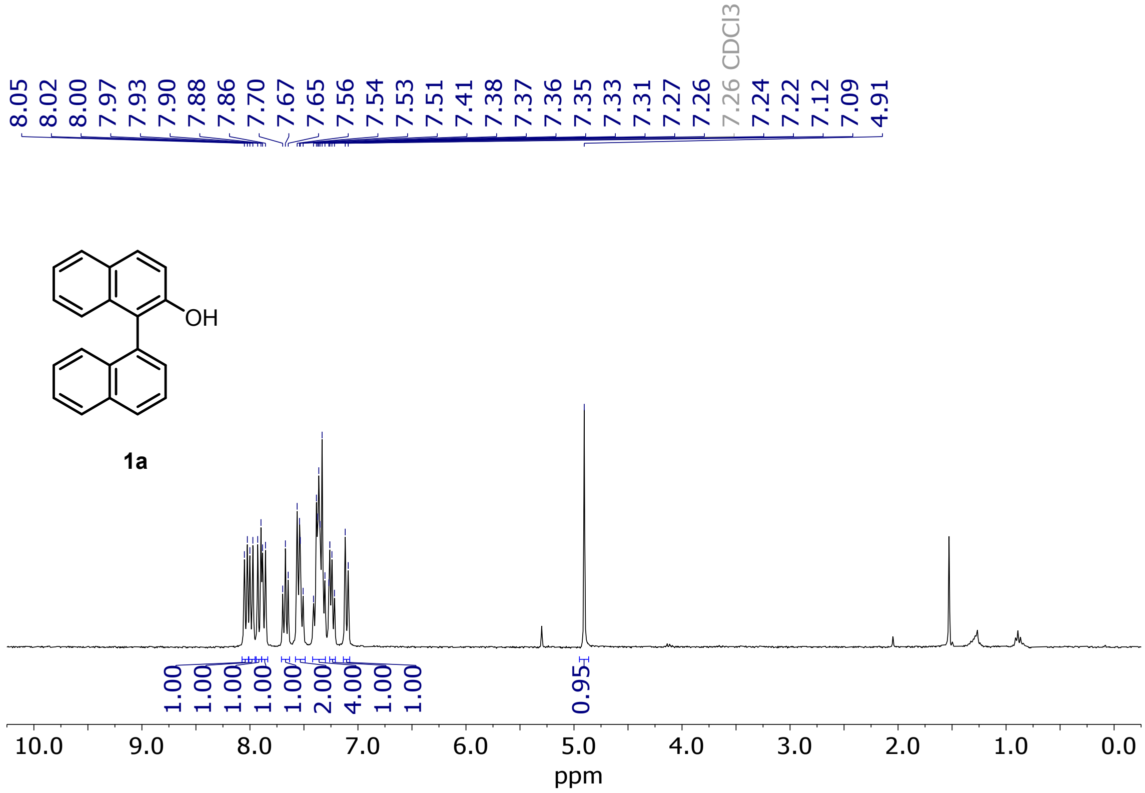

DEPT-135

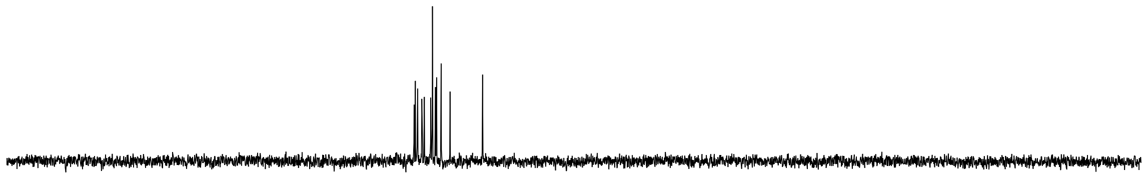

^{13}C NMR (75 MHz, $CDCl_3$)

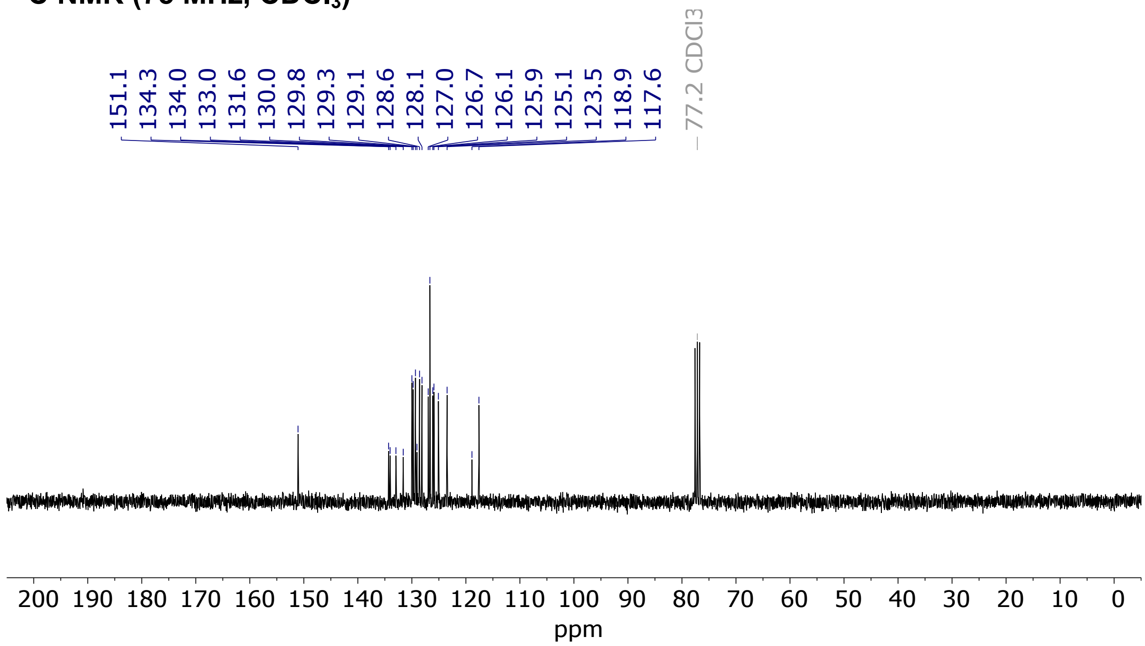

^{1}H NMR (300 MHz, $CDCl_3$)

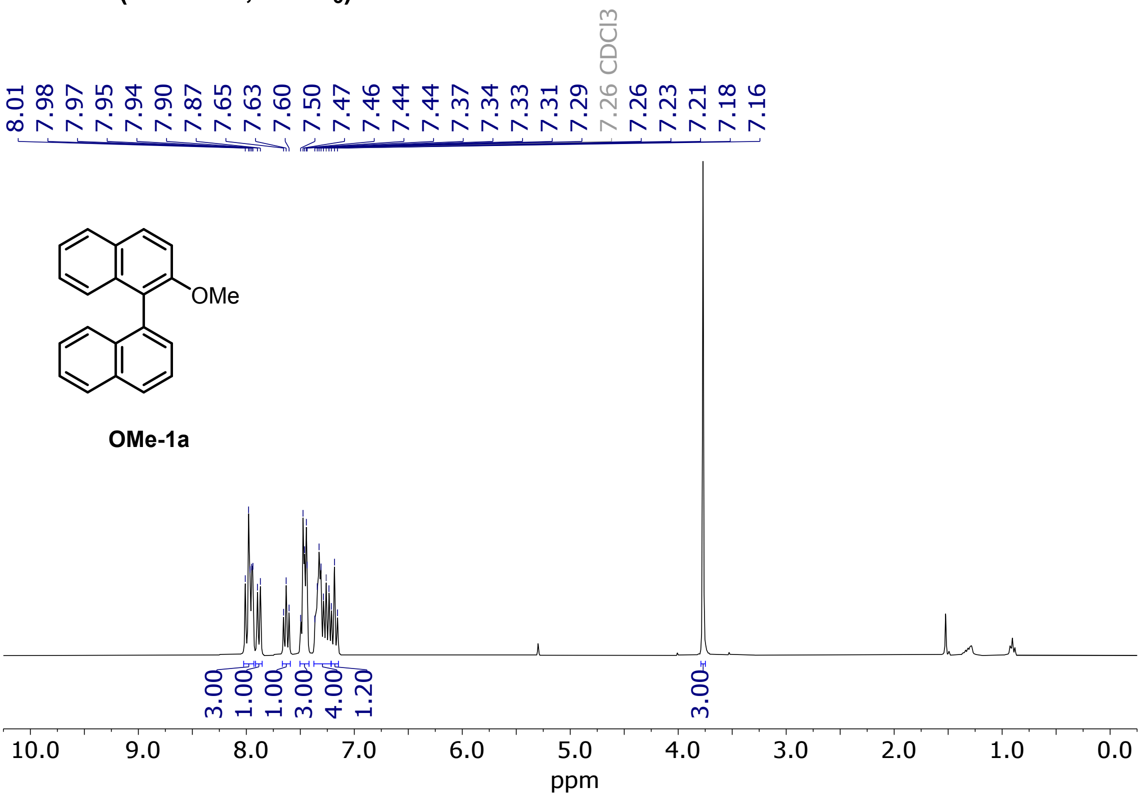

DEPT-135

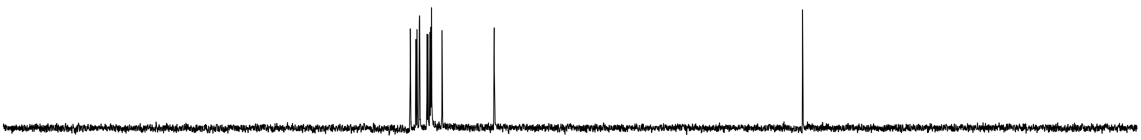

^{13}C NMR (75 MHz, $CDCl_3$)

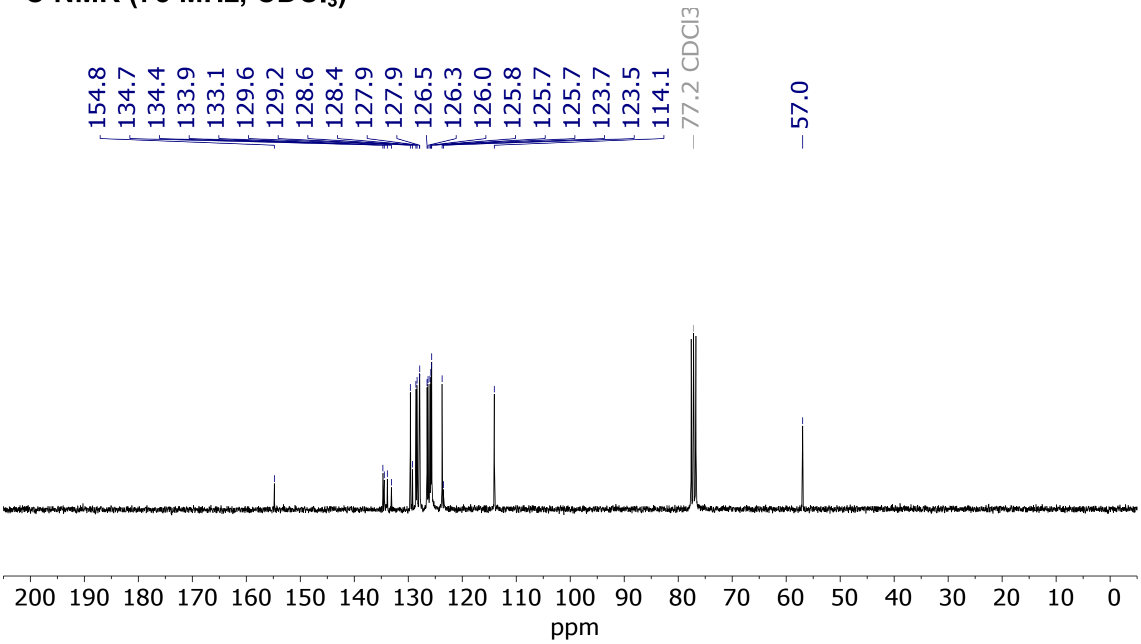

^{1}H NMR (300 MHz, $CDCl_3$)

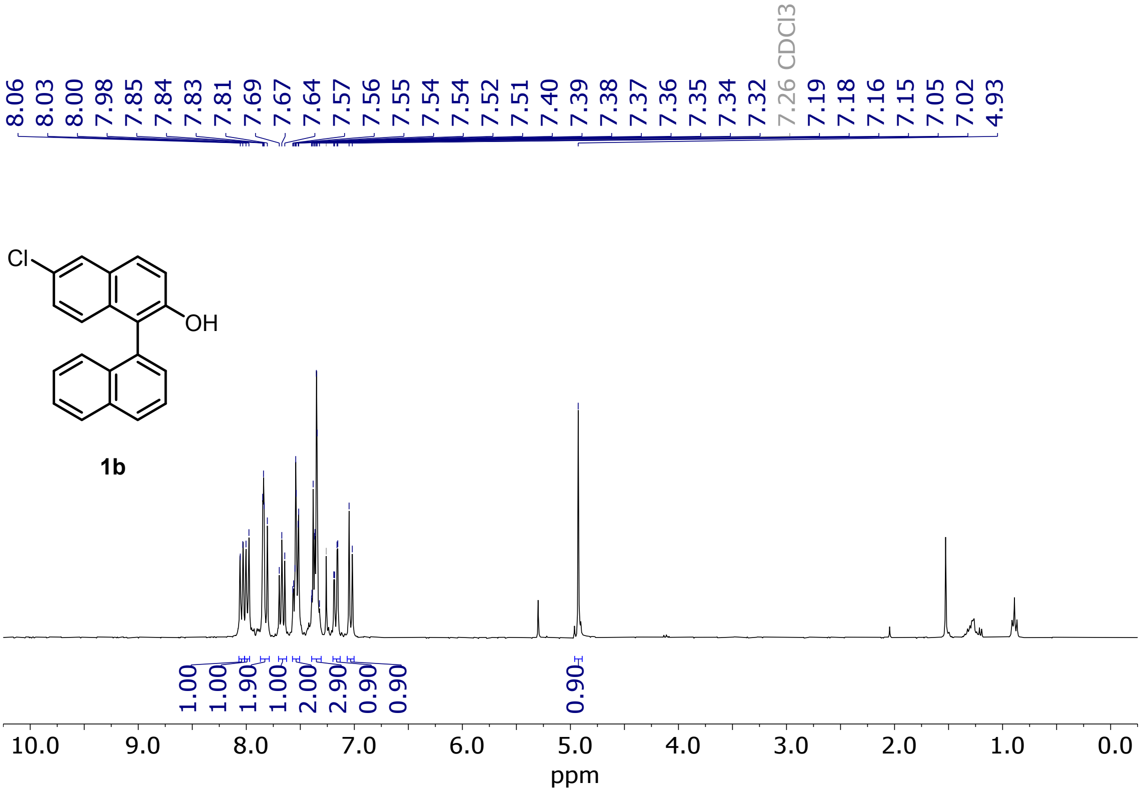

1b

DEPT-135

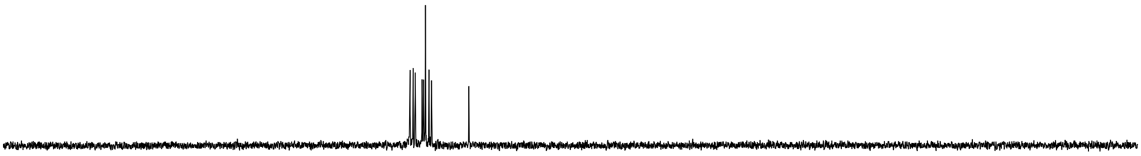

^{13}C NMR (75 MHz, $CDCl_3$)

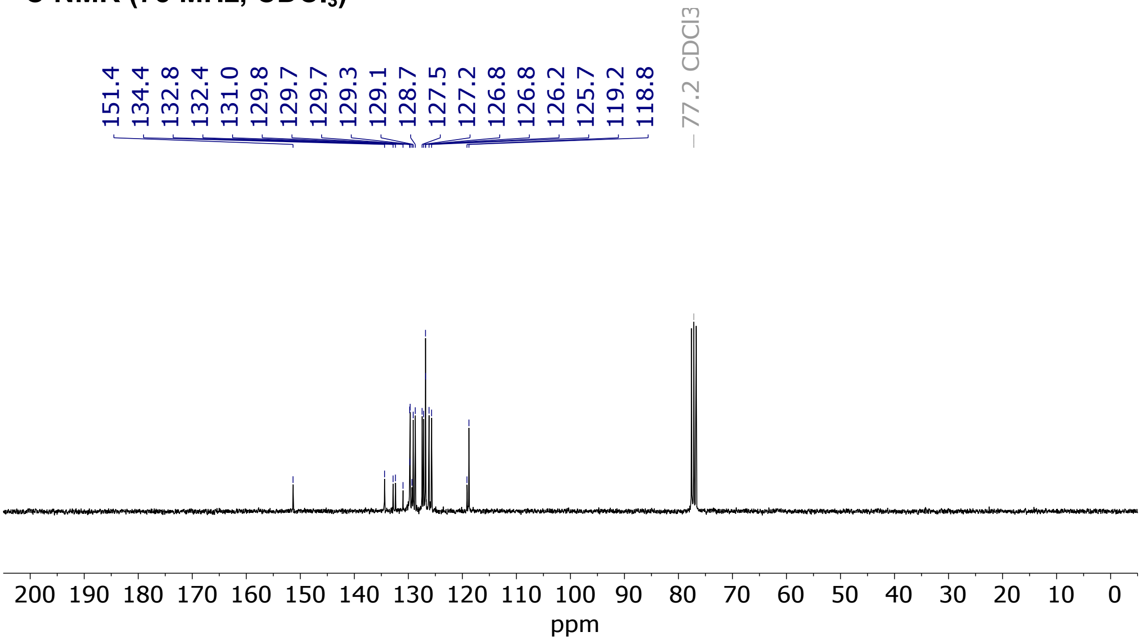

^{1}H NMR (300 MHz, DMSO-d_6)

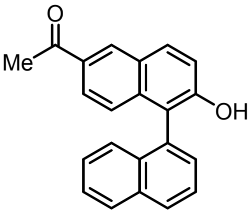

1c

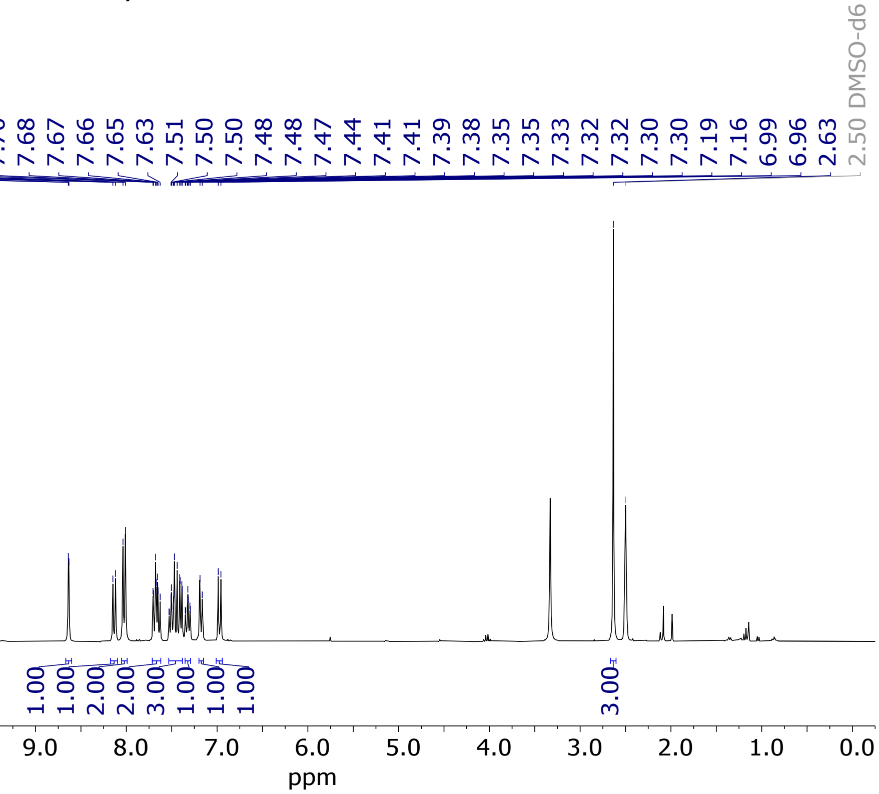

DEPT-135

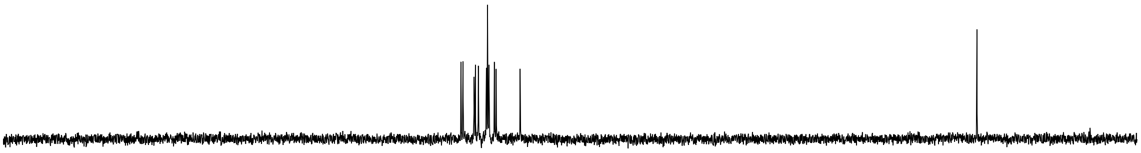

^{13}C NMR (75 MHz, DMSO-d_6)

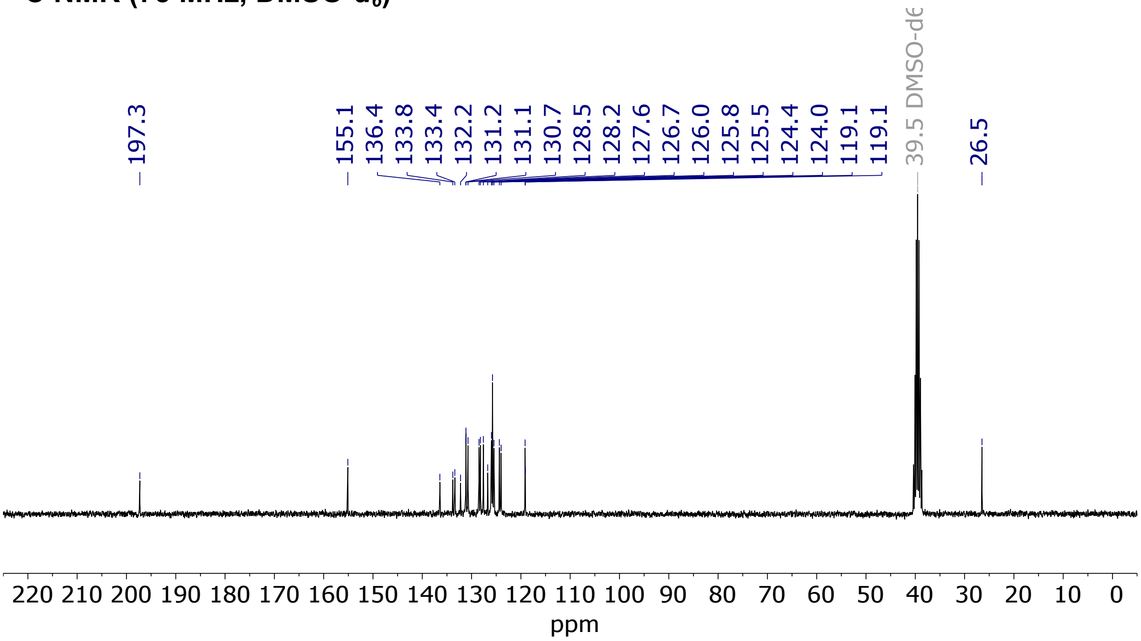

^{1}H NMR (300 MHz, $CDCl_3$)

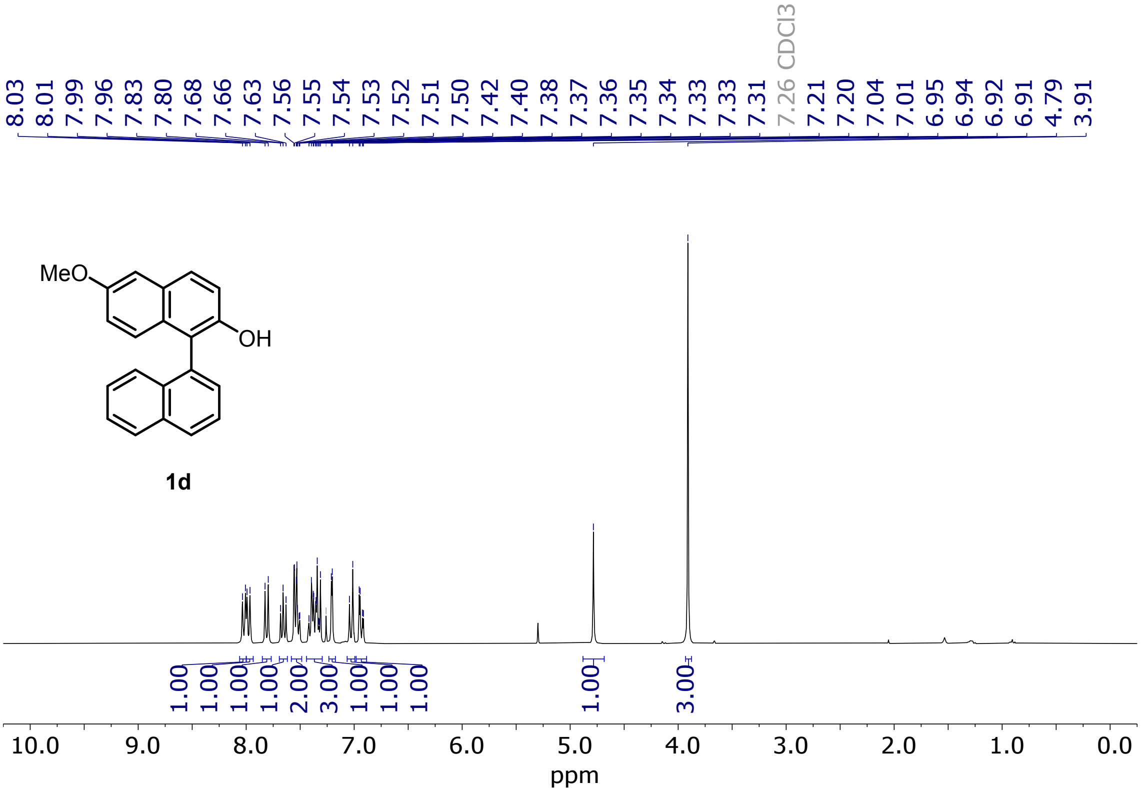

1d

DEPT-135

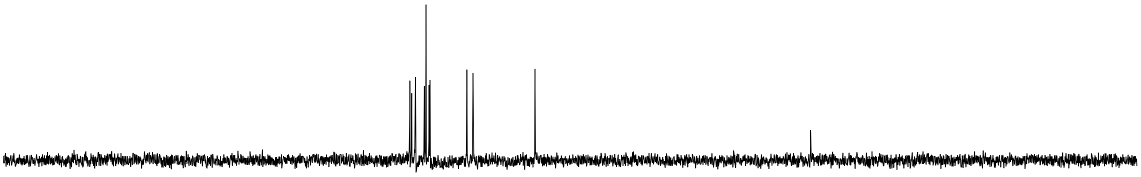

^{13}C NMR (75 MHz, $CDCl_3$)

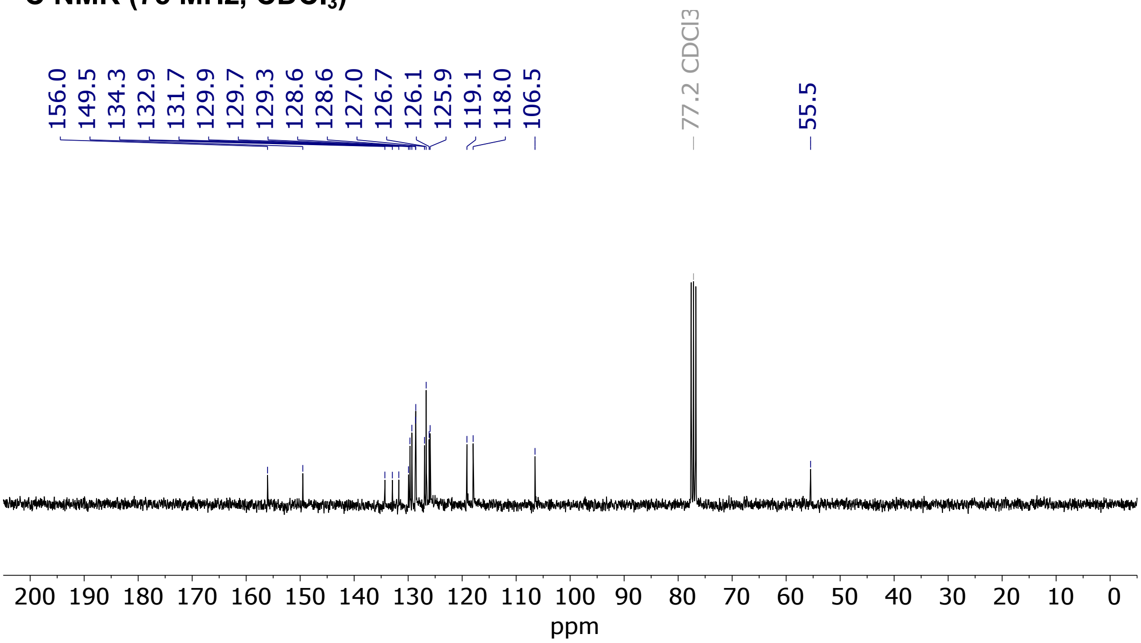

^{1}H NMR (300 MHz, $CDCl_3$)

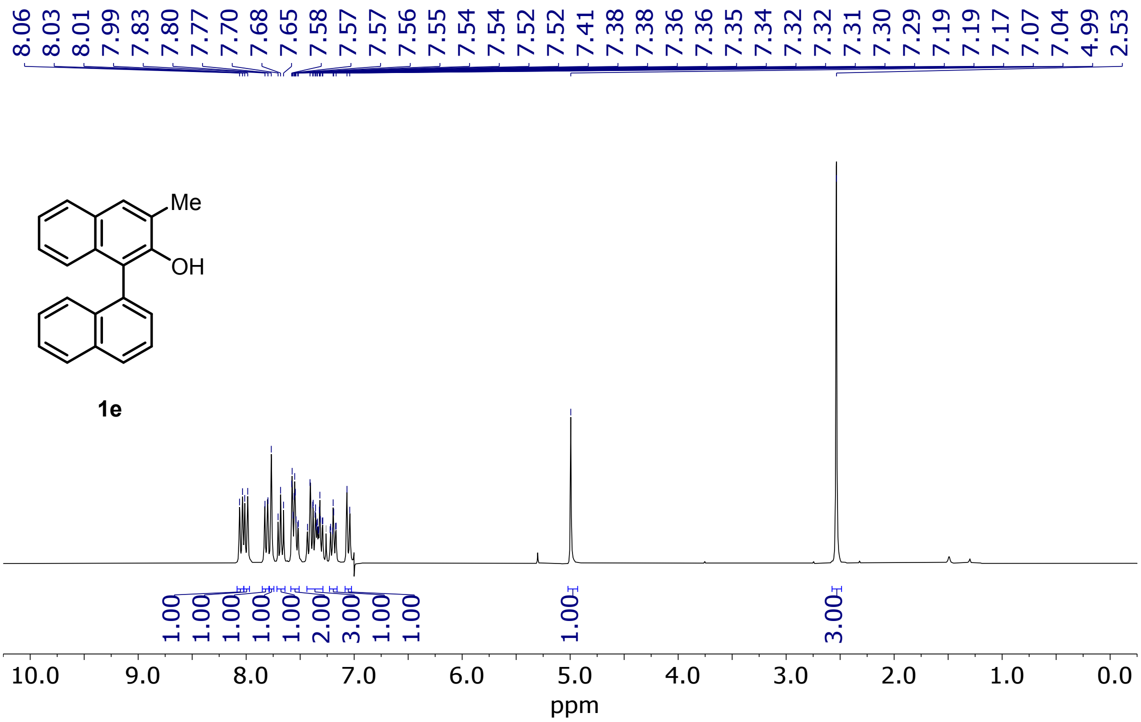

DEPT-135

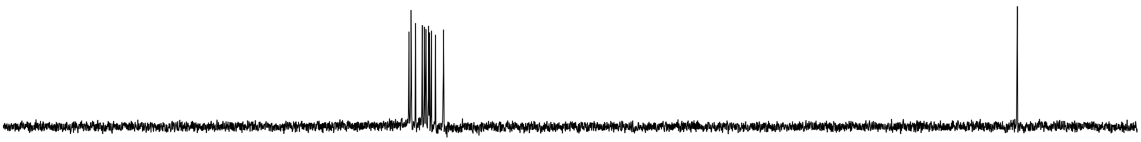

^{13}C NMR (75 MHz, $CDCl_3$)

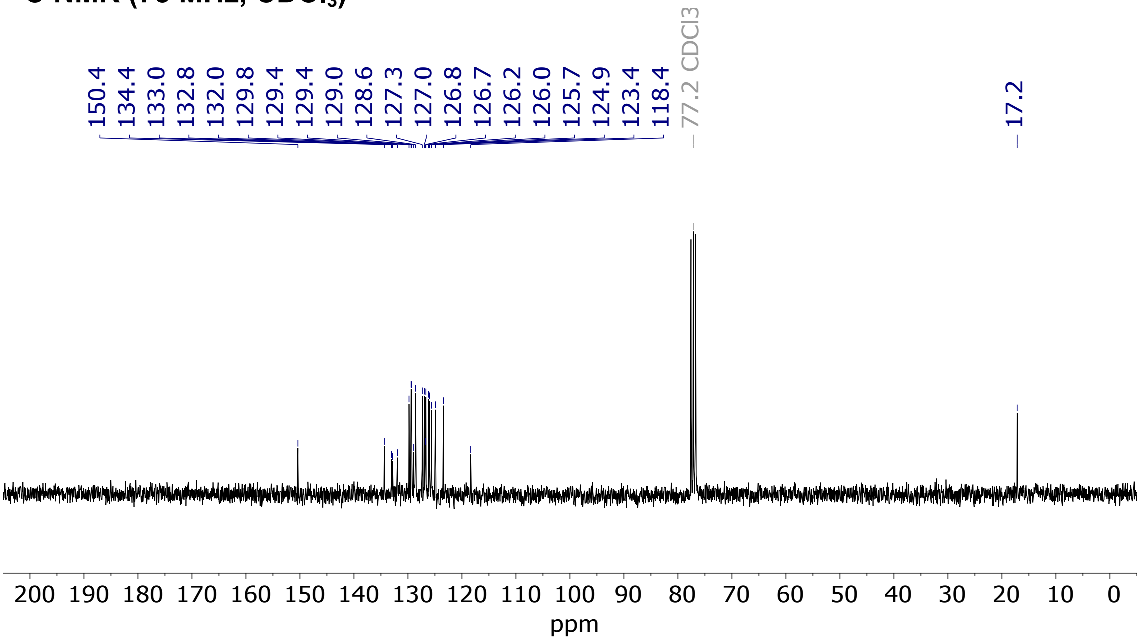

^{1}H NMR (500 MHz, $CDCl_3$)

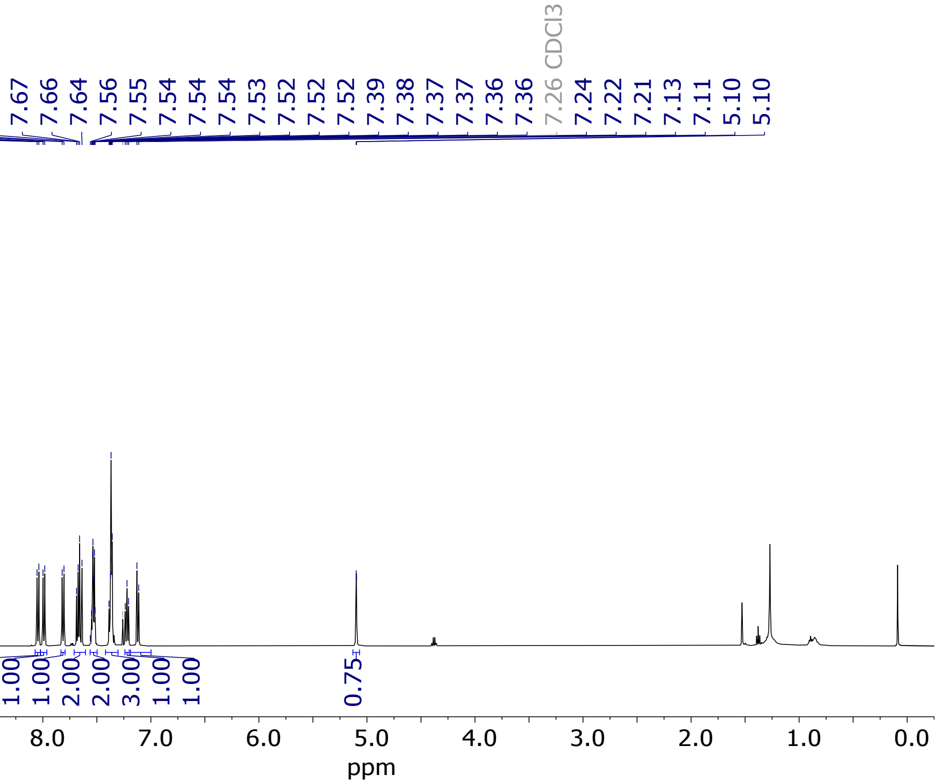

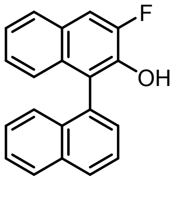

1f

DEPT-135

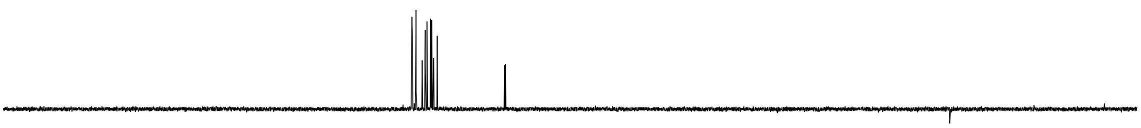

^{13}C NMR (126 MHz, $CDCl_3$)

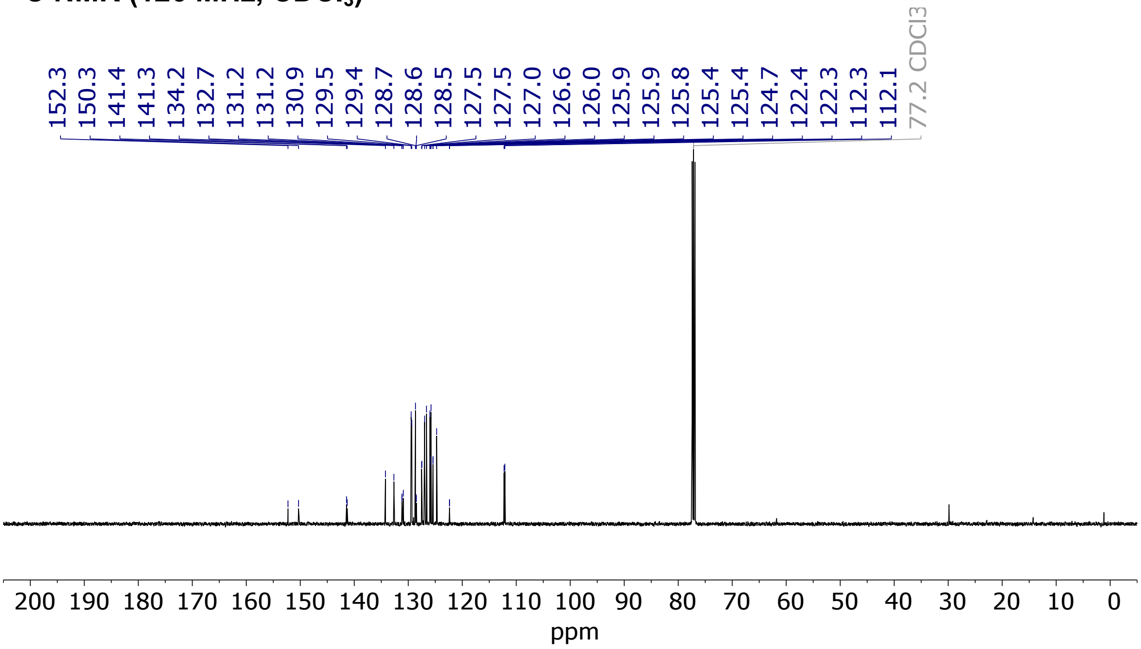

^{1}H NMR (300 MHz, $CDCl_3$)

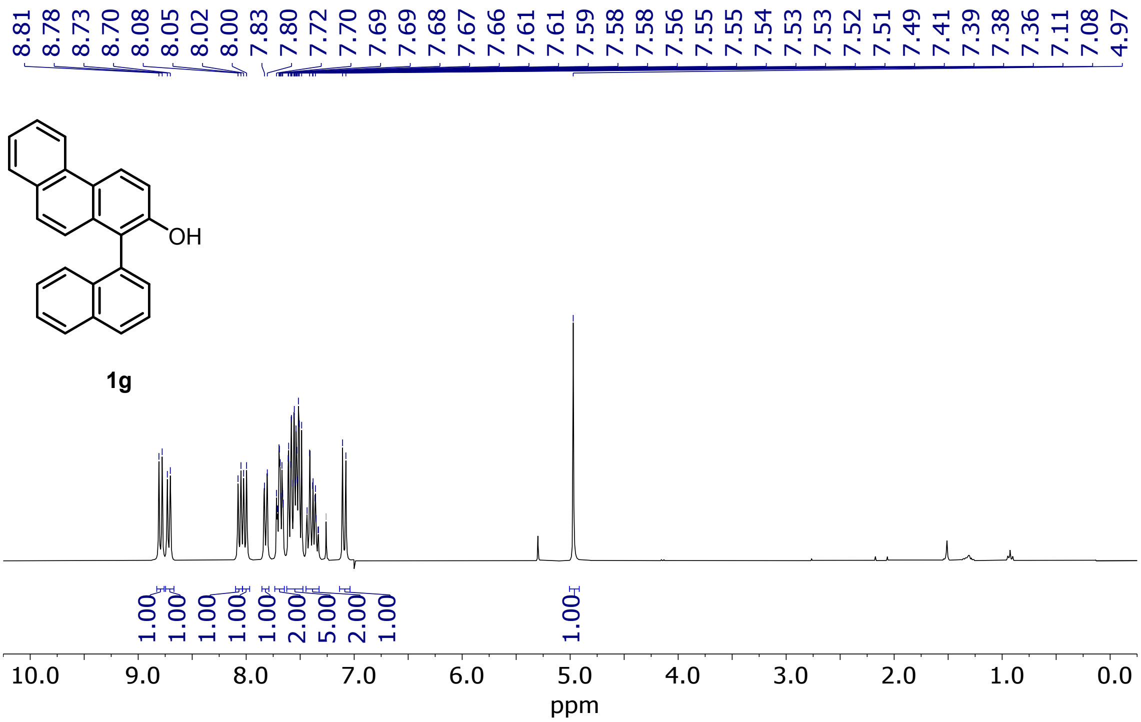

DEPT-135

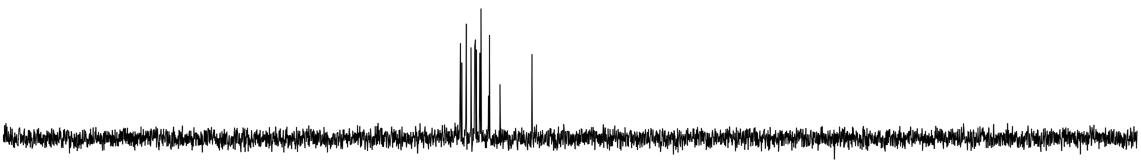

^{13}C NMR (75 MHz, $CDCl_3$)

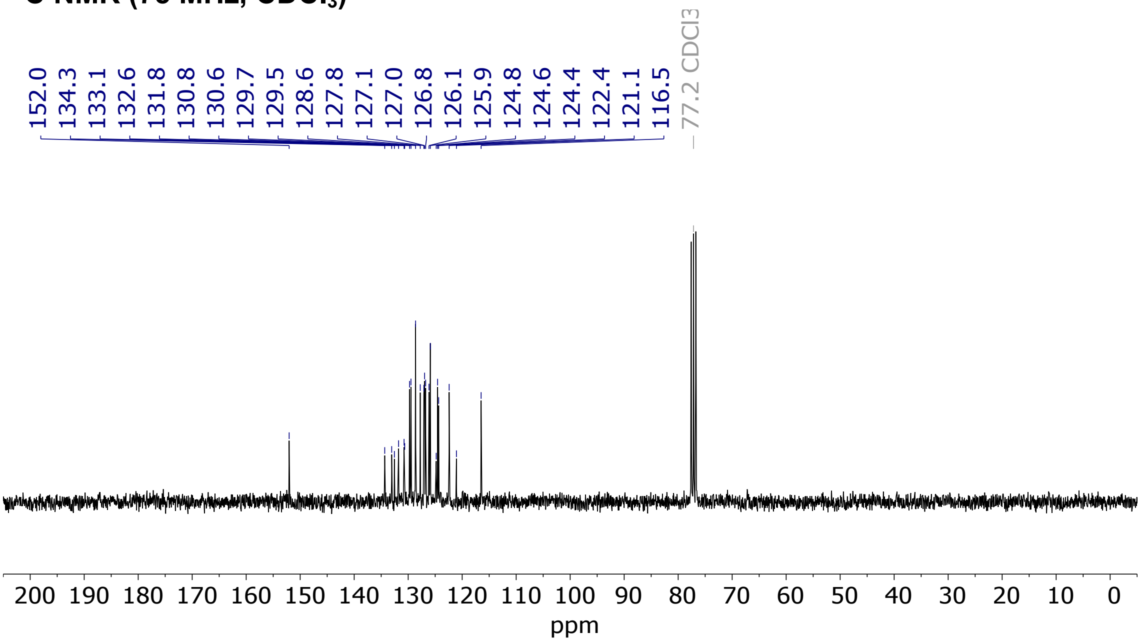

^{1}H NMR (500 MHz, $CDCl_3$)

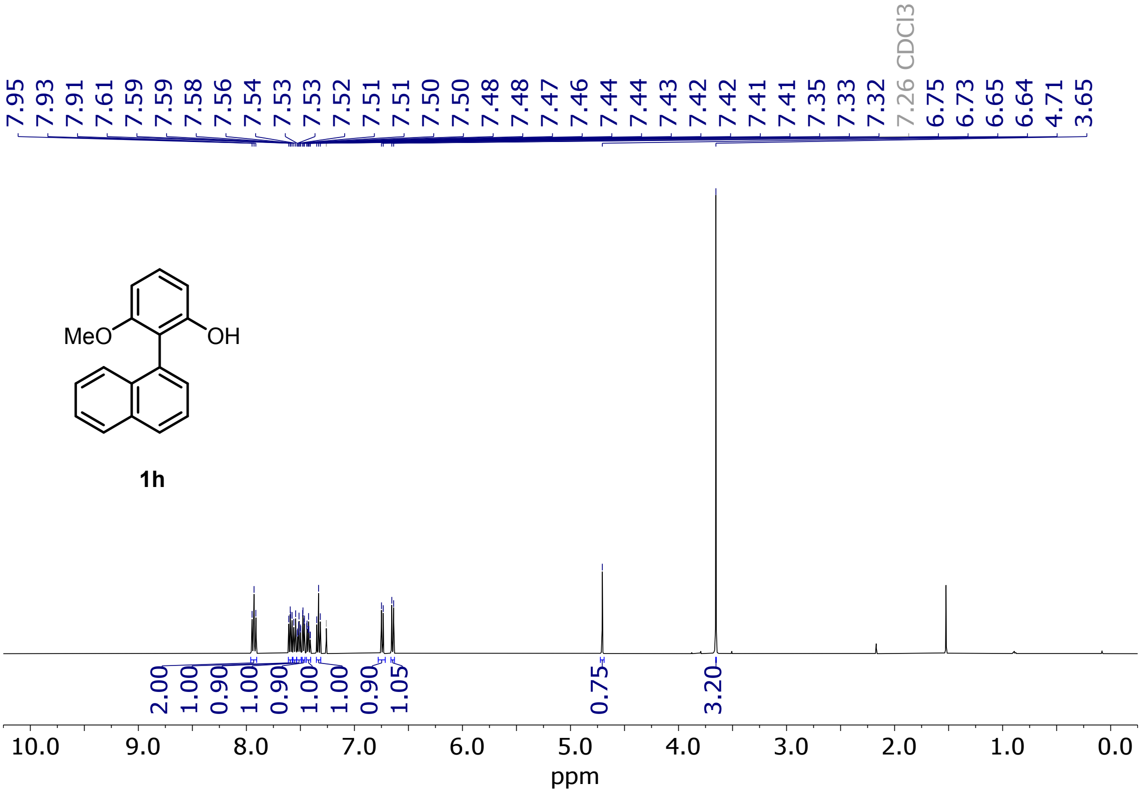

DEPT-135

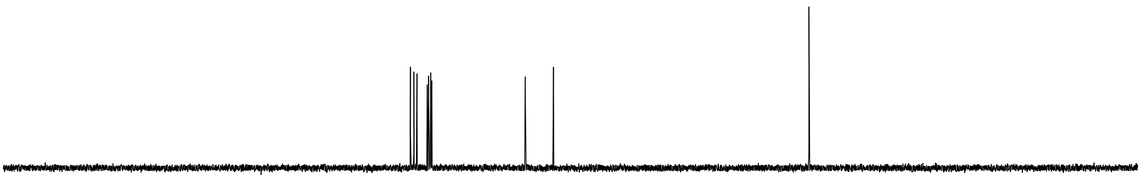

^{13}C NMR (126 MHz, $CDCl_3$)

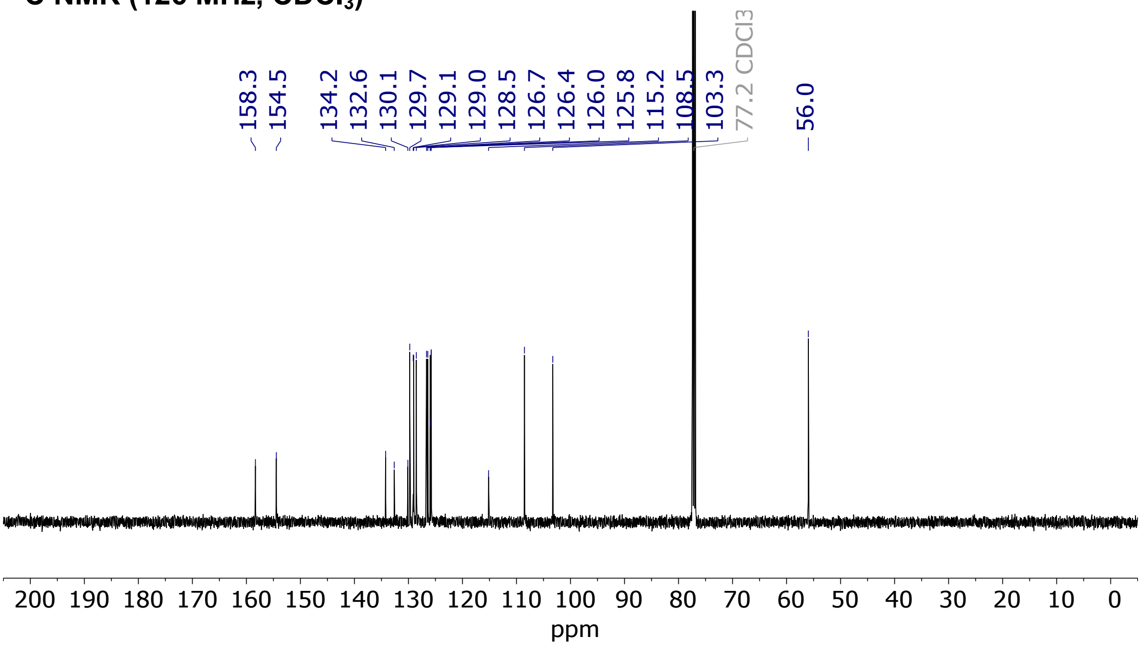

^{1}H NMR (300 MHz, CDCl$_3$)

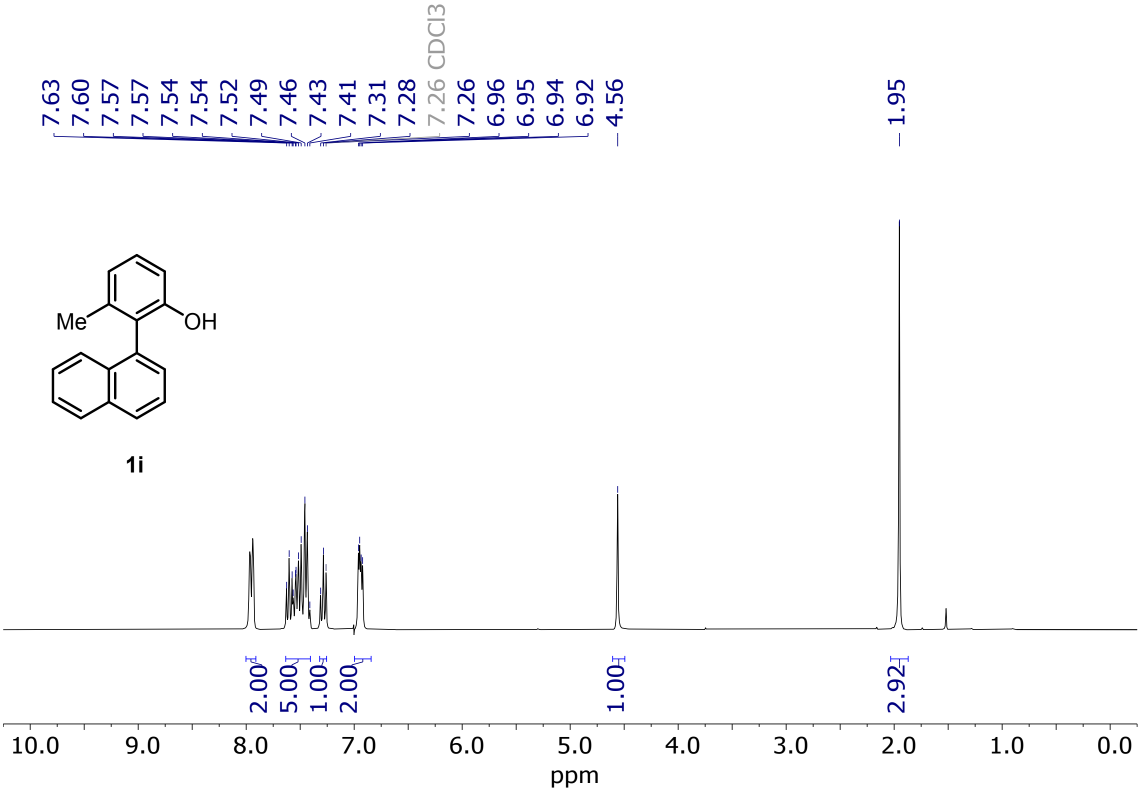

DEPT-135

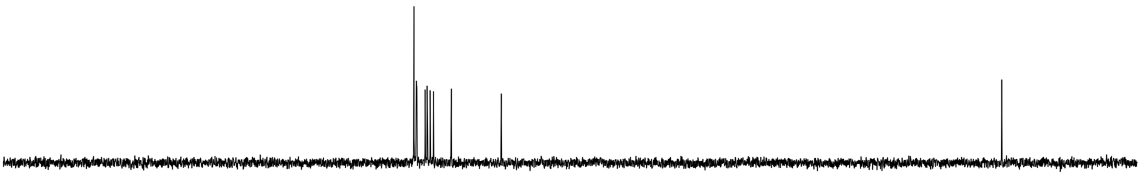

^{13}C NMR (75 MHz, CDCl$_3$)

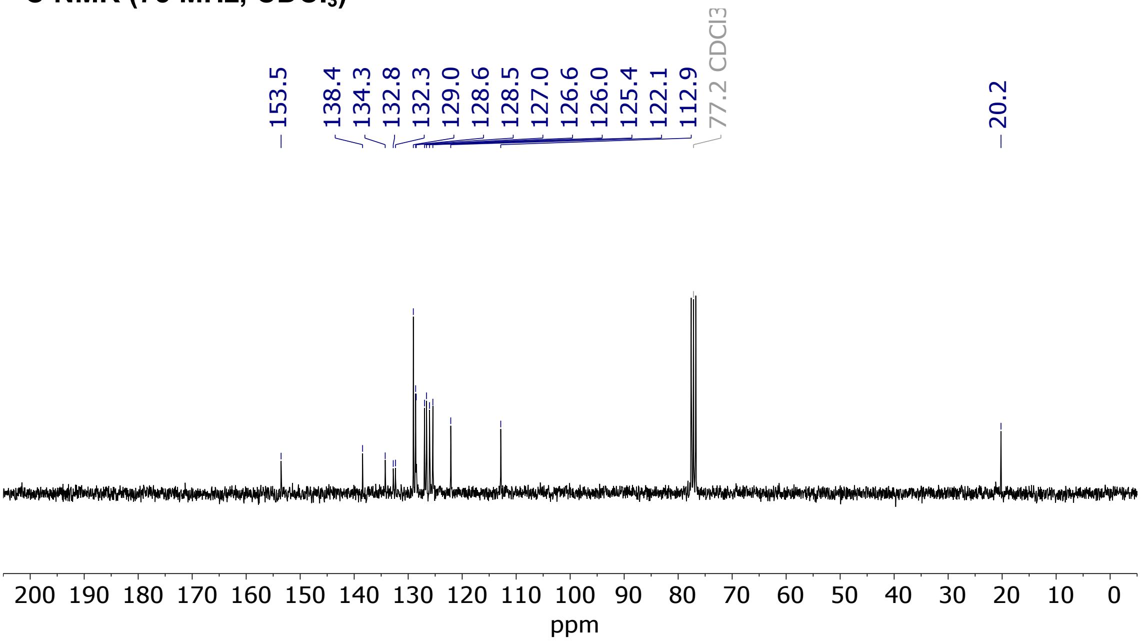

^{1}H NMR (300 MHz, CDCl$_3$)

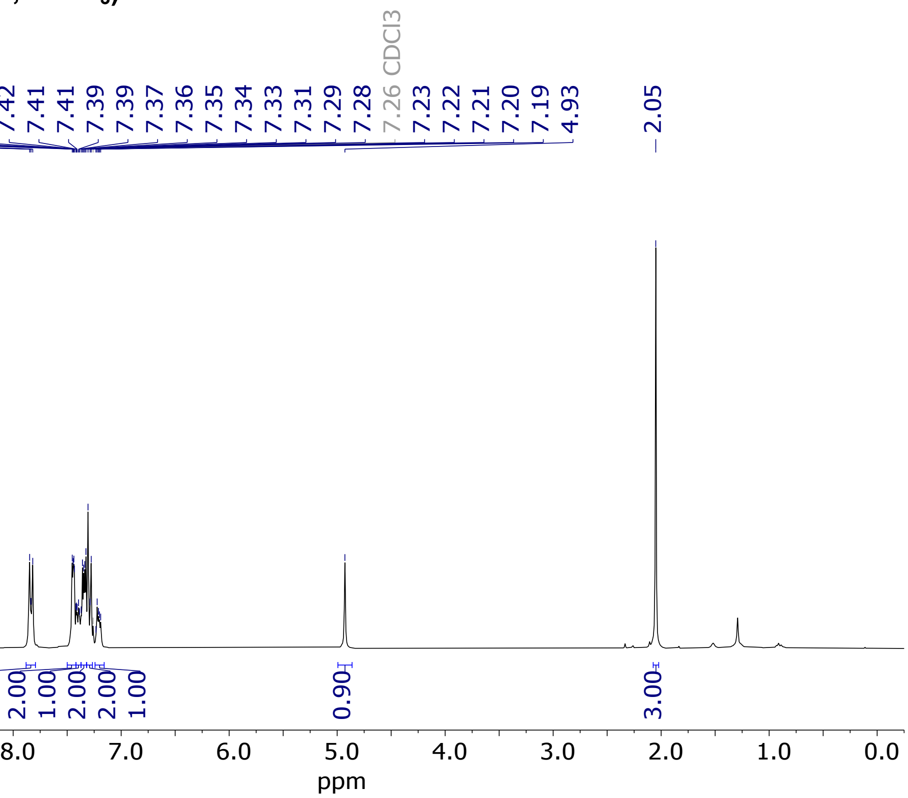

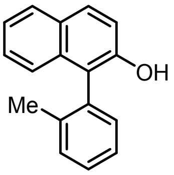

1j

DEPT-135

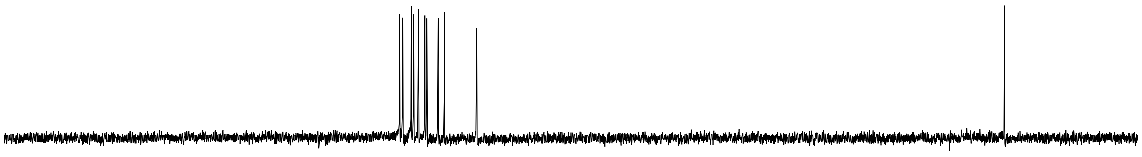

^{13}C NMR (75 MHz, CDCl$_3$)

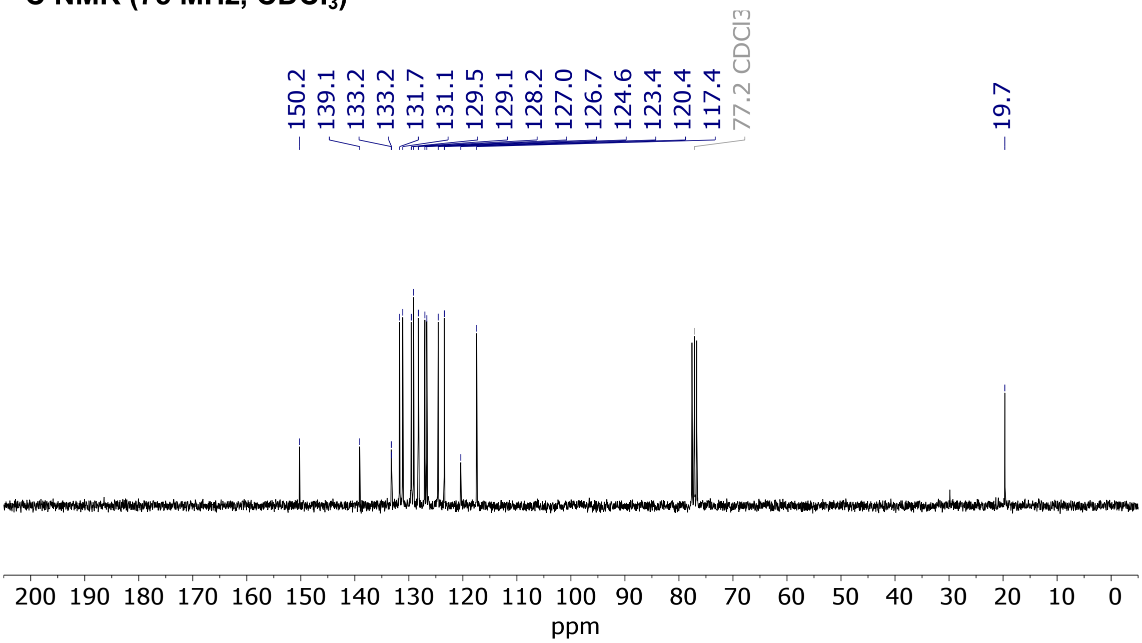

^{1}H NMR (300 MHz, $CDCl_3$)

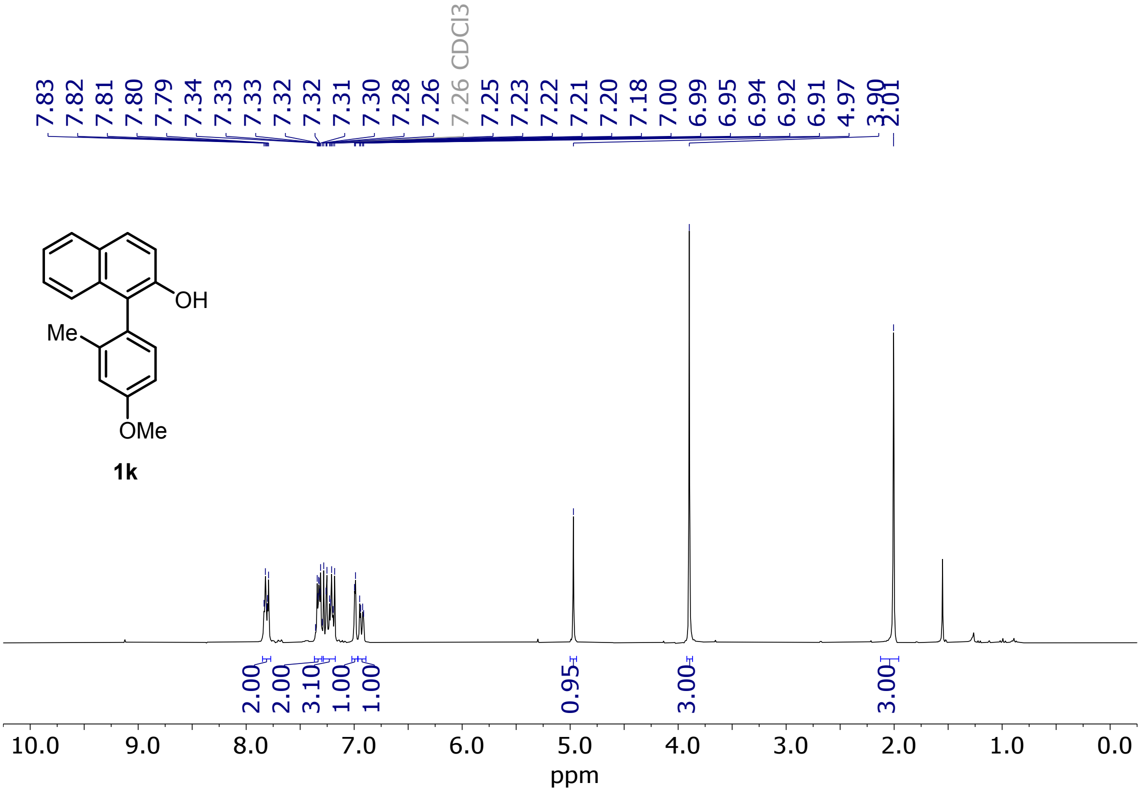

DEPT-135

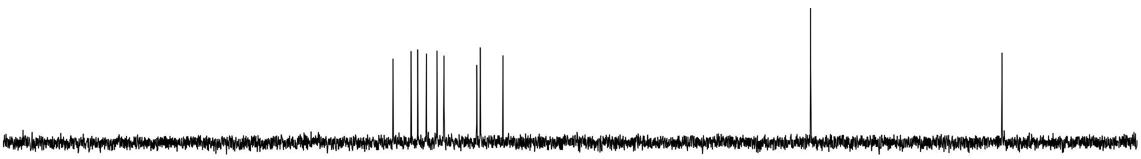

^{13}C NMR (75 MHz, $CDCl_3$)

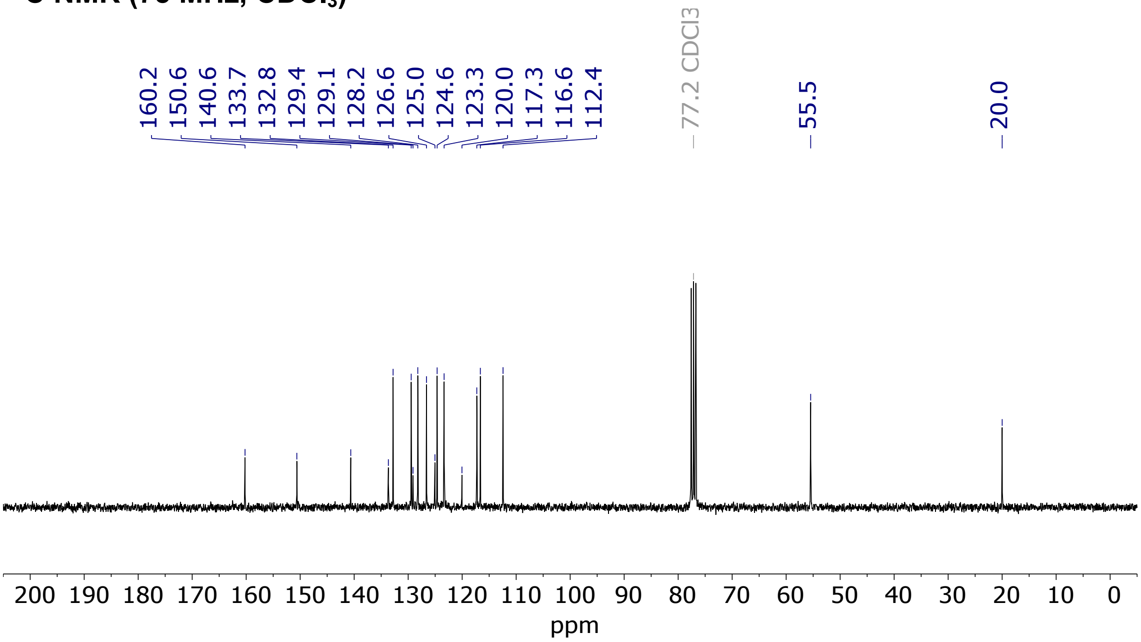

^{1}H NMR (300 MHz, $CDCl_3$)

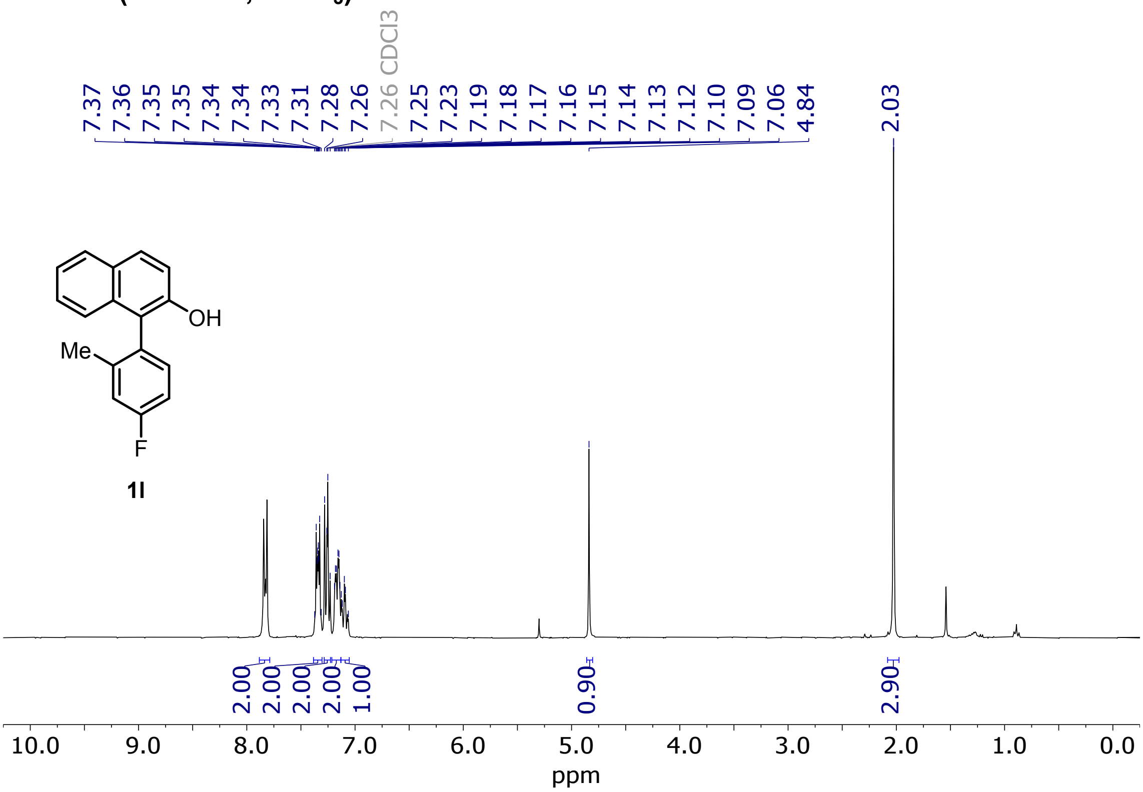

DEPT-135

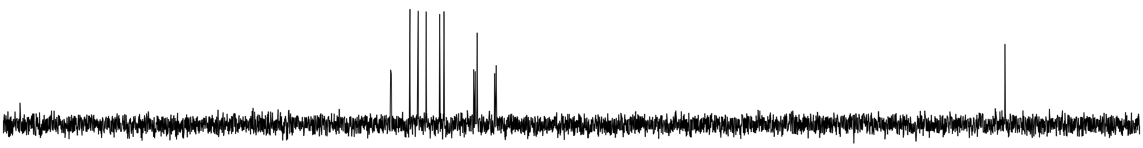

^{13}C NMR (75 MHz, $CDCl_3$)

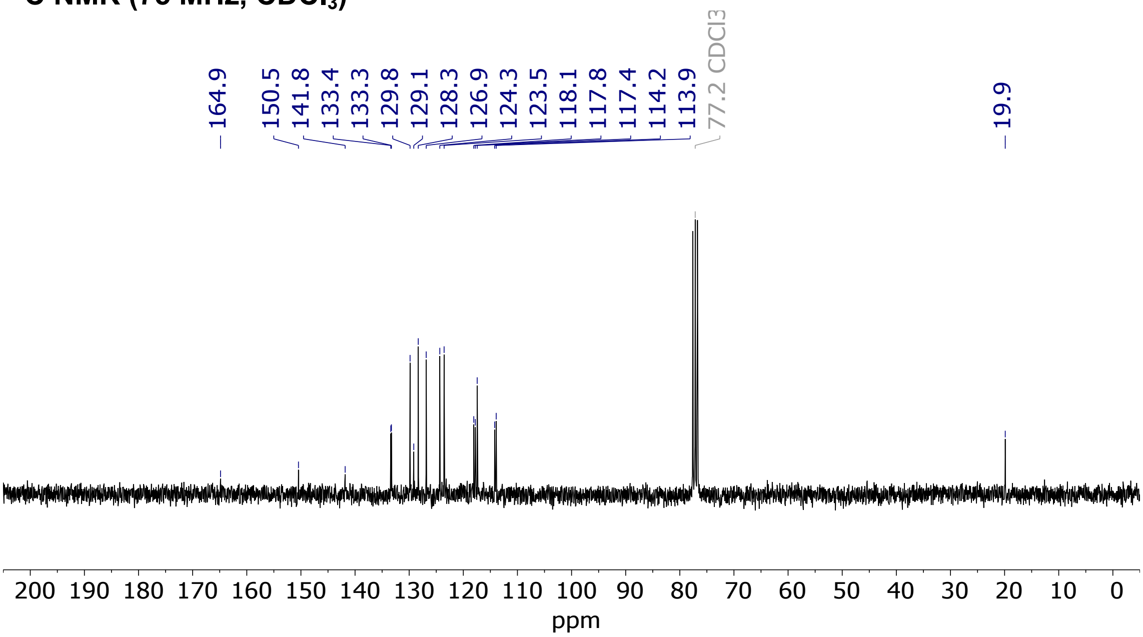

^{1}H NMR (500 MHz, $CDCl_3$)

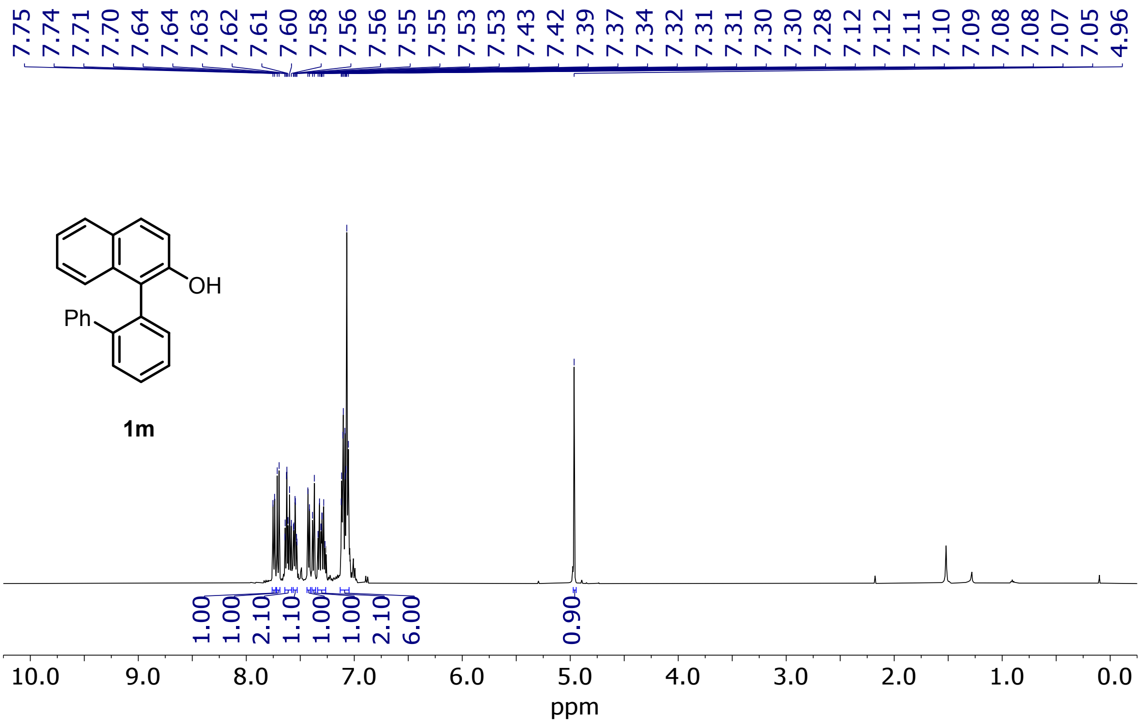

1m

DEPT-135

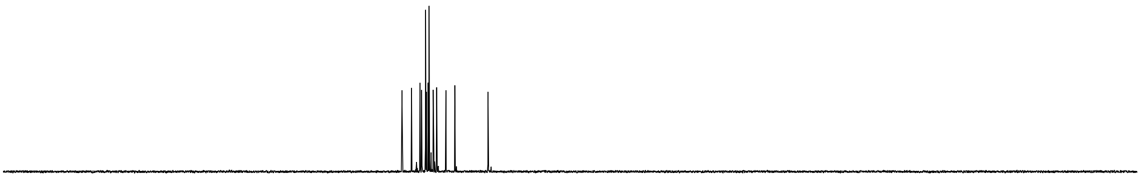

^{13}C NMR (126 MHz, $CDCl_3$)

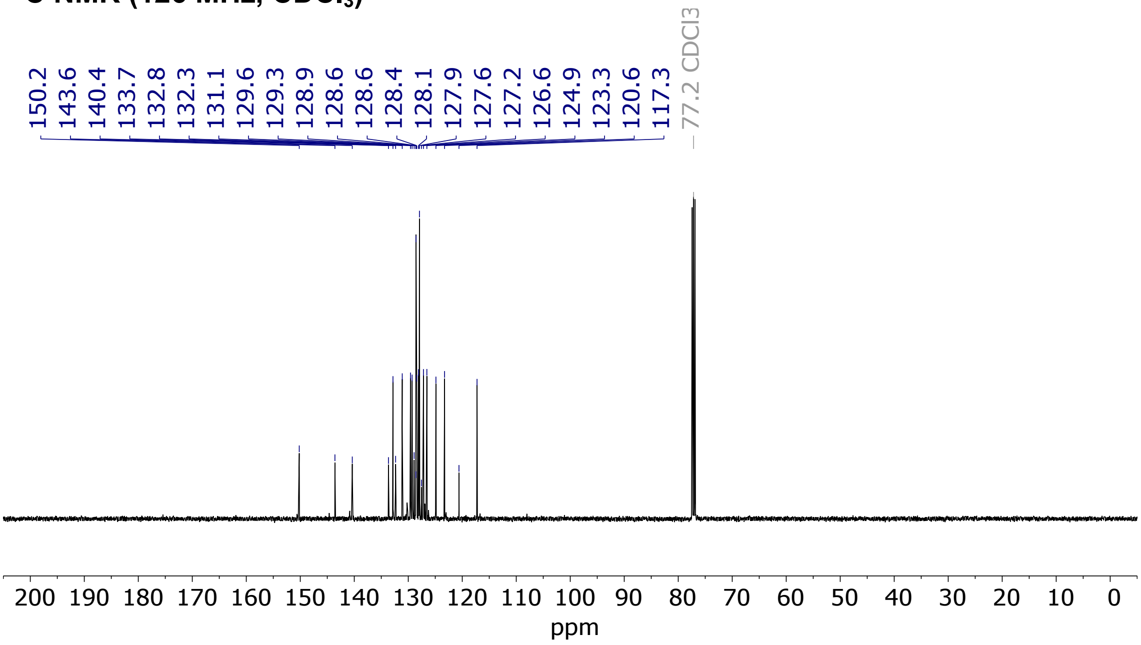

^{1}H NMR (300 MHz, $CDCl_3$)

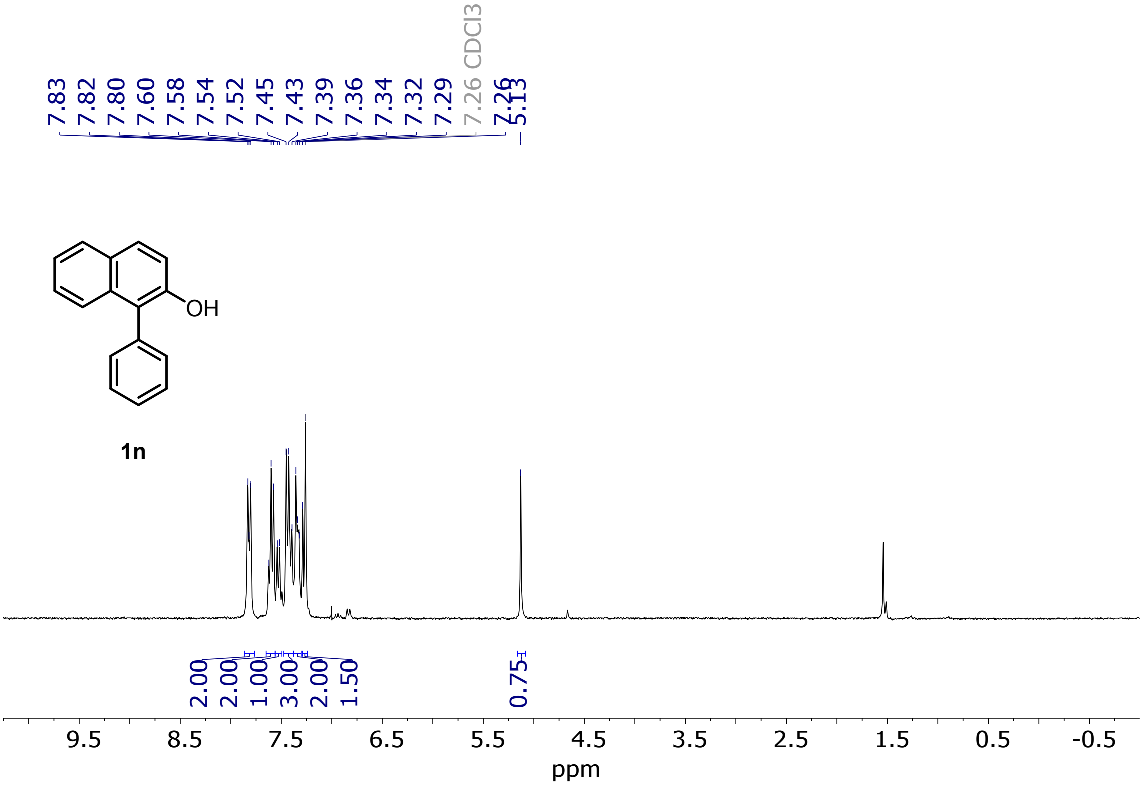

DEPT-135

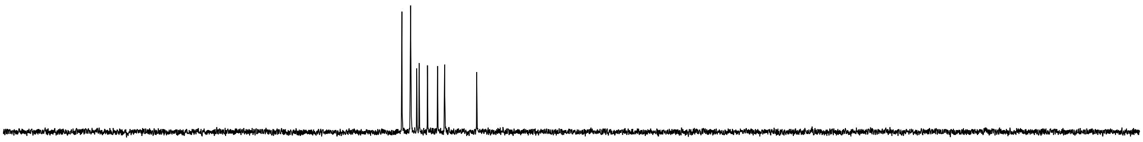

^{13}C NMR (75 MHz, $CDCl_3$)

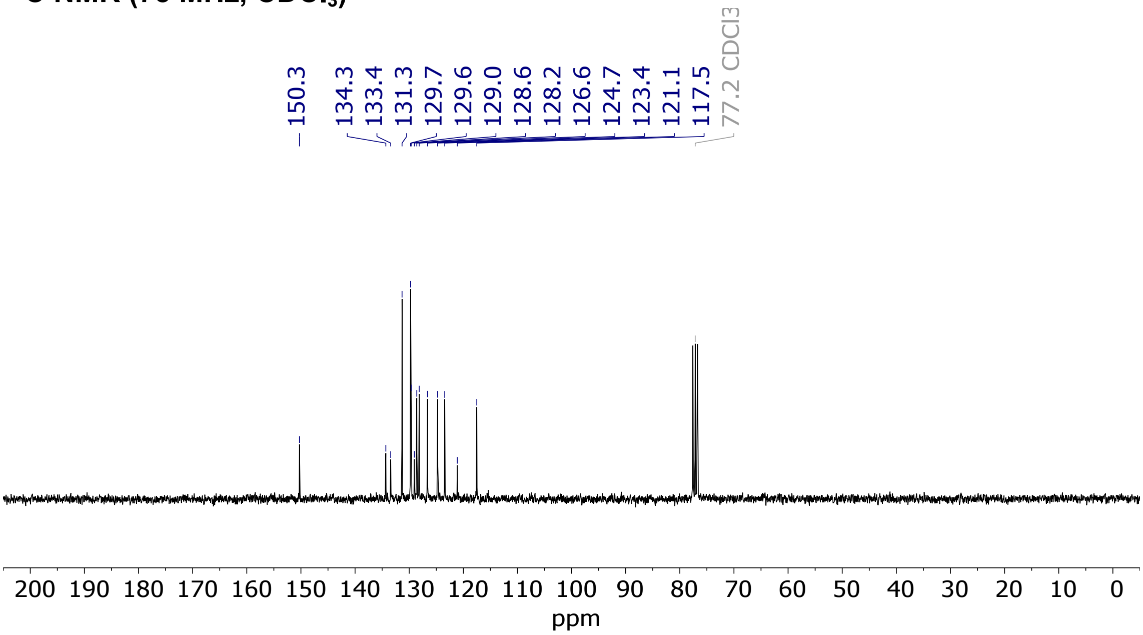

^{1}H NMR (300 MHz, $CDCl_3$)

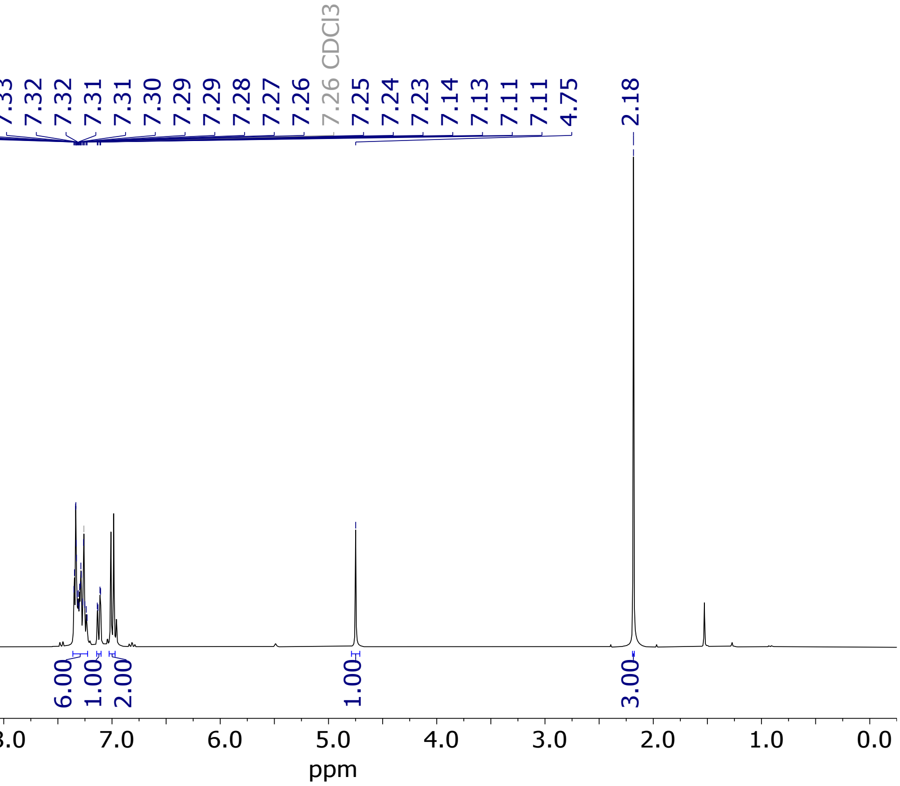

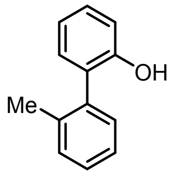

1o

DEPT-135

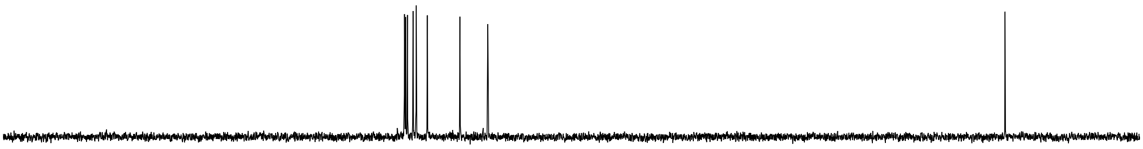

^{13}C NMR (75 MHz, $CDCl_3$)

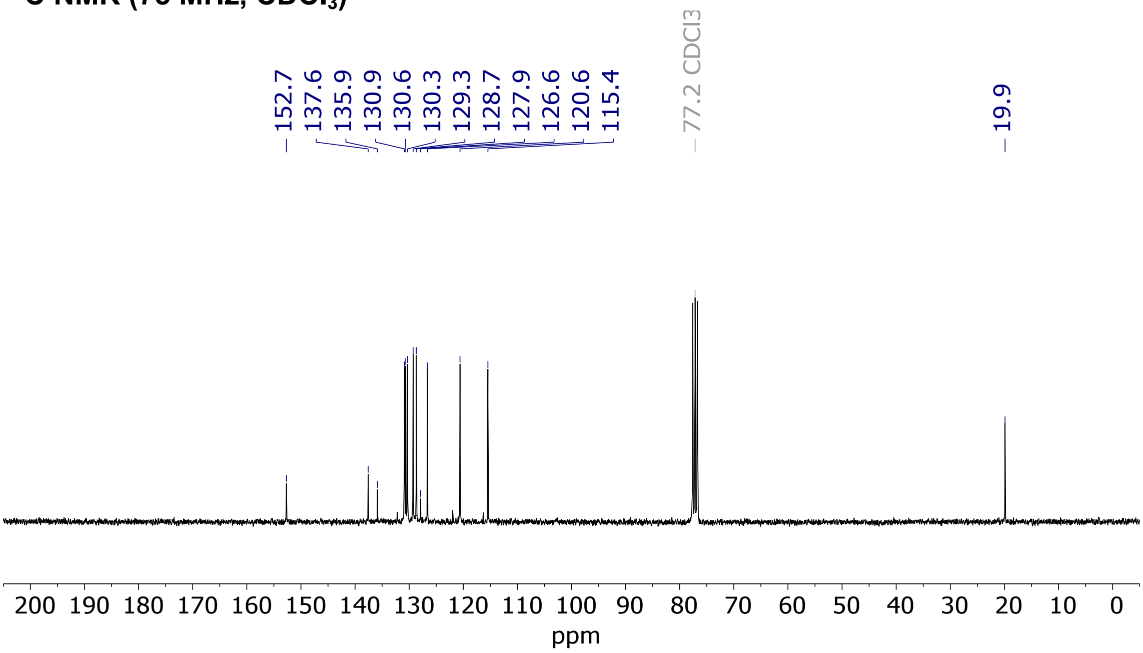

^{1}H NMR (500 MHz, $CDCl_3$)

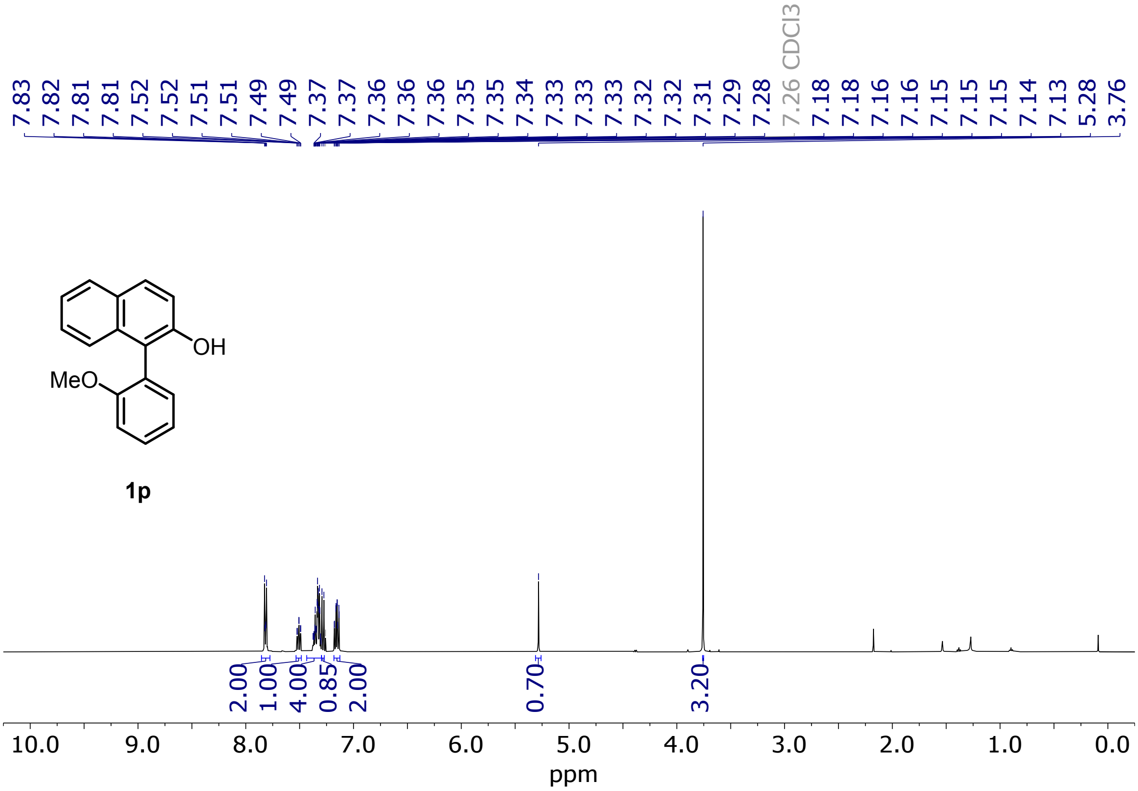

1p

DEPT-135

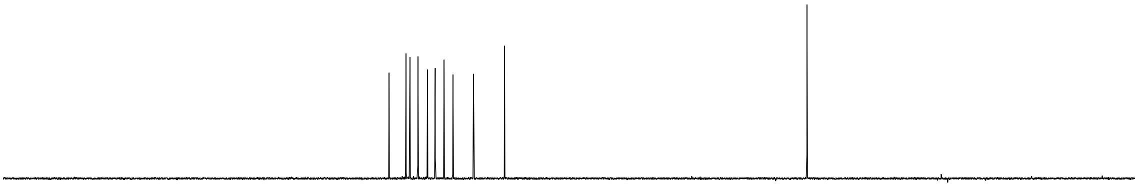

^{13}C NMR (126 MHz, $CDCl_3$)

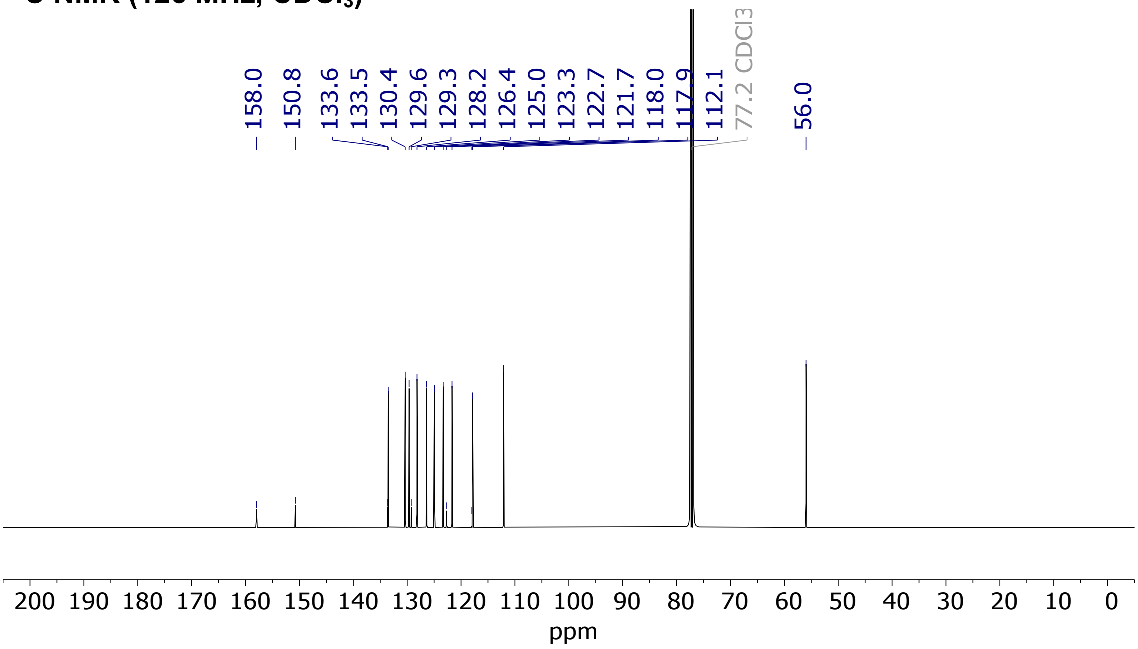

^{1}H NMR (500 MHz, $CDCl_3$)

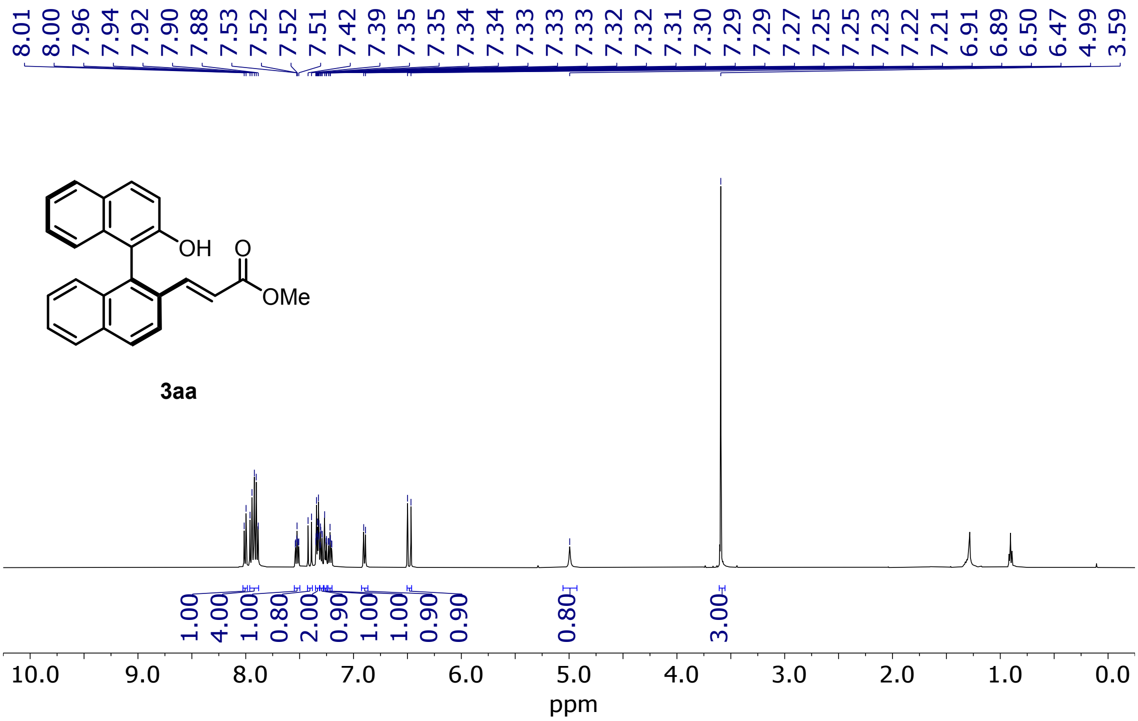

DEPT-135

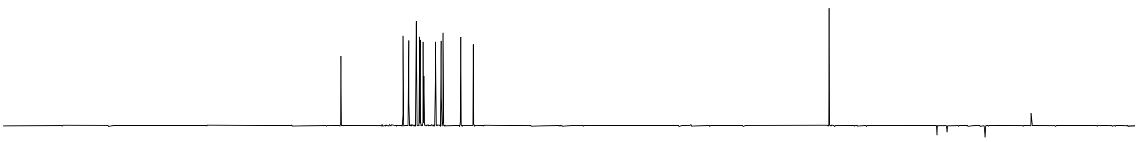

^{13}C NMR (126 MHz, $CDCl_3$)

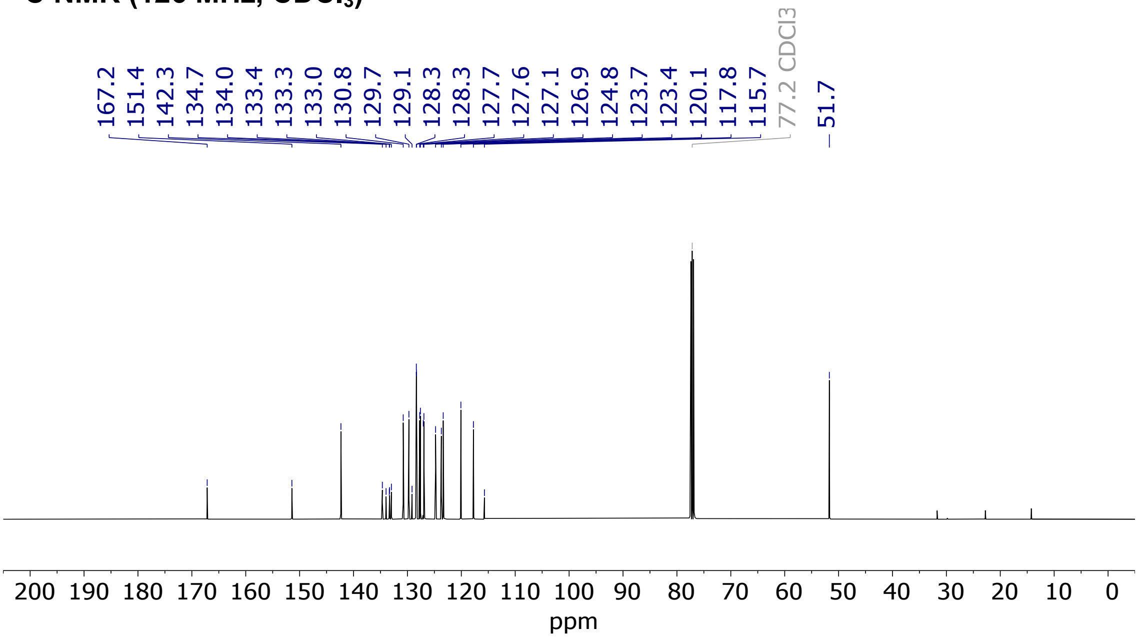

^{1}H NMR (500 MHz, $CDCl_3$)

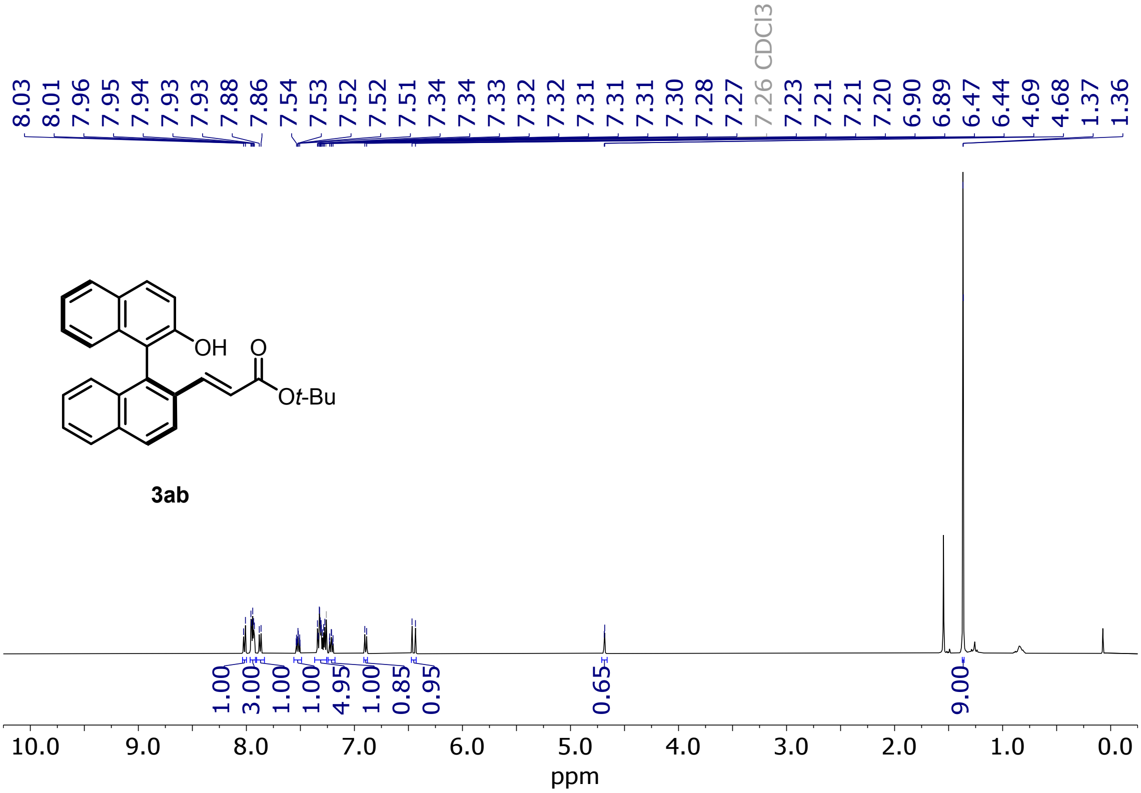

DEPT-135

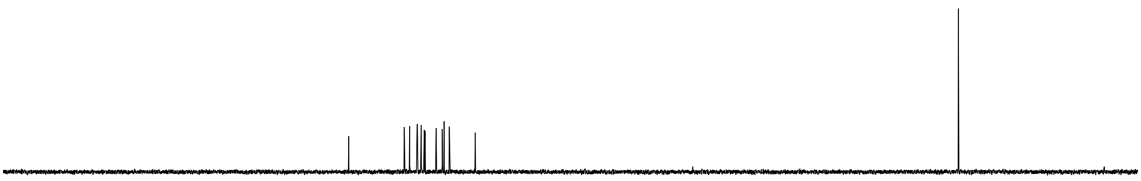

^{13}C NMR (126 MHz, $CDCl_3$)

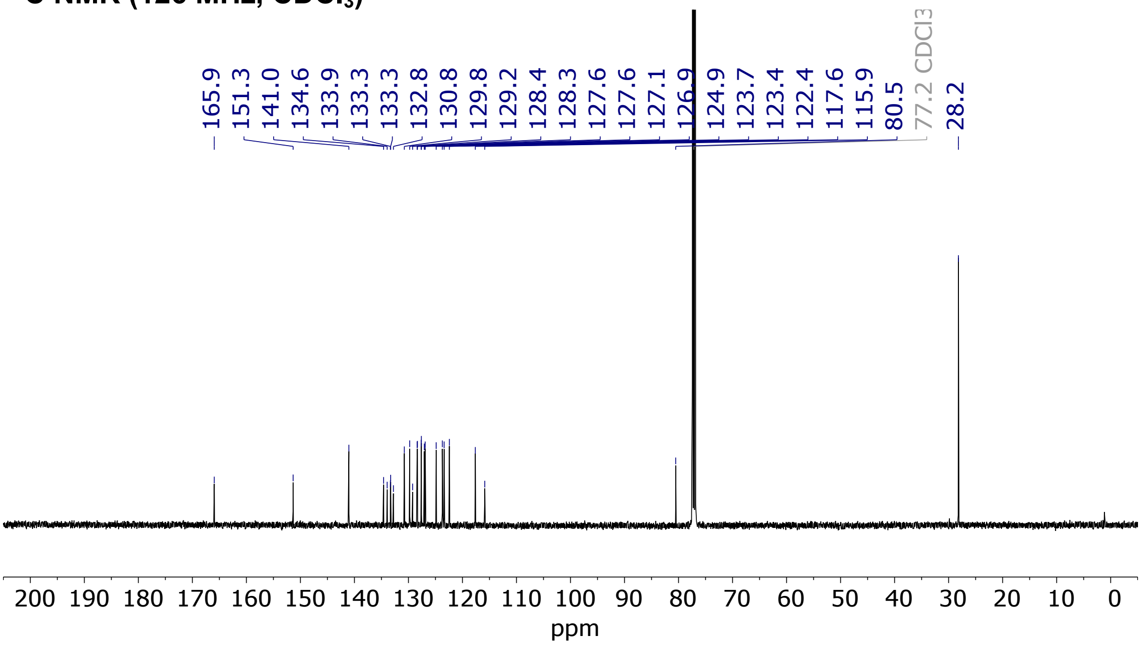

^{1}H NMR (300 MHz, CDCl$_3$)

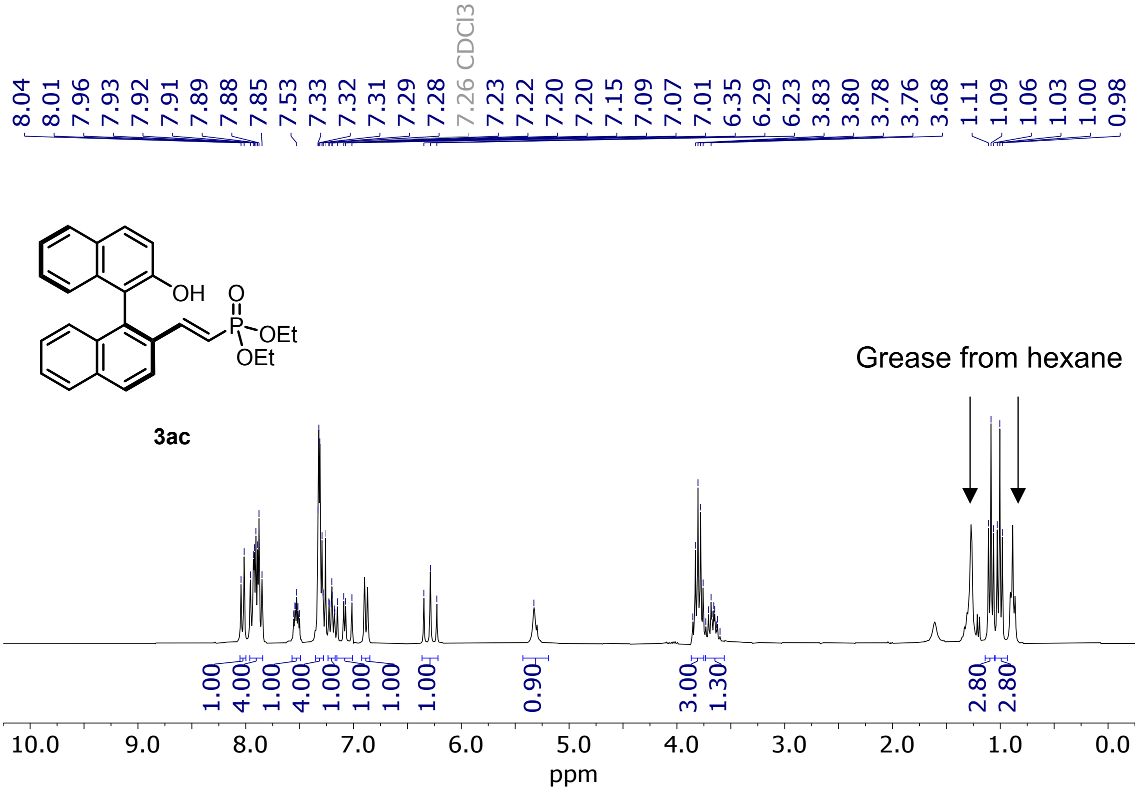

DEPT-135

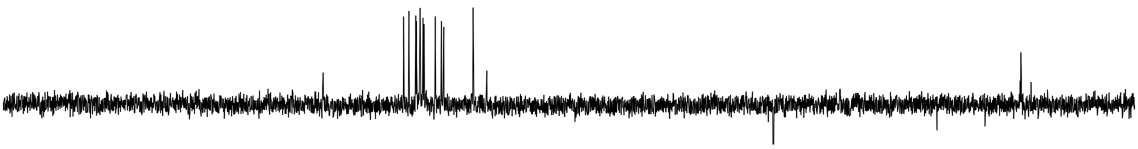

^{13}C NMR (75 MHz, CDCl$_3$)

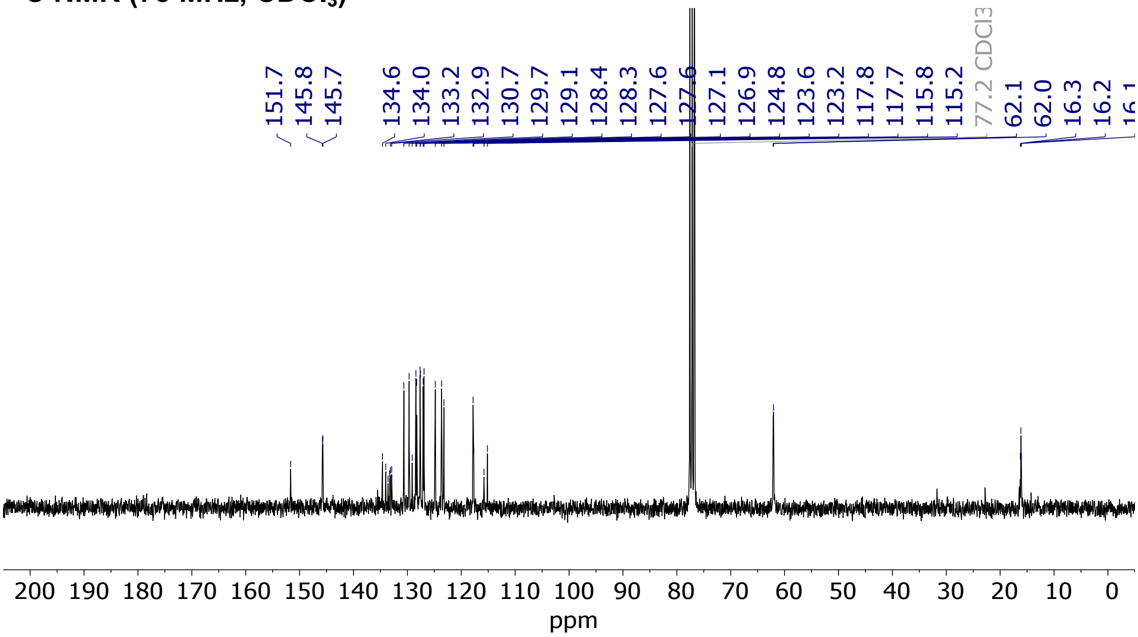

^{1}H NMR (300 MHz, $CDCl_3$)

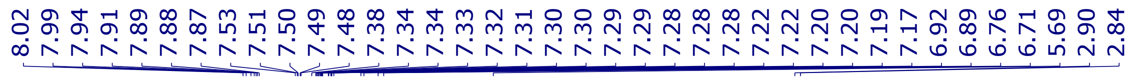

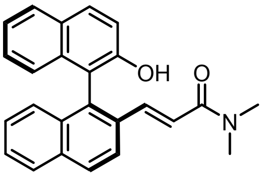

3ad

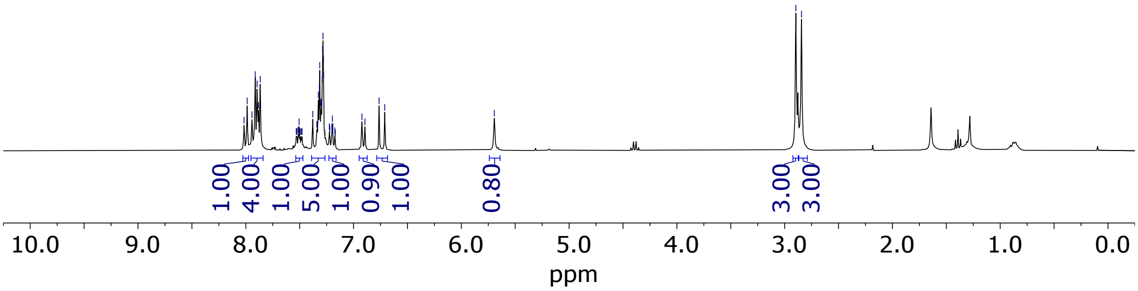

DEPT-135

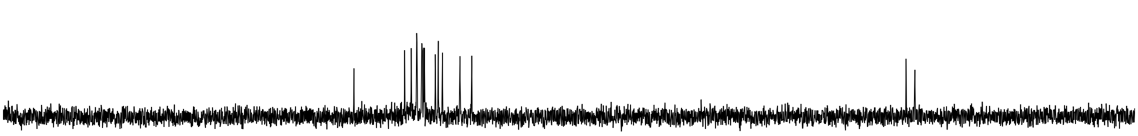

^{13}C NMR (75 MHz, $CDCl_3$)

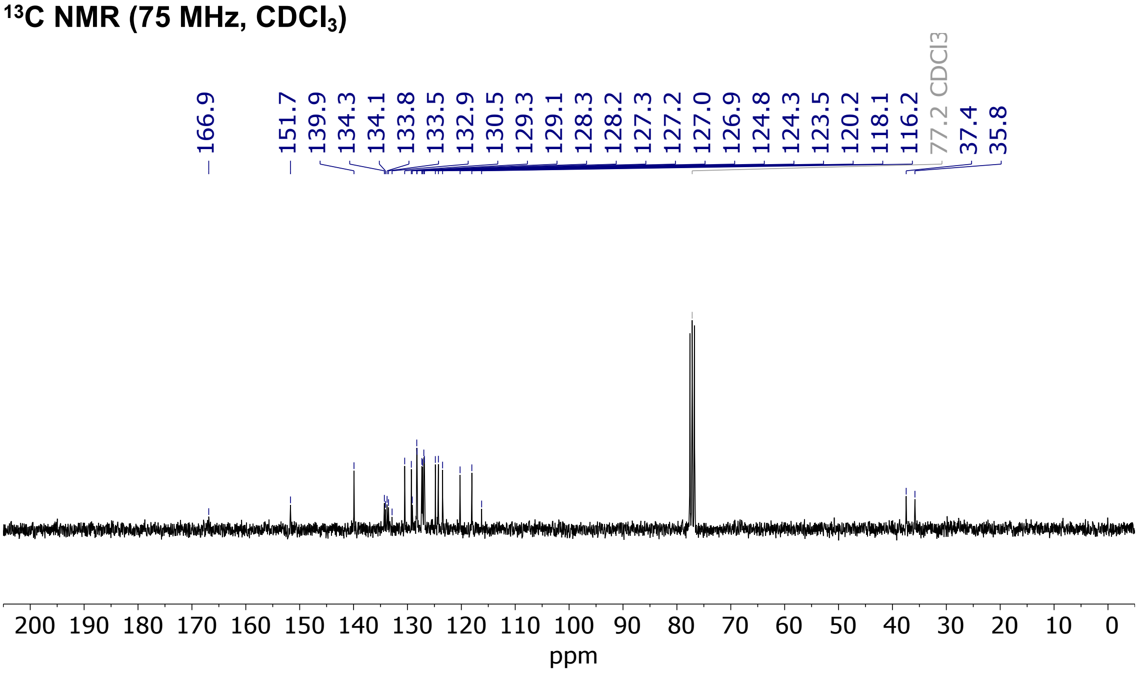

^{1}H NMR (300 MHz, $CDCl_3$)

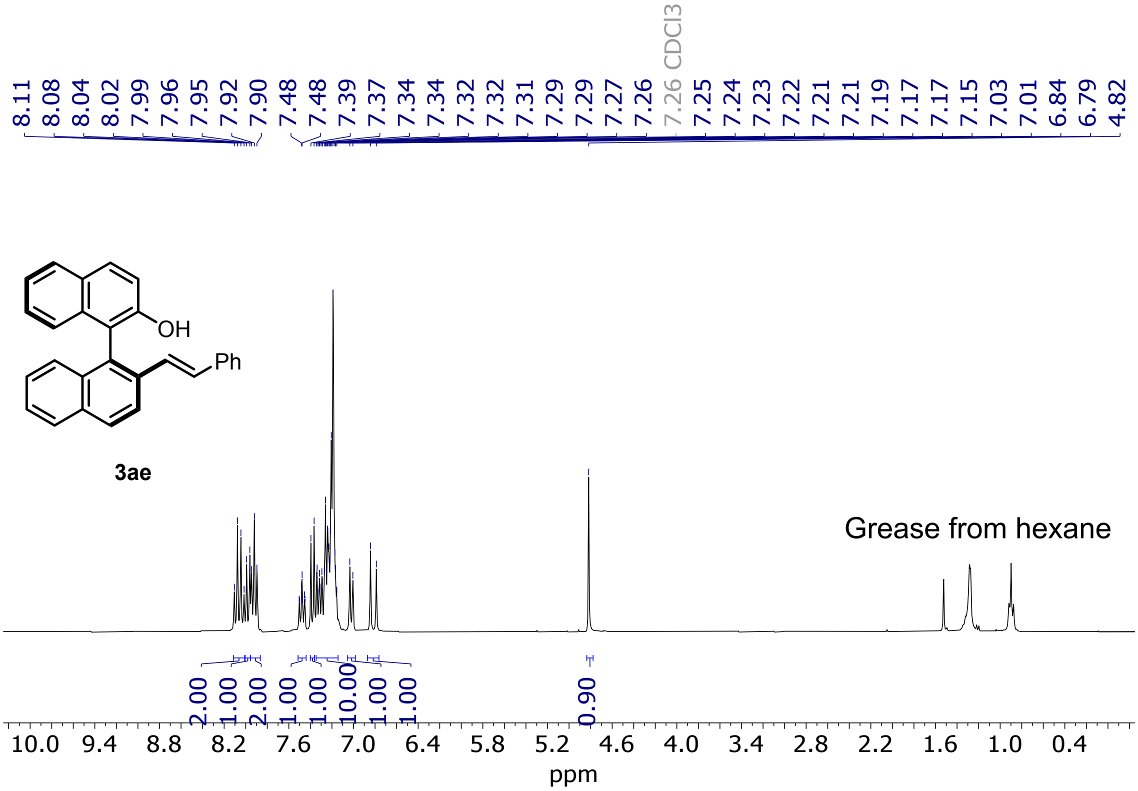

DEPT-135

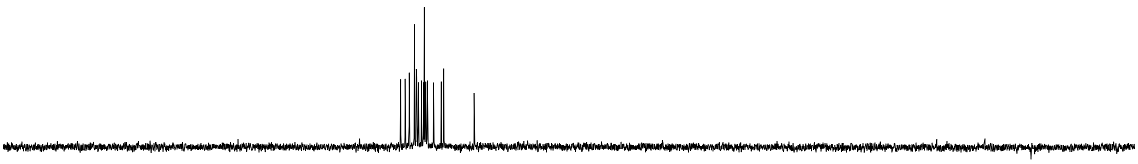

^{13}C NMR (75 MHz, $CDCl_3$)

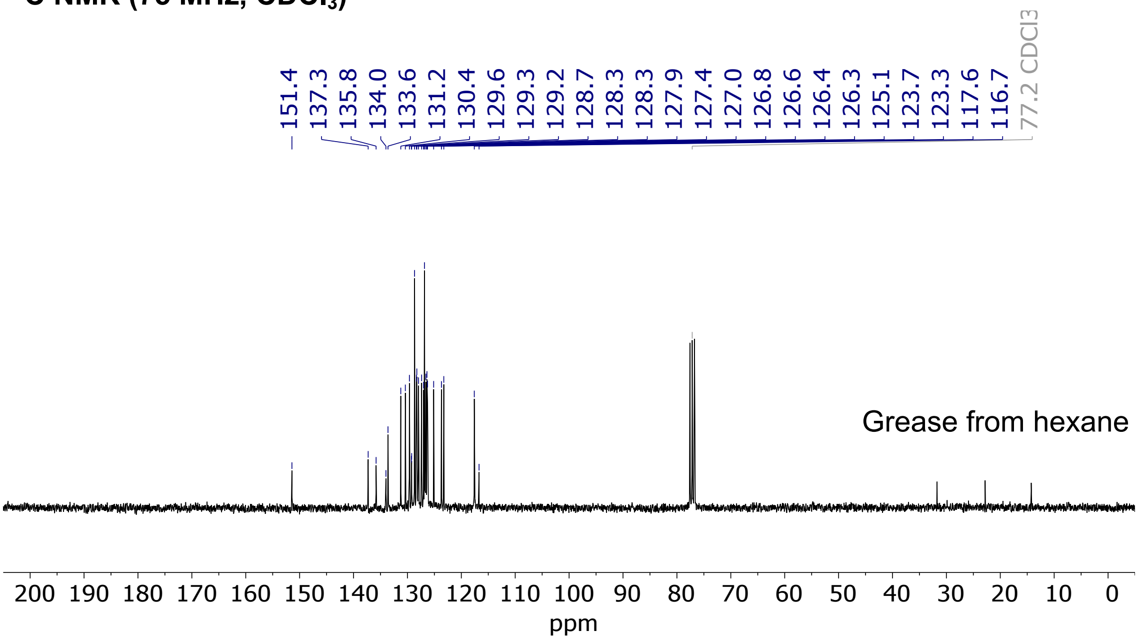

^{1}H NMR (300 MHz, $CDCl_3$)

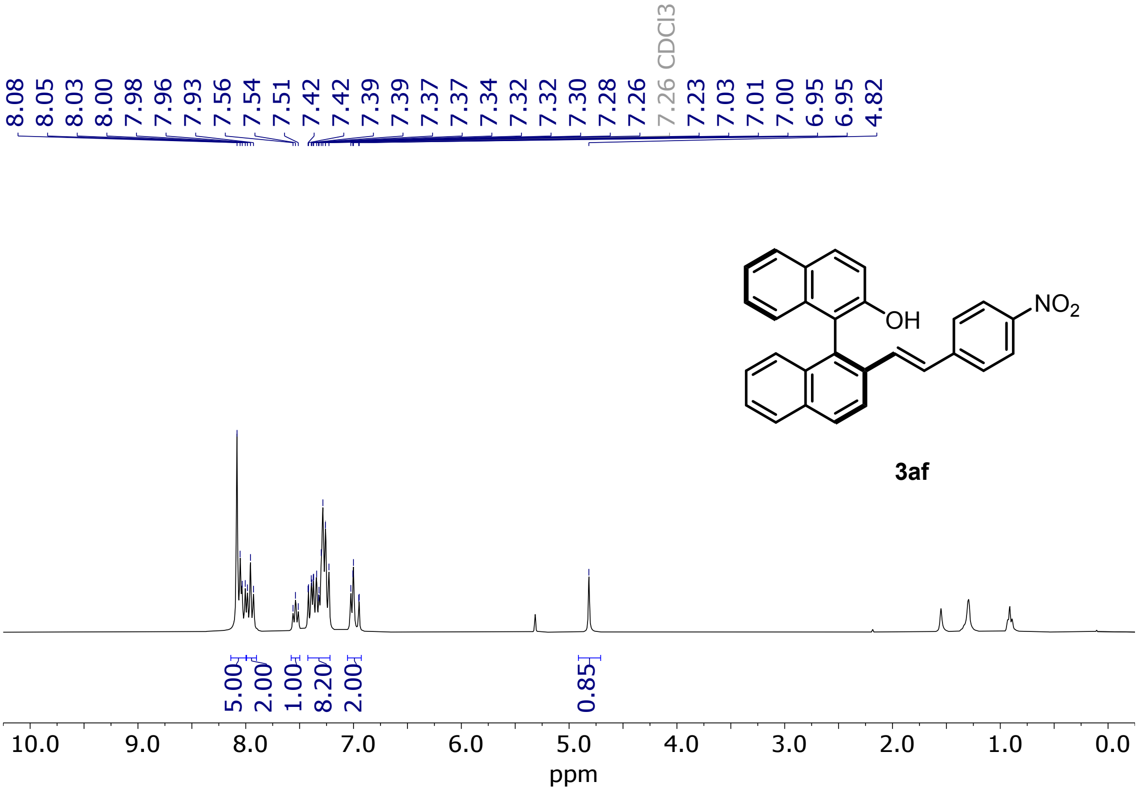

3af

DEPT-135

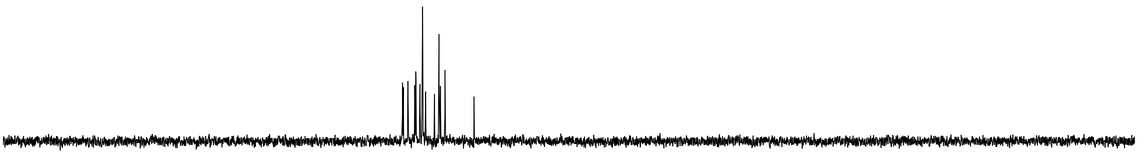

^{13}C NMR (75 MHz, $CDCl_3$)

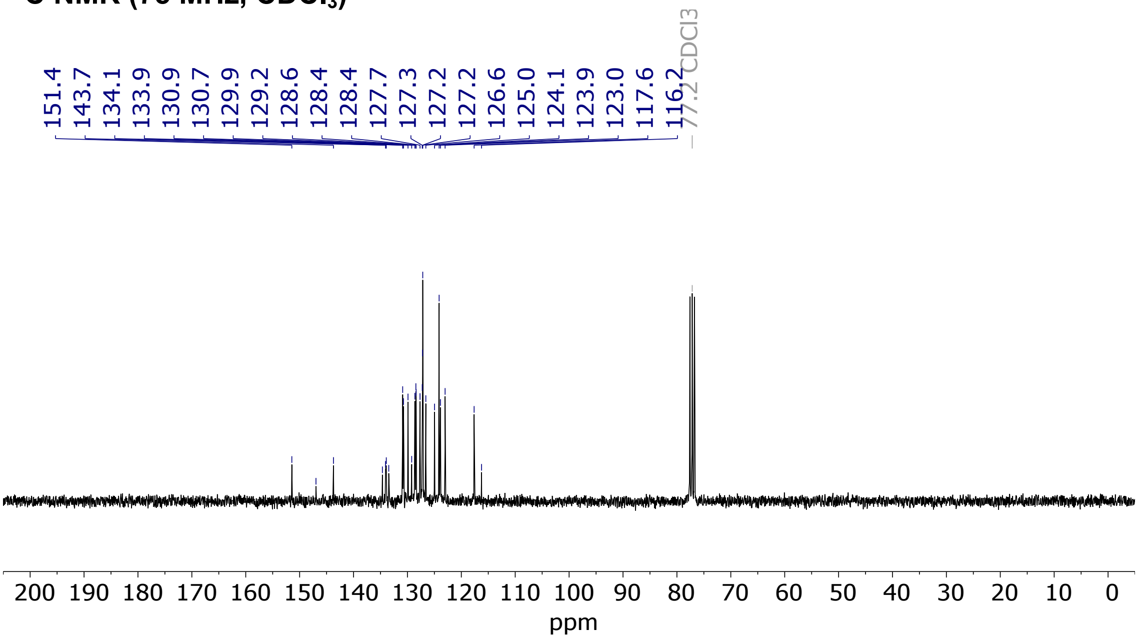

^{1}H NMR (300 MHz, $CDCl_3$)

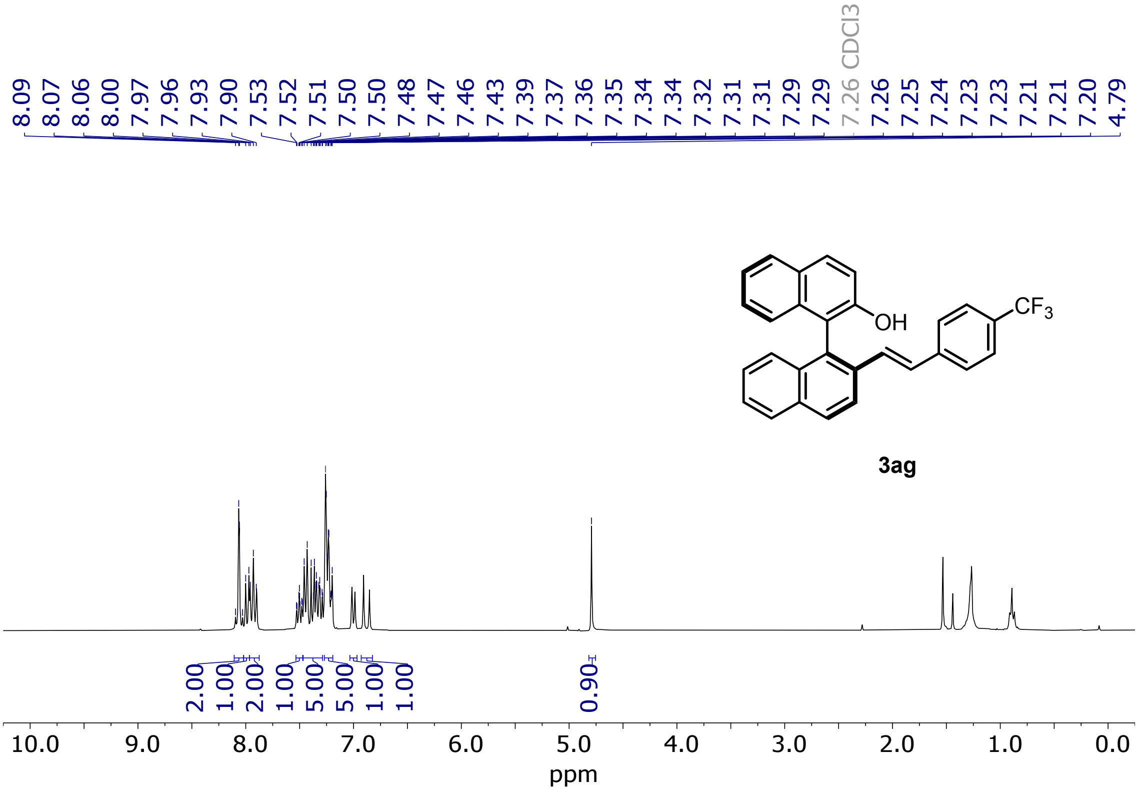

DEPT-135

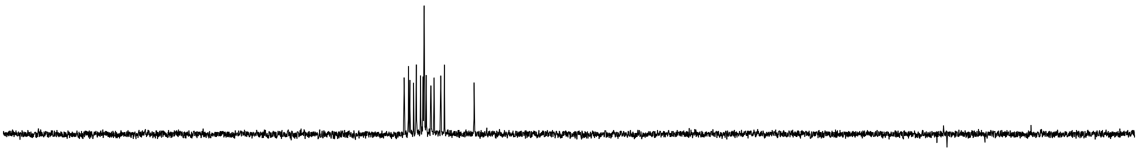

^{13}C NMR (75 MHz, $CDCl_3$)

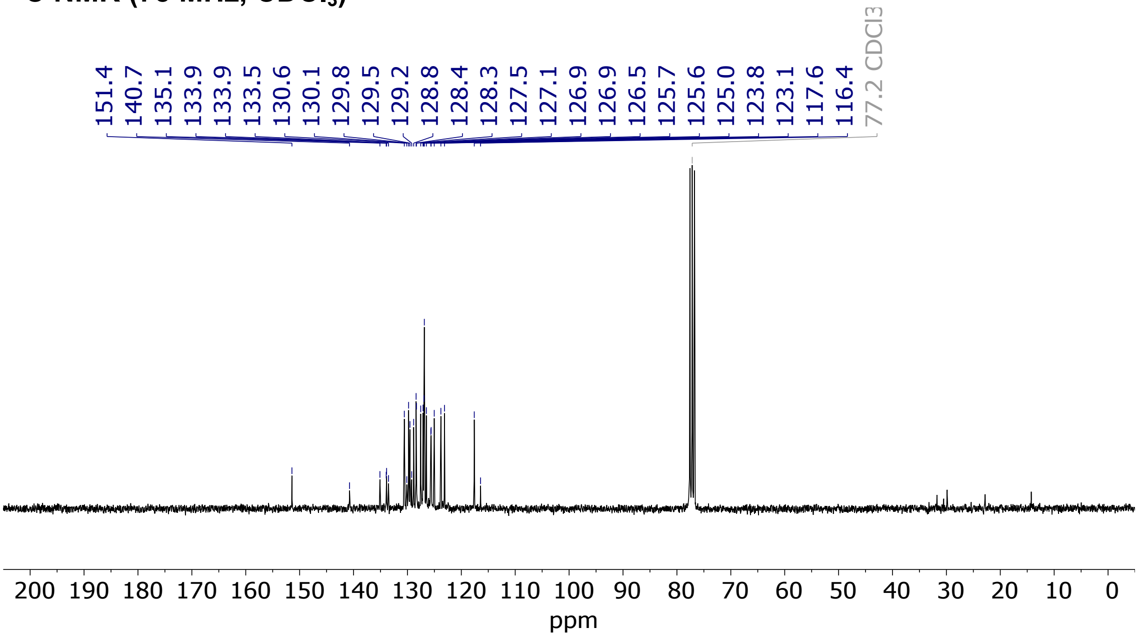

^{1}H NMR (300 MHz, CDCl$_3$)

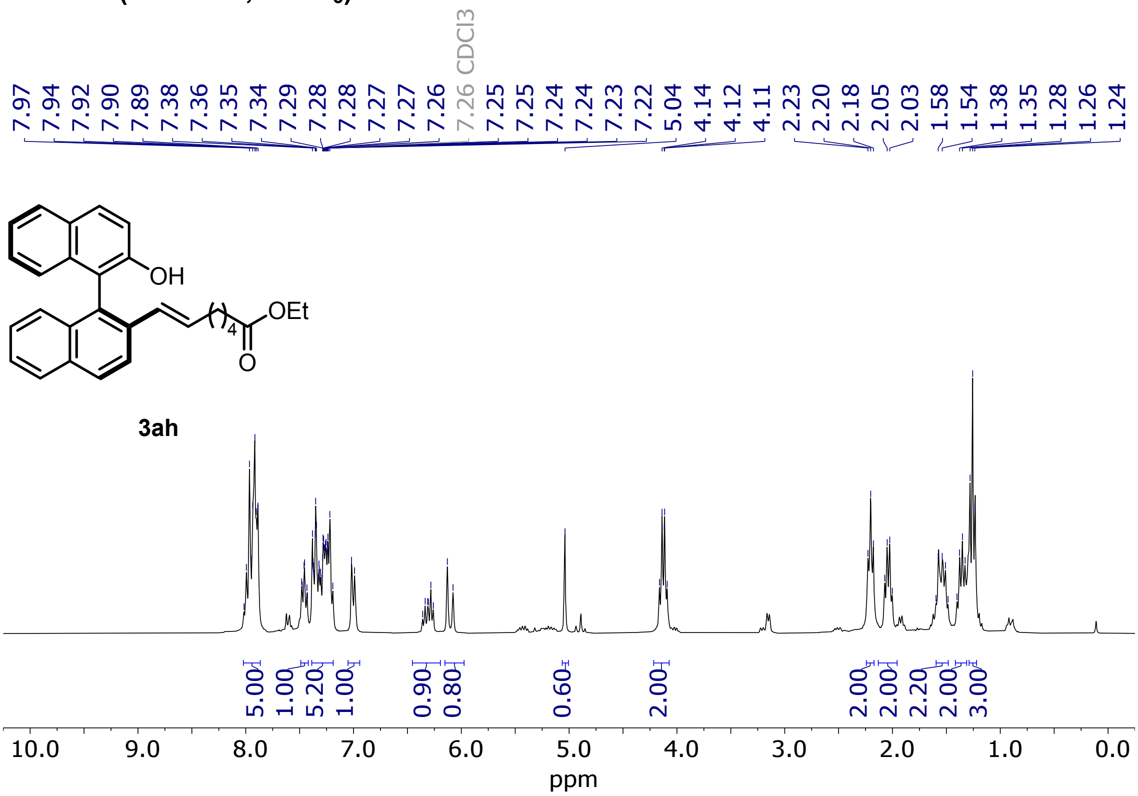

DEPT-135

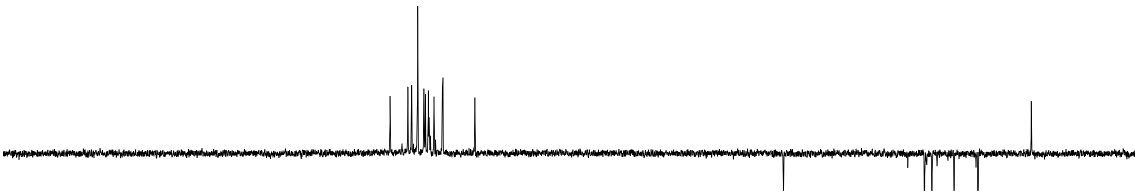

^{13}C NMR (75 MHz, CDCl$_3$)

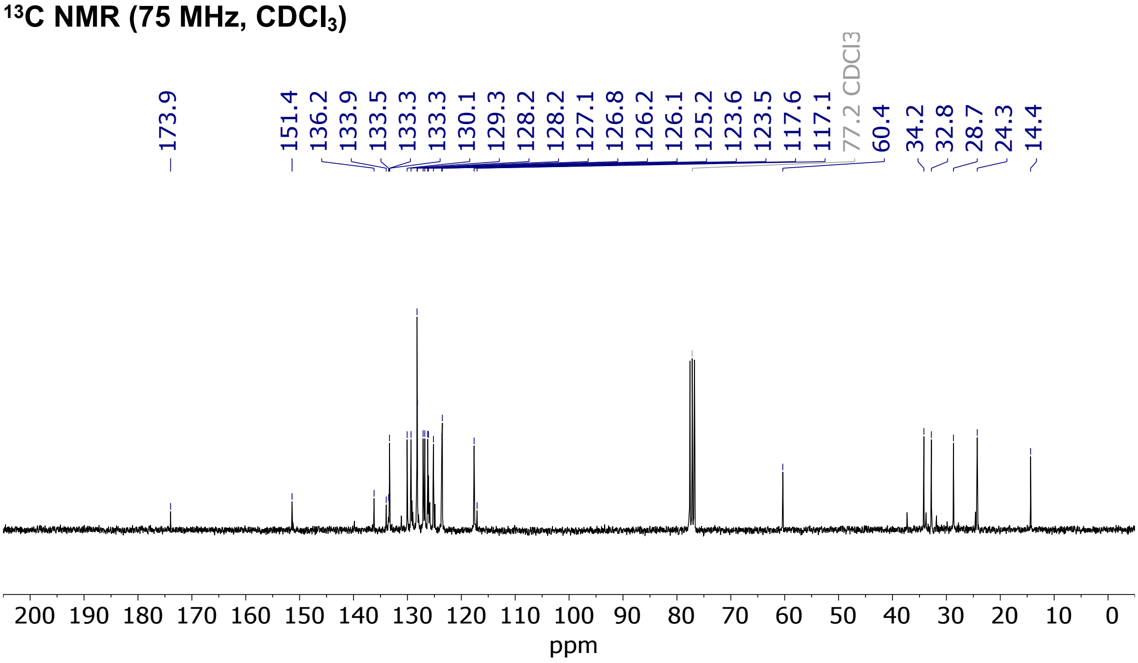

^{1}H NMR (300 MHz, $CDCl_3$)

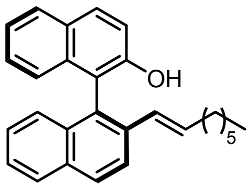

3ai

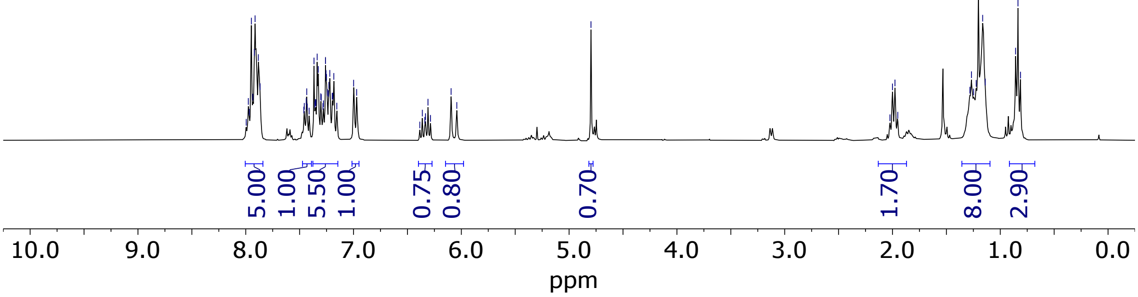

DEPT-135

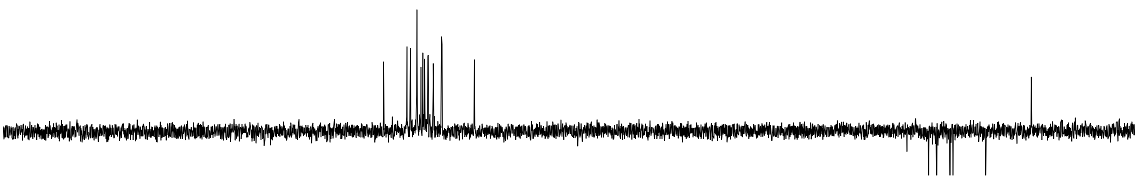

^{13}C NMR (75 MHz, $CDCl_3$)

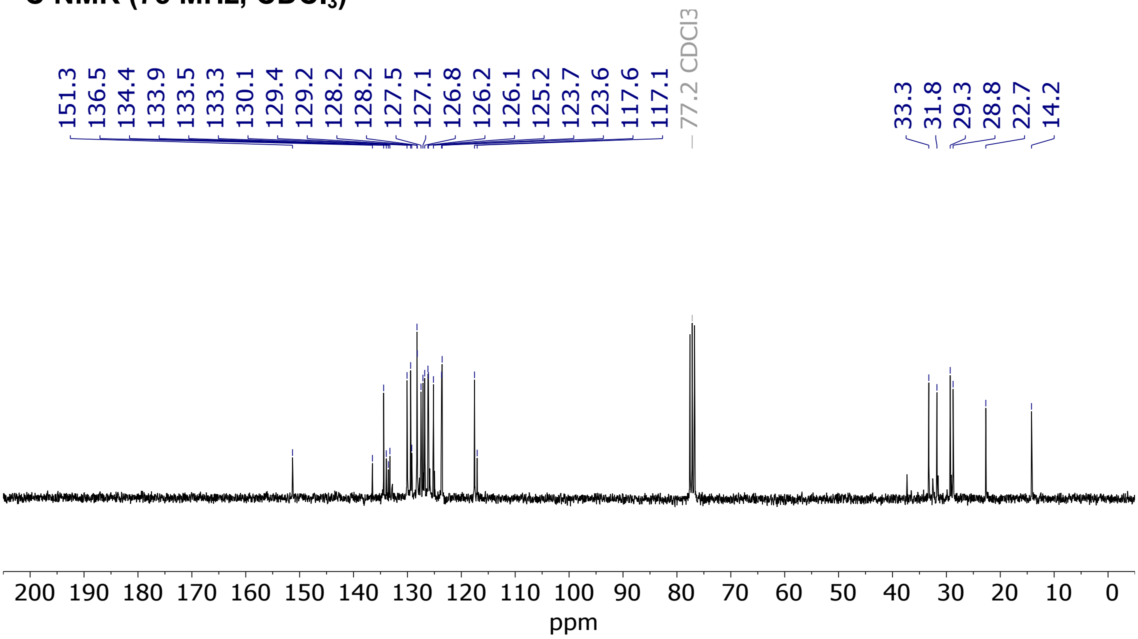

^{1}H NMR (300 MHz, $CDCl_3$)

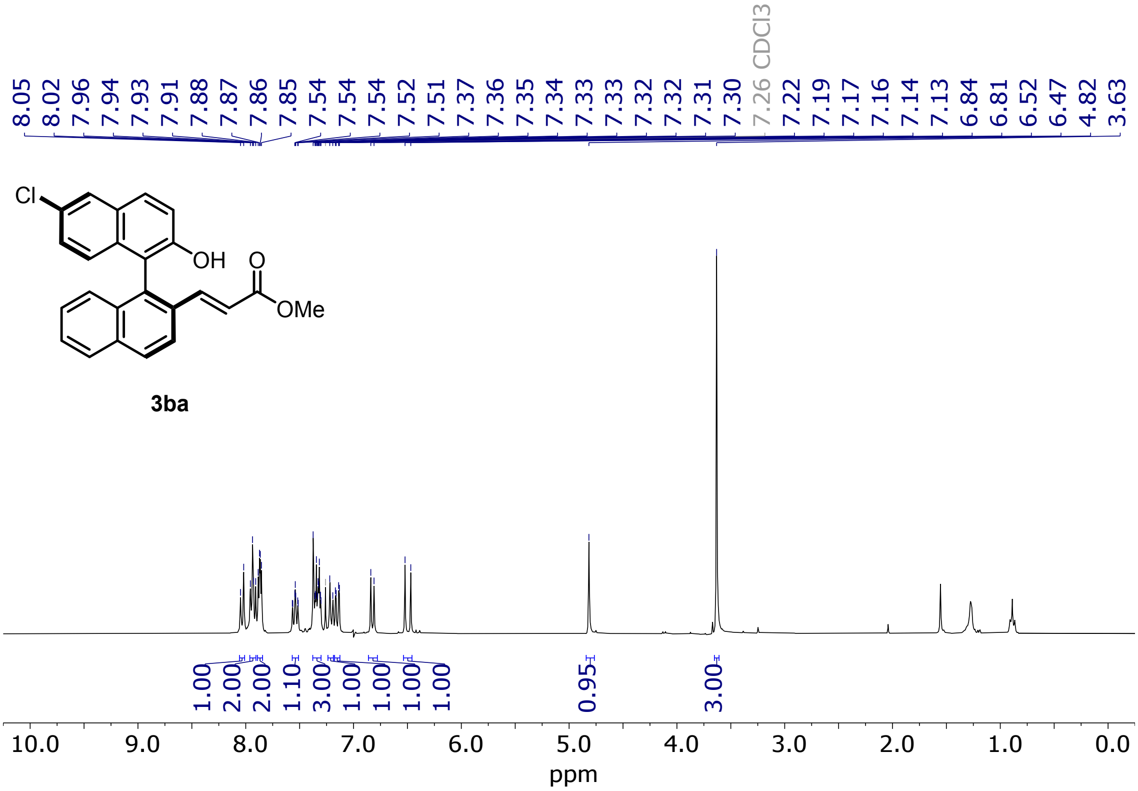

DEPT-135

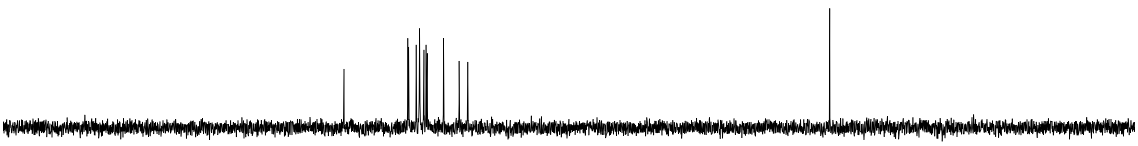

^{13}C NMR (75 MHz, $CDCl_3$)

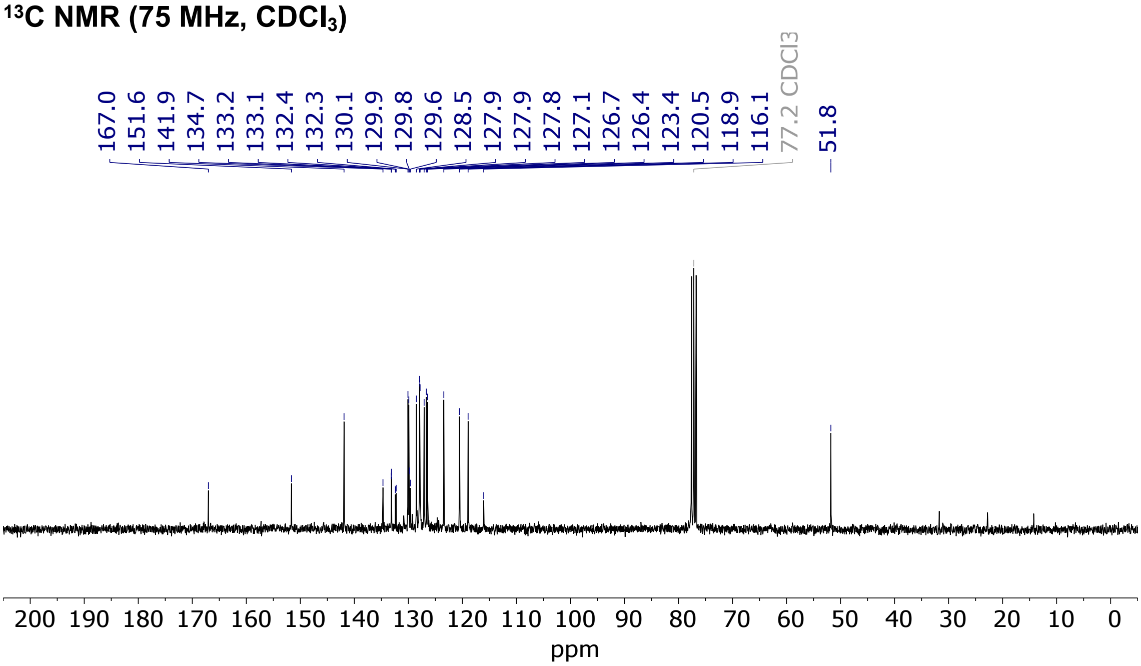

^{1}H NMR (300 MHz, DMSO-d_6)

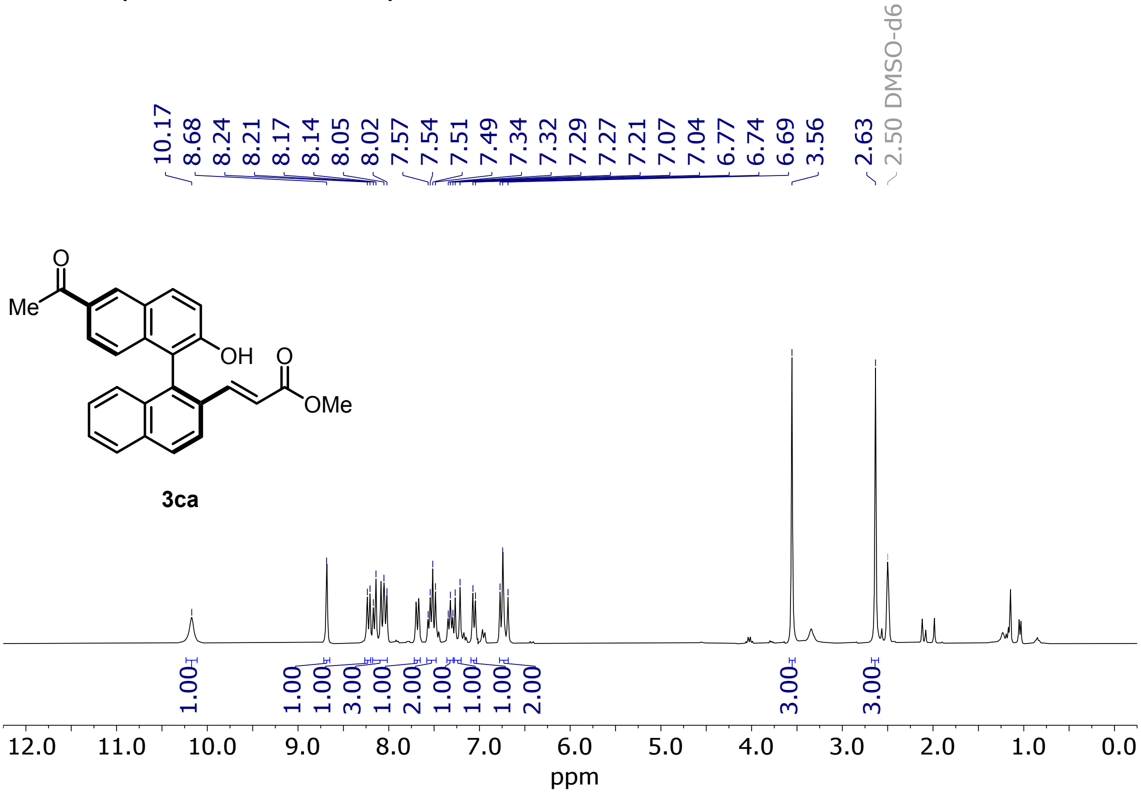

DEPT-135

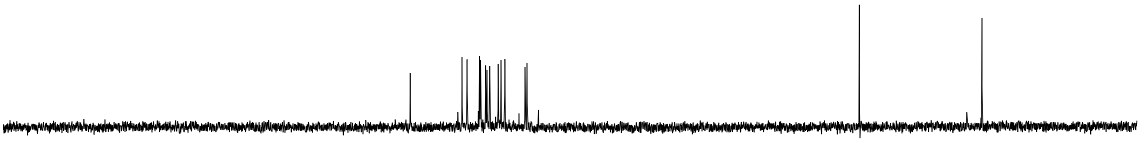

^{13}C NMR (75 MHz, DMSO-d_6)

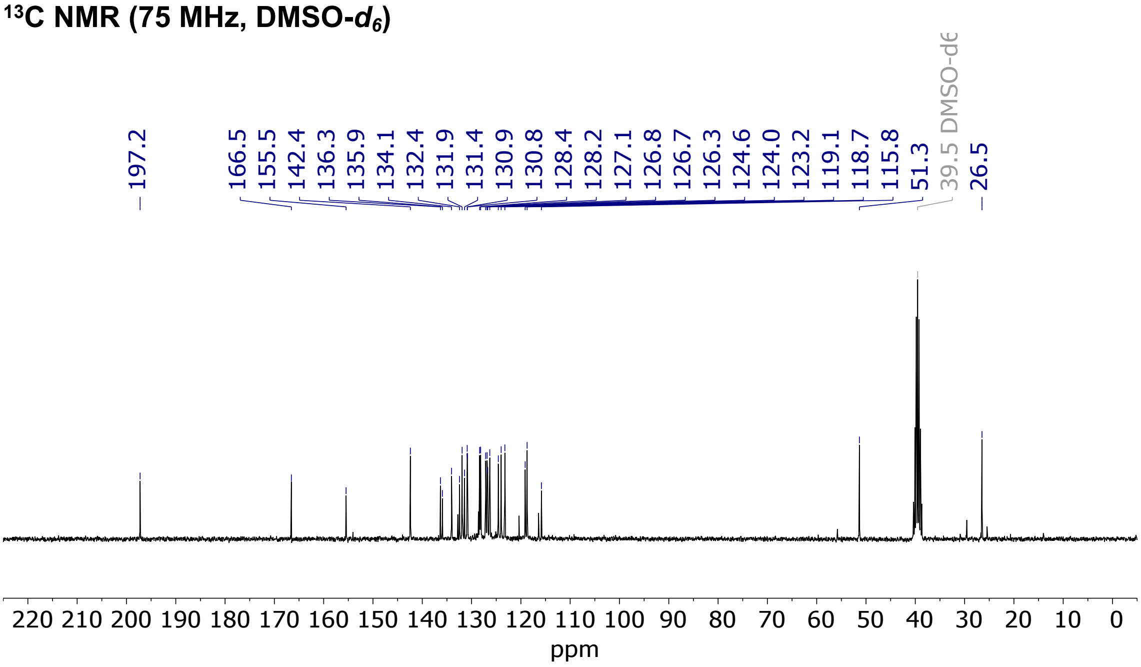

^{1}H NMR (300 MHz, $CDCl_3$)

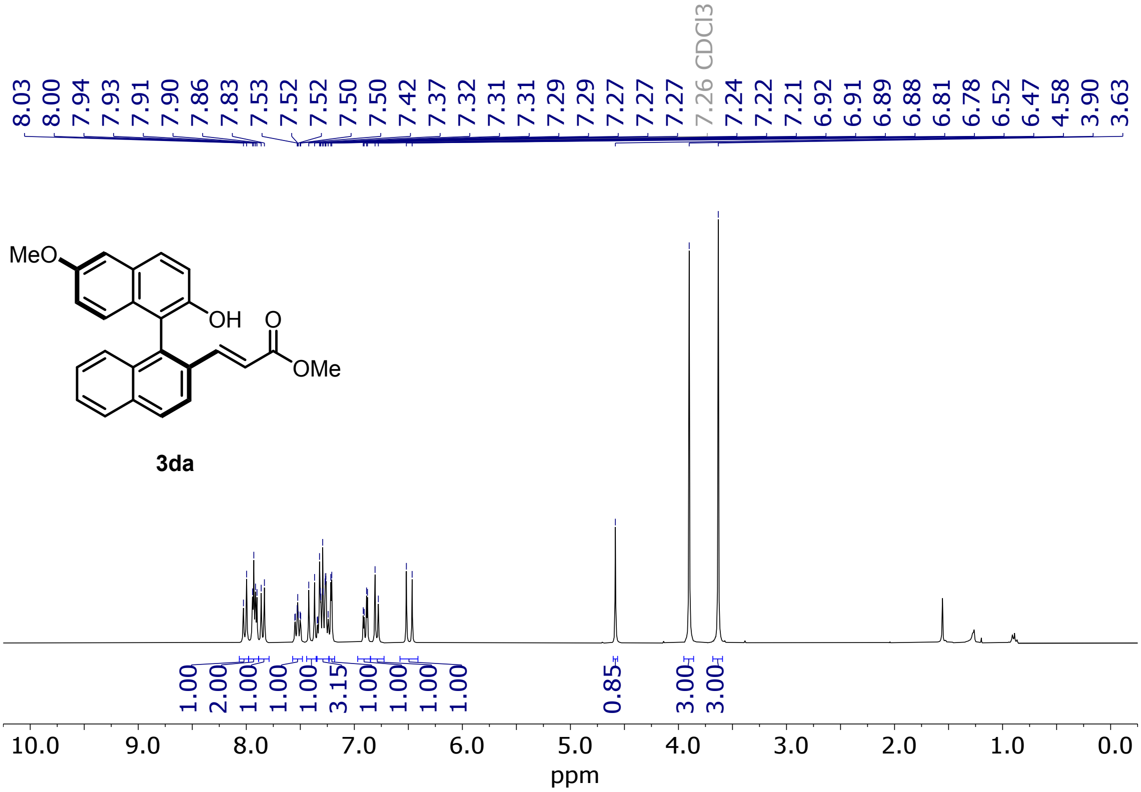

DEPT-135

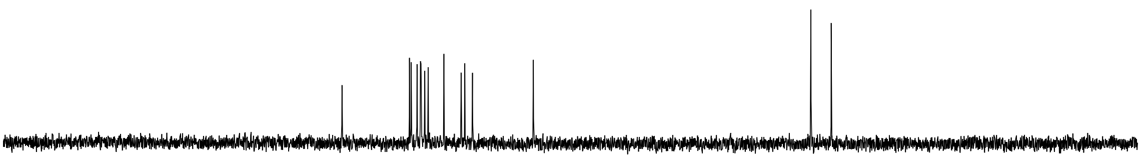

^{13}C NMR (75 MHz, $CDCl_3$)

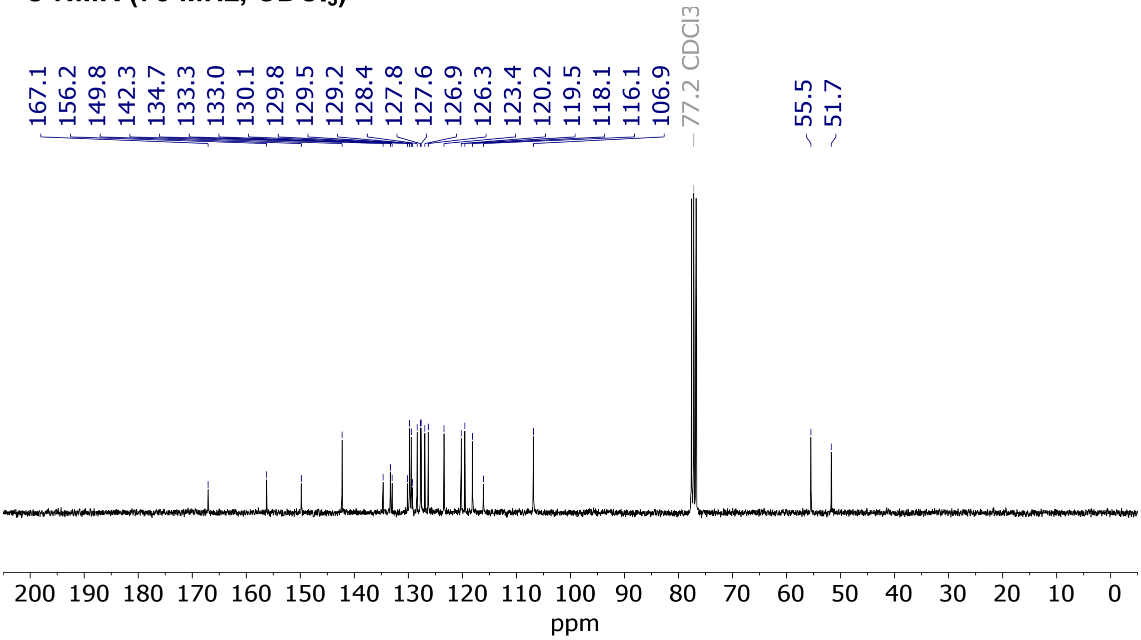

^{1}H NMR (300 MHz, $CDCl_3$)

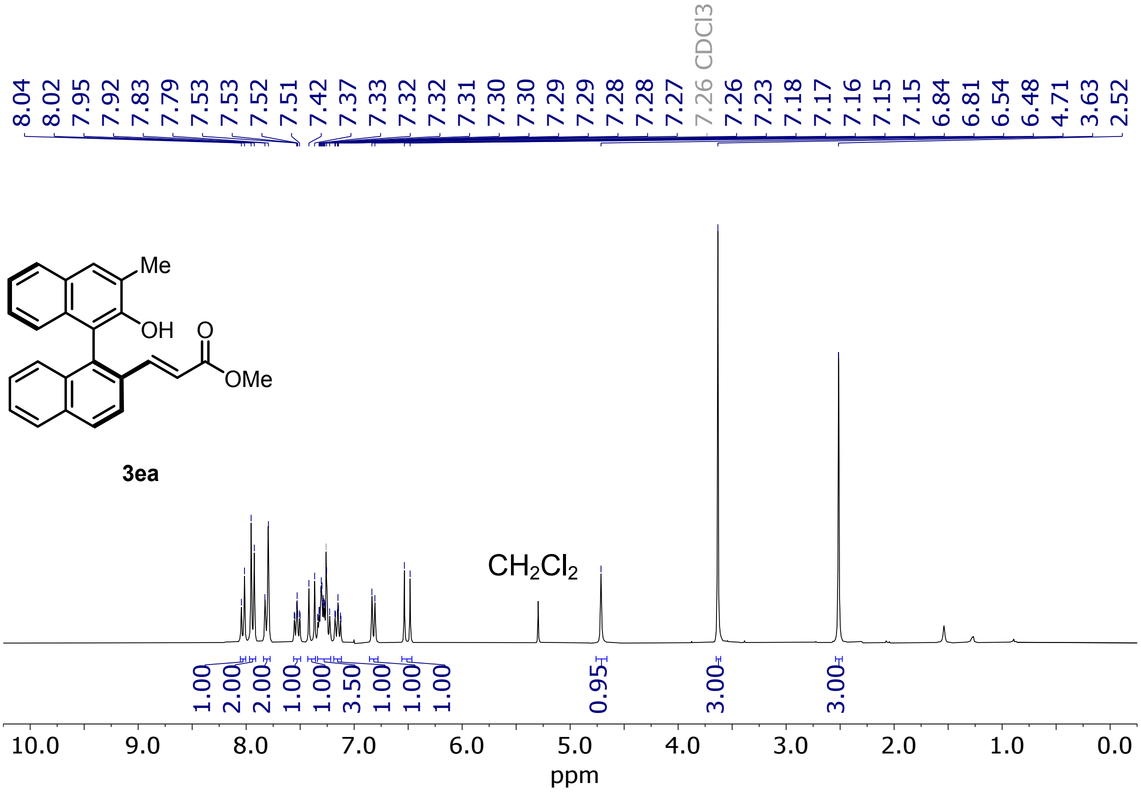

DEPT-135

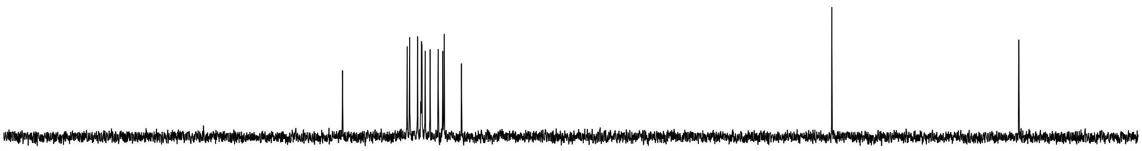

^{13}C NMR (75 MHz, $CDCl_3$)

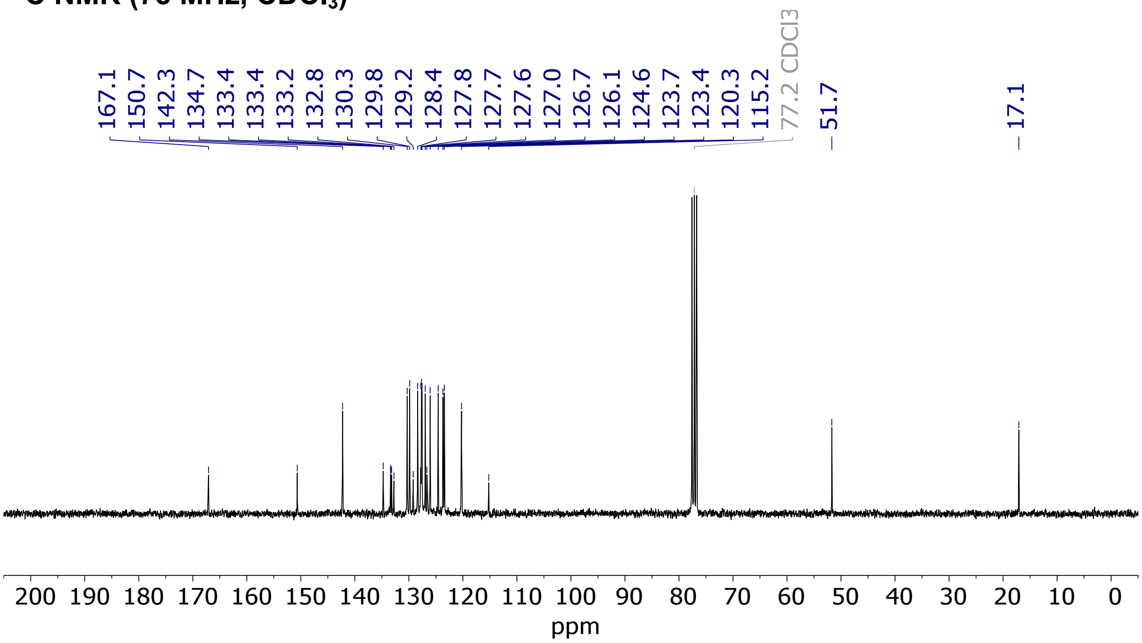

¹H NMR (300 MHz, $CDCl_3$)

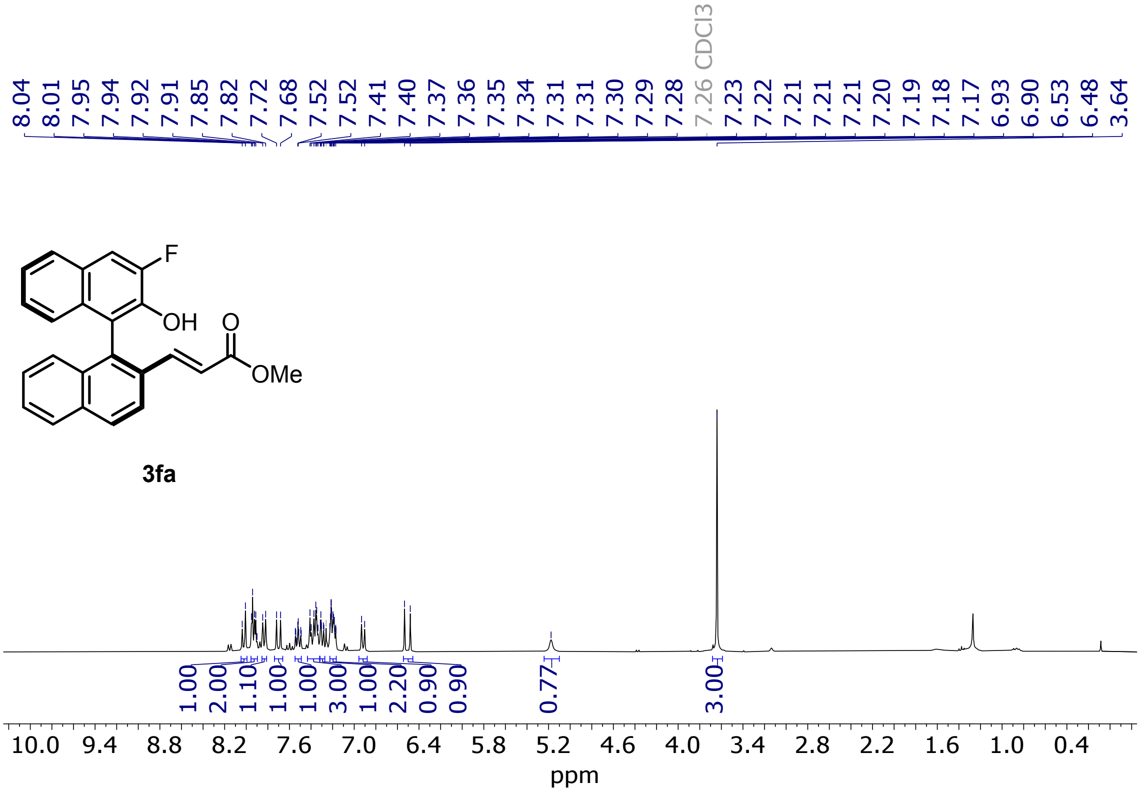

3fa

DEPT-135

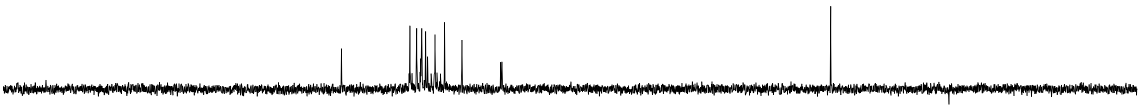

¹³C NMR (75 MHz, $CDCl_3$)

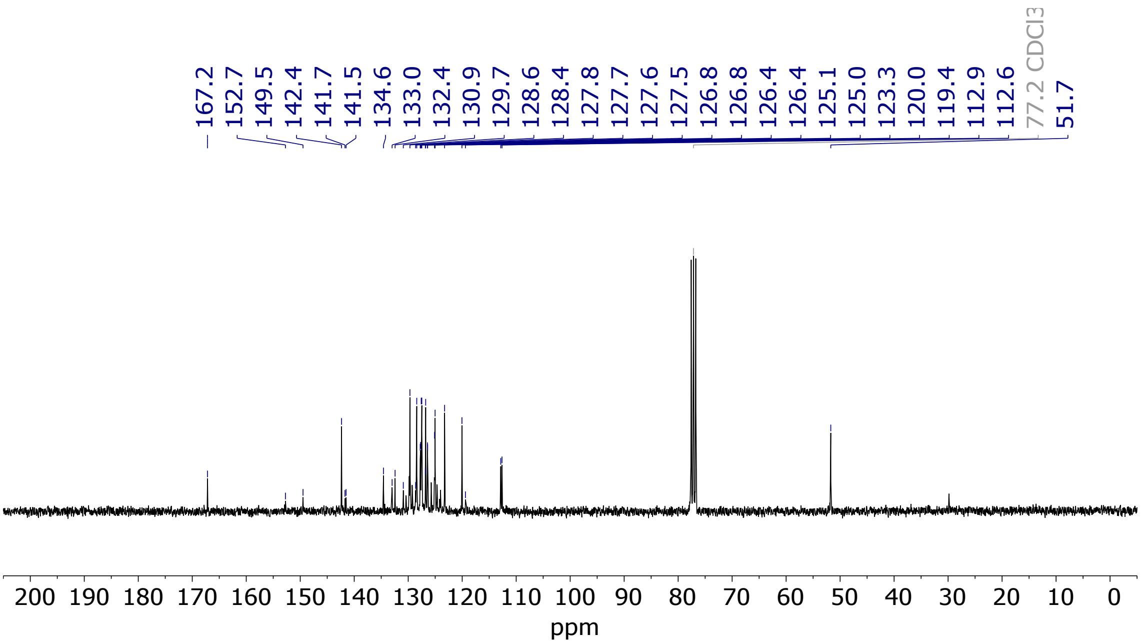

^{1}H NMR (300 MHz, $CDCl_3$)

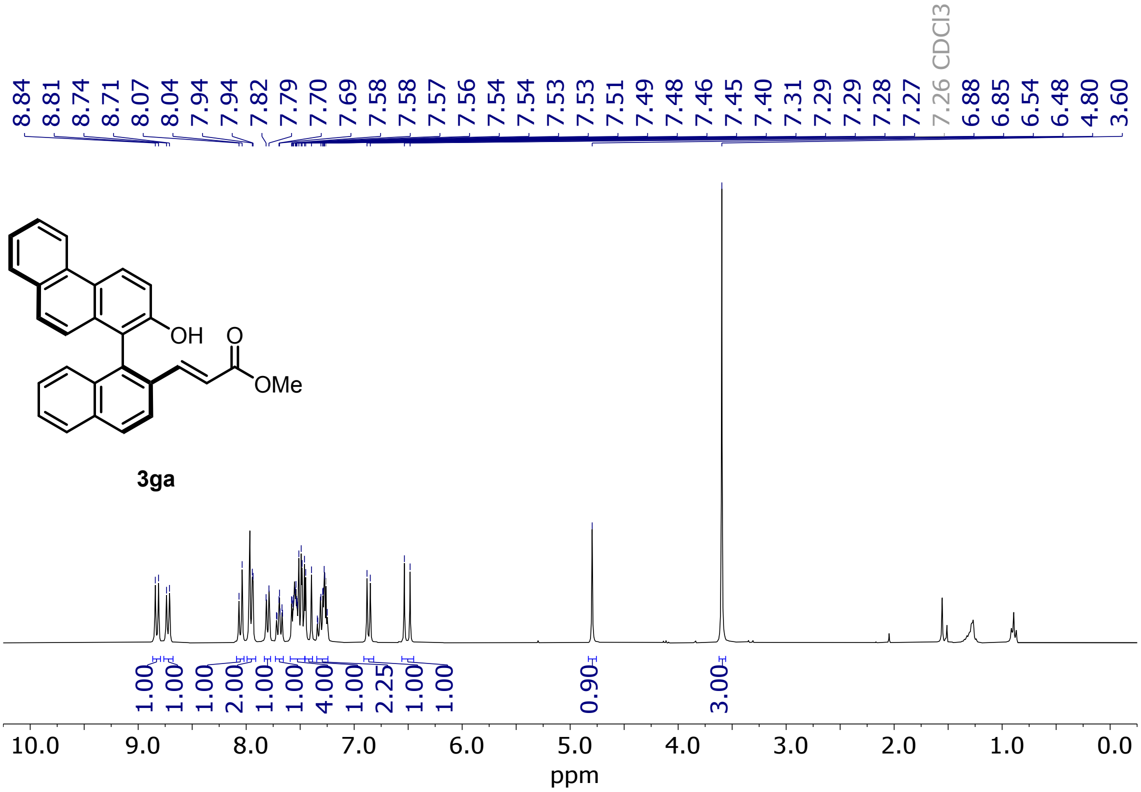

DEPT-135

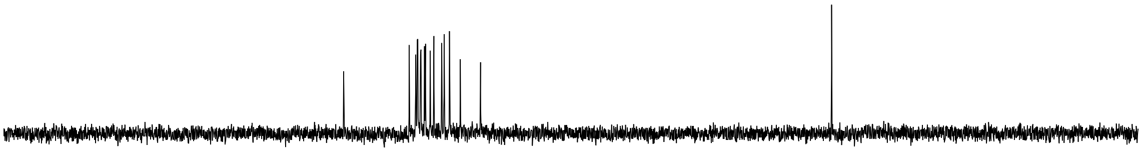

^{13}C NMR (75 MHz, $CDCl_3$)

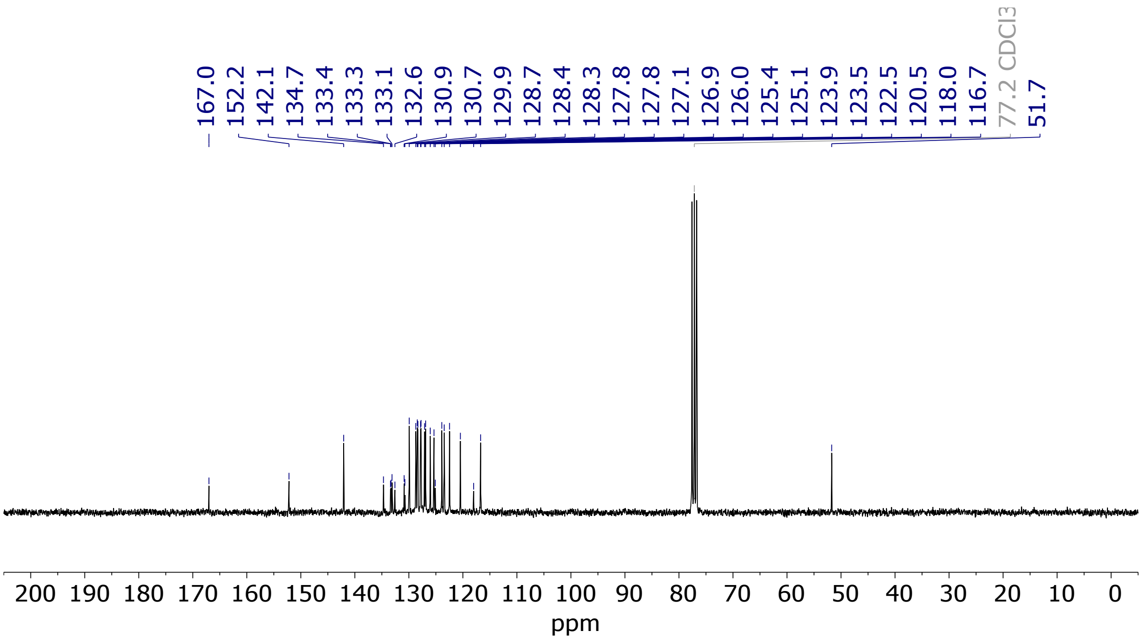

^{1}H NMR (500 MHz, $CDCl_3$)

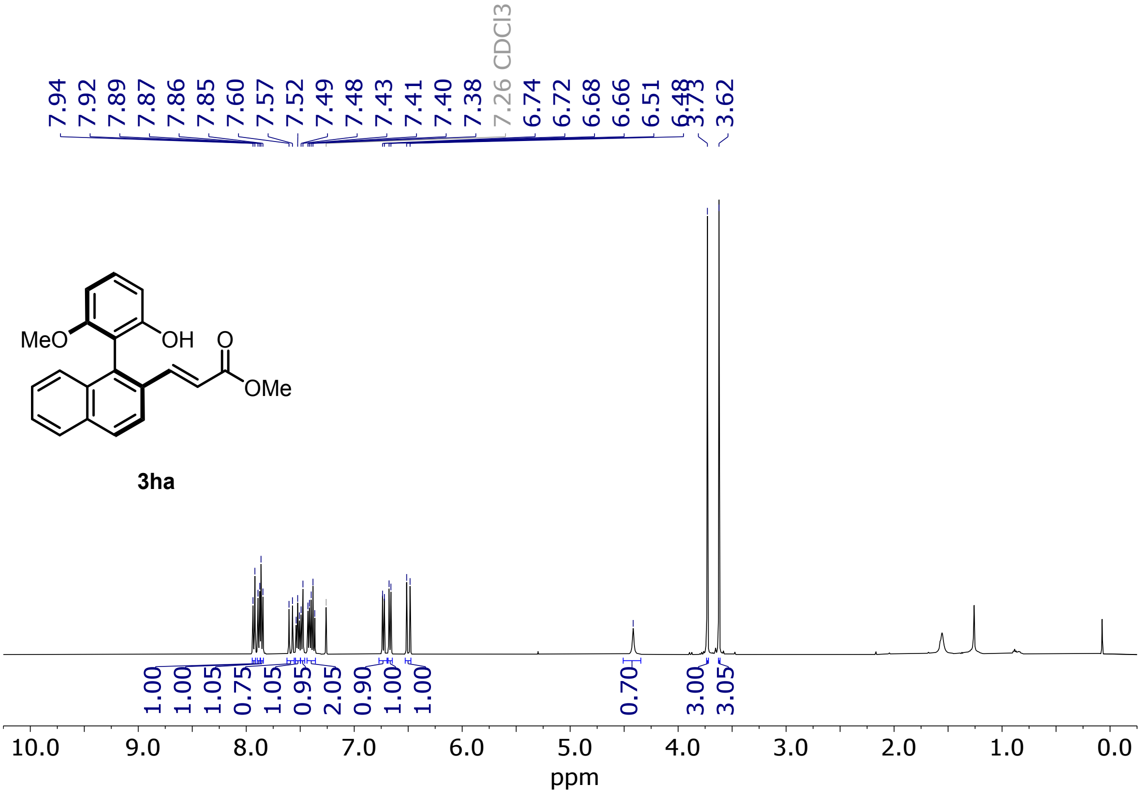

DEPT-135

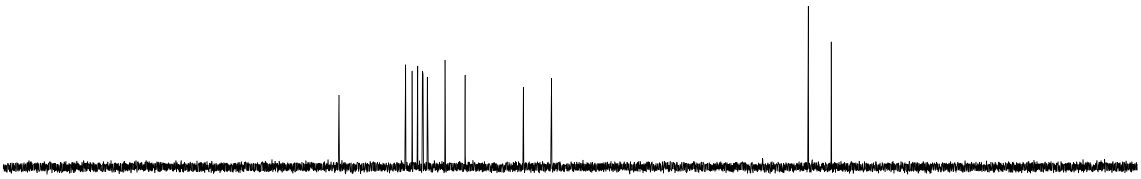

^{13}C NMR (126 MHz, $CDCl_3$)

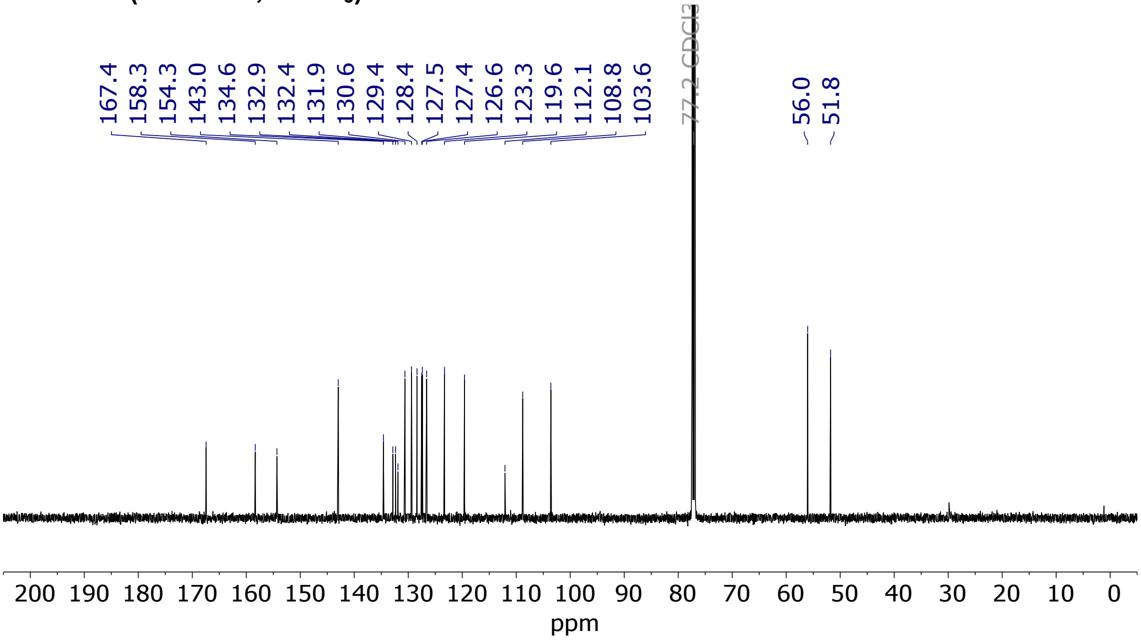

^{1}H NMR (300 MHz, $CDCl_3$)

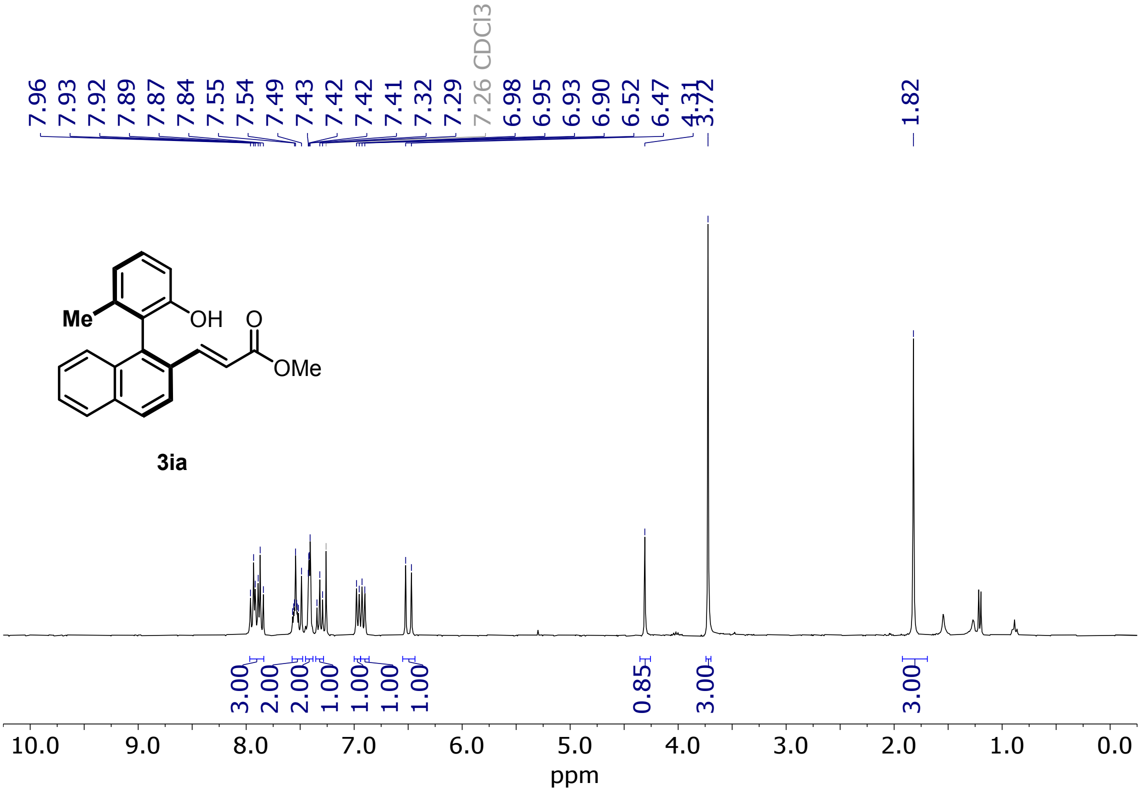

DEPT-135

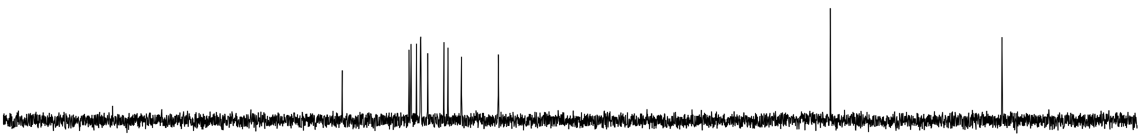

^{13}C NMR (75 MHz, $CDCl_3$)

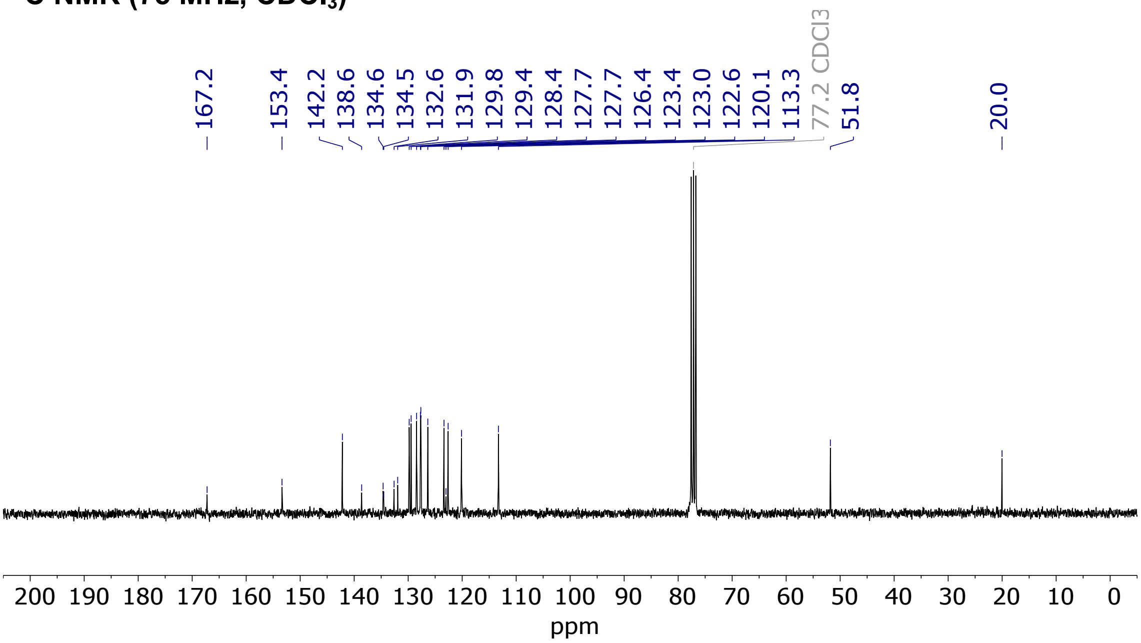

^{1}H NMR (500 MHz, $CDCl_3$)

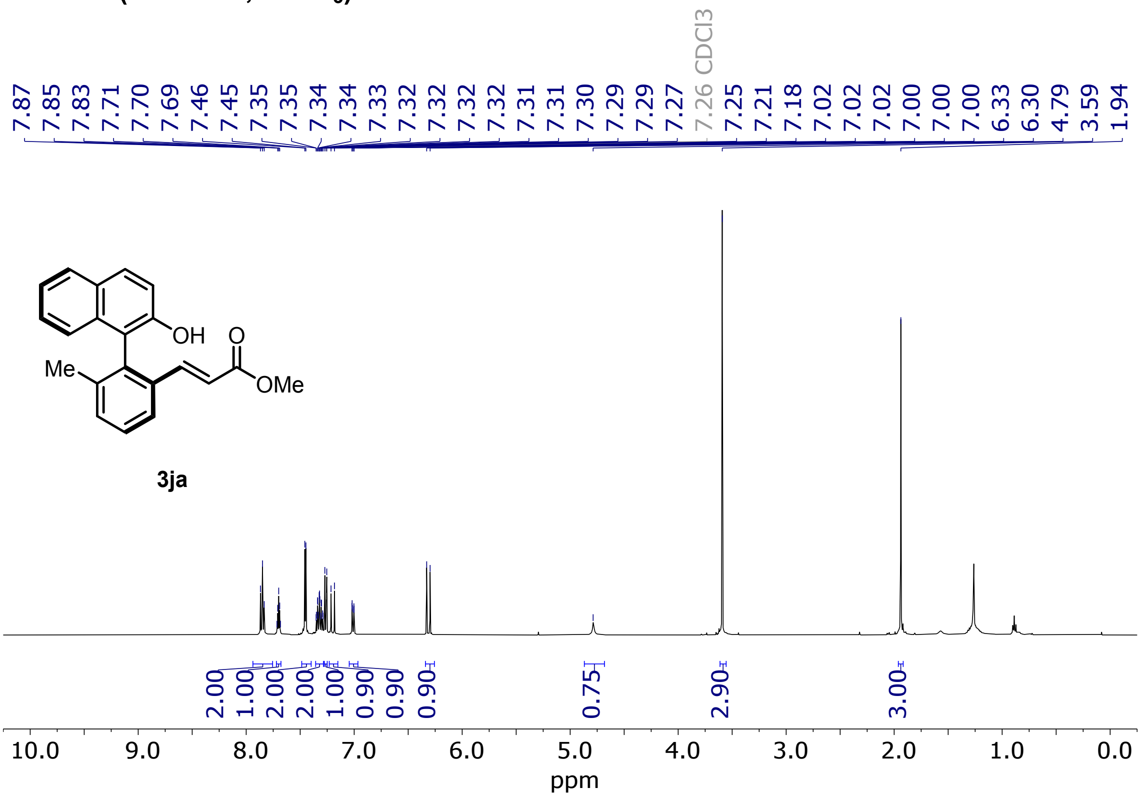

DEPT-135

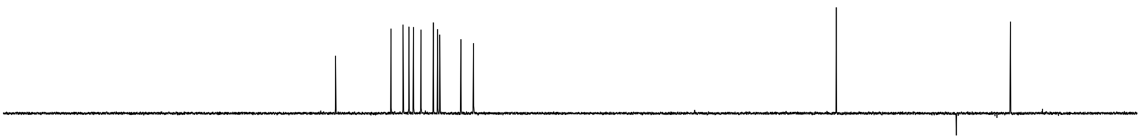

^{13}C NMR (126 MHz, $CDCl_3$)

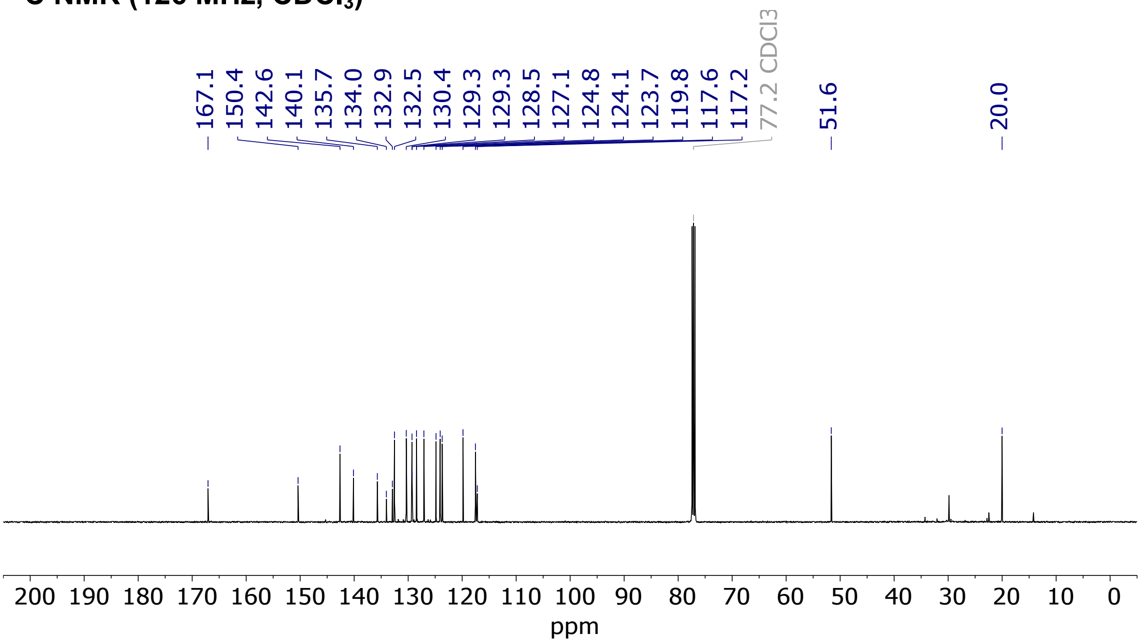

^{1}H NMR (300 MHz, $CDCl_3$)

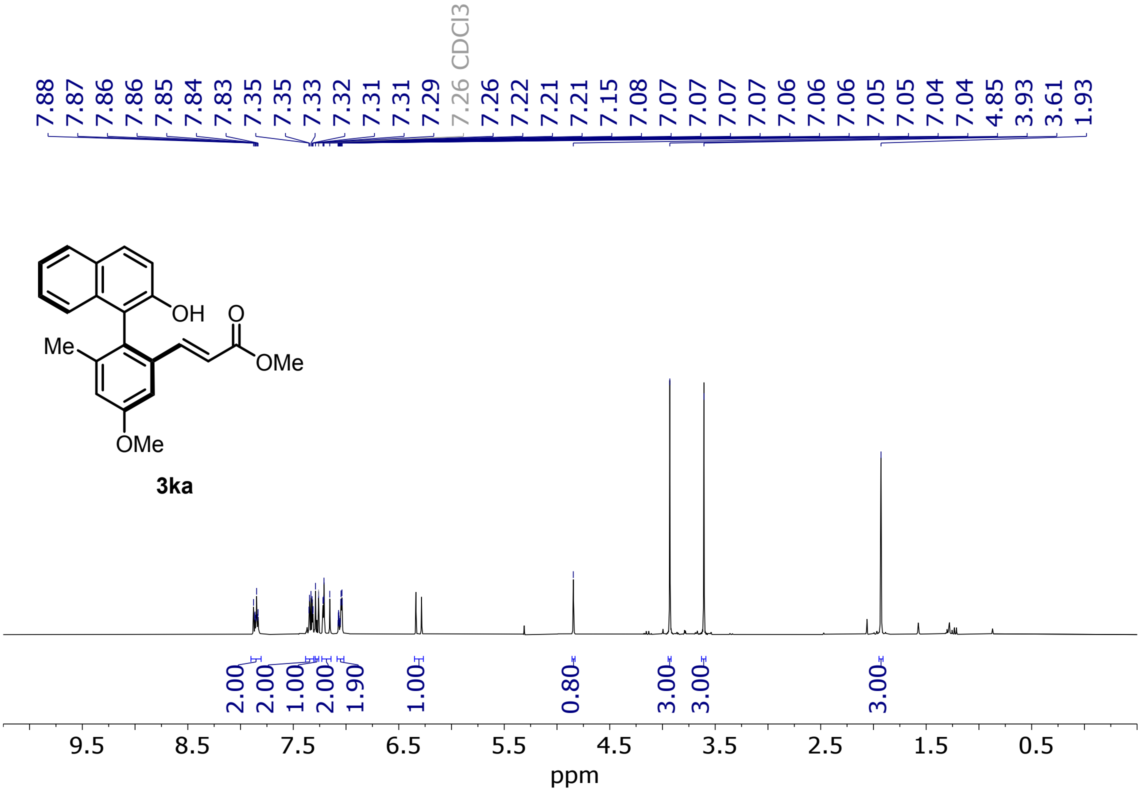

3ka

DEPT-135

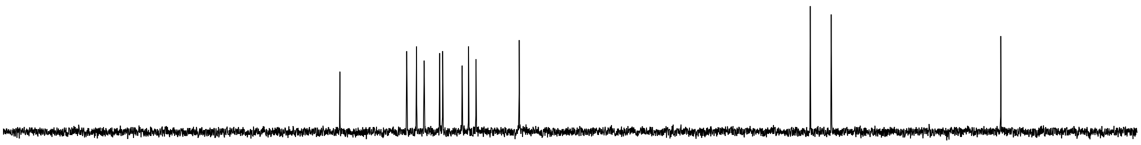

^{13}C NMR (75 MHz, $CDCl_3$)

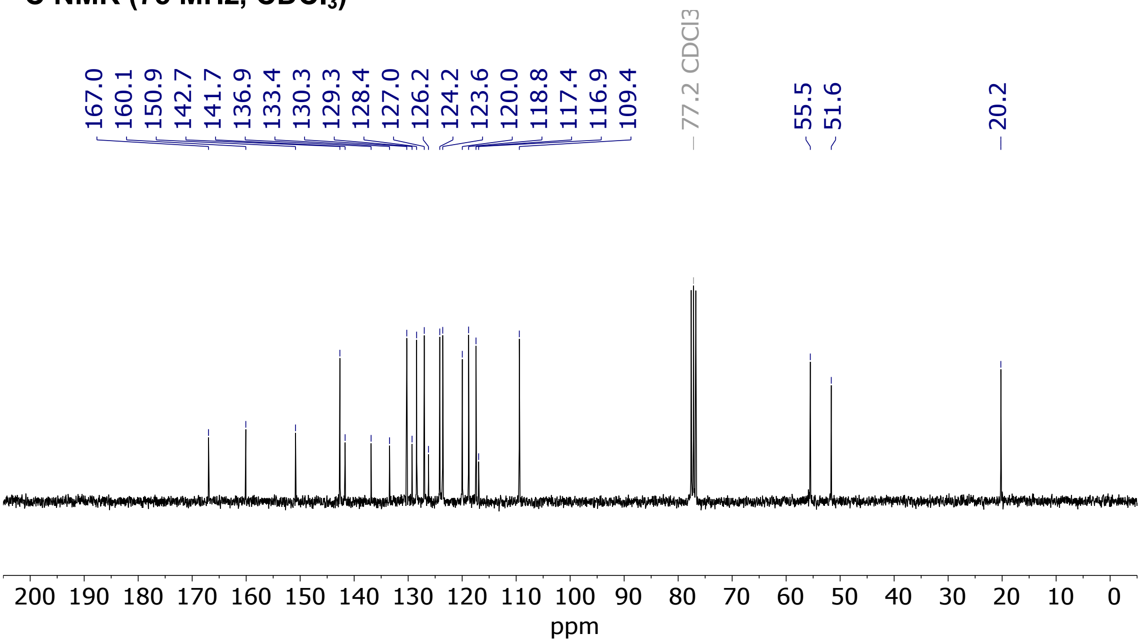

^{1}H NMR (300 MHz, $CDCl_3$)

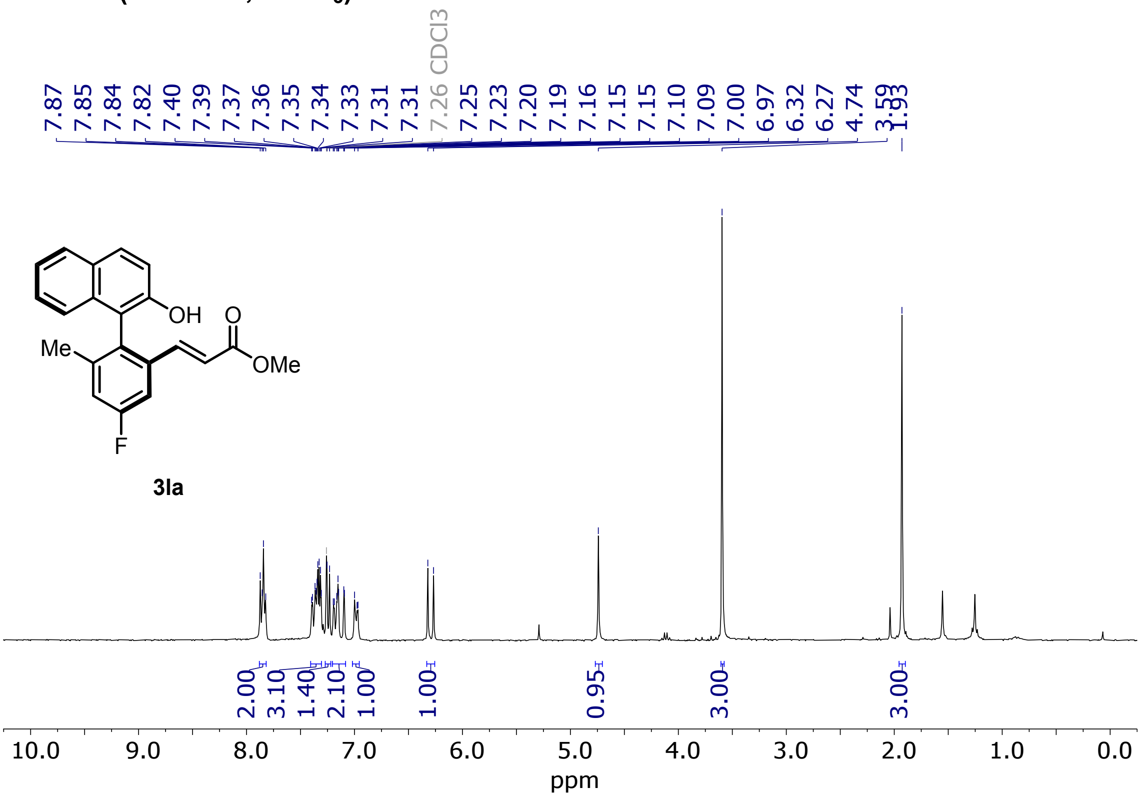

DEPT-135

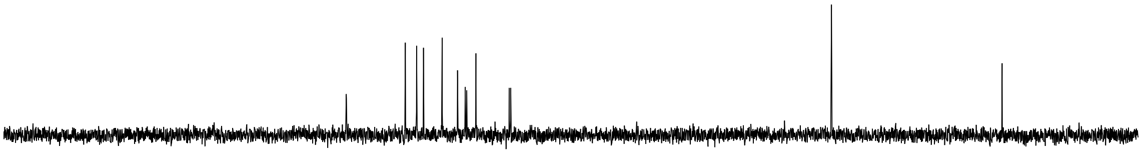

^{13}C NMR (75 MHz, $CDCl_3$)

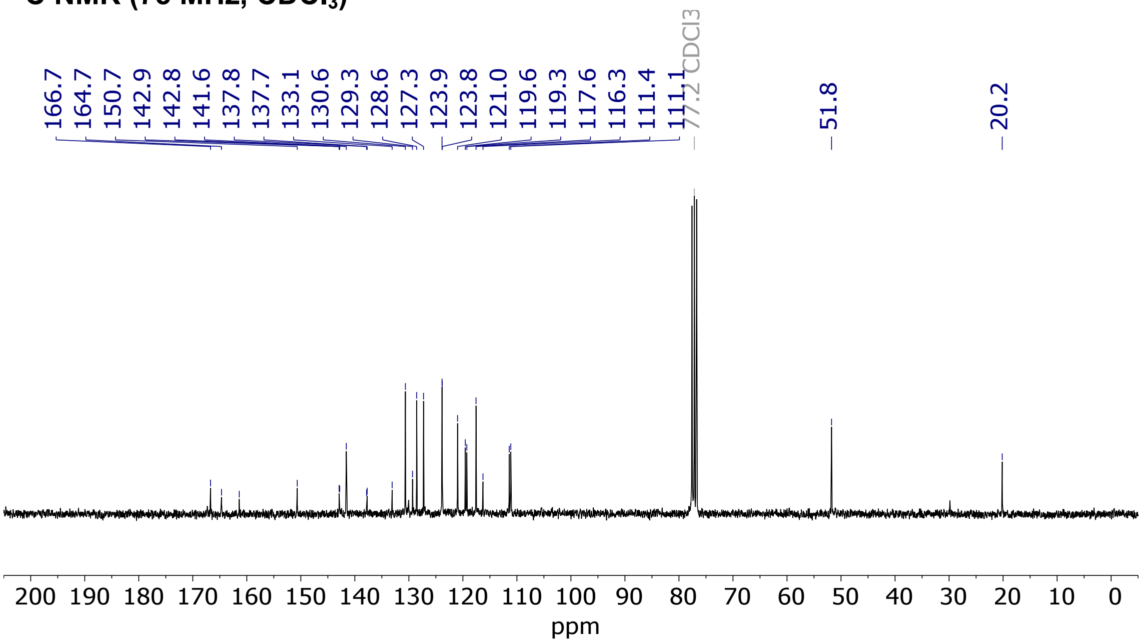

^{1}H NMR (500 MHz, $CDCl_3$)

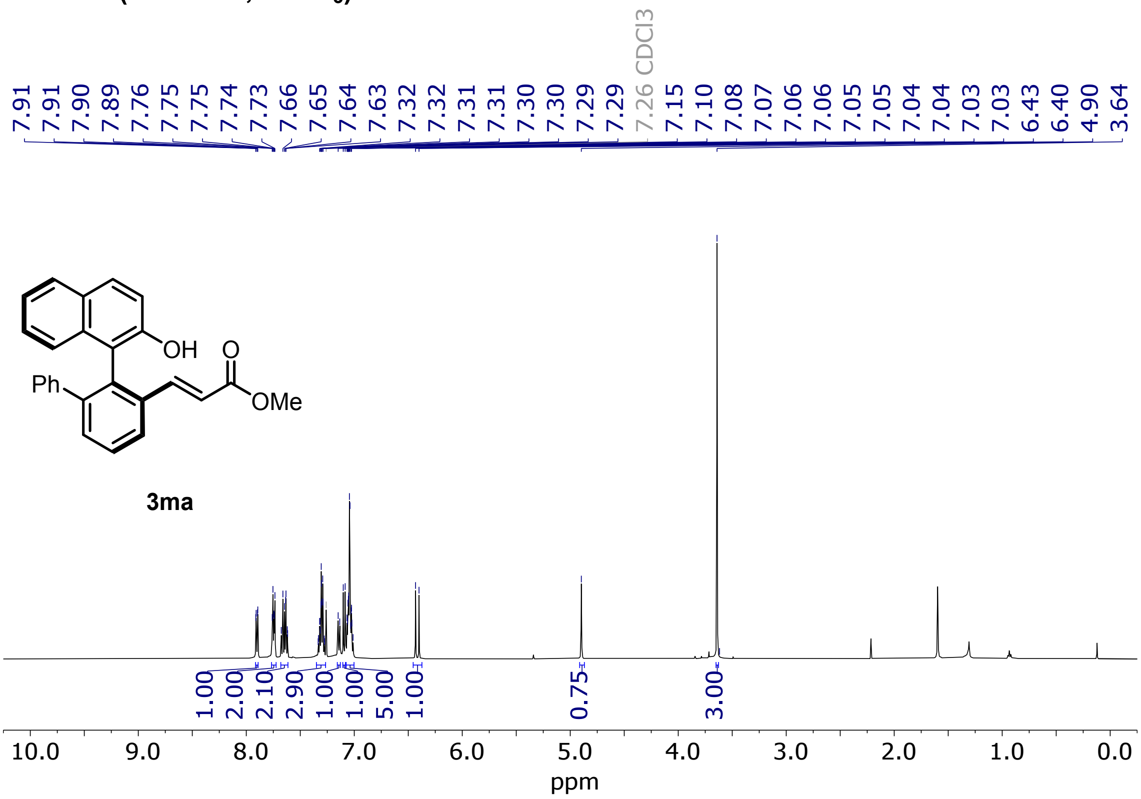

DEPT-135

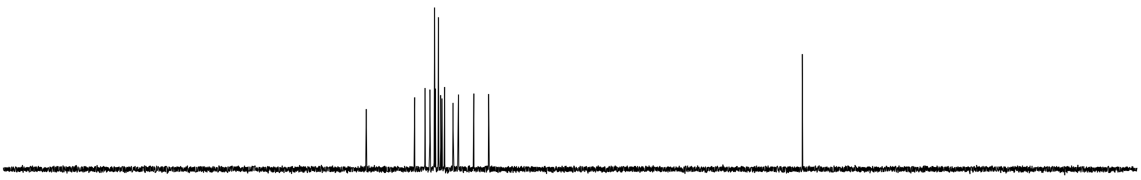

^{13}C NMR (126 MHz, $CDCl_3$)

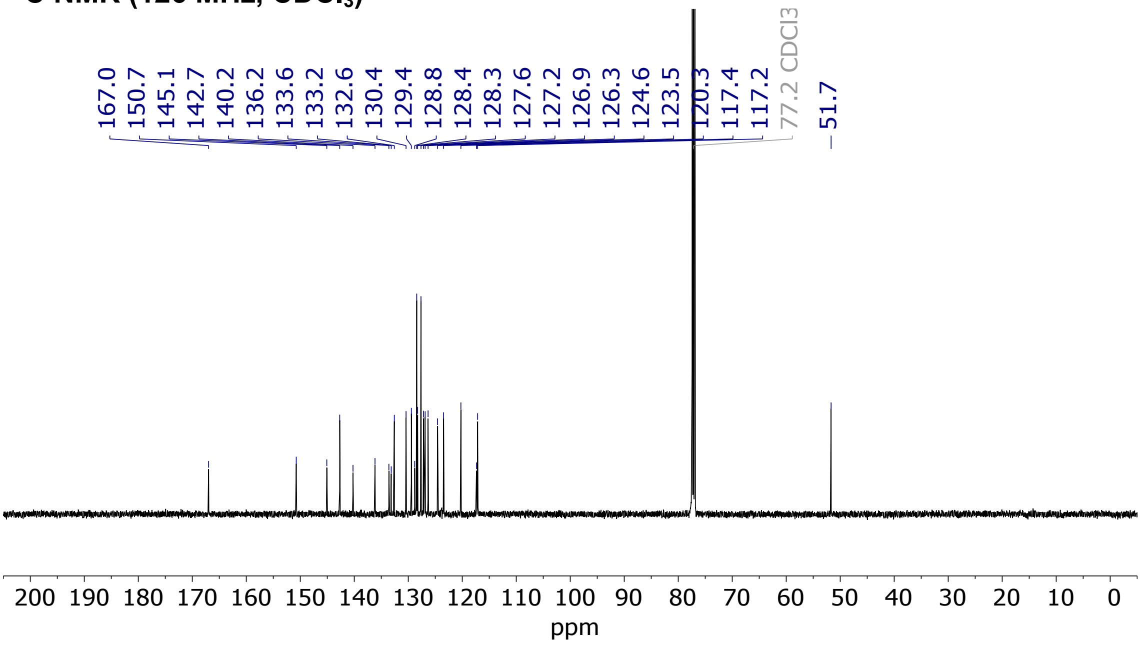

^{1}H NMR (500 MHz, $CDCl_3$)

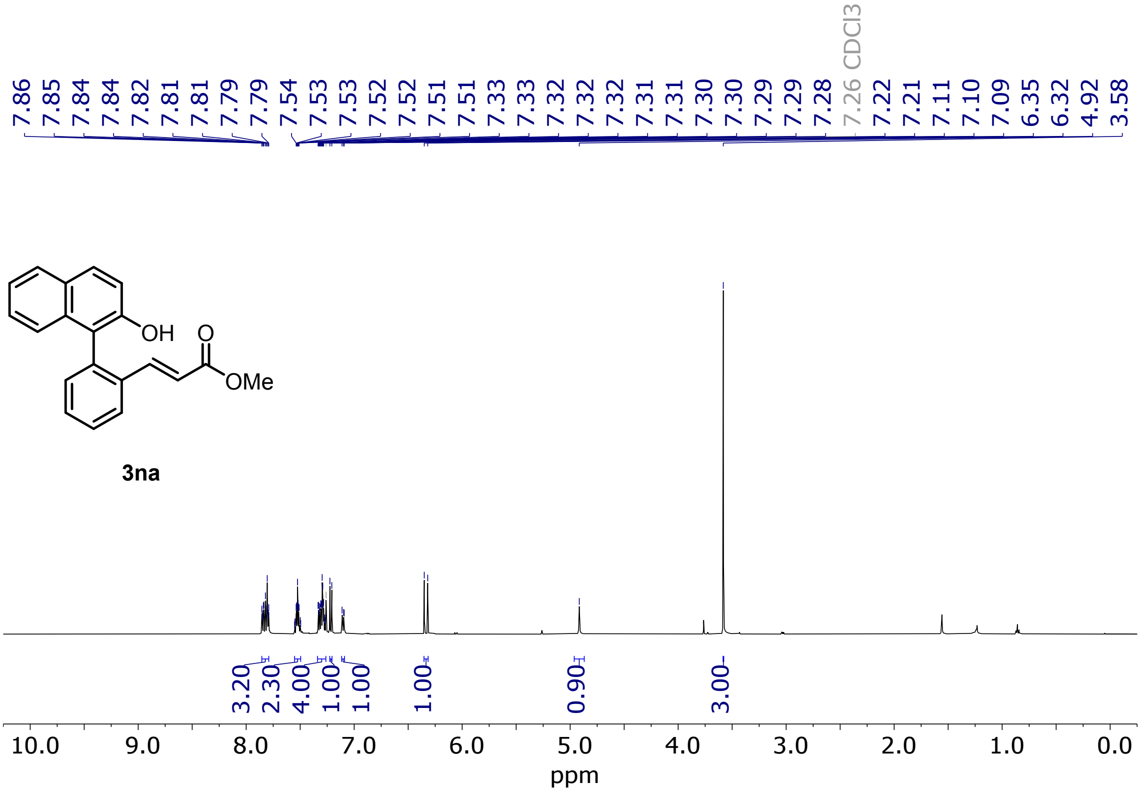

3na

DEPT-135

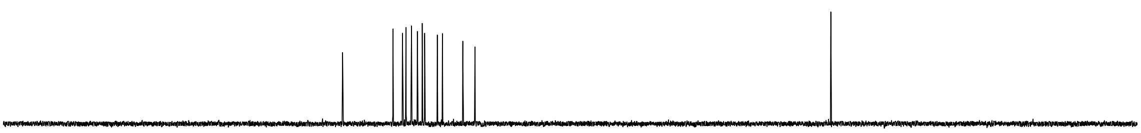

^{13}C NMR (126 MHz, $CDCl_3$)

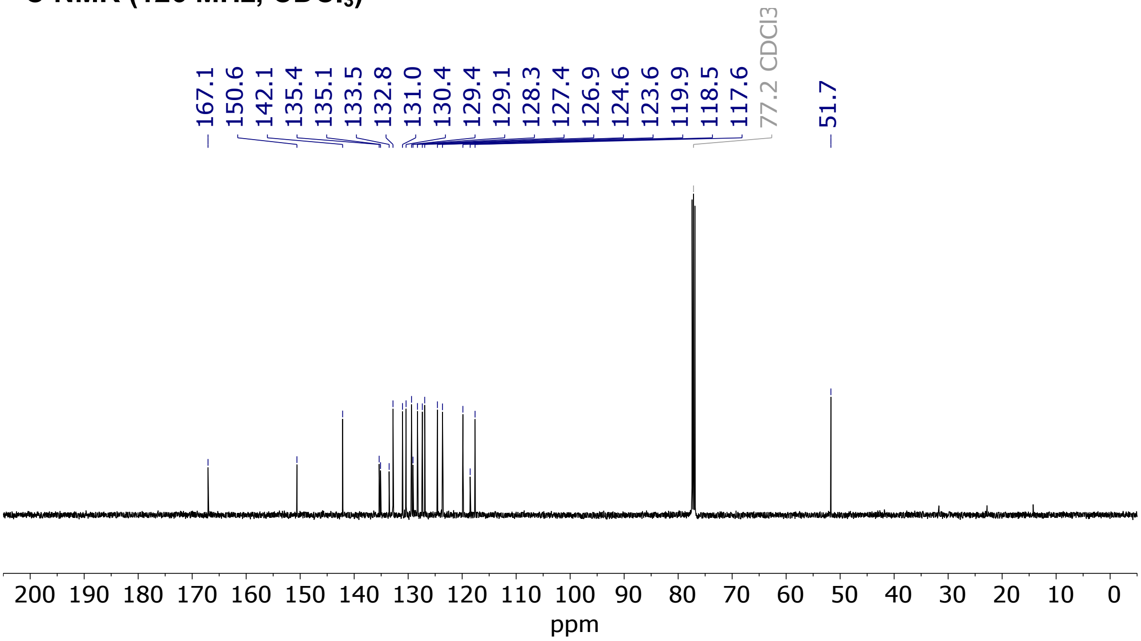

^1H NMR (500 MHz, $CDCl_3$)

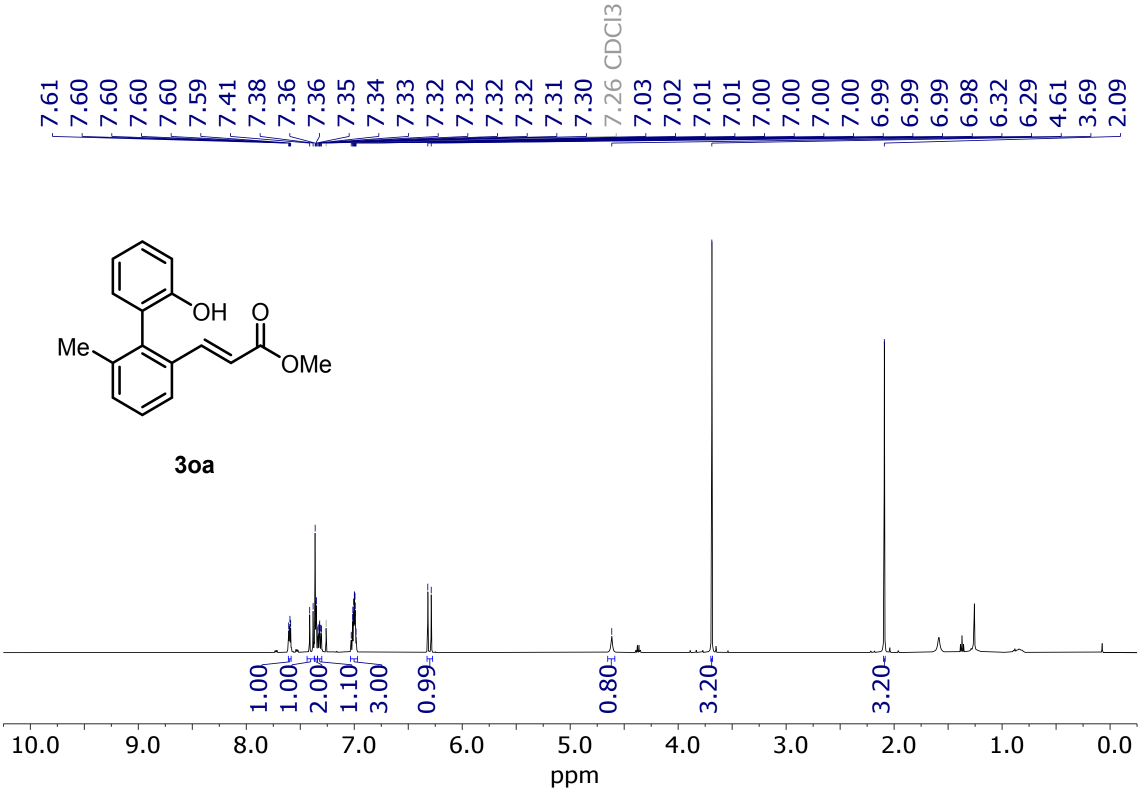

DEPT-135

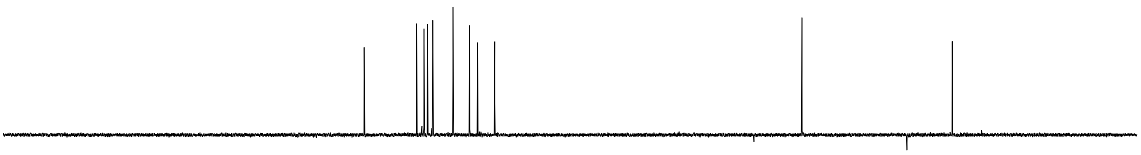

^{13}C NMR (126 MHz, $CDCl_3$)

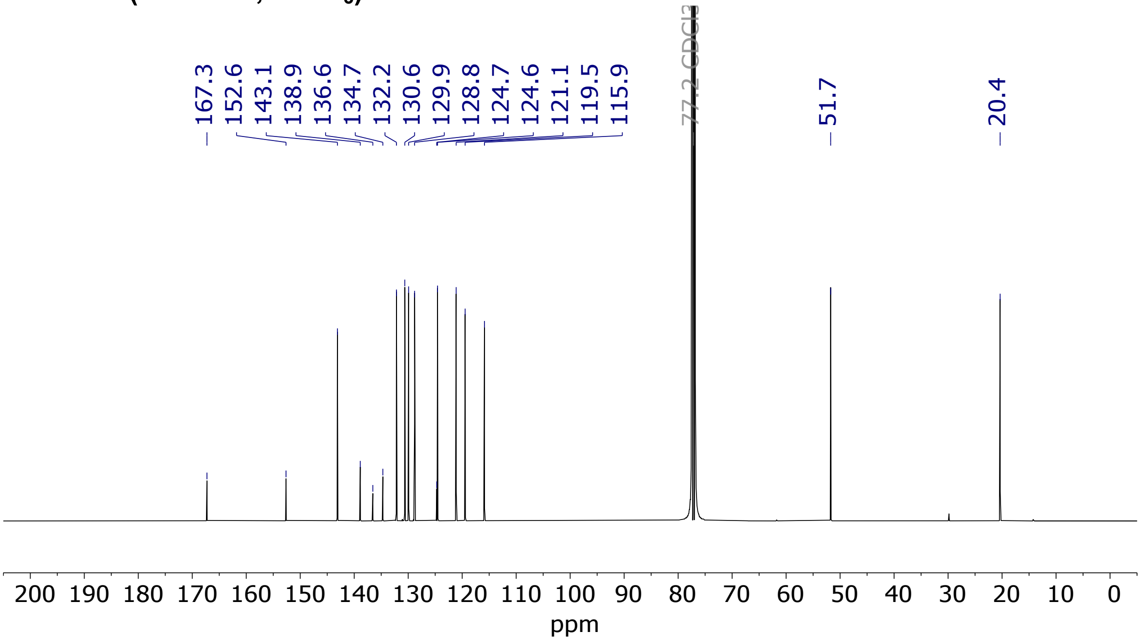

^{1}H NMR (500 MHz, $CDCl_3$)

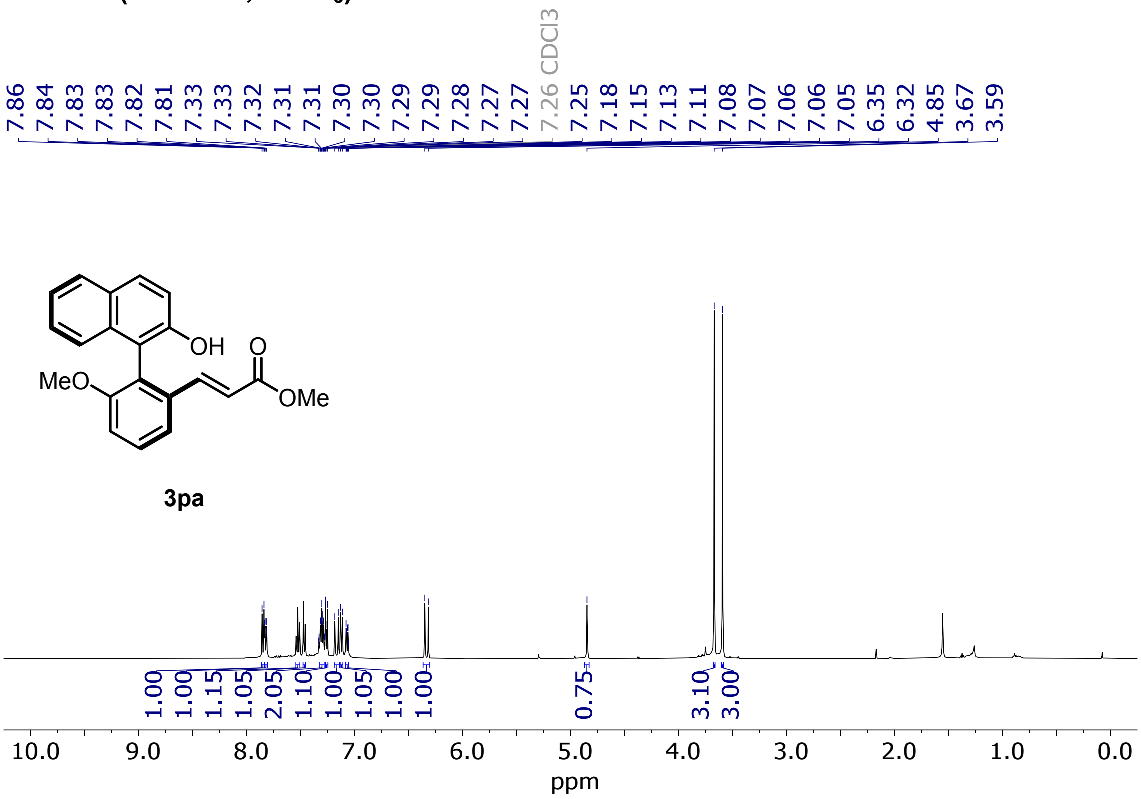

DEPT-135

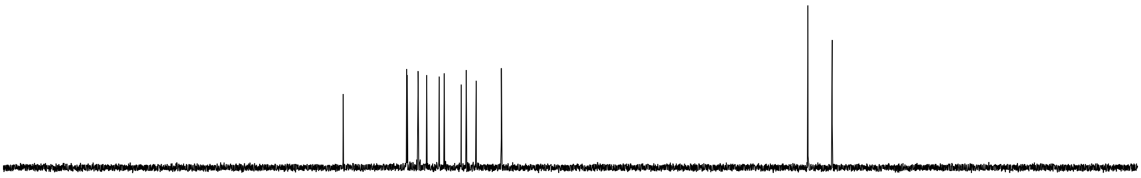

^{13}C NMR (126 MHz, $CDCl_3$)

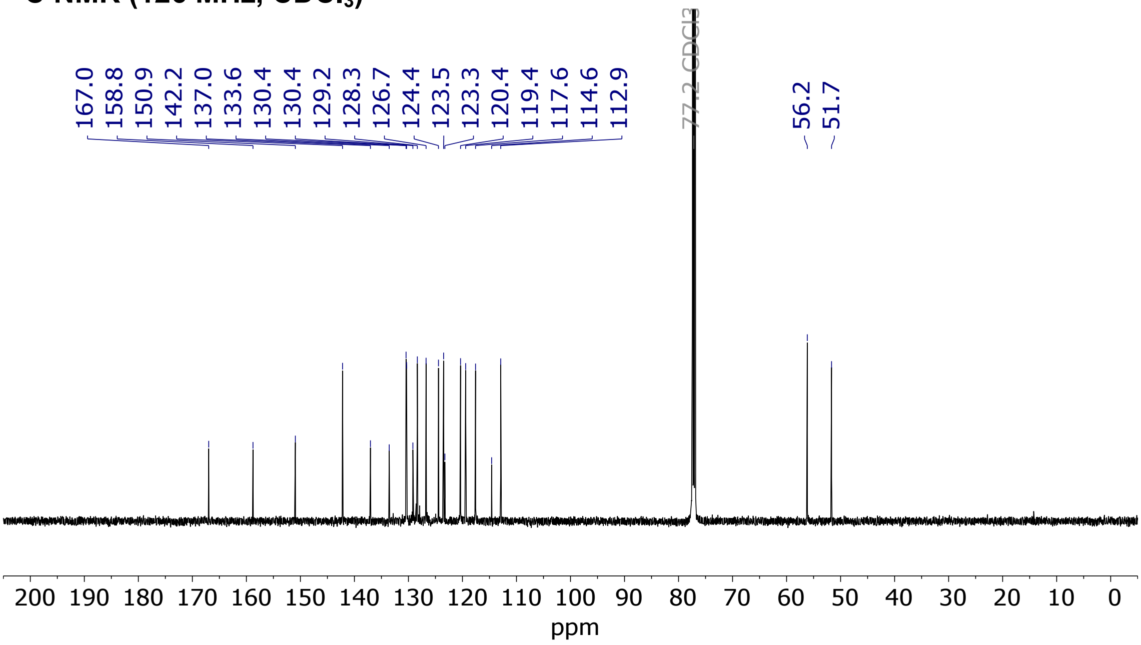

^{1}H NMR (300 MHz, CDCl$_3$)

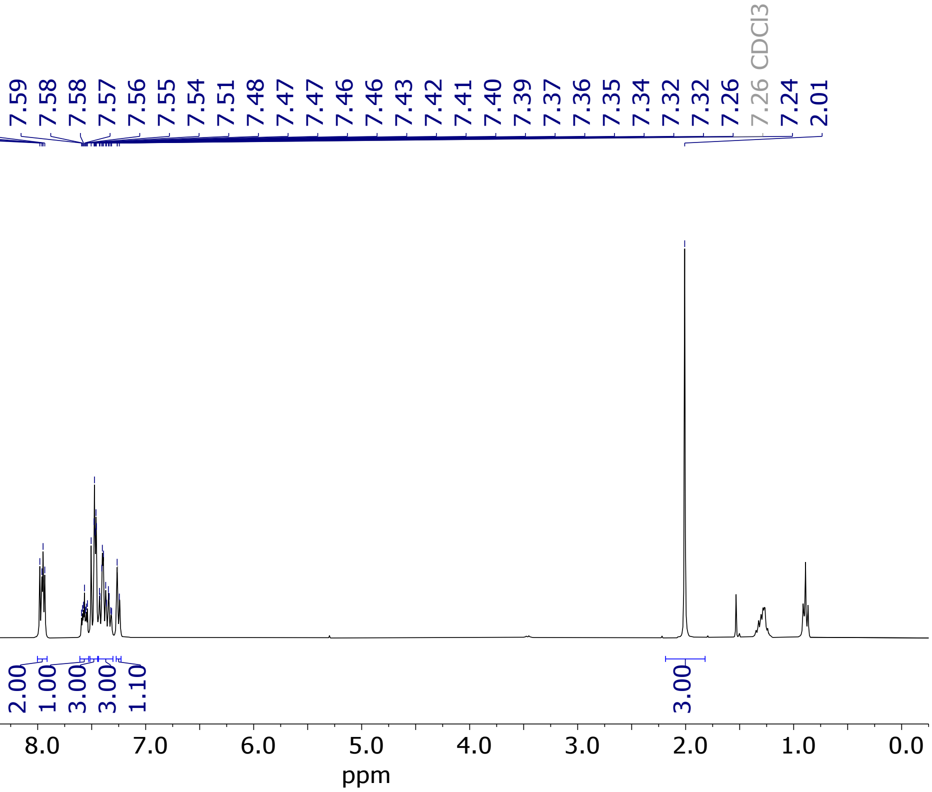

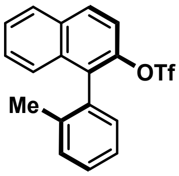

4

DEPT-135

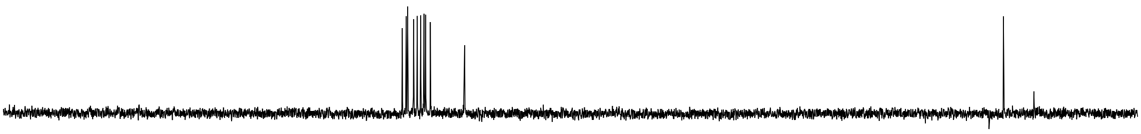

^{13}C NMR (75 MHz, CDCl$_3$)

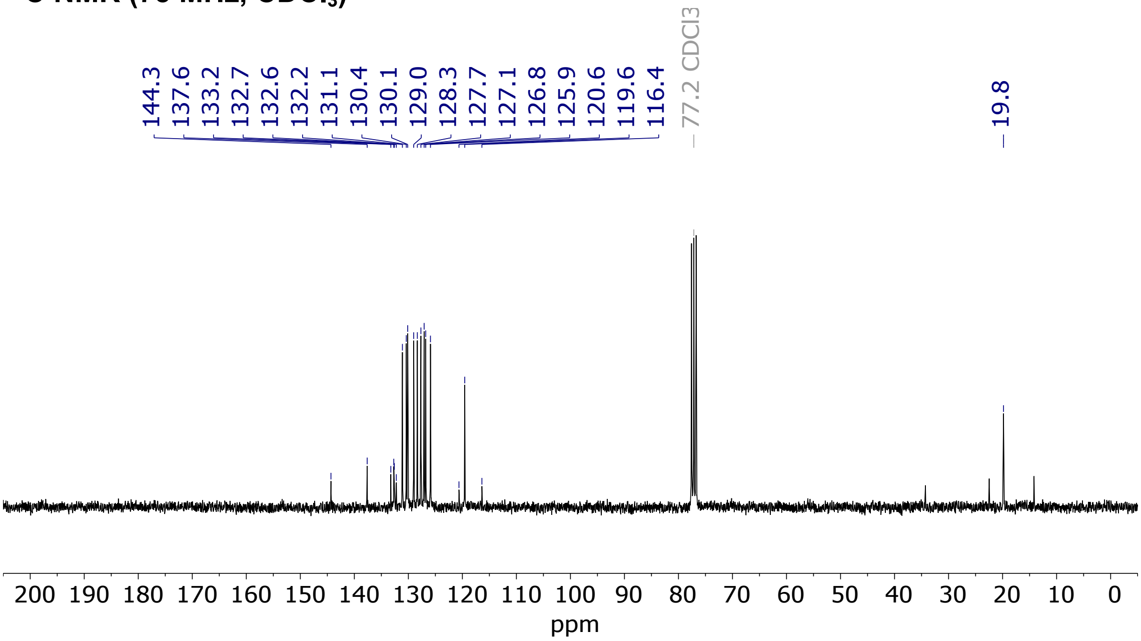

^{1}H NMR (500 MHz, $CDCl_3$)

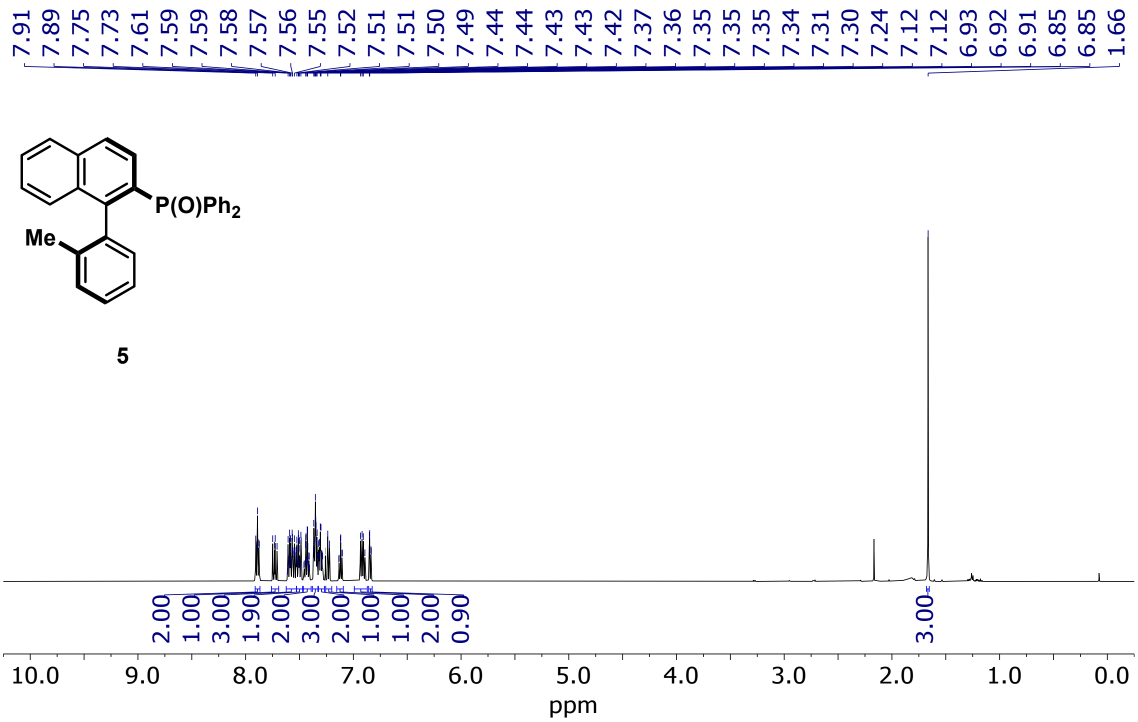

5

DEPT-135

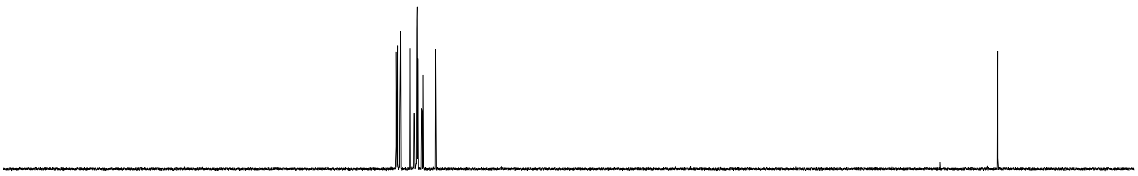

^{13}C NMR (126 MHz, $CDCl_3$)

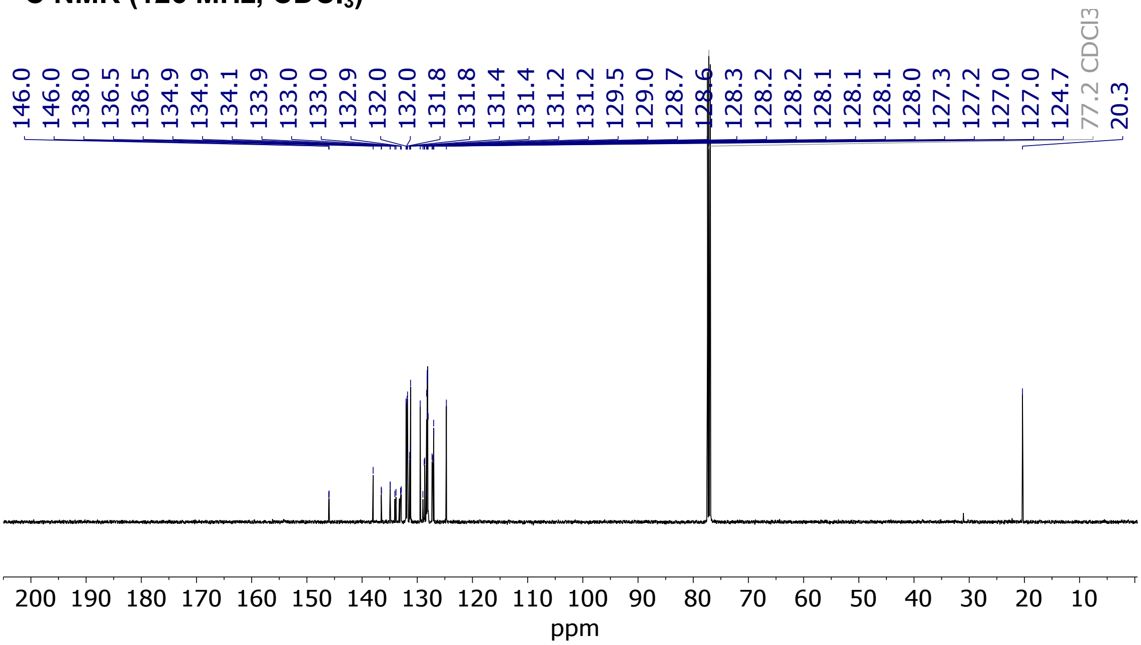

^{1}H NMR (500 MHz, $CDCl_3$)

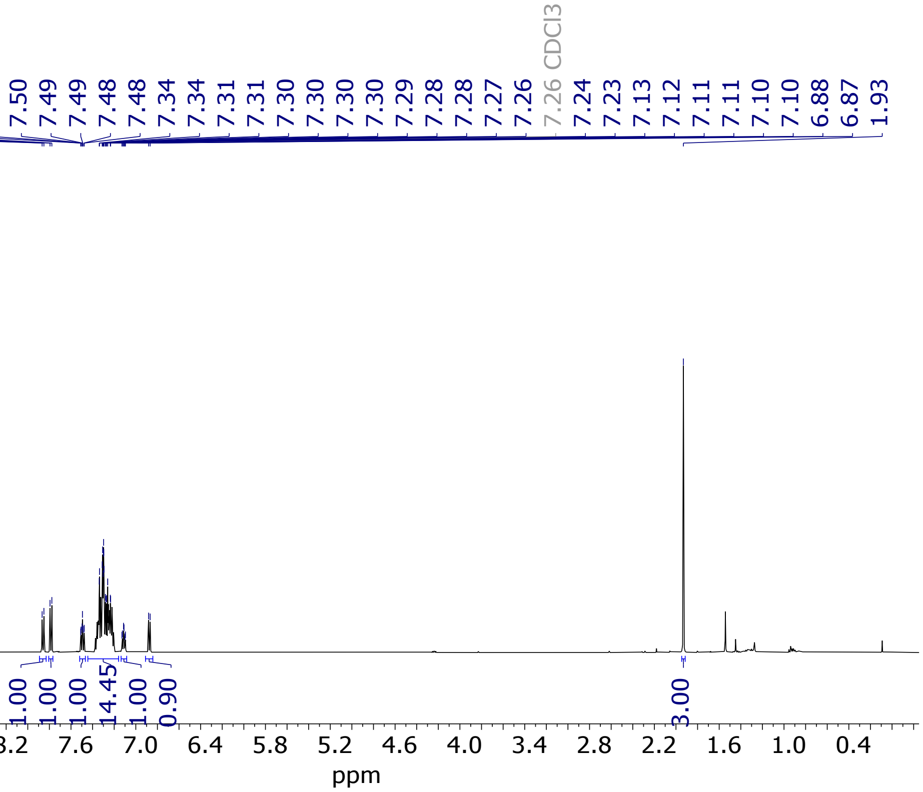

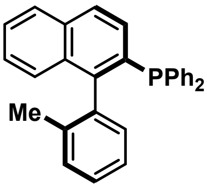

6

DEPT-135

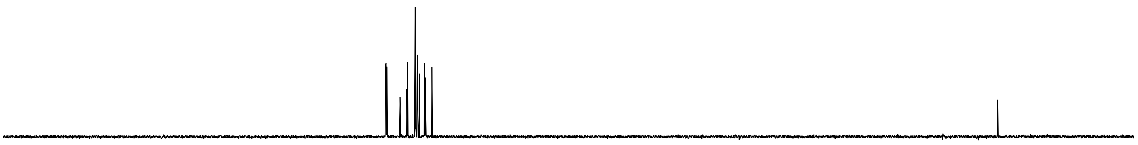

^{13}C NMR (126 MHz, $CDCl_3$)

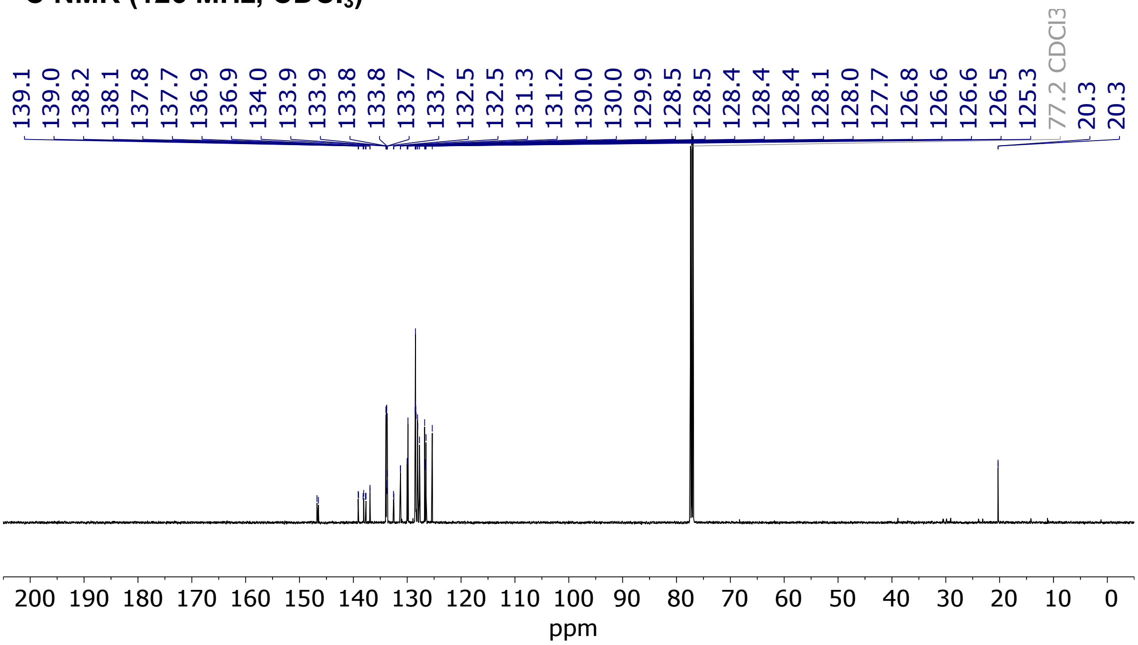

^{1}H NMR (300 MHz, $CDCl_3$)

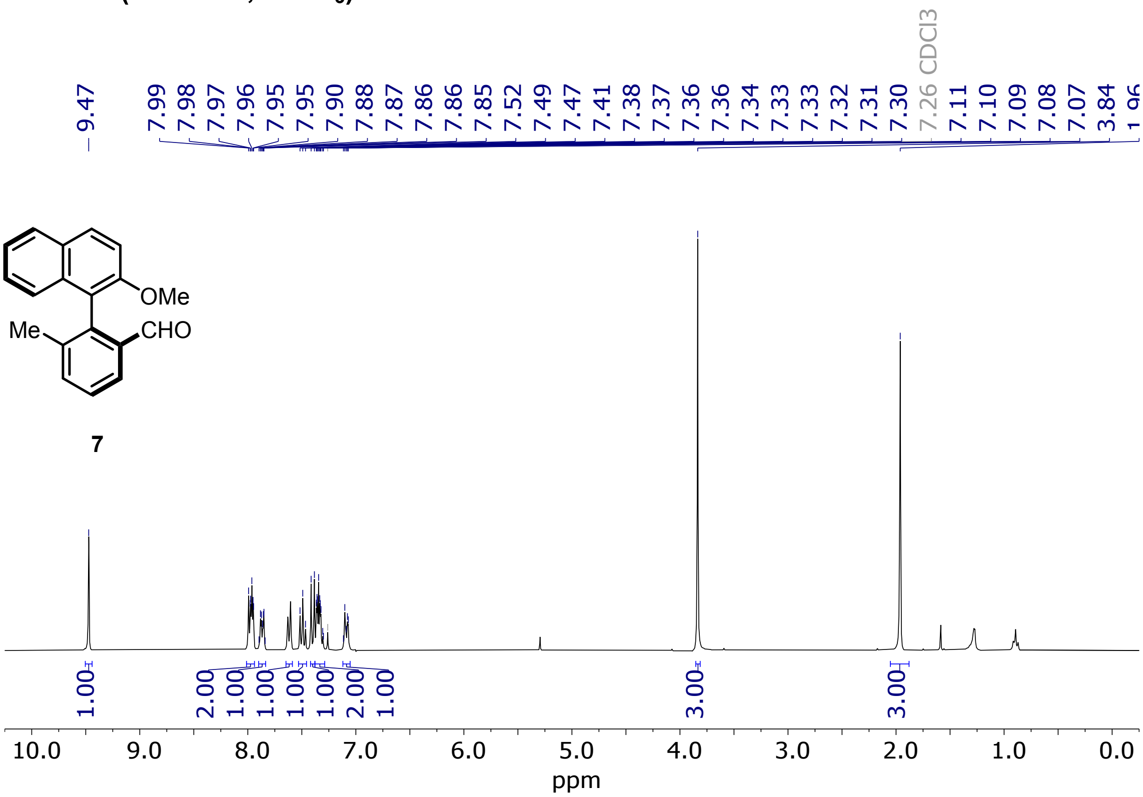

DEPT-135

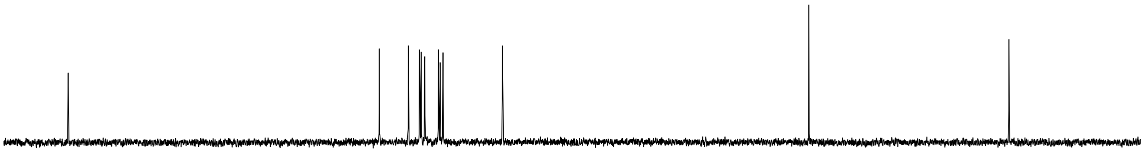

^{13}C NMR (75 MHz, $CDCl_3$)

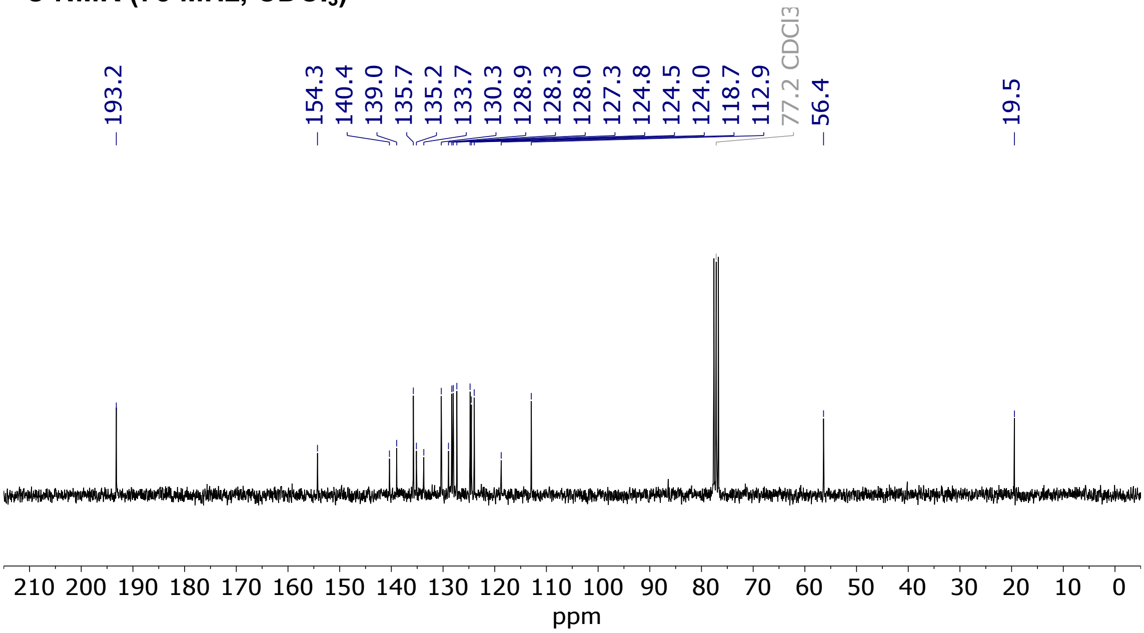

^{1}H NMR (300 MHz, $CDCl_3$)

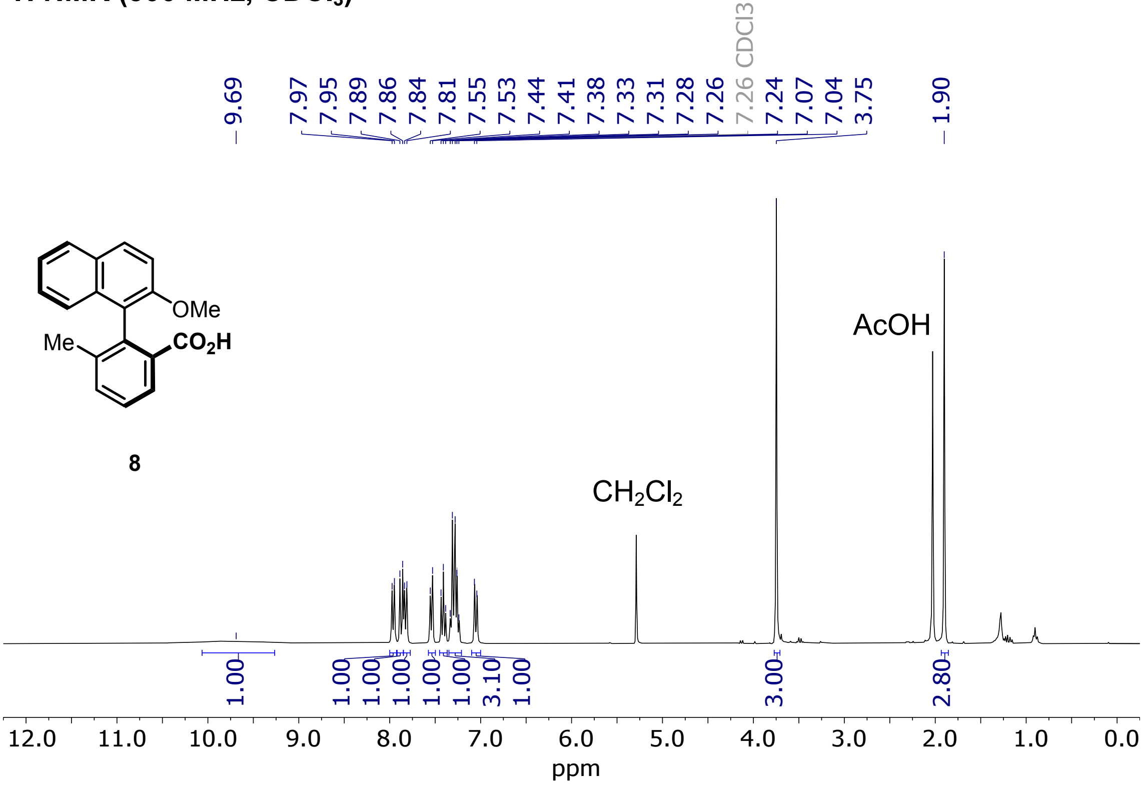

DEPT-135

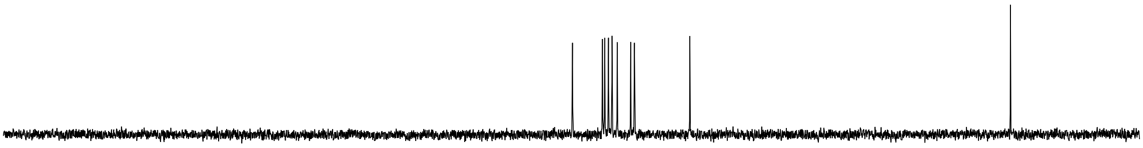

^{13}C NMR (75 MHz, $CDCl_3$)

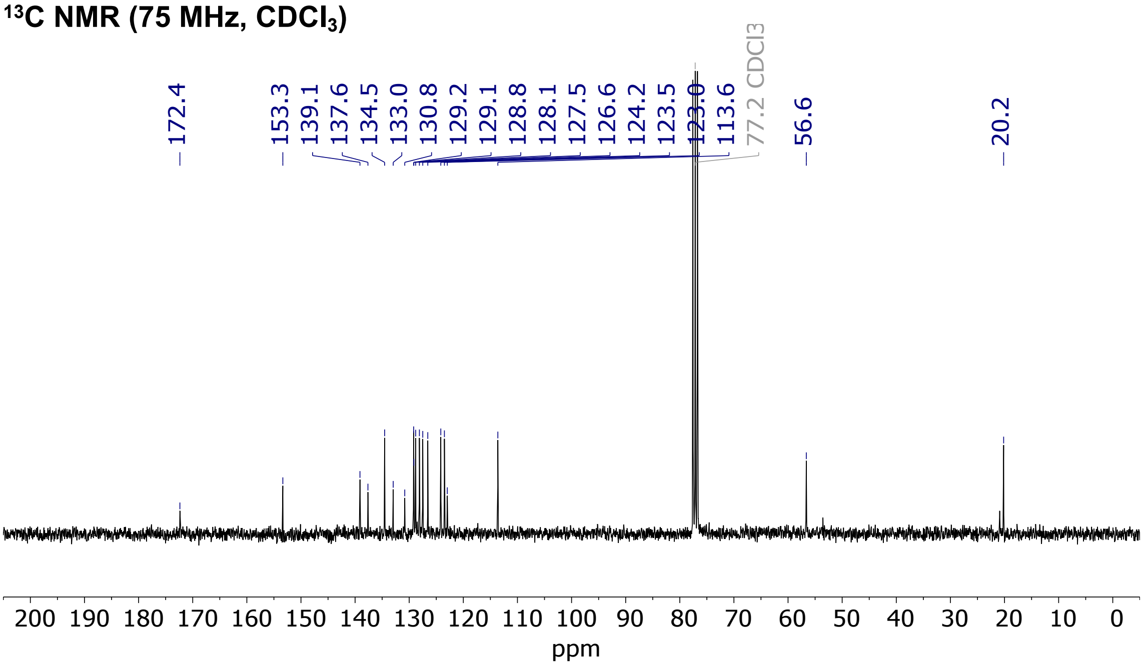

^{1}H NMR (300 MHz, $CDCl_3$)

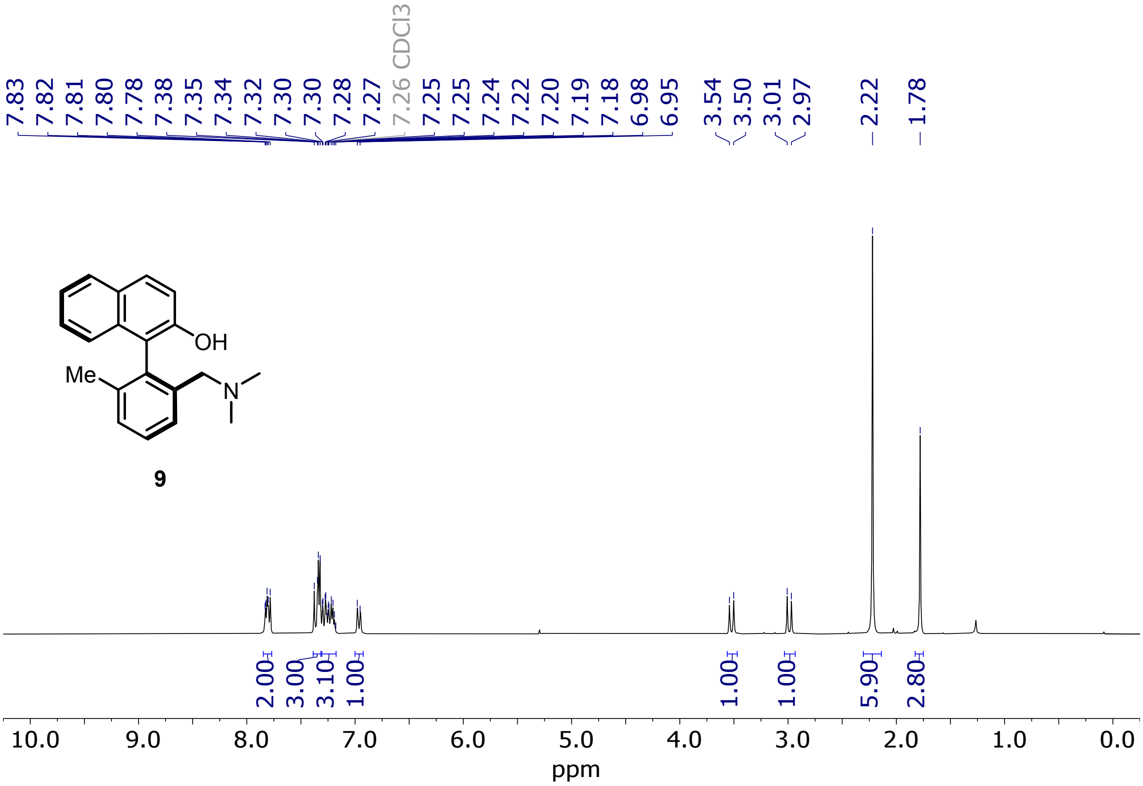

DEPT-135

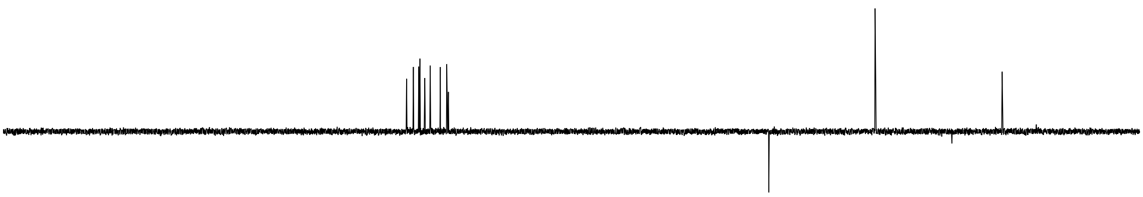

^{13}C NMR (75 MHz, $CDCl_3$)

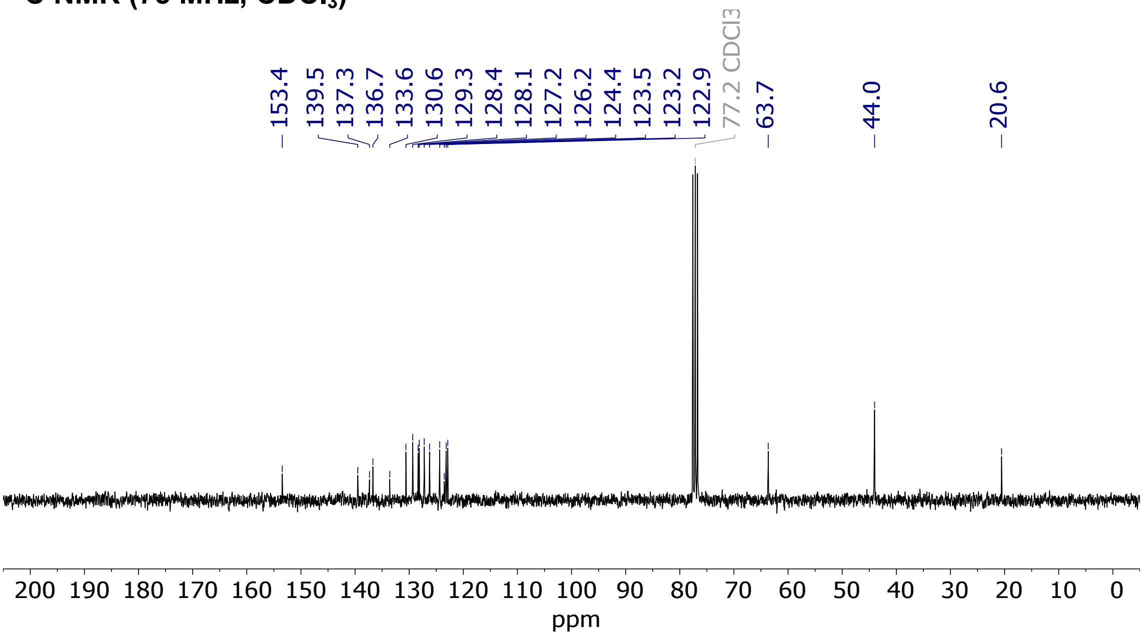

^{1}H NMR (300 MHz, CDCl$_3$)

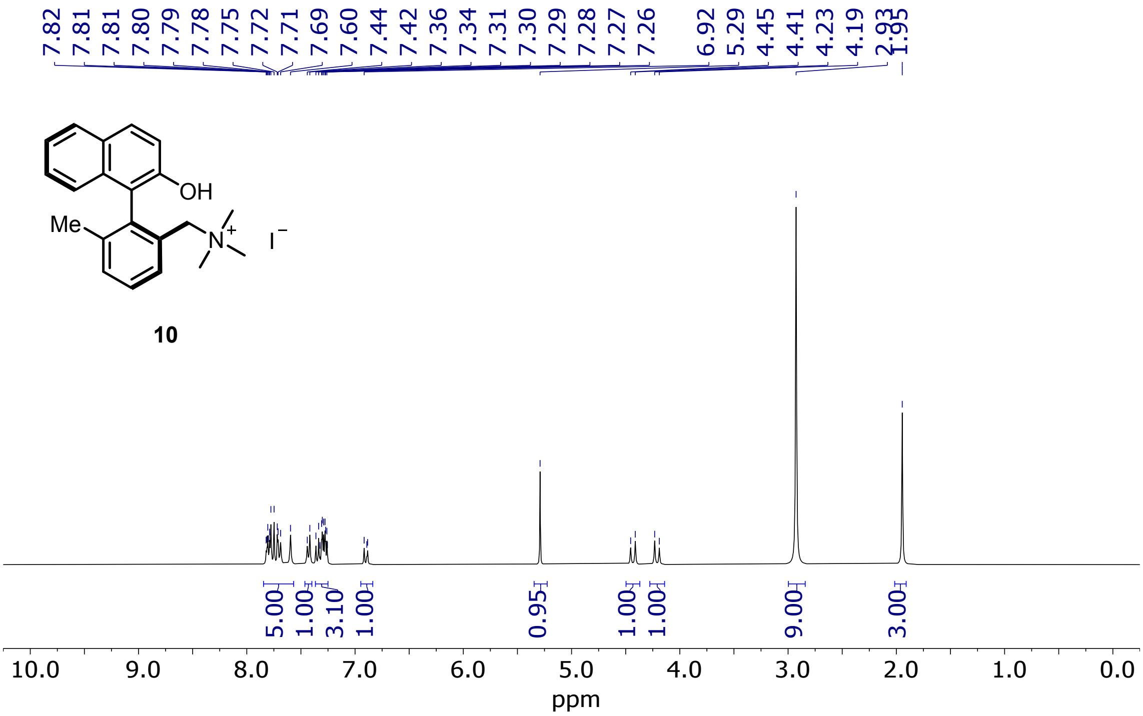

DEPT-135

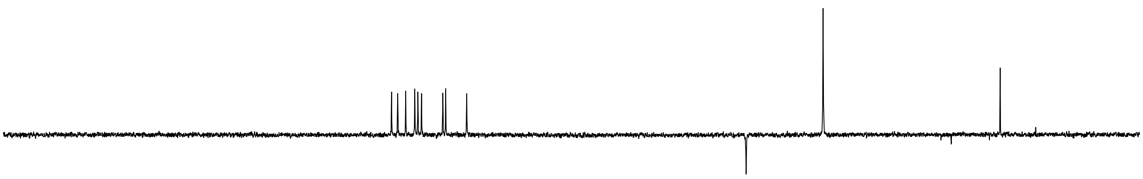

^{13}C NMR (75 MHz, CDCl$_3$)

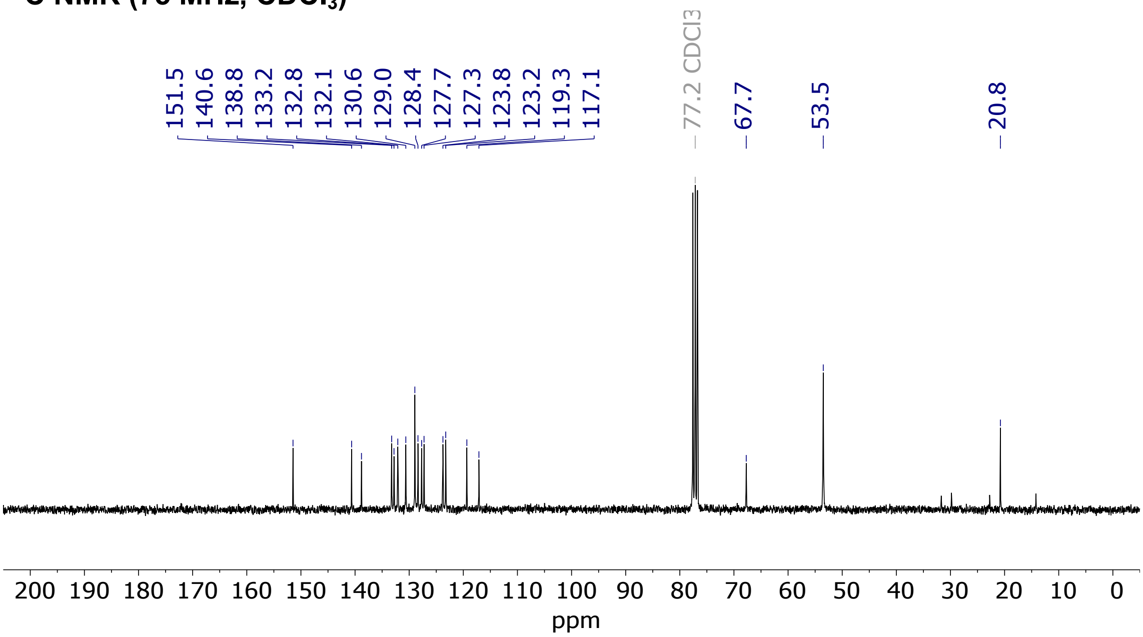

^{1}H NMR (300 MHz, $CDCl_3$)

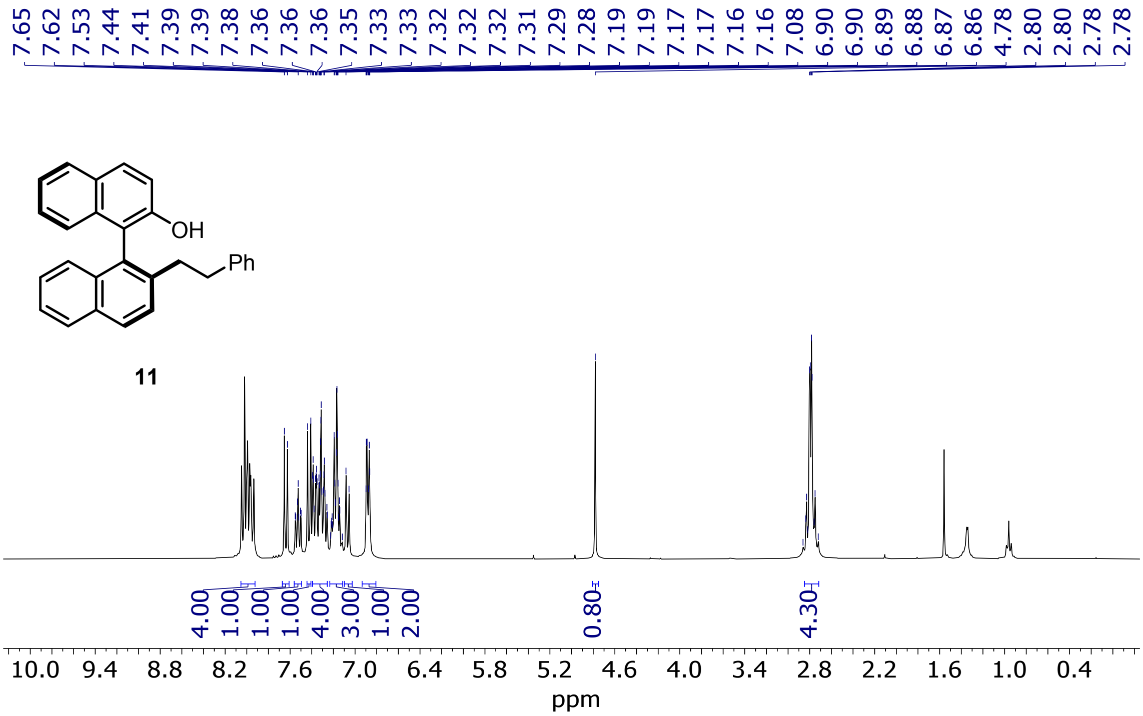

DEPT-135

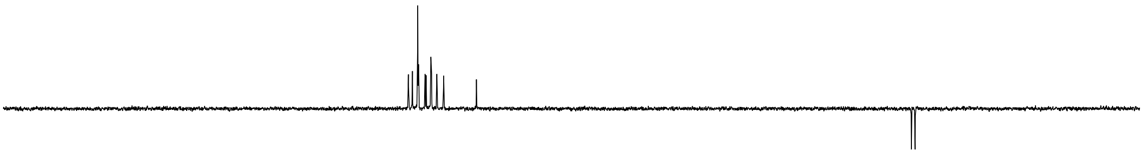

^{13}C NMR (75 MHz, $CDCl_3$)

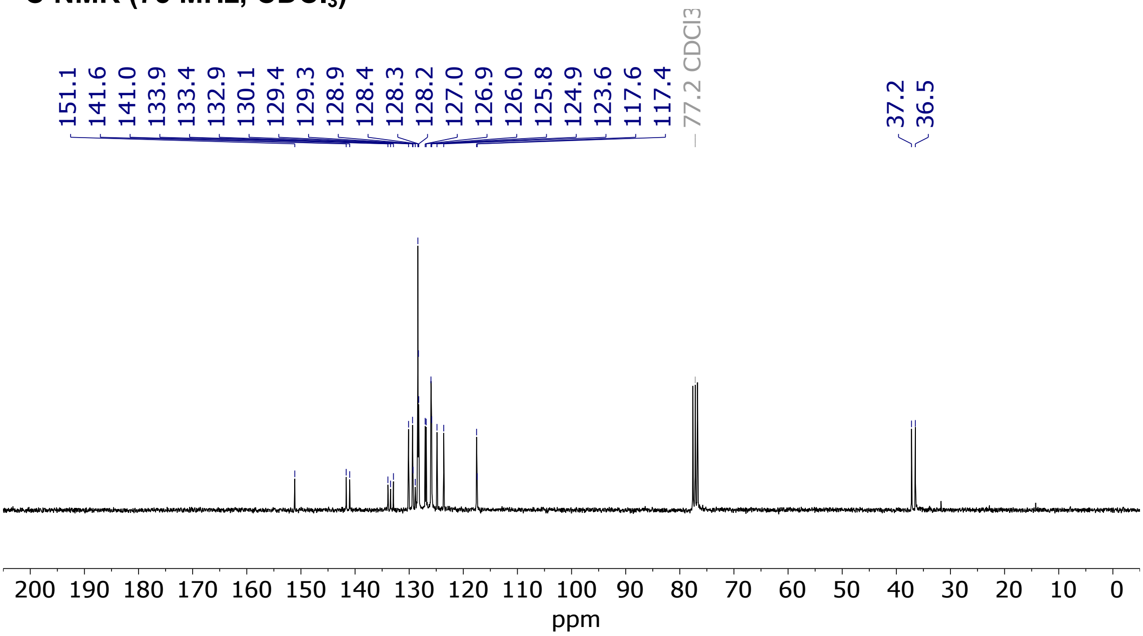

^{1}H NMR (300 MHz, $CDCl_3$)

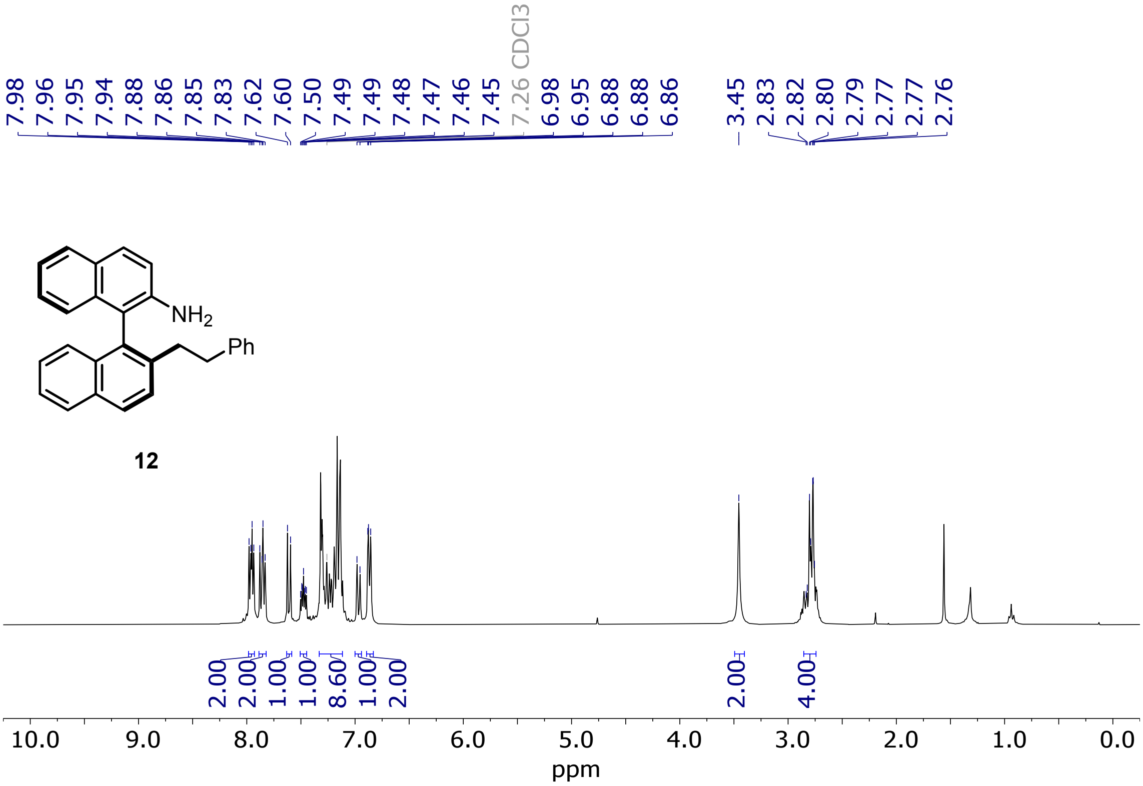

12

DEPT-135

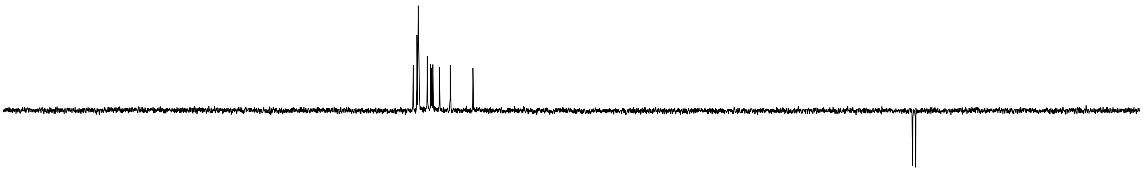

^{13}C NMR (75 MHz, $CDCl_3$)

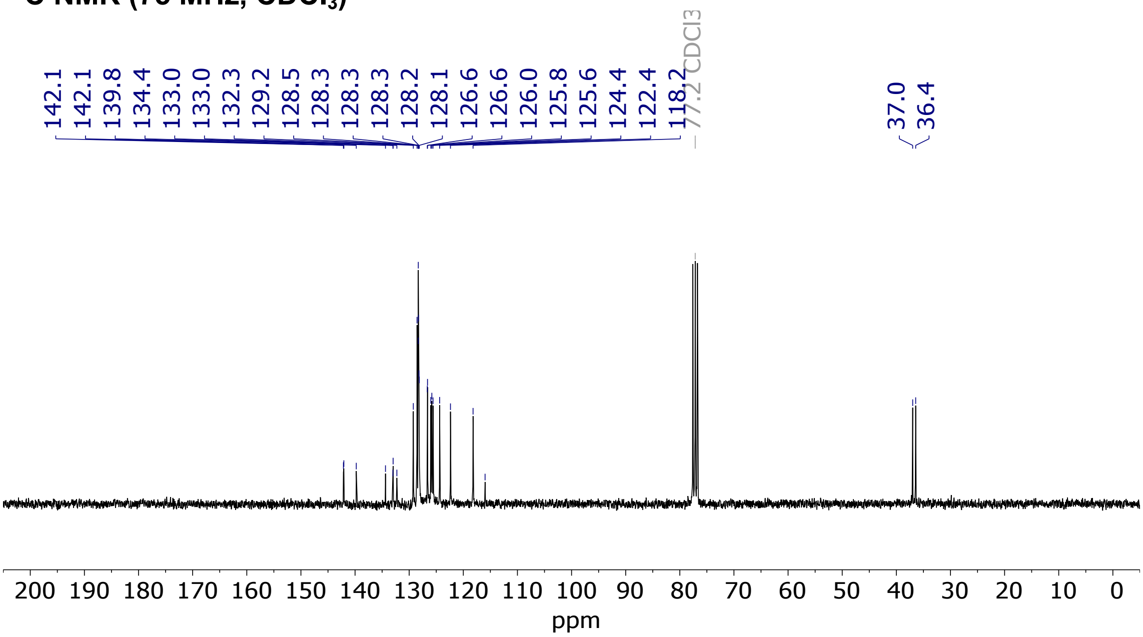

Supplement: Supplementary file 1 — cs3c03867_si_001.pdf [file cs3c03867_si_001.pdf]
